# 5′-Palmitate Lipid and Internal LNA-Piperidyl Triesters Enhance the RNA Affinity and Activity of Splice-Switching Oligonucleotides

**DOI:** 10.1021/jacs.6c07448

**Published:** 2026-06-09

**Authors:** Debashis Dhara, Tom Brown

**Affiliations:** Chemistry Research Laboratory, Department of Chemistry, 6396 University of Oxford, 12 Mansfield Road, Oxford OX1 3TA, U.K.

## Abstract

Therapeutic oligonucleotides represent a paradigm shift in medicine, but their modest therapeutic index remains a major obstacle. While chemical modification is essential for stability and cell uptake, only limited diversity is represented in oligonucleotides currently in the clinic. In fact, phosphorothioate and morpholino (PMO) are the only backbone modifications used clinically. Alternative phosphorus-based oligonucleotide backbones such as alkyl phosphonate and phosphoramidate have been explored, but their synthesis is challenging. In this work, we present the synthesis, physical, and biological properties of oligonucleotides containing THP and piperidyl phosphothiotriesters combined with LNA, 2′-OMe, 2′-MOE, and 2′-F ribose sugar derivatives. The LNA triester analogues increase duplex stability compared to phosphorothioate, while 2′-OMe, 2′-MOE, and 2′-F ribose phosphothiotriesters reduce it. Oligonucleotides containing LNA-piperidyl triester backbones enhance duplex stability to levels far beyond the classical phosphorothioate linkage, and their gymnotic splice-switching activity is significantly enhanced when conjugated to palmitate lipid.

## Graphical abstract

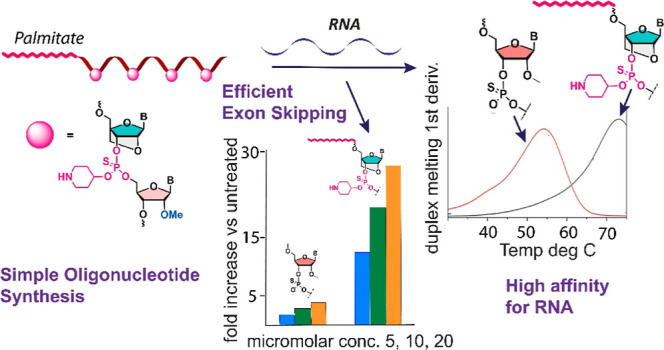

## Introduction

Single-stranded antisense oligonucleotides (ASOs),^1^ splice-switching oligonucleotides (SSOs),^2^ and double-stranded small interfering RNAs (siRNAs)^3,4^ represent a highly promising class of genetic medicines, directly targeting RNA to address the fundamental causes of disease.^1,5–7^ They function by modulating pre-mRNA splicing^8^ or by recruiting RNase H^9^ or Argonaute^10^ for mRNA degradation. SSOs partially restore protein function and are used to treat genetic disorders such as Duchenne muscular dystrophy (DMD)^11^ and spinal muscular atrophy (SMA).^12,13^ Oligonucleotides must be chemically modified for therapeutic applications,^14^ and substituents at the ribose sugar, namely, 2′-*O*-methyl (2′-OMe),^15^ 2′-*O*-(2-methoxyethyl) (2′-MOE),^16^ 2′-fluoro (2′-F),^17^ 2′-*O*-[2-(methylamino)-2-oxoethyl] (NMA),^18^ locked nucleic acid (LNA),^19,20^ constrained ethyl (cEt),^21^ and tricyclo, are well studied.^22^ These analogues improve the chemical and enzymatic stability, hence the overall therapeutic index of oligonucleotide drugs.

In contrast to modified sugars, a limited number of phosphodiester backbone modifications have gained traction. Most therapeutic oligonucleotides feature the anionic phosphorothioate (PS) internucleotide linkage, which has transformed the field.^23^ It greatly improves the nuclease stability and cellular uptake via albumin binding^24^ but slightly reduces RNA target-binding affinity. Phosphorothioate-modified oligonucleotides interact with a range of other proteins,^25–27^ and this has been associated with toxicity.^28^ Reducing the anionic charge of therapeutic oligonucleotides changes their physical and biological properties including RNA affinity and cell penetration.^29–32^ Phosphorodiamidate morpholino oligomers (PMOs), which are charge neutral/cationic (Figure 1A), are used for the treatment of DMD.^7^ Peptide nucleic acids (PNAs), which are also charge neutral (Figure 1B), have nucleobases linked by an *N*-(2-aminoethyl) glycine backbone.^33^ PNA has applications in molecular diagnostics^34^ but has not entered the clinic partly due to low aqueous solubility and poor cellular uptake. PNA forms extremely stable duplexes with complementary RNA, whereas PMOs form duplexes that are similar in stability to RNA duplexes. Studies on PMO and PNA are limited due to their demanding chemical synthesis and restricted commercial availability. Artificial nonphosphorus charge-neutral backbones, such as amide,^35–39^ sulfamate,^40,41^ and triazole,^42^ have been proposed as alternatives but have not yet been used as drug candidates. Such modifications are introduced via amide coupling or as dinucleotide phosphoramidites; hence, 8 or 16 different building blocks are needed to make any base sequence, which makes synthesis laborious. In contrast, phosphorus-based (*P*-based) charge-neutral backbones such as alkyl phosphonate, phosphotriester, and phosphoramidate^43^ are synthesized using more conventional nucleoside phosphoramidite monomers. Mesyl phosphoramidate^44^ and phosphoryl guanidine^45,46^ have also been reported as alternatives to the PS internucleoside linkage (Figure 1A). Mesyl phosphoramidate oligonucleotides recruit RNase H and have some advantages over phosphorothioates in terms of RNA affinity and nuclease stability. Splice-switching oligonucleotides currently in the clinic are all based on PMO or MOE-PS chemistry. A study in an SMA mouse model revealed that a Nusinersen analogue with MOE sugars and a phosphorothioate backbone is more active than the PMO version when administered by subcutaneous injection.^47^ However, in an mdx mouse model, PMOs have improved dystrophin exon 23 skipping activity via intramuscular injection compared to oligonucleotides with the 2′-OMe PS backbone.^48^ Thus, it is unclear whether a charge-neutral or anionic backbone, or a combination of both, will be more effective in any particular exon-skipping application. Synthetic methodology to produce oligonucleotides with chimeric anionic/charge-neutral and cationic backbones is therefore of interest.

**Figure 1:**
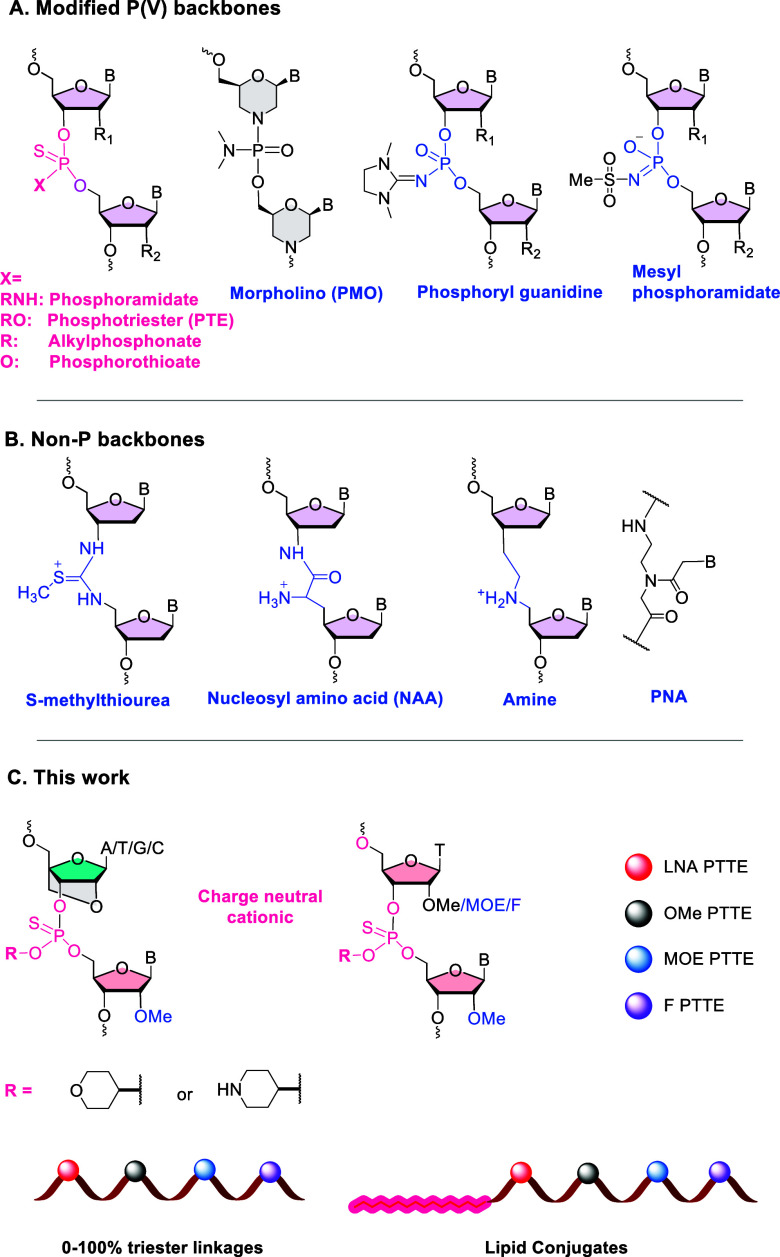
(**A**) Charge neutral/cationic *P*-backbones. (**B**) Non-P backbones. (**C**) Current work on triester oligonucleotides and their lipid conjugates.

Lipid conjugation has emerged as an important strategy to improve the pharmacological properties of therapeutic oligonucleotides. Coupling cholesterol, tocopherol, or fatty acids to oligonucleotides increases their interactions with plasma proteins. This prolongs circulation half-life and facilitates cell uptake, thereby increasing the potency of ASOs^49^ and siRNAs.^50^ The activity of lipid ASO conjugates correlates with their affinity for albumin,^51^ which increases with lipid chain length, peaking at C16–C22. The presence of double bonds in the lipids has minimal effect on activity.^51^ Palmitate conjugation significantly improves plasma exposure, interstitial delivery, and potency of ASOs targeting muscle,^52^ and cholesterol-conjugated siRNAs also have improved delivery properties.^53^ In addition, docosahexaenoic acid (DHA) and α-tocopherol oligonucleotide conjugates show enhanced delivery to muscle and the central nervous system.^54^ Despite the above studies, there is only limited data on lipid conjugates of oligonucleotides which have reduced anionic charge.

Cationic groups have previously been introduced on the nucleobase, sugar, and phosphate backbone.^55–58^ Their location is critical, with positive charges on the phosphate backbone having the greatest effect on biological properties. The aforementioned morpholino and phosphoramidate oligonucleotides fall into this class (Figure 1A). However, they are challenging to synthesize, particularly on a large scale. Nonphosphorus-based cationic backbones including amine,^58^ guanidinium, *S*-methylthiourea motifs,^59^ and nucleosyl amino acid (NAA)^60^ are also synthetically complex (Figure 1B), and limited biological data are available on these analogues. In contrast, here we report a simple approach to access the phosphorus-based piperid-4-yl triester linkage as an alternative cationic backbone (Figure 1C).

## Results and Discussion

Our overall aim is to improve the cell uptake and tissue distribution of therapeutic oligonucleotides. With this in mind, we recently developed a straightforward method for synthesizing LNA-alkyl phosphothiotriester (PTTE) and LNA-phosphotriester (PTE) oligonucleotides. The presence of a secondary alkyl group in the triester is important for chemical stability. These modified oligonucleotides retain biological activity and provide a versatile chemical template for functionalization, including ligand attachment via click chemistry.^61,62^ Building on this work, we now present new experimental data on the properties of other ribose sugar modifications combined with PTTE linkages. We have synthesized 2′-OMe, 2′-MOE, and 2′-F PTTE oligonucleotides containing tetrahydropyran-4-yl and piperid-4-yl triesters (Figure 1C) as well as oligonucleotides containing multiple LNA piperidyl triester linkages. The piperidyl triester can carry a positive charge by protonation, further reducing the overall anionic nature of the oligonucleotide (piperidine has a p*K*_a_H value of ∼11.2). We have also studied PTTE SSOs that are functionalized at the 5′-position with palmitate lipid, and we have employed cleavable and noncleavable linkers between the oligonucleotide and the lipid.

### Chemical Synthesis

In our previous work, oligonucleotides containing charge-neutral THP phosphothiotriester linkages displayed favorable physical and biological properties. In this study, we compare them with structurally similar cationic piperidyl analogues. The required P­(III) reagents **4** and **5** were synthesized by reacting tetrahydropyran-4-ol (**1**) and *N*-trifluoroacetyl 4-hydroxypiperidine (**2**) with bis­(diisopropylamino)­chlorophosphine (**3**) in the presence of triethylamine (Scheme 1). The 2′-modified thymidine nucleosides **6**, **7**, and **8** were reacted with THP-P­(III) reagent **4** in the presence of tetrazole to give modified thymidine phosphoramidites **9**, **10**, and **11**, respectively, in 65%–83% yield.

**Scheme 1:**
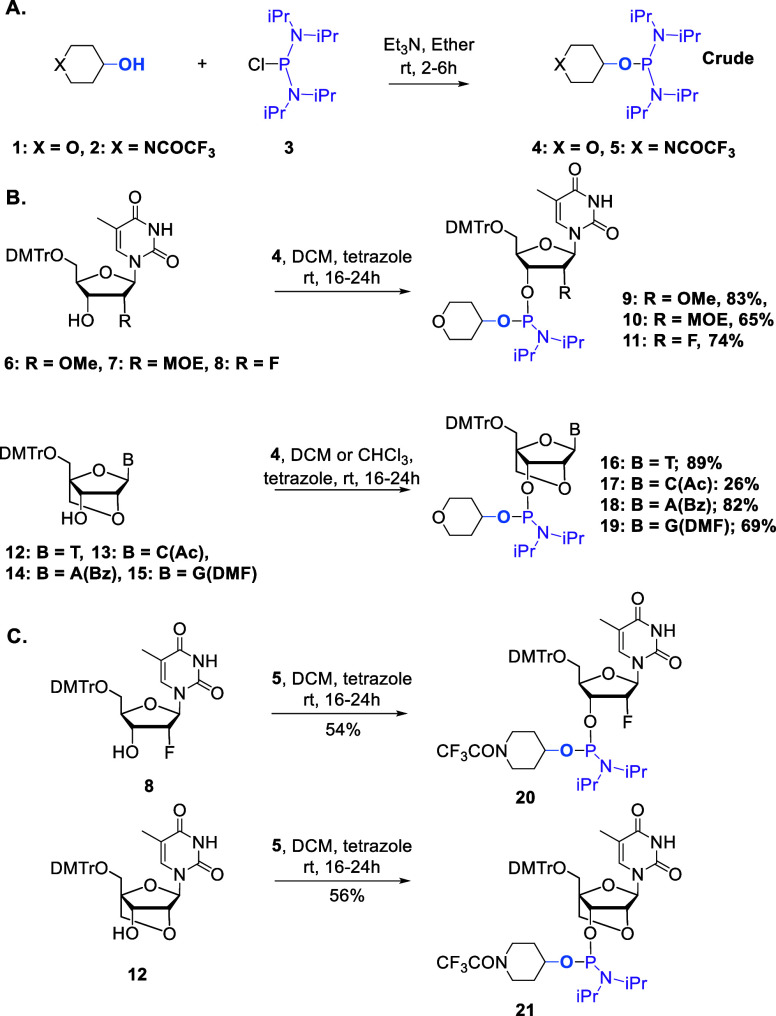
**(A)** Synthesis of Phosphoramidite Reagents. (**B**) Synthesis of Tetrahydropyran-4-yl Phosphoramidites **9–11** and **16–19**. (**C**) Synthesis of Piperid-4-yl Phosphoramidites **20** and **21**. DMTr = 4,4′-Dimethoxytrityl

Following the same procedure, 5′-DMT-protected locked nucleosides **12–15** were converted to phosphoramidite monomers **16–19**, respectively, with some improvements on our previous syntheses.^61^ The 2′-F nucleoside **8** was reacted with P­(III) reagent **5** to give piperidyl phosphoramidite **20** in 54% yield, and locked nucleic acid thymidine **12** was reacted with reagent **5** in the presence of tetrazole to give the LNA-piperidyl phosphoramidite **21**. The nucleoside phosphoramidites were purified by silica-gel flash chromatography to ensure efficient coupling during solid-phase oligonucleotide synthesis. However, for the cytidine and guanosine phosphoramidites **17** and **19**, silica-gel chromatographic purification led to some decomposition. Therefore, purification was done by passage through a short bed of silica. Full experimental protocols including detailed characterization of all compounds are in the Supporting Information (SI 1.2).

### Oligonucleotide Synthesis

Phosphoramidite monomers **9–11** and **16–21**, along with commercially available protected 2′-*O*-methyl and 2′-*O*-methoxyethyl derivatives of A, G, C, and U (Figure S2), were used to synthesize the oligonucleotides listed in Table 1. Solid-phase synthesis of oligonucleotides was carried out on the 1.0 μmol scale on an ABI394 DNA synthesizer, and monomer coupling efficiency was measured conductometrically by the release of DMT+ (Figures S143 and S144). EDITH (3-ethoxy-1,2,4-dithiazole-5-one) was used as a sulfurizing reagent and iodine/H_2_O for P­(III) oxidation. All oligonucleotides were cleaved from the solid support and deprotected with either a 1:1 mixture of THF–ethylene diamine (EDA) for 2 h at RT or 35% aqueous ammonia at RT for 16–24 h. Ammonia deprotection was used for oligonucleotides containing N4-benzoyl-C­(Me) because EDA gives an undesired adduct of this nucleobase. All oligonucleotides were purified by reversed-phase HPLC and analyzed by UPLC-MS (SI 2.0, SI Table T1). Oligonucleotide yields are given in SI Table T2.

**Table 1:**
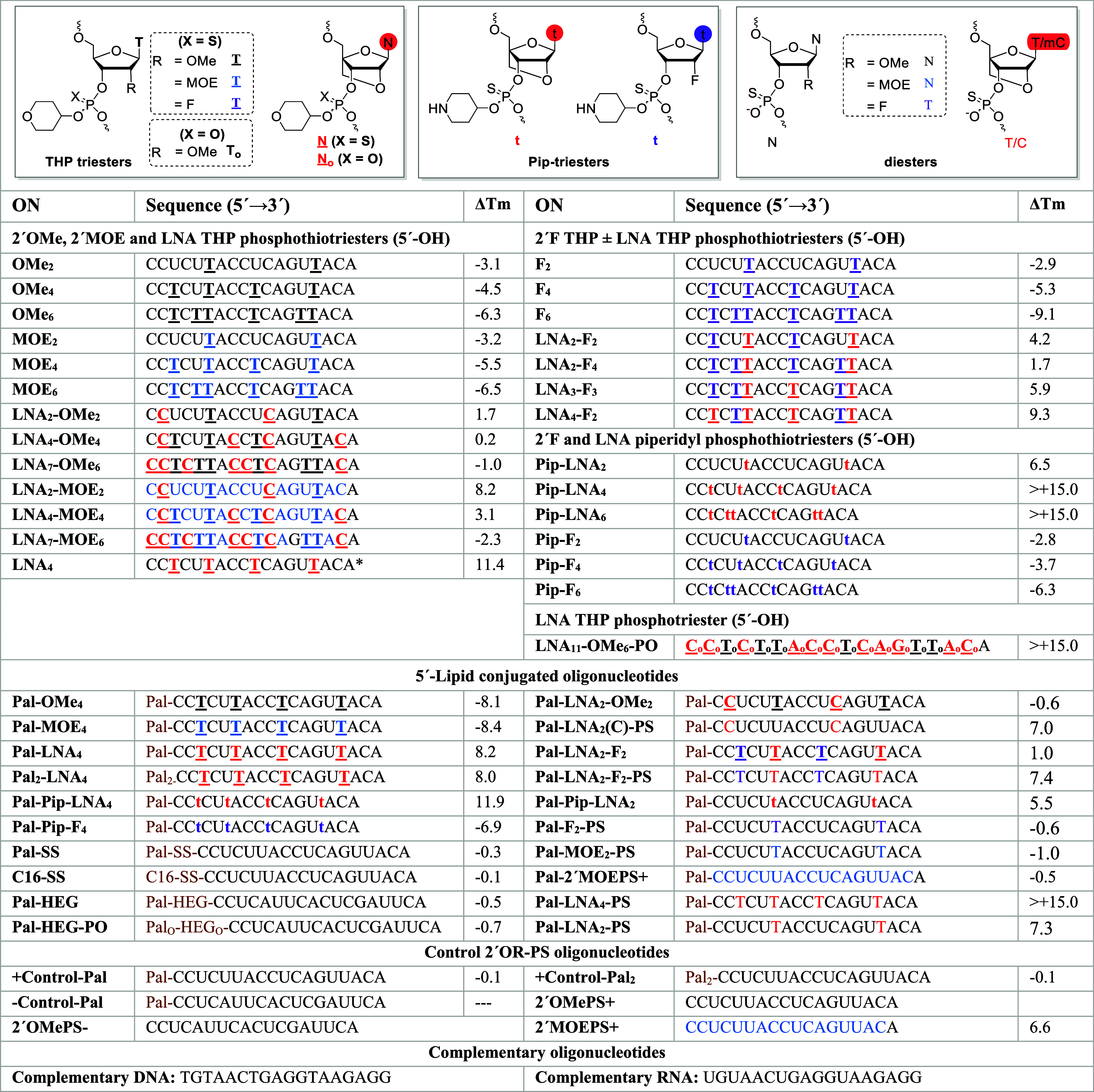
Duplex Melting Temperatures (in °C) against Complementary RNA^a^

a Nucleosides in black have 2′-OMe sugars. Nucleosides in blue have 2′-MOE sugars (O–CH_2_CH_2_OCH_3_). Nucleosides in red have locked nucleic acid sugars. Nucleosides in purple have 2′-F sugars. Nucleosides in bold underlined are tetrahydropyran-4-yl phosphothiotriesters. Nucleosides in red lowercase Are LNA-piperid-4-yl phosphothiotriesters. Nucleosides in purple lowercase are 2′-F-piperid-4-yl phosphothiotriesters. ‘o’ = phosphodiester linkage. For MOE sugars, C = 5-methyl cytidine. Pal = palmitate; SS = disulfide linker (Figure 2). Oligonucleotides that do not contain triesters have either ‘PS’ or ‘Control’ in their names. The LNA-C bases in **Pal-LNA**_**2**_ **(C)-PS** are methylated at position 5. Full chemical structures of oligonucleotides are in SI 8.0. ΔTm = difference in duplex melting temperature against RNA compared to the 2′-OMe PS positive control (2′-OMePS+). Tm of 2′-OMePS+ control vs RNA = 61.3 °C. Melting temperatures were recorded in 10 mM Na-phosphate buffer, pH = 7.0, containing 25 mM NaCl. Tm values are averages of three experiments with an error of ±0.50 °C. Nd = not determined. * = data from Dhara et al. 2024.^61^

The THP PTTE oligonucleotides **OMe**_**2**_, **OMe**_**4**_, and **OMe**_**6**_ and **MOE**_**2**_, **MOE**_**4**_, and **MOE**_**6**_ were produced in 36%–57% yields after purification. We also synthesized mixed LNA/2′-OMe THP PTTE oligonucleotides **LNA**_**2**_ **-OMe**_**2**_, **LNA**_**4**_ **-OMe**_**4**_, and **LNA**_**7**_ **-OMe**_**6**_ and their LNA/2′-MOE equivalents **LNA**_**2**_ **-MOE**_**2**_, **LNA**_**4**_ **-MOE**_**4**_, and **LNA**_**7**_ **-MOE**_**6**_ in 41%–53% yields. Oligonucleotides **LNA**_**4**_ **-OMe**_**4**_ and **LNA**_**4**_ **-MOE**_**4**_ have 8 neutral PTTE linkages, i.e., a 47% reduced charge, and **LNA**_**7**_ **-OMe**_**6**_ and **LNA**_**7**_ **-OMe**_**6**_ have 13 such linkages, a 76% reduction in charge. We synthesized a fully charge-neutral phosphotriester oligonucleotide **LNA**_**11**_ **-OMe**_**6**_ **-PO**, which was isolated in sufficient quantity for physical studies (Table 1). We could not purify this oligonucleotide by HPLC; therefore, we partially purified it by spin column (3 kD molecular weight cutoff) and confirmed its integrity by MALDI-MS. Analysis by ESI^–^ UPLC-MS was not possible due to the absence of a negative charge. Currently, we are optimizing the purification and analysis of fully charge-neutral triester oligonucleotides.

Oligonucleotides **F**_**2**_, **F**_**4**_, and **F**_**6**_ containing 2′-F THP PTTE linkages and **LNA**_**2**_ **-F**_**2**_, **LNA**_**2**_ **-F**_**4**_, **LNA**_**3**_ **-F**_**3**_, and **LNA**_**4**_ **-F**_**2**_ with mixed 2′-F/LNA THP phosphothiotriester linkages were obtained in good yields (52%–61%). Next, we focused on synthesizing piperidyl PTTE oligonucleotides. Alkylamines in the PTTE backbone should be protonated in biological systems, reducing the overall anionic charge of oligonucleotides to potentially improve cell uptake. In this study, the piperidyl triester was chosen as it is derived from the secondary alcohol piperidin-4-ol and should therefore form chemically stable triester linkages.^61^ LNA PTTE oligonucleotides **Pip-LNA**_**2**_, **Pip-LNA**_**4**_, and **Pip-LNA**_**6**_ were obtained in similar yields to the LNA THP counterparts, but slight cleavage of the piperidyl group was observed. We also synthesized **Pip-F**_**2**_, **Pip-F**_**4**_, and **Pip-F**_**6**_, which contain the 2′-fluororibose sugar. Their yields were lower than those of the LNA-Pip PTTE oligonucleotides (20%–33%), possibly due to the smaller size and electronegativity of the F atom leading to some cleavage of the PTTE linkages during oligonucleotide deprotection.

To explore the synergy between 5′-lipids and triester linkages on the properties of PTTE oligonucleotides, a series of 5′-monopalmitate conjugates were prepared (Figure 2, Table 1), namely, **Pal-OMe**_**4**_, **Pal-MOE**_**4**_, **Pal-LNA**_**4**_, **Pal-Pip-LNA**_**4**_, **Pal-Pip-F**_**4**_, and **Pal-LNA**_**4**_ **-PS**. THP groups are present in **Pal-LNA**_**4**_, whereas **Pal-LNA**_**4**_ **-PS** is an LNA phosphoro­thioate control with no triesters. A 5′-bis-palmitate conjugate **Pal**_**2**_ **-LNA**_**4**_ was also prepared using doubler phosphoramidite **26** and palmitate phosphoramidite **22**.

**Figure 2:**
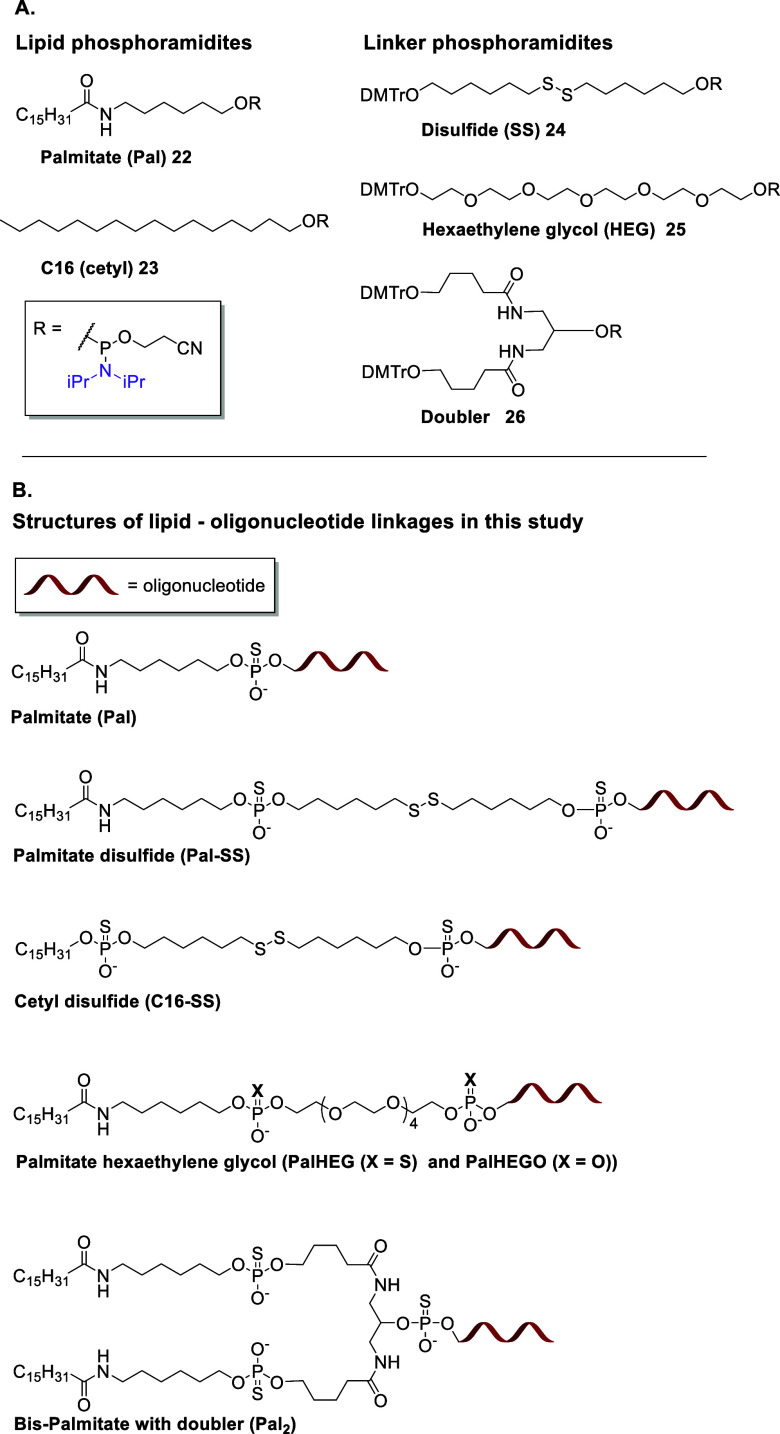
**(A)** Commercial phosphoramidite monomers used in the synthesis of lipid conjugate oligonucleotides. **(B)** Structures of the lipid–oligonucleotide conjugates.

Oligonucleotides **Pal-SS**, **C16-SS**, **Pal-HEG**, and **Pal-HEG-PO** are 5′-lipid conjugates of the **2′OMePS+** oligonucleotide control and differ in the structures of the lipid and linker. **C16-SS** has a cetyl lipid and C6-disulfide linker, which were added via monomers **23** and **24**, whereas **Pal-SS** was constructed by coupling palmitate monomer **22** to disulfide monomer **24**. For **Pal-HEG** and **Pal-HEG-PO**, the palmitate lipid is attached to the oligonucleotide by a hydrophilic hexaethylene glycol (HEG) linker **25**. **Pal-HEG-PO** has phosphodiester linkages instead of phosphorothioates between the HEG, the lipid, and the oligonucleotide. Phosphodiester and disulfide linkages are cleavable in cells, giving the possibility of the oligonucleotide being liberated from the lipid after cell uptake. This might be expected to affect cell trafficking. The oligonucleotide **+Control-Pal** is the 5′-palmitate version of positive control **2′OMePS+**, **+Control-Pal**_**2**_ is its bis-palmitate version, and **-Control-Pal** is the palmitate analogue of the negative control **2′OMePS–**.

Three additional pairs of palmitate oligonucleotides were made in which one of each pair has triester linkages and the other does not. This enabled us to compare the effects of the THP and piperidine PTTE linkages with the equivalent purely phosphorothioate oligonucleotides. The pairs are **Pal-LNA**_**2**_ **-OMe**_**2**_ and **Pal-LNA**_**2**_ **(C)-PS**, **Pal-LNA**_**2**_ **-F**_**2**_ and **Pal-LNA**_**2**_ **-F**_**2**_ **-PS**, and **Pal-Pip-LNA**_**2**_ and **Pal-LNA**_**2**_ **-PS**. We also synthesized three other nontriester palmitate oligonucleotides, **Pal-F**_**2**_ **-PS**, **Pal-MOE**_**2**_ **-PS**, and **Pal-2′MOEPS+**, to further reveal the effects of the 2′-F and 2′-MOE sugars. **Pal-2′MOEPS+** was obtained as a mixture of ethylenediamine adducts (1 to 2 additions on average) formed during deprotection with EDA/THF. This is due to the presence of the N4-benzoyl-protecting group on the commercial C­(Me) MOE nucleoside.

### Duplex Stability

#### Effects of LNA-, 2′-OMe, 2′-MOE, and 2′-F THP-PTTE Linkages

Previously, we found that LNA THP-PTTE linkages increase duplex stability by approximately 2–3 °C per modification compared to **2′OMePS+**, which is ∼2 °C lower than the increase observed for LNA-PS phosphodiester linkages.^61^ Duplex stability strongly depends on alkyl groups in the PTTE backbone, but the nature of the sugar is also important; 2′-OMe THP-PTTE linkages reduce duplex stability by 1–2 °C per modification against complementary RNA, as observed for **OMe**_**2**_, **OMe**_**4**_, and **OMe**_**4**_ (Table 1). A similar reduction was also observed for 2′-MOE THP-PTTE linkages in **MOE**_**2**_, **MOE**_**4**_, and **MOE**_**6**_. Oligonucleotides containing mixed sugars, **LNA**_**2**_ **-OMe**_**2**_ and **LNA**_**4**_ **-OMe**_**4**_, containing four and eight THP-PTTE linkages, respectively, showed ΔTm values of +1.7 °C and +0.2 °C compared to **2′OMePS+**, and incorporation of 13 THP-PTTE linkages in **LNA**_**7**_ **-OMe**_**6**_ resulted in only a 1.0 °C reduction in duplex stability. Hence, these chimeric oligonucleotides can have 76% THP-PTTE modifications without significant loss of duplex stability. In comparison, **LNA**_**2**_ **-MOE**_**2**_ and **LNA**_**4**_ **-MOE**_**4**_ exhibited ΔTm values of +8.2 °C and +3.1 °C, respectively, while the **LNA**_**7**_ **-MOE**_**6**_ combination reduced duplex stability slightly. The 2′-F THP-PTTE modification in oligonucleotides **F**_**2**_, **F**_**4**_, and **F**_**6**_ reduces duplex stability by 1.0–2.5 °C per modification, whereas chimeric **LNA**_**2**_ **-F**_**2**_, **LNA**_**2**_ **-F**_**4**_, **LNA**_**3**_ **-F**_**3**_, and **LNA**_**4**_ **-F**_**2**_ combinations stabilize the duplex, increasing ΔTm by 1.7–9.3 °C. The higher ratio of 2′-F THP-PTTE groups as in **LNA**_**2**_ **-F**_**4**_ reduces duplex stability (ΔTm = +1.7 °C), whereas incorporation of higher ratios of LNA THP-PTTE enhances stability (**LNA**_**4**_ **-F**_**2**_, ΔTm = +9.3 °C). Finally, the fully charge-neutral phosphotriester oligonucleotide **LNA**_**11**_ **-OMe**_**6**_ **-PO** displayed a high ΔTm against RNA (>15 °C).

#### Effect of Pip-PTTE Linkages

The **Pip-LNA**_**2**_, **Pip-LNA**_**4**_, and **Pip-LNA**_**6**_ combinations increase duplex stability significantly (by more than 3.0 °C per modification). This stabilization is attributed mostly to the LNA sugars, but clearly the piperidyl group is superior to THP. This can be seen by comparing **LNA**_**4**_ (+11.4 °C) with **Pip-LNA**_**4**_ (>+15 °C). Notably, of more than 20 different PTTE alkyl groups we have evaluated in this and our previous work (C3–C16 and several click conjugates),^61^ the piperidyl group has the greatest positive effect on duplex stability. This may be due to protonation of the nitrogen atom, which reduces anionic repulsion between phosphate groups in the duplex and possibly influences hydration and hydrogen bonding interactions. Combinations of piperidyl and 2′-fluororibose sugar in **Pip-F**_**2**_, **Pip-F**_**4**_, and **Pip-F**_**6**_ reduce duplex stability against RNA by 2.8–6.3 °C and are less destabilizing than the corresponding 2′-F THP analogues (**F**_**2**_, **F**_**4**_, and **F**_**6**_).

Overall, these findings indicate that both THP and Pip combined with LNA PTTE increase duplex stability relative to the **2′OMePS+** control, whereas 2′-OMe, 2′-MOE, and 2′-F PTTE linkages with THP and Pip reduce duplex stability. Therefore, LNA PTTE can be combined with 2′-OMe, 2′-MOE, or 2′-F PTTE linkages in an oligonucleotide to adjust its duplex stability to any desired level.

#### Effects of Combined Palmitate and PTTE Linkages

5′-Palmitate conjugation would not be expected to influence duplex stability. Indeed, the all-phosphorothioate palmitate control oligonucleotides are almost as stable as the nonpalmitate versions (∼−0.1 °C). However, the duplexes formed by the PTTE palmitate conjugates are ∼3 °C less stable than their nonpalmitate equivalents, suggesting interactions between the palmitate lipid and the charge-neutral triester moieties in the single strands. The ΔTm values of **Pal-LNA**_**2**_ **-OMe**_**2**_ vs **Pal-LNA**_**2**_ **(C)-PS** and **Pal-LNA**_**2**_ **-F**_**2**_ vs **Pal-LNA**_**2**_ **-F**_**2**_ **-PS** show that THP-PTTE reduces duplex stability by ∼1.5–2.0 °C per modification, whereas comparing **Pal-Pip-LNA**_**2**_ with **Pal-LNA**_**2**_ **-PS** indicates that in the presence of 5′-palmitate, the piperidyl triester reduces duplex stability by ∼1.0 °C per modification.

#### Duplex Stability of Pip-PTTE Oligonucleotides at pH 5.5

We also measured the melting temperatures (Tm) of the piperidyl triester oligonucleotides at pH 5.5 (SI 3.1). Pip-LNA_4_ and Pip-LNA_6_ exhibit very high Tm values against complementary RNA at different salt concentrations; therefore, for these constructs, it was not possible to measure the effect of the cationic backbones. For oligonucleotides containing 2, 4, and 6 piperidyl backbones and no LNA sugars, the Tm values against complementary DNA and RNA are not significantly higher at pH 5.5 than at pH 7.0. This is not surprising as the p*K*_a_ of piperidine is around 11; hence, the triesters will be cationic across the full biologically relevant pH range.

In general, Tm values of PTTE triester oligonucleotides against complementary DNA show similar trends to those against RNA (SI 3.0, Tables T3–T14).

#### Conformational Studies

Circular dichroism (CD) was used to evaluate changes in global duplex structure by measurement of polarized light absorption from 200 to 330 nm (Figure 3). LNA-phosphothiotriesters, mixed LNA-phosphothiotriesters, and 2′-OMe, 2′-MOE, and 2′-F phosphothiotriesters have mostly minor structural effects, even in oligonucleotides that contain large numbers of PTTE linkages. The positive band at ∼270 nm and negative band ∼210 were observed in all oligonucleotides, although the intensities and precise wavelength maxima differ (SI 4.0). The wavelength maxima and minima of the PTTE oligonucleotides hybridized to complementary RNA are closely aligned with the control **2′OMePS+**:RNA duplex (Figure 2). The greatest deviation is in the intensity of the negative band around 210 nm, which grows progressively weaker in proportion to the number of PTTE linkages, suggesting reduced helicity. This is most prevalent for **LNA**_**11**_ **-OMe**_**6**_ **-PO**, which has 100% charge-neutral phosphotriester backbones. The positive peak around 270 nm, arising from base stacking, is less affected by the PTTE linkages. Changes to the CD spectra of duplexes with complementary DNA are also the greatest for **LNA**_**11**_ **-OMe**_**6**_ **-PO** (SI 4.0, Figure S108).

**Figure 3:**
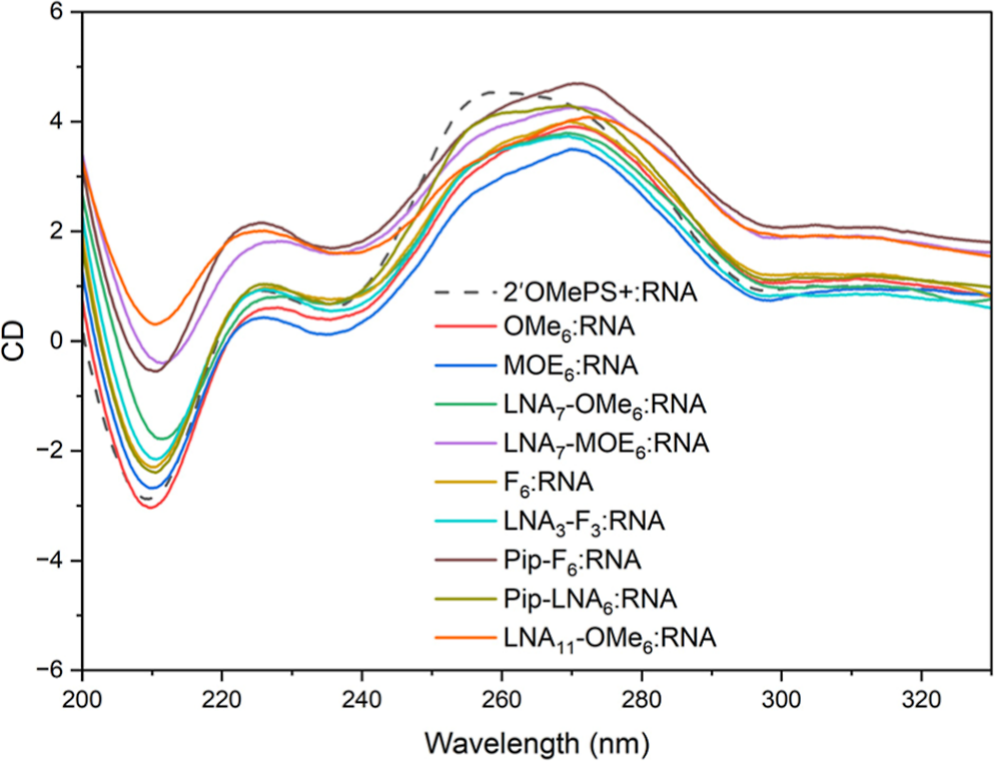
CD spectra of oligonucleotide–RNA duplexes. The *Y*-axis is ellipticity θ (10^–3^ deg·cm^2^/dmol).

#### Biological Activity

Splice-switching oligonucleotides (SSOs) bind to their target pre-mRNA to redirect the spliceosome, enabling control of splicing elements such as enhancers or silencers. This can lead to exon exclusion, intron retention, or the selection of alternative splice sites. These mechanisms are relevant to many genetic diseases. In this study, we evaluated splice switching using the HeLa pLuc/705 cell line,^63^ which contains a luciferase gene disrupted by an intron causing aberrant splicing and lack of expression of luciferase protein. Oligonucleotides that target this defective splice site restore luciferase expression with luminescence proportional to the amount of protein. We evaluated the biological activity of more than 40 oligonucleotides containing 2 to 13 phosphothiotriesters in various combinations under transfection and gymnosis conditions (Figures 4–6). We were interested in comparing lipofectamine-assisted with gymnotic splice switching activity as a function of the nature and number of the PTTE linkages to determine any improvements in splice switching due to enhanced cell uptake or more efficient exon skipping.

**Figure 4:**
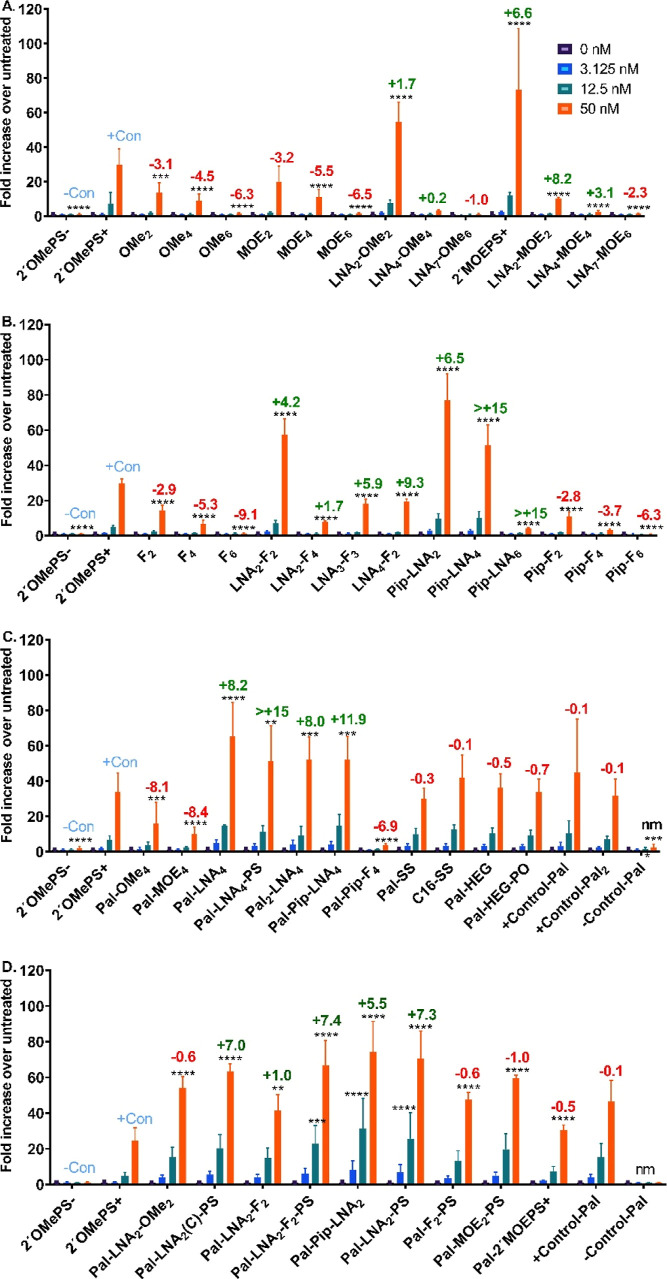
Activity of ONs in HeLa pLuc/705 cells under transfection conditions and relationship to melting temperature. ONs were transfected into HeLa pLuc/705 cells at the indicated concentrations using Lipofectamine 2000, and luciferase activity was measured 48 h later. In all cases, luminescence was normalized to total protein quantity and to untreated cells. Data are means ± standard deviations for three biological replicates (*n* = 3), where each biological replicate was performed in technical triplicate. Statistics are two-way analysis of variance (ANOVA) with Dunnett’s multiple comparisons test against **2′OMePS+**, α = 0.05: **P ≤ 0.0011, ***P ≤ 0.0005, and ****P ≤ 0.0001. Numbers above the bars indicate change in melting temperature relative to **2′MOEPS+** (+con). Red indicates lower Tm, and green indicates higher Tm. Nm = not measured.

**Figure 5:**
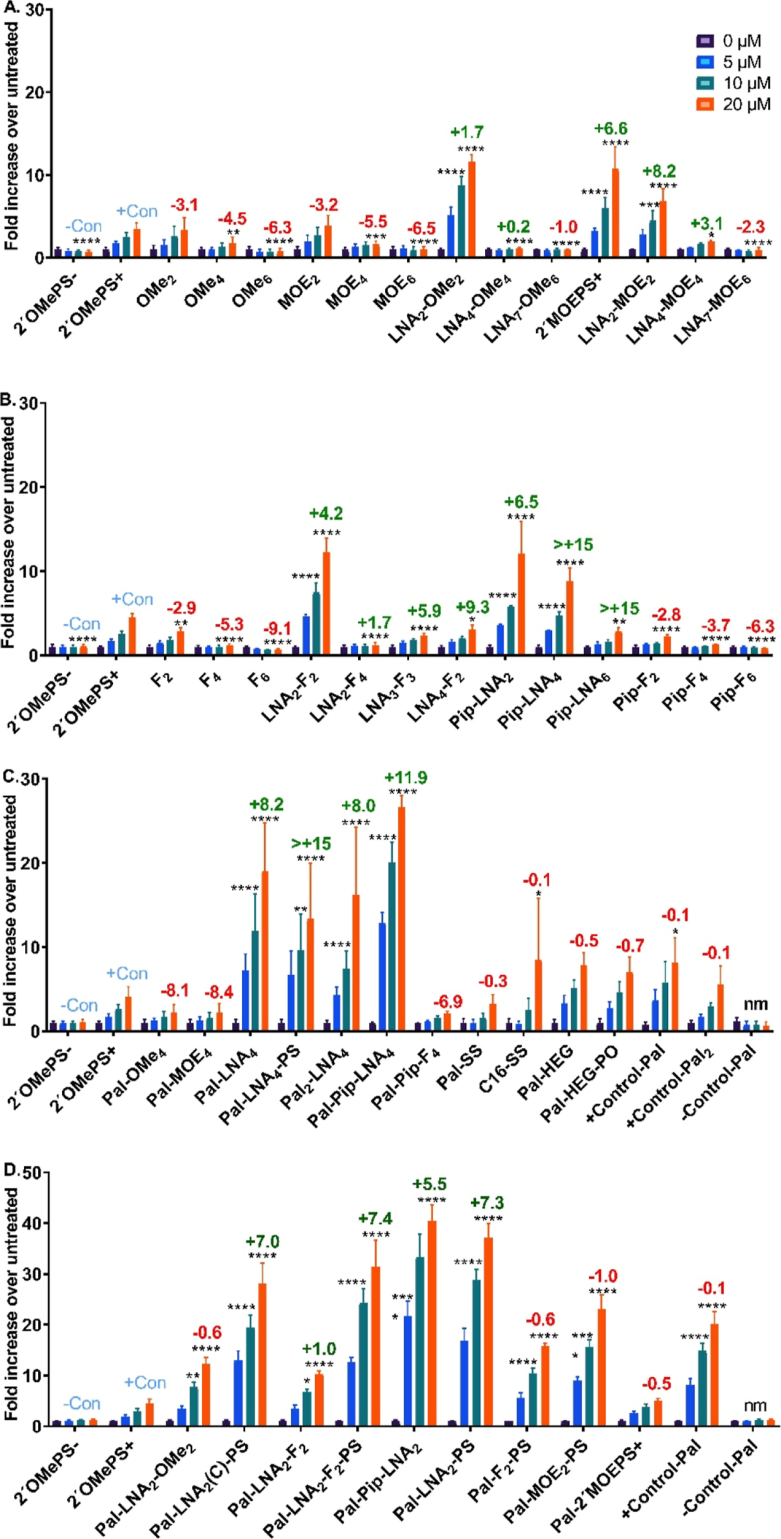
Activities under gymnotic conditions of ONs in HeLa pLuc/705 cells and relationship to melting temperature. ONs were treated to HeLa pLuc/705 cells at the indicated concentrations in the absence of a transfection reagent, and luciferase activity was measured 72 h later. In all cases, luminescence was normalized to total protein quantity and untreated cells. Data are means ± standard deviations for three biological replicates (*n* = 3), where each biological replicate was performed in technical triplicate. Statistics are two-way analysis of variance (ANOVA) with Dunnett’s multiple comparisons test against **2′OMePS+**, α = 0.05: *P ≤ 0.0184, **P ≤ 0.0092, ***P ≤ 0.0010, and ****P ≤ 0.0001. Numbers above the bars indicate change in melting temperature relative to **2′MOEPS+** (+con). Red indicates lower Tm, and green indicates higher Tm.

**Figure 6:**
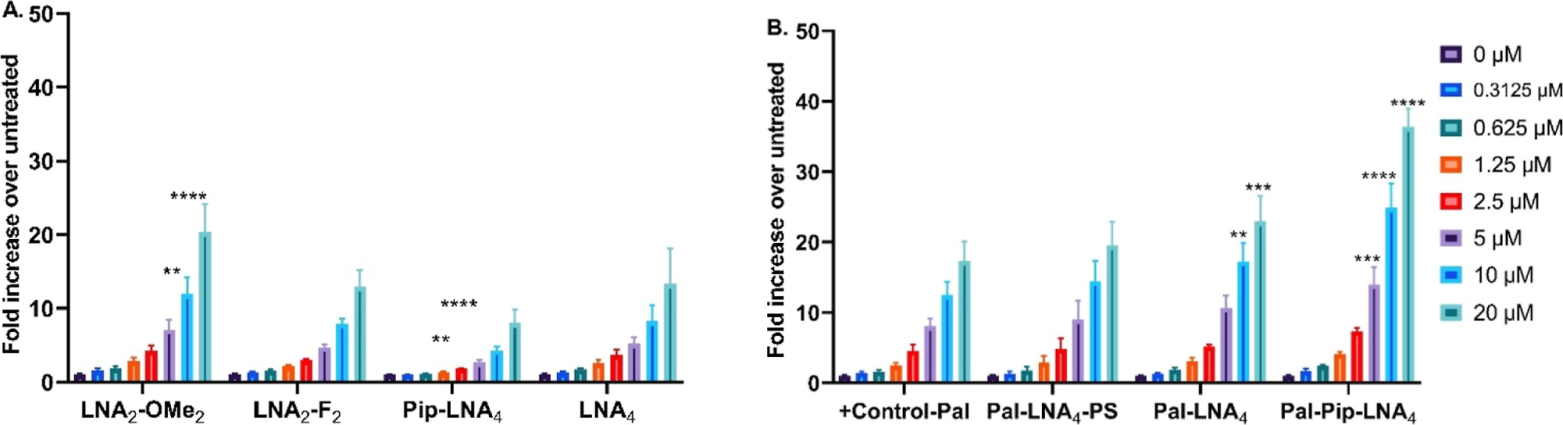
Seven-point gymnotic dose response of selected ONs in HeLa pLuc/705 cells. Oligonucleotides were administered to HeLa pLuc/705 cells at the indicated concentrations without the use of a transfection reagent, and luciferase activity was assessed after 72 h. Luminescence values were normalized to both total protein content and untreated controls. Statistics are two-way analysis of variance (ANOVA) with Dunnett’s multiple comparisons test against **LNA**_**4**_ (A) and **+Control-Pal** (B), α = 0.05: **P < 0.0032, ***P < 0.0005, and ****P < 0.0001. Results are means ± standard deviations from three independent biological replicates (*n* = 3), each performed in technical triplicate.

Under transfection conditions (Figure 4), **LNA**_**2**_ **-OMe**_**2**_, **2′MOEPS+**, **LNA**_**2**_ **-F**_**2**_, **Pip-LNA**_**2**_, and **Pip-LNA**_**4**_ showed improved activity relative to the control **2′OMePS+**. However, **LNA**_**4**_ **-OMe**_**4**_, **LNA**_**7**_ **-OMe**_**6**_, **LNA**_**4**_ **-MOE**_**4**_, and **LNA**_**7**_ **-MOE**_**6**_ were not active. The activity of **LNA**_**2**_ **-MOE**_**2**_ is much lower than expected, only ∼8-fold compared to untreated cells, yet it forms a stable duplex with complementary RNA (ΔTm = +8.2 °C). In contrast, the related **LNA**_**2**_ **-OMe**_**2**_ has greatly enhanced splice-switching activity with a ΔTm of only +1.7 °C. The difference in activity between these two structurally similar oligonucleotides is unexpected. The cumulative data suggest that a general requirement for the activity oligonucleotides containing phosphothiotriester linkages in transfection conditions is at least two LNA PTTE linkages and no more than four PTTE linkages in total. Among lipid conjugates, only **Pal-OMe**_**4**_, **Pal-MOE**_**4**_, and **Pal-F**_**4**_ showed lower activity than the **2′OMePS+**, whereas other palmitate conjugates showed similar or slightly improved activity compared to **2′OMePS+** and **+control-Pal**. As expected, palmitate conjugation does not generally enhance activity during transfection. Highly lipophilic **Pal-2′MOEPS+** showed slightly lower activity than the **+control-Pal**.

Under gymnosis conditions, **LNA**_**2**_ **-OMe**_**2**_, **2′MOEPS+**, and **LNA**_**2**_ **-MOE**_**2**_ showed significantly higher activity than **2′OMePS+** (3.3-, 3.0-, and 1.9-fold, respectively, at 20 μM, Figure 5A). Interestingly, **LNA**_**2**_ **-OMe**_**2**_ has similar activity to **2′MOEPS+**, although the Tm value of the latter is 4.9 °C higher. This shows that charge-neutral PPTE backbones can improve gymnotic activity and suggests that RNA affinity is not the only factor. Oligonucleotides **LNA**_**2**_ **-F**_**2**_ (4 × THP), **Pip-LNA**_**2**_ (2 × Pip), and **Pip-LNA**_**4**_ (4 × Pip) all showed higher activity than **2′OMePS+**, (2.7-, 2.6-, and 1.9-fold at 20 μM, respectively, Figure 5B). As found for transfection, oligonucleotides containing PTTE linkages require at least two LNA PTTE linkages and no more than four PTTE linkages in total to show significant activity during gymnosis.

The activities of **LNA**_**2**_ **-OMe**_**2**_, **LNA**_**2**_ **-F**_**2**_, **Pip-LNA**_**4**_, and **LNA**_**4**_ under gymnosis conditions at seven concentrations on a single plate (Figure 6A) display a clear trend in concentration dependence. Activity follows the order **LNA**_**2**_ **-OMe**_**2**_ > **LNA**_**2**_ **-F**_**2**_-**LNA**_**4**_ > **Pip-LNA**_**4**_, whereas their ΔTm values against complementary RNA follow the order **Pip-LNA**_**4**_ > **LNA**_**4**_-**LNA**_**2**_ **-F**_**2**_ > **LNA**_**2**_ **-OMe**_**2**_. The most active triester analogue **LNA**_**2**_ **-OMe**_**2**_ has a ΔTm value of only 1.5 °C, emphasizing that other factors in addition to target affinity influence gymnotic activity.

The 5′-monopalmitate oligonucleotide conjugates are the most active by gymnosis (Figure 5C, D); for example, **+Control-Pal** is ∼2-fold more active than its nonlipid **2′OMePS+** equivalent. In addition, **Pal-LNA**_**4**_ (4 × THP), **Pal-LNA**_**4**_ **-PS** (0 × THP), **Pal**_**2**_ **-LNA**_**4**_ (4 × THP), and **Pal-Pip-LNA**_**4**_ (4 × Pip) have higher activity than **2′OMePS+** (4.6-, 3.2-, 3.9-, and 6.8-fold at 20 μM). The conjugates with cleavable 5′-palmitate, i.e., **Pal-SS**, **C16-SS**, **Pal-HEG**, and **Pal-HEG-PO**, have similar gymnotic activity to **+Control-Pal**, whereas the most lipophilic oligonucleotide studied (**+Control-Pal**_**2**_ **)** is slightly less active (Figure 5C). Among all lipid conjugates, **Pal-Pip-LNA**_**4**_ has the highest gymnotic activity, reaching almost half the activity achieved by transfection, albeit at the much higher doses that are possible in the absence of a transfection agent.

The relative splice-switching activities of **Pal-LNA**_**2**_ **-OMe**_**2**_ (4 × THP) and **Pal-LNA**_**2**_ **(C)-PS** are 2.7- and 6.3-fold higher, respectively, compared to **2′OMePS+** at 20 μM. **Pal-LNA**_**2**_ **-F**_**2**_ (4 × THP) and **Pal-LNA**_**2**_ **-F**_**2**_ **-PS** are 2.3- and 7.0-fold more active than **2′OMePS+** and correlate with their Tm values. Addition of palmitate to **Pip-LNA**_**2**_ increases the activity considerably (Figure 5D). Among all oligonucleotides tested, **Pip-Pal-LNA**_**2**_ showed the highest splice-switching activity, i.e., 11.3-, 11.0-, and 9.0-fold at 5 μM, 10 μM, and 20 μM, respectively, compared to **2′OMePS+**. It is also slightly more active than the **Pal-LNA**_**2**_ **-PS** control oligonucleotide. **Pal-F**_**2**_ **-PS** and **Pal-MOE**_**2**_ **-PS** have similar activity to **+Control-Pal**, whereas the highly lipophilic **Pal-2′MOEPS+** has very low activity. However, this could be due to the ethylenediamine adducts. The splice switching activities of oligonucleotides compared to **2′OMePS+** at three different concentrations are tabulated in SI 5.3.

Encouraged by the activity of the palmitate conjugates, we compared the activities of **+Control-Pal**, **Pal-LNA**_**4**_ **-PS** (LNA phosphorothioate control), **Pal-LNA**_**4**_ (4 × THP), and **Pal-Pip-LNA**_**4**_ under gymnosis conditions at seven concentrations on a single plate (Figure 6B). Gymnotic activity followed the order **Pal-Pip-LNA**_**4**_ > **Pal-LNA**_**4**_ > **Pal-LNA**_**4**_ **-PS** > **+Control-Pal**, whereas ΔTm values against complementary RNA followed the order **Pal-LNA**_**4**_ **-PS** > **Pal-Pip-LNA**_**4**_ > **Pal-LNA**_**4**_ > **+Control-Pal**. The lower activity of **Pal-LNA**_**4**_ **-PS** could be attributed to off-target effects due to excessively high duplex stability. The high activity of **Pal-Pip-LNA**_**4**_ (2.1-fold at 20 μM relative to **+Control-Pal**) is possibly due to the cationic nature of the piperidyl moiety.

Finally, we noticed that under gymnotic conditions, **Pip-LNA**_**4**_ (4 × Pip) has lower activity than related THP triester oligonucleotides including **LNA**_**4**_ (4 × THP) (Figure 6A). We therefore compared the activity of **Pip-LNA**_**2**_ (2 × Pip) with **LNA**_**2**_ (2 × THP) and the **LNA**_**2**_ **-PS** control (SI 5.2). This confirmed that incorporation of the piperidyl PTTE reduces gymnotic activity, which is opposite to the trend observed for palmitate conjugates, where gymnotic activity follows the order **Pal-Pip-LNA**_**4**_ > **Pal-LNA**_**4**_ > **Pal-LNA**_**4**_ **-PS** and **Pal-Pip-LNA**_**2**_ > **Pal-LNA**_**2**_ **-PS**. These results indicate that the impact of the piperidyl group on gymnotic activity depends on other modifications in the oligonucleotide, suggesting changes in cell uptake/trafficking mechanisms.

We have previously shown that oligonucleotides with a moderately but not excessively high Tm (∼8 °C greater than the control **2′OMEPS+**) exhibit enhanced splice-switching activity.^61^ In this study, we confirm that Tm is an important factor in splice switching; i.e., oligonucleotides with lower duplex stability than **2′OMEPS+** are not significantly active under transfection or gymnosis. Also, oligonucleotides containing mixed LNA PTTE and 2′-OMe or 2′-MOE or 2′-F (**LNA**_**4**_ **-OMe**_**4**_, **LNA**_**7**_ **-OMe**_**6**_, **LNA**_**4**_ **-MOE**_**4**_, and **LNA**_**7**_ **-MOE**_**6**_, **LNA**_**2**_ **-F**_**4**_ **)** do not show significant activity via transfection or gymnosis when more than four PTTE are present even though they form slightly more stable duplexes than the control **2′OMEPS+**.

#### Cell Viability Assays

We evaluated all oligonucleotides for potential cytotoxicity in HeLa pLuc/705 cells following 72 h treatment at a concentration of 20 μM, which is consistent with the gymnotic splice-switching assays. Cell viability was assessed using two complementary assays that measure distinct aspects of cellular health. The ATP-based luminescent assay quantifies intracellular ATP levels, which directly reflect metabolically active and viable cells, while the resazurin assay measures cellular metabolic activity through the enzymatic reduction of resazurin to a fluorescent product, providing an independent indicator of functional cells. Across all oligonucleotides tested, viability ranged between ∼95% and ∼120% relative to untreated controls in both assays (SI 5.1, Figure S141). In addition, representative micrograph images of cells captured after 72 h of treatment showed normal cell morphology and confluency, further supporting the absence of overt toxicity (Figure S140). The strong agreement between these independent readouts supports advancement into disease-relevant animal models.

## Conclusions

We have synthesized oligonucleotides containing different therapeutically relevant sugars combined with THP and (for the first time) piperidyl PTTE linkages. Conveniently, the LNA, 2′-OMe, 2′-MOE, and 2′-F protected nucleosides used as final intermediates to make the modified phosphoramidites are readily available. PTTE monomers could therefore be readily prepared in kilogram scale for large-scale oligonucleotide synthesis. RNA affinity is an important factor for efficient splice switching, and comparing several modified ribose triester derivatives indicates that LNA-PTTE provides the most stable duplexes, whereas 2′-OMe, 2′-MOE, and 2′-F PTTE backbones are less effective. We also show that duplex stability can be fine-tuned by judiciously combining LNA-PTTE with the other PTTE backbones. Cell studies show a reduction in exon-skipping activity for oligonucleotides containing two to six 2′-OMe, 2′-MOE, or 2′-F PTTE modifications associated with lowering of duplex stability. In contrast, chimeric PTTE oligonucleotides with higher LNA to 2′-OMe, 2′-MOE, or 2′-F ratios were more active than the 2′-O-methyl phosphorothioate control. Introducing piperidyl into the PTTE backbone improves duplex stability and, in combination with palmitate, greatly enhances gymnotic exon-skipping activity. It will therefore be interesting to carry out animal studies where toxicity, biodistribution, and tissue selectivity can be determined. With appropriate controls, this should make it possible to determine if the toxic effects of LNA originating from its high affinity for partly complementary RNA (off-target effects)^64–67^ and undesired protein binding^68,69^ are eliminated when the LNA sugar is attached to triesters.

As of now, all clinically approved oligonucleotides that contain phosphorothioate backbones are diastereomeric at the phosphorus. However, stereopure phosphorothioate oligonucleotides have interesting properties and can be synthesized by P­(V)^70^ or P­(III)^71^ chemistry. Synthetic efforts to produce stereopure phosphotriester or phosphothioester oligonucleotides might also lead to improved properties. Such studies will be simplified by the fact that THP and piperidyl are achiral.

## Supplementary Material



## Notes

Experimental procedures, analytical data for novel compounds including 1D (^1^H, ^13^C, and ^31^P) NMR spectra and HRMS, DMT + analysis for oligonucleotide assembly, UPLC-ESI-MS of oligonucleotides, UV melting data, and CD spectra (PDF)
