# Supplementary material for: 5′-Palmitate Lipid and Internal LNA-Piperidyl Triesters Enhance the RNA Affinity and Activity of Splice-Switching Oligonucleotides
Source: J Am Chem Soc. 2026 Jun 9;148(24):25198–209. doi: 10.1021/jacs.6c07448 (PMC13307365; doi:10.1021/jacs.6c07448)
Supplement: Supplementary file 1 [file ja6c07448_si_001.pdf]

5´-Palmitate Lipid and Internal LNA-Piperidyl Triesters Enhance the RNA Affinity and Activity of Splice-Switching Oligonucleotides

Debashis Dhara[1] and Tom Brown[1,*]

[1]Department of Chemistry, University of Oxford, Chemistry Research Laboratory, 12 Mansfield Road, Oxford OX1 3TA, United Kingdom

Table of Contents

1.0 Chemical Synthesis 3

1.1 General Procedure 3

1.2 Experimental Procedures 4

2.0 Oligonucleotide Synthesis and Analysis 11

2.1 Oligonucleotide Synthesis, Deprotection and Purification 11

2.3 Calculated and Observed Masses of Oligonucleotides 13

2.4 Yields of Oligonucleotide Synthesis (1.0µM Scale) 15

2.5 UPLC-ESI-MS of Oligonucleotides 17

2.5.1 2´-OMe oligonucleotides 17

2.5.2 Mixed 2´-OMe and 2´-MOE chimeric oligonucleotides 20

2.5.3 Mixed 2´-OMe, 2´-MOE, and LNA chimeric oligonucleotides 23

2.5.4 2´-F oligonucleotides 31

2.5.4 Piperid-4-yl PTTE oligonucleotides 38

2.5.4 Lipid conjugated oligonucleotides 44

3.0 Thermal Stability Studies 67

3.1 Thermal Stability Studies in Acidic pH 82

4.0 Circular Dichroism Studies 83

5.0 Biological Experiments 91

5.1 Cell Viability Assay 93

5.2 Gymnosis Data of Pip-LNA_2, LNA_2 and LNA_2-PS 96

5.3 Relative Splice-Switching Activity (SSA) 97

6.0 NMR Spectra of Compounds 98

7.0 Selected Trityl Readings of Solid Phase Synthesis 114

8.0 Detailed Chemical Structures of Oligonucleotides 116

9.0 References 119

1.0 Chemical Synthesis

1.1 General Procedure

Solvents: acetonitrile (MeCN), dichloromethane (DCM), chloroform ($CHCl_3$), ethyl acetate (EtOAc), ether (Et_2O), pentane, hexane, cyclohexane, triethylamine (Et_3N) were collected from an mBraun SPS-800 bench top solvent purification system, having passed through anhydrous alumina columns. Solvents for nucleoside phosphitylation reactions were degassed by bubbling argon through before use. Solvents for purification such as hexane (Hex) and ethyl acetate (EtOAc) were degassed by bubbling argon through and were mixed with 1% dry triethylamine. Reactions requiring anhydrous conditions were performed under an argon atmosphere, using oven-dried glassware which was allowed to attain room temperature whilst flowing dry argon through. Thin layer chromatography (TLC) was performed using Merck pre-coated 0.23 mm thick plates (Kieselgel 60 F254) and visualised using UV light (λ = 254 nm). Flash column chromatography was carried out using silica gel (60 μm particle size).

NMR spectroscopy: Each compound was analysed by ^{1}H, ^{13}C and ^{31}P NMR spectra which were recorded on a Bruker AVIIIHD 400 MHz spectrometer using an internal deuterium lock at ambient probe temperatures. The service was provided by the Department of Chemistry NMR Spectroscopy Service, University of Oxford. NMR chemical shifts (δ) are quoted to the nearest 0.01 ppm and are referenced relative to residual solvent peaks. Spectra were recorded in $CDCl_3$. Chemical shifts are reported in ppm (δ) relative to residual solvent peaks, $CHCl_3$ in the case of $CDCl_3$ at 7.28 or 77.0 ppm for the ^{1}H and ^{13}C spectra, respectively. Coupling constants are reported in Hertz (Hz).

Mass spectrometry: High-resolution mass spectra (HRMS) were recorded by the Chemistry Department Mass Spectroscopy Service, University of Oxford on a Thermo Scientific Exactive Mass Spectrometer (Waters Equity autosampler and pump) by electrospray ionization (ESI) and an Agilent 7200 Accurate Mass QTOF GCMS

Analysis of Oligonucleotides: All oligonucleotides were characterised by negative-mode ultra-performance liquid chromatography (UPLC) mass spectrometry using a Waters Xevo G2-XS QTOF mass spectrometer with an Acquity UPLC system. The system was equipped with an Acquity oligonucleotide BEH C18 UPLC column (particle size: 1.7 μm; pore size: 130 Å; column dimensions: 2.1 mm x 50 mm). Data were analysed using Waters MassLynx software v 4.1 or Waters UNIFI Scientific Information System software.

MALDI-TOF-MS analysis: Oligonucleotides **LNA_{11}-OMe_6-PO** was analysed by MALDI-TOF-MS analysis. For MALDI-TOF-MS analysis, HCCA (α-cyano-4-hydroxycinnamic acid) was saturated and dissolved in 1 mL of a solvent mixture consisting of 70% water, 30% acetonitrile, and 0.1% trifluoroacetic acid (TFA). The resulting suspension was vortex-mixed for 1 minute, sonicated for 15 minutes, and vortex-mixed again for one minute. For sample application, 0.5 μL of the matrix solution was applied to an 800 μm AnchorChip target and allowed to dry, after which 0.5 μL of oligonucleotide solution was layered on top of the dried matrix spot and left to dry at room temperature.

1.2 Experimental Procedures

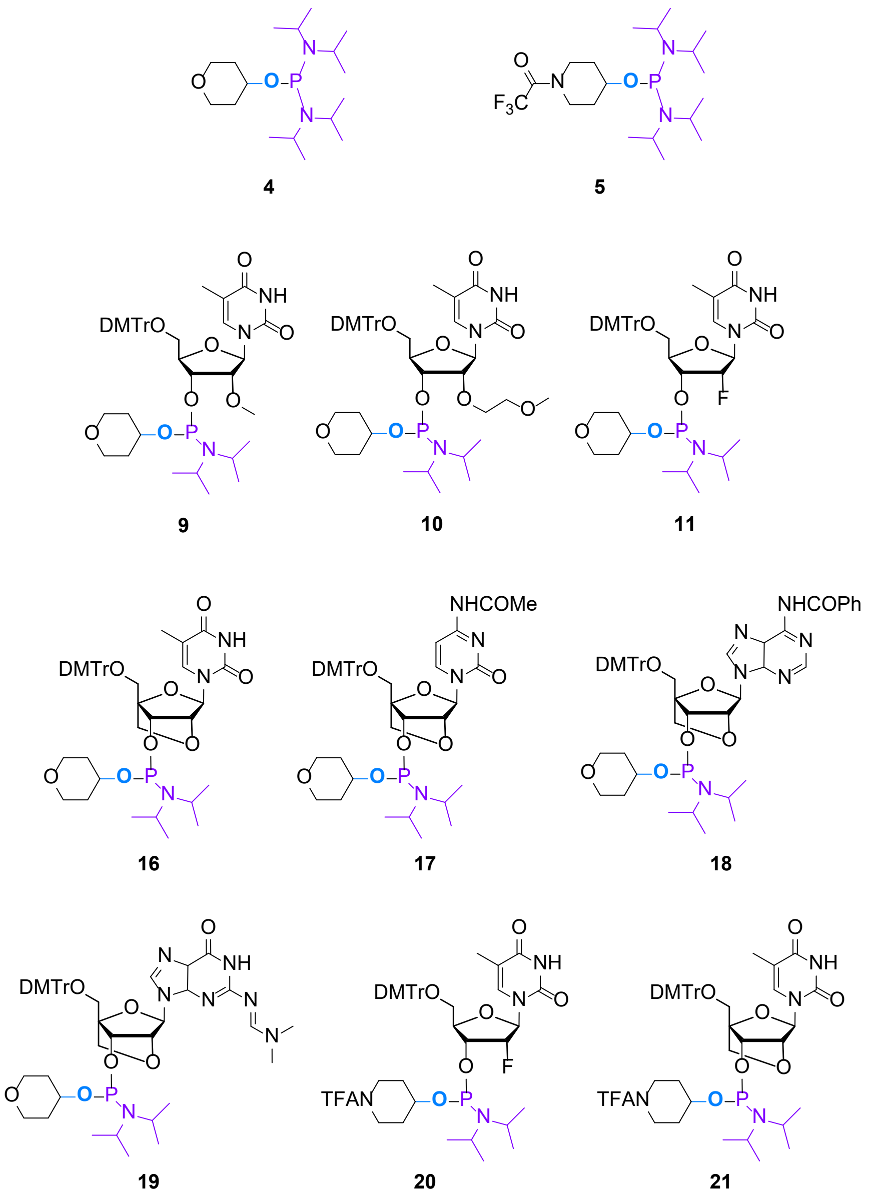

Figure S1: Chemical structure of compounds synthesised in this study.

N,N,N',N'-tetraisopropyl-1-((tetrahydro-2H-pyran-4-yl)oxy)phosphanediamine (4): To a suspension of bis(diisopropyl)chlorophosphine **2** (2.5 g, 9.37 mmol, 1.0 equiv.) in anhydrous Et_2O (30.0 mL) was added anhydrous triethylamine (3.92 mL, 28.1 mmol, 3.0 equiv.). Then tetrahydro-4-pyranol (**1**, 1.34 mL, 5.62 mmol, 1.5 equiv.) was added slowly by a syringe. The reaction was left for 2h under argon. The triethylamine salt was then removed by cannula filtration and the solids were washed with dry Et_2O (2 x 10 mL). The combined filtrate was concentrated on a rotary evaporator. The crude product was dried over high vacuum for 30 minutes then 30 mL of MeCN and 30 mL of pentane were added. The biphasic mixture was transferred to a separatory funnel and the acetonitrile part was discarded. The Pentane fraction was collected and then concentrated on a rotary evaporator. The crude product was finally dried

over high vacuum pump to give the desired product **3** as a white solid (2.8 g, 90%). ^{1}H NMR (400 MHz, $CDCl_3$) δ 3.85 (ddd, J = 10.9, 6.8, 3.7 Hz, 3H), 3.79-3.74 (m, 1H), 3.57 – 3.37 (m, 6H), 1.85-1.78 (m, 2H), 1.67 – 1.54 (m, 2H), 1.10 (dd, J = 6.7, 1.3 Hz, 24H). ^{13}C NMR (101 MHz, CDCl3) δ 67.89, 67.70, 65.36, 44.63, 44.50, 34.26, 34.22, 24.54, 24.45, 24.24, 24.18. **^{31}P NMR** See Reference 1**.** **HRMS** (ESI^{+}): m/z [M+H]$^{+}$ calc. $C_{17}H_{38}N_2O_2P$ 333.2665; found 333.2707.

1-(4-((bis(diisopropylamino)phosphaneyl)oxy)piperidin-1-yl)-2,2,2-trifluoroethan-1-one (**5**): To a suspension of bis(diisopropyl)chlorophosphine **3** (1.5 g, 5.62 mmol, 1.0 equiv.) dissolved in anhydrous Et_2O (25.0 mL) was added anhydrous Triethylamine (2.35 mL, 16.87 mmol, 3.0 equiv.). Then 2,2,2-trifluoro-1-(4-hydroxypiperidin-1-yl)ethan-1-one (**2**, 1.34 mL, 5.62 mmol, 1.5 equiv.) was added slowly by syringe. The reaction was left to for 1h under argon. The solution was then filtered from the triethylamine salt by cannula. Solids were washed with dry Et_2O (2 x 15 mL), the combined filtrate was concentrated on a rotary evaporator and the crude product was dried over high vacuum for 30 minutes. To the crude material 20 mL of MeCN and 20 mL of pentane were added. The biphasic mixture was transferred to a separatory funnel and the acetonitrile part was discarded. The Pentane part was collected and then concentrated on a rotary evaporator. The crude product was finally dried over a high vaccum pump to give the desired product **3** as a white solid (1.6 g, 3.74, 67%). The amidite **5** had, **^{1}H NMR** (400 MHz, $CDCl_3$) δ 3.99 – 3.87 (m, 2H), 3.71-3.63 (m, 1.5H), 3.62-3.49 (m, 4.2H), 1.86-1.76 (m, 4.6H), 1.17 (dd, J = 6.8, 4.9 Hz, 28H). **^{31}P NMR** (162 MHz, $CDCl_3$) δ 111.31. **^{19}F NMR** (376 MHz, $CDCl_3$) δ -68.92. **HRMS** (ESI^{+}): m/z [M+H]$^{+}$ calc. $C_{19}H_{38}F_3N_3O_2P$ 428.2648; found 428.2661.

5′-*O*-(4,4′-dimethoxytrityl)-2′-*O*-methyl thymidine-3′-*O*-[(*O*-(tetrahydro-2H-pyran-4-yl)-*N,N′*-diisopropylphosphoramidite] (9): 2´-O-Methyl thymidine monomer **6** (600 mg, 1.04 mmol, 1.0 equiv.) was dissolved in dry, degassed DCM (20 mL). Phosphitylating reagent **4** (694 mg, 1.09 mmol, 2.0 equiv.) was added followed by the addition of tetrazole (0.45 M in acetonitrile, 2.31 mL, 1.04 mmol, 1.0 equiv.). The reaction mixture was left to stir at rt for 16h under an atmosphere of argon. At completion, following a TLC analysis, the reaction was diluted with 20 mL of DCM. The diluted reaction mixture was transferred to a separatory funnel and washed with saturated NaCl solution (20 mL). The DCM part was collected and dried over anhydrous $MgSO_4$, filtered, and concentrated on a rotary evaporator. The crude residue was purified by flash chromatography (40:60 Hex:EtOAc, both solvent were mixed with 1% triethylamine) to give the desired DMT-OMe-T-THP phosphoramidite **9** as a white solid (700 mg, 869 µmol, 83%). The amidite **9** had, **^{1}H NMR** (400 MHz, $CDCl_3$) δ 8.17 (s, 1H), 7.70 (d, J = 1.8 Hz, 0.5H), 7.63 (s, 0.5H), 7.57 (s, 0.5H), 7.34 (t, J = 8.2 Hz, 2H), 7.29 – 7.10 (m, 9H), 6.77 (ddt, J = 9.2, 4.5, 2.2 Hz, 4H), 6.02 (dt, J = 8.4, 2.8 Hz, 1H), 4.51 (dd, J = 8.7, 4.4 Hz, 1H), 4.41 (ddt, J = 14.7, 9.7, 4.6 Hz, 1H), 4.26 – 4.20 (m, 1H), 4.16 (d, J = 3.9 Hz, 0.5H), 4.06 – 3.96 (m, 1H), 3.96 – 3.92 (m, 0H), 3.86 (ddt, J = 17.1, 11.9, 5.8 Hz, 3H), 3.77 (d, J = 6.3 Hz, 0.3H), 3.73 (d, J = 1.9 Hz, 6H), 3.62 – 3.50 (m, 3H), 3.47 (dd, J = 6.8, 1.8 Hz, 5H), 3.46 – 3.31 (m, 1H), 1.96 – 1.88 (m, 2H), 1.88 – 1.67 (m, 1H), 1.67 – 1.51 (m, 5H), 1.26 (d, J = 9.5 Hz, 4H), 1.20 (dd, J = 6.9, 2.0 Hz, 12H), 1.13 (s, 1H), 0.96 (dd, J = 6.8, 1.7 Hz, 3H). **^{13}C NMR** (101 MHz, $CDCl_3$) δ 163.40, 158.70, 150.11, 144.11, 135.39, 135.12, 130.21, 130.12, 130.11,

128.29, 128.19, 127.89, 127.88, 127.16, 113.18, 113.15, 113.12, 110.99, 87.01, 86.82, 83.24, 77.26, 76.94, 76.62, 70.39, 69.67, 65.23, 64.96, 64.86, 62.08, 58.53, 58.08, 55.17, 45.13, 45.07, 43.16, 43.04, 42.87, 34.17, 33.87, 33.66, 24.66, 24.58, 24.39, 24.32, 22.97, 22.95, 22.78, 22.75, 11.48. **^{31}P NMR** (162 MHz, $CDCl_3$) δ 148.15, 147.05. **HRMS** (ESI$^+$): m/z [M+H]$^+$ calc. $C_{43}H_{57}N_3O_{10}P$ 806.3776; found 806.3781.

5′-*O*-(4,4′-dimethoxytrityl)-2′-*O*-methoxyethyl thymidine-3′-*O*-[(*O*-(tetrahydro-2H-pyran-4-yl)-*N,N*′-diisopropylphosphoramidite] (10): MOE-DMT-T nucleoside **7** (500 mg, 808 µmol, 1.0 equiv.) was dissolved in dry, degassed DCM (20 mL). Phosphitylating reagent **4** (537 mg, 1.62 mmol, 2.0 equiv.) was added followed by the addition of tetrazole (0.45 M in acetonitrile, 1.79 mL, 808 µmol, 1.0 equiv.). The reaction mixture was left to stir at rt for 16 h under an atmosphere of argon. At completion, the reaction was diluted with 20 mL of DCM. The diluted reaction mixture was transferred to a separatory funnel and washed with saturated NaCl solution (50 mL). The DCM part was collected and dried over anhydrous $MgSO_4$, filtered, and concentrated on a rotary evaporator. The crude residue was purified by flash chromatography (50:50, Hex:EtOAc with 1% triethylamine) to give the desired DMT-MOE-T-THP phosphoramidite **10** as a white solid (450 mg, 529µmol, 65%). **^{1}H NMR** (400 MHz, $CDCl_3$) δ 8.46 (s, 1H), 7.65 (s, 0H), 7.60 (s, 0.5H), 7.38 (t, J = 6.9 Hz, 2.3H), 7.32 – 7.16 (m, 4.8H), 6.80 (dq, J = 9.5, 2.6 Hz, 4.2H), 6.17 (d, J = 1.9 Hz, 0.3H), 6.04 (q, J = 3.4 Hz, 1H), 4.54 (d, J = 9.9 Hz, 1H), 4.41 (h, J = 5.3 Hz, 1.2H), 4.21 (dt, J = 9.5, 4.7 Hz, 1.6H), 4.06 (s, 1H), 3.93 – 3.75 (m, 5.7H), 3.75 (s, 6.6H), 3.62 – 3.42 (m, 9.4H), 3.44 – 3.24 (m, 2.8H), 3.30 (s, 2.3H), 1.99 – 1.91 (m, 1.3H), 1.77 (ddd, J = 22.5, 11.2, 3.8 Hz, 1.2H), 1.70 – 1.59 (m, 1.5H), 1.30 (d, J = 4.9 Hz, 4.1H), 1.23 (d, J = 6.7 Hz, 9.9H), 1.13 (dd, J = 14.0, 6.7 Hz, 8.8H). **^{13}C NMR** (101 MHz, $CDCl_3$) δ 163.85, 163.82, 158.89, 158.87, 150.47, 144.38, 135.93, 135.50, 135.44, 135.38, 135.34, 130.39, 130.35, 130.31, 128.46, 128.39, 128.10, 127.29, 113.39, 113.36, 113.33, 111.00, 87.27, 87.15, 83.64, 83.32, 82.34, 81.58, 77.48, 72.44, 71.03, 70.38, 69.94, 69.88, 68.75, 65.55, 65.48, 65.40, 65.30, 65.17, 65.06, 62.92, 62.57, 59.26, 55.36, 45.34, 45.28, 43.32, 43.20, 43.14, 43.02, 34.42, 34.08, 34.04, 33.90, 33.86, 24.91, 24.82, 24.78, 24.71, 24.61, 24.58, 24.53, 23.18, 23.16, 22.99, 22.96, 14.19, 11.72. **^{31}P NMR** (162 MHz, $CDCl_3$) δ 147.64, 146.89. **HRMS** (ESI$^+$): m/z [M+H]$^+$ calc. $C_{45}H_{61}N_3O_{11}P$ 850.4038; found 850.4060 and m/z [M+Na]$^+$ calc. $C_{45}H_{60}N_3O_{11}PNa$ 872.3858; found 872.3868.

5′-*O*-(4,4′-dimethoxytrityl)-2′-deoxy-2′-fluoro thymidine-3′-*O*-[(*O*-(tetrahydro-2H-pyran-4-yl)-*N,N*′-diisopropylphosphoramidite] (11): 2′-F-DMT-T nucleoside **8** (900 mg, 1.60 mmol, 1.0 equiv.) was dissolved in dry, degassed DCM (30 mL). Phosphitylating reagent **4** (1.06 g, 3.20 mmol, 2.0 equiv.) was added followed by the addition of tetrazole (0.45 M in acetonitrile, 3.55 mL, 1.60 mmol, 1.0 equiv.). The reaction mixture was purged with argon and then left to stir at rt for 24 h under an atmosphere of argon. Following a TLC analysis, the reaction was diluted with 30 mL of DCM. The diluted reaction mixture was transferred to a separatory funnel and washed with saturated NaCl solution (50 mL). The DCM part was collected and the aqueous layer was washed with DCM (20 mL). The DCM parts were combined and was passed though dry $MgSO_4$. The residue was purified by flash chromatography (60:40 Hex:EtOAc, mixed with 1% triethylamine) to give the phosphoramidite **11** as a white solid (940 mg, 1.18 mmol, 74%). The **11** had **^{1}H NMR** (400

MHz, $CDCl_3$) δ 8.70 (s, 1.8H), 7.37 (t, J = 7.4 Hz, 3.9H), 7.30 – 7.16 (m, 14.4H), 6.79 (ddd, J = 9.1, 6.0, 3.1 Hz, 7.9H), 6.07 (dd, J = 15.9, 2.5 Hz, 1.8H), 5.12 (dt, $^2J_{H-F}$ = 28.0 Hz, J = 3.8 Hz, 0.8H), 5.07 – 4.88 (m, 1H), 4.64 – 4.44 (m, 1.75H), 4.21 (dd, J = 11.9, 7.1 Hz, 2.1H), 3.88 – 3.82 (m, 2H), 3.77 – 3.75 (m, 14H), 3.67 – 3.27 (m, 12H), 1.88 – 1.80 (td, J = 14.2, 7.2 Hz, 1.5H), 1.64 – 1.60 (m, 3.4H), 1.30 (s, 4H), 1.28 – 1.08 (m, 38H). **^{13}C NMR** (101 MHz, $CDCl_3$) δ 163.75, 163.71, 158.94, 158.91, 158.89, 150.13, 144.32, 144.22, 135.56, 135.39, 135.30, 135.25, 130.42, 130.40, 130.34, 130.32, 128.50, 128.37, 128.10, 127.38, 127.32, 113.39, 113.37, 113.33, 111.49, 93.69, 93.10 ($^1J_{C-F}$, 236 Hz), 91.76, 91.18 ($^1J_{C-F}$, 232 Hz), 88.25, 87.91, 87.11, 87.09, 82.25, 69.76, 69.60, 68.85, 68.67, 65.40, 65.36, 65.23, 65.20, 61.91, 61.40, 60.52, 55.36, 44.65, 44.59, 43.43, 43.31, 43.19, 34.28, 34.25, 34.18, 24.83, 24.75, 24.67, 24.60, 24.55, 24.47, 23.81, 23.78, 22.77, 22.75, 22.46, 11.78, 11.70. **^{31}P NMR** (162 MHz, $CDCl_3$) δ 148.41, 148.36, 148.12, 148.04. **^{19}F NMR** (377 MHz, $CDCl_3$) δ -201.91 (d, J = 9.1 Hz), -202.42 (d, J = 13.1 Hz). **HRMS** (ESI^+): m/z $[M+H]^+$ calc. $C_{42}H_{54}FN_3O_9P$ 794.3576; found 794.3563 and m/z $[M+Na]^+$ calc. $C_{42}H_{53}FN_3O_9PNa$ 816.3396; found 816.3379.

5′-*O*-(4,4′-dimethoxytrityl)-2′-*O*,4′-*C*-methylenethymidine-3′-*O*-[(*O*-(tetrahydro-2H-pyran-4-yl)-*N,N*′-diisopropylphosphoramidite] (16): Locked nucleic acid nucleoside **12** (600 mg, 1.05 mmol, 1.0 equiv.) was dissolved in dry, degassed DCM (10 mL). Phosphitylating reagent **3** (697 mg, 2.10 mmol, 2.0 equiv.) was added followed by the addition of tetrazole (0.45 M in acetonitrile, 2.62 mL, 1.05 mmol, 1.0 equiv.). The reaction mixture was left to stir at rt for 16h under an atmosphere of argon. At completion, the reaction was diluted with dry, degassed DCM (10 mL) and washed with saturated KCl solution (10 mL). The DCM part was passed though $MgSO_4$ and then concentrated in a rotary evaporator. The crude product was purified by flash chromatography (50:50 Hex:EtOAc with 1% triethylamine) to give the DMT-LNA-T-THP phosphoramidite **16** (750 mg, 933 µmol, 89%) as a white solid as a mixture of two diastereomers. The phosphoramidite **16** had R_f 0.31 (1:1, Hex:EtOAc, mix with 1% Et_3N). **^{31}P NMR** (162 MHz, $CDCl_3$) δ 147.19, 146.60. **HRMS** (ESI^+): m/z $[M+H]^+$ calc. $C_{43}H_{55}N_3O_{10}P$ 804.3620; found 804.3641. The analytical data were matched with the previous deported data.(1)

N4-Acetyl 5′-*O*-(4,4′-dimethoxytrityl)-2′-*O*,4′-*C*-methylencytidine-3′-*O*-[(*O*-(tetrahydro-2H-pyran-4-yl)-*N,N*′-diisopropylphosphoramidite] (17): *Protocol 1:* Locked nucleoside **13** (300 mg, 500 µmol, 1.0 equiv.) was dissolved in anhydrous chloroform (12 mL). Phosphitylating reagent 3 (333 mg, 1.0 mmol, 2.0 equiv.) was added followed by the addition of tetrazole (0.45 M in acetonitrile, 1.11 mL, 500 µmol, 1.0 equiv.). The reaction was left to stir at rt for 24h under an atmosphere of argon. At completion, the reaction was diluted with chloroform (10 mL), transferred to a separatory funnel and washed with saturated NaCl solution (15 mL). The chloroform part was collected and dried over MgSO4 and then concentrated on a rotary evaporator. The crude product was purified by flash chromatography (100% EtOAc, mixed with 1% triethylamine and 1% MeOH) to give the DMT-LNA-C-THP phosphoramidite **17** (110 mg, 132 µmol, 26%) as a white solid. **^{1}H NMR** (400 MHz, $CDCl_3$) δ 8.80 (s, 1H), 8.39 (dd, J = 7.5, 2.2 Hz, 1H), 7.45 (tt, J = 6.3, 1.4 Hz, 2.1H), 7.39 – 7.23 (m, 9.4H), 6.86 (ddd, J = 8.9, 3.6, 2.4 Hz, 4.7H), 5.78 (d, J = 2.1 Hz, 1H), 4.71 (d, J = 12.5 Hz, 1H), 4.24 (d, J = 9.1 Hz, 0.7H), 4.14 (d, J = 7.0 Hz, 0.73), 3.99 – 3.88 (m, 0.5H), 3.91 – 3.67

(m, 11.3H), 3.56 (d, J = 10.4 Hz, 0.9H), 3.56 – 3.39 (m, 4H), 3.43 – 3.28 (m, 1H), 2.25 (s, 3H), 1.74 – 1.68 (m, 0.7H), 1.57 – 1.47 (m, 0.3H), 1.48 – 1.38 (m, 1H), 1.11 (dd, J = 8.2, 6.8 Hz, 7H), 1.03 (d, J = 6.8 Hz, 4H), 0.97 (d, J = 6.7 Hz, 2H). **^{13}C NMR** (101 MHz, $CDCl_3$) δ 162.97, 158.77, 149.85, 144.22, 135.26, 135.15, 130.23, 130.16, 130.08, 128.23, 128.10, 127.99, 127.23, 113.26, 113.23, 88.35, 88.08, 86.76, 77.35, 77.04, 76.72, 72.26, 65.05, 64.94, 57.97, 55.25, 46.88, 45.22, 45.15, 43.25, 43.19, 34.13, 33.96, 33.78, 25.09, 24.56, 24.48, 23.05, 23.03, 22.86, 22.83, 22.34, 19.16, 14.06. **^{31}P NMR** (162 MHz, $CDCl_3$) δ 147.17, 146.43. **HRMS** (ESI^+): m/z $[M+H]^+$ calc. $C_{44}H_{56}N_4O_{10}P$ 831.3729; found 831.3748 and m/z $[M+Na]^+$ calc. $C_{44}H_{55}N_4O_{10}PNa$ 853.3548; found 853.3541.

Protocol 2: Locked nucleic acid intermediate **13** (400 mg, 667 µmol, 1.0 equiv.) was dissolved in anhydrous chloroform (15 mL). Phosphitylating reagent **4** (444 mg, 1.33 mmol, 2.0 equiv.) was added followed by tetrazole (0.45 M in acetonitrile, 1.48 mL, 667 µmol, 1.0 equiv.). The reaction was left to stir at rt for 24h under an atmosphere of argon. At completion, the reaction was diluted with chloroform (30 mL), transferred to a separatory funnel and washed with saturated NaCl solution (2 0mL). The chloroform part was collected and dried over $MgSO_4$ and then concentrated on a rotary evaporator. The crude product was washed with pentane (2 x 20 mL) and then dried under high vaccum for 3 h to give the crude phosphoramidite **17** as a white solid (crude weight, 520 mg, 626 µmol, 93%). The analytical data matched with above. The crude product was directly used in the solid phase synthesis.

N6-Benzoyl-5′-*O*-dimethoxytrityl-2′-*O*,4′-*C*-methyleneadenine-3′-*O*-[(*O*-(tetrahydro-2H-pyran-4-yl)-*N,N′*-diisopropylphosphoramidite (18): Locked nucleic acid intermediate **14** (1.0 g, 1.45 mmol, 1.0 equiv.) was dissolved in dry, degassed DCM (20 mL). The solution was purged with argon for 10-15 minutes. Phosphitylating reagent **4** (969 mg, 2.91 mmol, 2.0 eqv.) was added followed by tetrazole (0.45M in MeCN, 3.24 mL, 1.0 equiv.) and the reaction mixture was left to stir at rt for 16h under argon. At completion, the reaction mixture was diluted with dry DCM (30 mL) and washed with saturated KCl solution (10 mL). The DCM part was collected and the aqueous layer was washed with DCM (30 mL). The DCM parts were combined and dried over $MgSO_4$. The work up was performed under argon. The DCM part was concentrated and the crude residue was purified by flash chromatography (90:10 Hex:EtOAc, mixed with 1% Et_3N) to give the desired phosphoramidite product **18** as a white solid (1.1 g, 1.2 mmol 82%). The analytical data matched with the previous deported data.(1)

N2-Dimethylformamidine-5′-*O*-dimethoxytrityl-2′-*O*,4′-*C*-methylenguanosine-3′-*O*-[(*O*-(tetrahydro-2H-pyran-4-yl)-*N,N′*-diisopropylphosphoramidite (19): The locked nucleoside intermediate **15** (300 mg, 458 µmol, 1.0 equiv.) was dissolved in mixture of $CHCl_3$:MeCN:DMF (1:1:1). Phosphitylating reagent **4** (305 mg, 916 µmol, 2.0 eqv.) was added followed by tetrazole (0.45M in MeCN, 1.01 mL, 458 µmol, 1.0 equiv.). The reaction mixture was left to stir at rt for 16h under argon. After TLC analysis, the reaction mixture was concentrated, diluted with $CHCl_3$ (20 mL) and washed with saturated NaCl solution (20 mL). The $CHCl_3$ layer was collected, dried over Na_2SO4 and then concentrated. The crude product was purified by flash chromatography (40:60 Hex:EtOAc, EtOAc with 10% methanol and 1% Et_3N). The fraction corresponding to the desired product was concentrated to give the product **19** as a white solid (280 mg, 316 µmol, 69%). **^{1}H NMR** (400 MHz, $CDCl_3$) δ 8.92 (s, 1H), 8.61

(d, J = 3.8 Hz, 1H), 7.87 (d, J = 3.7 Hz, 1H), 7.51 – 7.41 (m, 2.2H), 7.40 – 7.07 (m, 9H), 6.88 – 6.80 (m, 4.4H), 5.98 (d, J = 5.0 Hz, 1H), 4.70 (d, J = 17.7 Hz, 1H), 4.33 (d, J = 9.6 Hz, 0.6H), 4.22 (d, J = 7.2 Hz, 0.3H), 4.09 (t, J = 7.3 Hz, 1H), 3.98 (dd, J = 7.8, 3.8 Hz, 1H), 3.82 – 3.70 (m, 8.5H), 3.49 (d, J = 21.1 Hz, 2H), 3.44 (s, 0.2H), 3.20 (d, J = 2.5 Hz, 3.2H), 3.11 (s, 3H), 2.35 (s, 1.7H), 1.54 – 1.47 (m, 2.2H), 1.39 – 1.34 (m, 1H), 1.07 (t, J = 7.4 Hz, 5.8H), 0.96 (d, J = 6.8 Hz, 3.2H), 0.90 (d, J = 6.8 Hz, 2.4H). ^{13}C NMR (101 MHz, $CDCl_3$) δ 158.60, 158.02, 135.56, 135.12, 130.12, 130.06, 129.04, 128.23, 128.20, 128.12, 127.94, 126.97, 125.30, 113.22, 113.20, 87.45, 86.52, 73.06, 65.03, 55.23, 43.17, 43.04, 41.36, 35.25, 33.94, 29.71, 24.37. **^{31}P NMR** (162 MHz, $CDCl_3$) δ 147.47, 146.45. **HRMS** (ESI$^+$): m/z [M+H]$^+$ calc. $C_{46}H_{61}N_7O_9P$ 886.4263; found 886.4201 and m/z [M+Na]$^+$ calc. $C_{46}H_{60}N_7O_9PNa$ 908.4082; found 908.4040.

5′-*O*-(4,4′-dimethoxytrityl)-2′-deoxy-2′-fluoro-thymidine -3′-*O*-[(*O*-(*N*-trifluoacetyl-piperidine-4-yl)-*N,N*′-diisopropylphosphoramidite] (20): The 2′-fluoro nucleoside **8** (600 mg, 1.07 mmol, 1.0 equiv.) was dissolved in dry, degassed DCM (20 mL). Phosphitylating reagent **5** (684 mg, 1.60 mmol, 1.5 eq) was added. The reaction mixture was purged with argon for 5 minutes. Tetrazole (0.45M in MeCN, 2.37 mL, 1.07 mmol, 1.0 equiv.) was added and the reaction mixture was left to stir at rt for 16h. Following a TLC analysis, the reaction mixture was diluted with DCM (20 mL), transferred to a separatory funnel and washed with saturated NaCl solution (30 mL). The DCM part was collected, passed though dry $MgSO_4$,and concentrated on a rotary evaporator. The crude residue was purified by flash chromatography (60:40 Hex:EtOAc, mixed with 1% triethylamine) to give the desired phosphoramidite **20** as a white solid (510 mg, 574 µmol, 54%). The compound **20** had R_f 0.75, 0.60 (1:1 Hex:EtOAc). **^{1}H NMR** (400 MHz, $CDCl_3$) δ 8.58 (s, 1H), 7.64 (s, 0.3H), 7.54 (d, J = 1.5 Hz, 0.5H), 7.39 (ddd, J = 7.8, 4.5, 1.6 Hz, 2.3H), 7.34 – 7.21 (m, 8.7H), 6.83 (dt, J = 8.9, 2.7 Hz, 4.8H), 6.18 – 6.03 (m, 1.1H), 5.25 (q, $^2J_{H\text{-}F}$ = 52 Hz, J = 3.5 Hz, 0.3H), 5.16 – 5.06 (m, 0.6H), 4.97 (dd, J = 4.5, 2.1 Hz, 0.3H), 4.67 – 4.63 (m, 0.2H), 4.59 – 4.47 (m, 1H), 4.29 – 4.08 (m, 3.1H), 3.79 (s, 6.3H), 3.85 – 3.70 (m, 0.8H), 3.70 – 3.48 (m, 2H), 3.32 (ddd, J = 13.7, 10.8, 3.0 Hz, 1H), 1.91 – 1.75 (m, 1H), 1.67 – 1.62 (m, 0.9H), 1.54 – 1.49 (m, 0.9H), 1.30 – 1.14 (m, 18.2H), 1.01 (d, J = 6.8 Hz, 3.6H). **^{13}C NMR** (101 MHz, $CDCl_3$, partial) δ 163.55, 158.99, 158.95, 150.23, 150.04, 144.24, 144.07, 135.47, 135.14, 130.48, 130.31, 128.55, 128.35, 128.13, 127.47, 127.39, 113.41, 113.38, 113.30, 111.59, 93.23, 87.10, 81.90, 61.25, 60.53, 55.38, 44.65, 44.60, 43.60, 43.47, 43.28, 40.22, 33.20, 32.27, 24.72, 24.66, 23.81, 23.79, 22.75, 21.19, 14.33, 11.80, 11.69. **^{31}P NMR** (162 MHz, $CDCl_3$) δ 150.00, 149.95, 149.89, 149.84, 149.15, 149.05, 148.67, 148.57. **^{19}F NMR** (565 MHz, $CDCl_3$) δ -68.92 – 69.00 (m), -203.12 (dq, J = 52.2, 15.2 Hz), -203.62 (dq, J = 52.2, 14.9 Hz). **HRMS** (ESI$^+$): m/z [M+H]$^+$ calc. $C_{44}H_{54}F_4N_4O_9P$ 889.3559; found 889.3557 and m/z [M+Na]$^+$ calc. $C_{44}H_{53}F_4N_4O_9PNa$ 911.3379; found 911.3371.

5′-*O*-(4,4′-dimethoxytrityl)-2′-*O*,4′-*C*-methylenethymidine-3′-*O*-[(*O*-(*N*-trifluoacetyl-piperidine-4-yl)-*N,N*′-diisopropylphosphoramidite] (21): The locked nucleic acid intermediate **12** (600 mg, 1.05 mmol,) was dissolved in dry, degassed DCM (15 mL). Phosphitylating reagent **5** (672 mg, 1.57 mmol, 1.5 equiv.) was added and the solution was purged with argon for 5 minutes. Tetrazole solution (0.45M in MeCN, 2.33 mL, 1.05 mmol, 1.0 equiv.) was added and the reaction was left to stir at rt for 16h. At completion, the reaction was diluted with 20 mL of DCM, transferred to a separatory funnel and washed with saturated

NaCl solution. The DCM part was collected, passed through anhydrous $MgSO_4$ and concentrated to dryness on a rotary evaporator. The residue was purified by flash chromatography (55:45 Hex:EtOAc, mixed with 1% triethylamine) to give the DMT-LNA-T phosphoramidite **21** as a white solid (525 mg, 584 µmol, 56%). The compound **21** had R_f 0.65, 0.55 (1:1 Hex:EtOAc). ^{1}H NMR (400 MHz, $CDCl_3$) δ 8.72 (s, 2H), 7.66 (d, J = 11.1 Hz, 1.5H), 7.39 (dt, J = 8.0, 4.7 Hz, 3H), 7.34 – 7.21 (m, 7.1H), 7.26 – 7.14 (m, 3.6H), 6.78 (ddd, J = 8.5, 5.1, 2.6 Hz, 6.3H), 5.61 (d, J = 4.1 Hz, 1.5H), 4.60 (d, J = 11.2 Hz, 0.8H), 4.45 (d, J = 10.0 Hz, 0.6H), 4.30 (d, J = 9.5 Hz, 0.6H), 4.14 – 4.01 (m, 2H), 3.85 – 3.76 (m, 0.7H), 3.77 (s, 1H), 3.76 (d, J = 3.2 Hz, 1.9H), 3.73 (s, 9.5H), 3.69 (s, 0.8H), 3.65 – 3.46 (m, 4.1H), 3.45 – 3.27 (s, 7H), 1.96 (d, J = 17.1 Hz, 1.3H), 1.76 – 1.64 (m, 1H), 1.68 – 1.48 (m, 5H), 1.44 – 1.36 (m, 1H), 1.27 – 1.13 (m, 4.8H), 1.16 – 1.00 (m, 12.9H), 0.97 – 0.93 (m, 4.6H). **^{13}C NMR** (101 MHz, $CDCl_3$) δ 163.81, 158.95, 158.87, 149.75, 144.44, 144.21, 135.45, 135.37, 135.20, 134.64, 130.46, 130.36, 130.28, 130.19, 128.48, 128.24, 128.09, 127.41, 127.26, 113.38, 113.34, 113.30, 110.59, 110.52, 88.11, 87.66, 87.56, 86.86, 78.32, 72.54, 72.30, 67.72, 60.52, 58.25, 55.35, 53.56, 43.50, 43.37, 42.27, 39.88, 33.24, 32.26, 31.71, 24.76, 24.69, 24.54, 22.78, 14.33, 14.24, 12.65, 12.51. **^{31}P NMR** (162 MHz, $CDCl_3$) δ 148.12, 148.10, 147.69, 147.18. **^{19}F NMR** (376 MHz, $CDCl_3$) δ -68.92, -68.94, -68.98, -68.99. **HRMS** (ESI$^+$): m/z [M+H]$^+$ calc. $C_{45}H_{55}F_3N_4O_{10}P$ 899.3602; found 899.3595 and m/z [M+Na]$^+$ calc. $C_{45}H_{54}F_3N_4O_{10}PNa$ 921.3422; found 921.3440.

2.0 Oligonucleotide Synthesis and Analysis

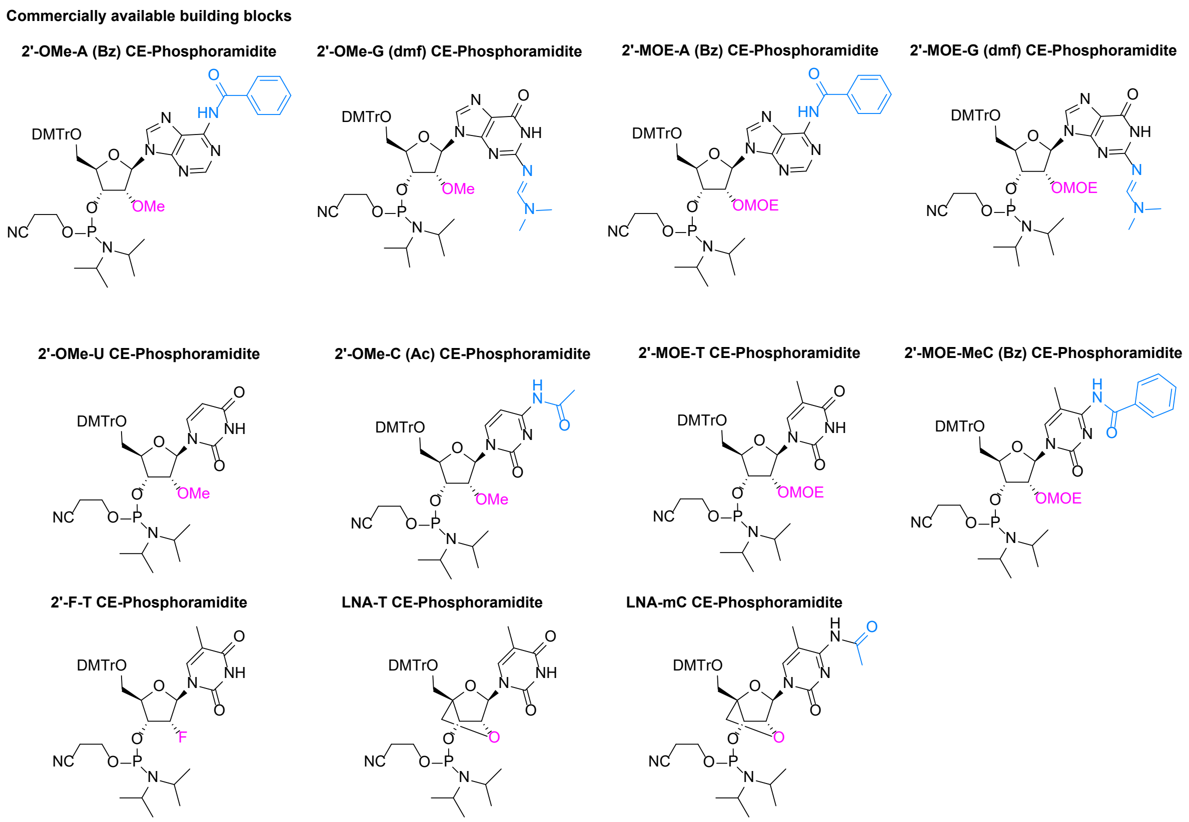

Figure S2: List of phosphoramidite monomers used in solid phase oligonucleotide synthesis.

2.1 Oligonucleotide Synthesis, Deprotection and Purification

2′OMePS+, 2′OMePS-, and modified oligonucleotide synthesis

Oligonucleotides were synthesised on an Applied Biosystems 394 automated DNA/RNA synthesiser using a standard phosphoramidite cycle of detritylation, coupling, capping, and sulfurisation/oxidation on a 1.0 µmole scale. Detritylation, coupling, capping, oxidation and activation reagents are identical to those used for standard DNA/RNA synthesis. Trichloroacetic acid (TCA) (3% in CH_2Cl_2) was used for detritylation, 5-Benzylthio-1*H*-tetrazole (BTT) (0.3 M in MeCN) was used as an activator. Sulfurization was achieved using 3-ethoxy-1,2,4-dithiazole-5-one and oxidation was achieved by using iodine/water. Pre-packed nucleoside SynBase™ CPG 1000/110 resins (Link Technologies) were used, and β-cyanoethyl phosphoramidite monomers (DMT-2′O-methyl-rA(Bz), DMT-2′O-methyl-rG(dmf), DMT-2′-O-methyl-rC(Ac), DMT-2′O-methyl-rU, DMT-2′O-methoxyethyl-rA(Bz), DMT-2′O-methoxyethyl-rG(dmf), DMT-2′-O-methoxyethyl-rC(Me)(Bz), DMT-2′O-methoxyethyl-rU and DMT-LNA-T and DMT-LNA-mC CE-Phosphoramidites were dissolved in anhydrous MeCN (10% CH_2Cl_2 was added when 2′OMe U phosphoramidite was used) to a concentration of 0.1 M immediately prior to use with a coupling time of 6 min.

Phosphoramidite monomers synthesised for this project (section 1.2 above) were dissolved to a concentration of 0.1 M in MeCN immediately prior to use and used with a coupling time of 6 min. Stepwise coupling efficiencies were determined by automated trityl cation conductivity monitoring and were >97% in all cases. In all cases after solid phase oligonucleotide synthesis the DMT group at the 5′-position was not removed from the oligonucleotide. The oligonucleotide was subject to cleavage from the solid support and deprotected and purified by HPLC to give purified DMT-ON oligonucleotides. The DMT group was then cleaved in liquid phase by treatment with 80% aqueous AcOH.

Deprotection from the solid support (1.0 µmol Scale)

Protocol 1: Cleavage and deprotection were achieved by treatment with 0.5 mL of THF and 0.5 mL of ethylenediamine (EDA) for 2 hrs room temperature. The mixture of EDA-THF was discarded washed with THF (1mL) if needed and dried and then the resin was washed with 1 mL of DNase free water to give the crude oligonucleotides. The oligonucleotides were desalted using NAP-10 columns (Cytiva). The DMT-ON oligonucleotides were purified by HPLC and then DMT group was removed by using the procedure below.

Protocol 2: Cleavage from the solid support and deprotection of protecting groups were achieved by treatment with 2 mL of 35% aqueous ammonia for 16-24 hours at room temperature. The solids were filtered by (0.5 µm pore size filter) then washed with water. The combined water fractions were concentrated on a rotary evaporator at 55 °C. The residue was dissolved in DNAse free water to give the crude oligonucleotides which were desalted using Nap-10 column (Cytiva).

Condition of DMT-group removal (100 nmol – 1.0 µmol Scale)

100 µL of 80% AcOH was added to the dry purified DMT-ON oligonucleotide. The reaction was left for 30 mins at rt. The solution became light red in colour. The reaction was quenched by 600 µL of water and 300 µL of 2M TEAA buffer (pH 8.5). The detritylated oligonucleotides were desalted using NAP-10 columns (Cytiva) and then freeze dried or purified by HPLC to give the final purified oligonucleotides which were characterised by UPLC-MS.

Synthesis of Complementary DNA, dTGTAACTGAGGTAAGAGG (5′→3′)

Please see reference 1

Synthesis of Complementary RNA, rUGUAACUGAGGUAAGAGG (5′→3′)

Please see reference 1

2.3 Calculated and Observed Masses of Oligonucleotides

Table T1:

Nucleoside in Black have 2´-OMe sugars. Nucleoside in blue have 2´-MOE sugars, nucleosides in red are locked nucleic acids, and nucleosides in purple have 2´-F sugars. Nucleosides in bold and under-lined are tetrahydropyran-4-yl phosphothiotriesters. Nucleosides in red lowercase are LNA-piperid-4-yl phosphothiotriesters. Nucleosides in purple lowercase are 2´-F -piperid-4-yl phosphothiotriesters. The LNA-C bases in Pal-LNA2(C)-PS are methylated at position 5. ‘o’ = phosphodiester linkage. Strikethrough = phosphotriester linkages.

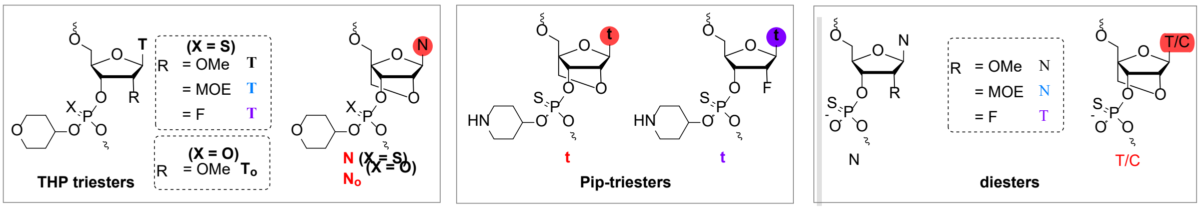

ON	Sequence (5’→3’)	Mass Expected	Mass Observed
**OMe$_2$**	CCU CU**T** ACC UCA GU**T** ACA	6295.17	6295.20
**OMe$_4$**	CC**T** CU**T** ACC **T**CA GU**T** ACA	6491.46	6491.60
**OMe$_6$**	CC**T** C**TT** ACC **T**CA G**TT** ACA	6687.75	6686.98
**MOE$_2$**	CCU CU**T** ACC UCA GU**T** ACA	6383.27	6383.60
**MOE$_4$**	CC**T** CU**T** ACC **T**CA GU**T** ACA	6667.67	6667.80
**MOE$_6$**	CC**T** C**TT** ACC **T**CA G**TT** ACA	6952.07	6952.40
**LNA$_2$-OMe$_2$**	C**C**U CU**T** ACC U**C**A GU**T** ACA	6459.37	6559.83
**LNA$_4$-OMe$_4$**	C**CT** CU**T** A**CC** **TC**A GU**T** A**C**A	6819.87	6818.98
**LNA$_7$-OMe$_6$**	**CCT** **CTT** A**CC** **TC**A G**TT** A**C**A	7262.46	7261.29
**2´MOEPS+**	CCT CTT ACC TCA GTT ACA	7030.13	7030.18
**LNA$_2$-MOE$_2$**	C**C**T CT**T** ACC T**C**A GT**T** ACA	7246.41	7244.35
**LNA$_4$-MOE$_4$**	C**CT** CT**T** A**CC** **TC**A GT**T** A**C**A	7462.69	7462.48
**LNA$_7$-MOE$_6$**	**CCT** **CTT** A**CC** **TC**A G**TT** A**C**A	7702.99	7700.52
**F$_2$**	CCU CU**T** ACC UCA GU**T** ACA	6271.10	6270.63
**F$_4$**	CC**T** CU**T** ACC **T**CA GU**T** ACA	6443.31	6442.78
**F$_6$**	CC**T** C**TT** ACC **T**CA G**TT** ACA	6615.53	6615.81
**LNA$_2$-F$_2$**	CC**T** CU**T** ACC **T**CA GU**T** ACA	6463.35	6462.80
**LNA$_2$-F$_4$**	CC**T** C**TT** ACC **T**CA G**TT** ACA	6635.57	6635.83
**LNA$_3$-F$_3$**	CC**T** C**TT** ACC **T**CA G**TT** ACA	6645.59	6644.85
**LNA$_4$-F$_2$**	CC**T** C**TT** ACC **T**CA G**TT** ACA	6655.61	6654.86
**Pip-LNA$_2$**	CCU CUt ACC UCA GUt ACA	6289.17	6288.67
**Pip-LNA$_4$**	CCt CUt ACC tCA GUt ACA	6479.46	6478.86
**Pip-LNA$_6$**	CCt Ctt ACC tCA Gtt ACA	6669.75	6669.05
**Pip-F$_2$**	CCU CUt ACC UCA GUt ACA	6269.13	6268.65
**Pip-F$_4$**	CCt CUt ACC tCA GUt ACA	6439.38	6438.82
**Pip-F$_6$**	CCt Ctt ACC tCA Gtt ACA	6609.63	6608.99

**LNA$_{11}$-OMe$_6$-PO**	C$_o$C$_o$T$_o$C$_o$T$_o$T$_o$A$_o$C$_o$C$_o$T$_o$C$_o$A$_o$G$_o$T$_o$T$_o$A$_o$C$_o$A	7317.83	7317.7
**Lipid conjugates of charge neutral backbone oligonucleotides**			
**Pal-OMe$_4$**	Pal-CCT CUT ACC TCA GUT ACA	6925.09	6925.30
**Pal-MOE$_4$**	Pal-CCT CUT ACC TCA GUT ACA	7101.30	7101.50
**Pal-LNA$_4$**	Pal-CCT CUT ACC TCA GUT ACA	6917.02	6917.10
**Pal-LNA$_4$-PS**	Pal-CCT CUT ACC TCA GUT ACA	6580.55	6580.30
**Pal$_2$-LNA$_4$**	Pal$_2$-CCT CUT ACC TCA GUT ACA	7719.04	ESI-MS
**Pal-Pip-LNA$_4$**	Pal-CCt CUt ACC tCA GUt ACA	6913.09	6913.14
**Pal-Pip-F$_4$**	Pal-CCt CUt ACC tCA GUt ACA	6873.01	6873.10
**Pal-SS**	Pal-SS-CCU CUU ACC UCA GUU ACA	6876.99	6877.00
**C16-SS**	C16-SS-CCU CUU ACC UCA GUU ACA	6763.83	6763.70
**Pal-HEG**	Pal-HEG-CCUCAUUCACUCGAUUCA	6876.81	6877.00
**Pal-HEG-PO**	Pal$_o$-HEG$_o$-CCUCAUUCACUCGAUUCA	6860.74	6861.00
**+Control-Pal**	Pal-CCU CUU ACC UCA GUU ACA	6532.51	6532.70
**+Control-Pal$_2$**	Pal$_2$-CCUCAUUCACUCGAUUCA	7329.30	ESI-MS
**-Control-Pal**	Pal-CCU CAU UCA CUC GAU UCA	6532.51	6532.60
**Pal-LNA$_2$-OMe$_2$**	Pal-CCU CUT ACC UCA GUT ACA	6893.01	6893.12
**Pal-LNA$_2$(C)-PS**	Pal-CCU CUU ACC UCA GUU ACA	6556.54	6566.56
**Pal-LNA$_2$-F$_2$**	Pal-CCT CUT ACC TCA GUT ACA	6896.99	6897.08
**Pal-LNA$_2$-F$_2$-PS**	Pal-CCT CUT ACC TCA GUT ACA	6532.46	6532.76
**Pal-Pip-LNA$_2$**	Pal-CCU CUt ACC UCA GUt ACA	6722.80	6722.95
**Pal-LNA$_2$-PS**	Pal-CCU CUT ACC UCA GUT ACA	6556.54	6555.86
**Pal-F$_2$-PS**	Pal-CCU CUT ACC UCA GUT ACA	6508.44	6506.80
**Pal-MOE$_2$-PS**	Pal-CCU CUT ACC UCA GUT ACA	6648.67	6647.93
**Pal-2´MOEPS+**	Pal-CCU CUU ACC UCA GUU ACA	7463.77	7463.47

2.4 Yields of Oligonucleotide Synthesis (1.0 µmol Scale)

Table T2:

Nucleoside in Black have 2´-OMe sugars. Nucleoside in blue have 2´-MOE sugars, nucleosides in red are locked nucleic acids, and nucleosides in purple have 2´-F sugars. Nucleosides in bold and underlined are tetrahydropyran-4-yl phosphothiotriesters. Nucleosides in red lowercase are LNA-piperid-4-yl phosphothiotriesters. Nucleosides in purple lowercase are 2´-F -piperid-4-yl phosphothiotriesters. The LNA-C bases in Pal-LNA2(C)-PS are methylated at position 5. 'o' = phosphodiester linkage. Strikethrough = phosphotriester linkages.

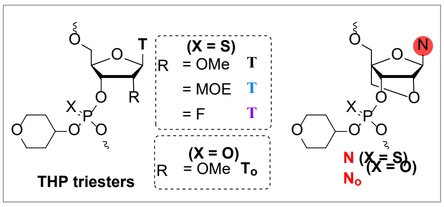

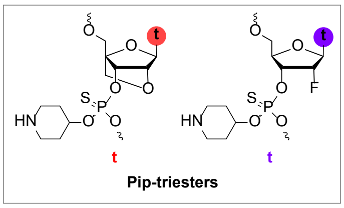

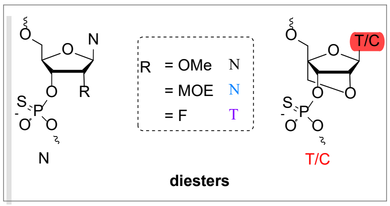

ON	Sequence (5'→3')	Product Obtained (nm)	Yield (%)
OMe$_2$	CCU CUT ACC UCA GUT ACA	569	57
OMe$_4$	CCT CUT ACC TCA GUT ACA	362	36
OMe$_6$	CCT CTT ACC TCA GTT ACA	522	52
MOE$_2$	CCU CUT ACC UCA GUT ACA	538	54
MOE$_4$	CCT CUT ACC TCA GUT ACA	356	36
MOE$_6$	CCT CTT ACC TCA GTT ACA	449	45
LNA$_2$-OMe$_2$	CCU CUT ACC UCA GUT ACA	464	46
LNA$_4$-OMe$_4$	CCT CUT ACC TCA GUT ACA	525	53
LNA$_7$-OMe$_6$	CCT CTT ACC TCA GTT ACA	306	31
2´MOEPS+	CCT CTT ACC TCA GTT ACA	420	42
LNA$_2$-MOE$_2$	CCT CTT ACC TCA GTT ACA	510	51
LNA$_4$-MOE$_4$	CCT CTT ACC TCA GTT ACA	403	40
LNA$_7$-MOE$_6$	CCT CTT ACC TCA GTT ACA	405	41
F$_2$	CCU CUT ACC UCA GUT ACA	524	52
F$_4$	CCT CUT ACC TCA GUT ACA	564	56
F$_6$	CCT CTT ACC TCA GTT ACA	546	55
LNA$_2$-F$_2$	CCT CUT ACC TCA GUT ACA	608	61
LNA$_2$-F$_4$	CCT CTT ACC TCA GTT ACA	518	52
LNA$_3$-F$_3$	CCT CTT ACC TCA GTT ACA	602	60
LNA$_4$-F$_2$	CCT CTT ACC TCA GTT ACA	576	58
Pip-LNA$_2$	CCU CUt ACC UCA GUt ACA	528	53
Pip-LNA$_4$	CCt CUt ACC tCA GUt ACA	556	56
Pip-LNA$_6$	CCt Ctt ACC tCA Gtt ACA	392	39
Pip-F$_2$	CCU CUt ACC UCA GUt ACA	314	31
Pip-F$_4$	CCt CUt ACC tCA GUt ACA	327	33
Pip-F$_6$	CCt Ctt ACC tCA Gtt ACA	197	20

**LNA$_{11}$-OMe$_{6}$-PO**	C$_o$C$_o$T$_o$C$_o$T$_o$T$_o$A$_o$C$_o$C$_o$T$_o$C$_o$A$_o$G$_o$T$_o$T$_o$A$_o$C$_o$A	15	2
**Lipid conjugates of charge neutral backbone oligonucleotides**			
**Pal-OMe$_4$**	Pal-CCT CUT ACC TCA GUT ACA	421	42
**Pal-MOE$_4$**	Pal-CCT CUT ACC TCA GUT ACA	441	44
**Pal-LNA$_4$**	Pal-CCT CUT ACC TCA GUT ACA	244	24
**Pal-LNA$_4$-PS**	Pal-CCT CUT ACC TCA GUT ACA	610	61
**Pal$_2$-LNA$_4$**	Pal$_2$-CCT CUT ACC TCA GUT ACA	248	25
**Pal-Pip-LNA$_4$**	Pal-CCt CUt ACC tCA GUt ACA	634	63
**Pal-Pip-F$_4$**	Pal-CCt CUt ACC tCA GUt ACA	138	14
**Pal-SS**	Pal-SS-CCU CUU ACC UCA GUU ACA	177	18
**C16-SS**	C16-SS-CCU CUU ACC UCA GUU ACA	127	13
**Pal-HEG**	Pal-HEG-CCU CAU UCA CUC GAU UCA	455	45
**Pal-HEG-PO**	Pal$_o$-HEG$_o$-CCU CAU UCA CUC GAUUCA	413	41
**+Control-Pal**	Pal-CCU CUU ACC UCA GUU ACA	550	55
**+Control-Pal$_2$**	Pal$_2$-CCU CAU UCA CUC GAU UCA	248	25
**-Control-Pal**	Pal-CCU CAU UCA CUC GAU UCA	--	--
**Pal-LNA$_2$-OMe$_2$**	Pal-CCU CUT ACC UCA GUT ACA	554	55
**Pal-LNA$_2$(C)-PS**	Pal-CCU CUU ACC UCA GUU ACA	526	53
**Pal-LNA$_2$-F$_2$**	Pal-CCT CUT ACC TCA GUT ACA	513	51
**Pal-LNA$_2$-F$_2$-PS**	Pal-CCT CUT ACC TCA GUT ACA	564	56
**Pal-Pip-LNA$_2$**	Pal-CCU CUt ACC UCA GUt ACA	571	57
**Pal-LNA$_2$-PS**	Pal-CCU CUT ACC UCA GUT ACA	649	65
**Pal-F$_2$-PS**	Pal-CCU CUT ACC UCA GUT ACA	620	62
**Pal-MOE$_2$-PS**	Pal-CCU CUT ACC UCA GUT ACA	628	63
**Pal-2´MOEPS+**	Pal-CCU CUU ACC UCA GUU ACA	554	55

2.5 UPLC-ESI-MS of Oligonucleotides

2.5.1 2´-OMe oligonucleotides

**OMe2**	CCU CU<u>**T**</u> ACC UCA GU<u>**T**</u> ACA	

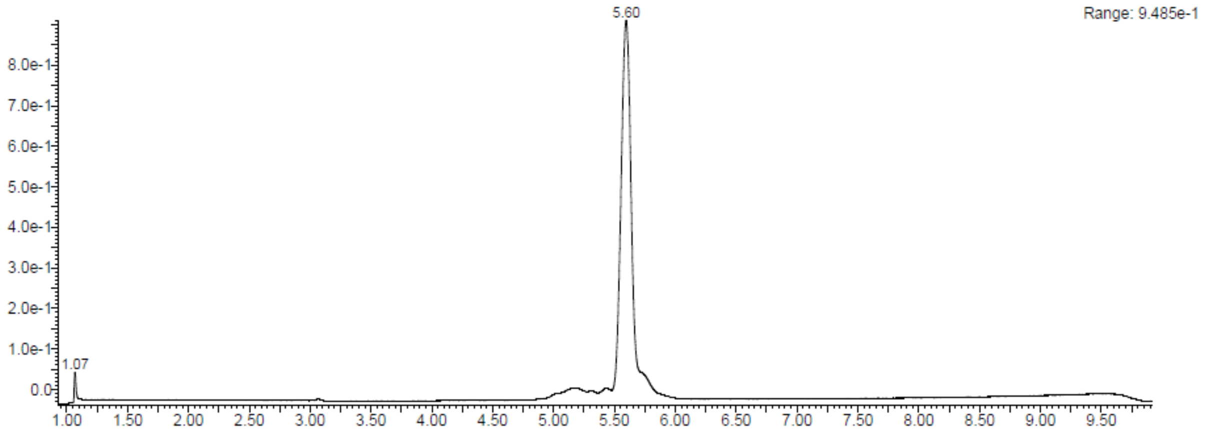

Figure S3: Reverse-phase UPLC of **OMe2** (UV absorbance at 260 nm vs time in min).

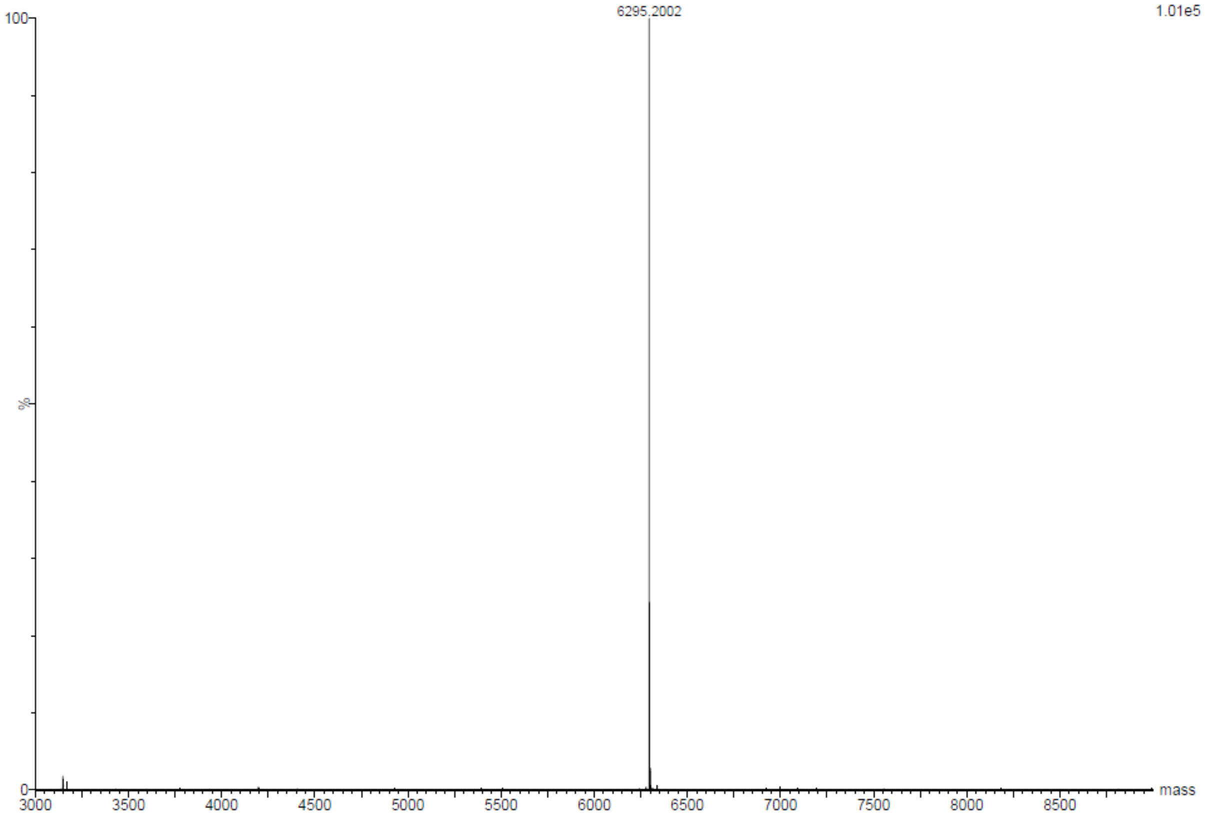

Figure S4: Mass spectrum (ES-) of **OMe2**. Required **6295.17** Da, found **6295.20** Da. y-axis = relative intensity (%), x-axis = mass in Da.

**OMe4**	CC**T** CU**T** ACC **T**CA GU**T** ACA	

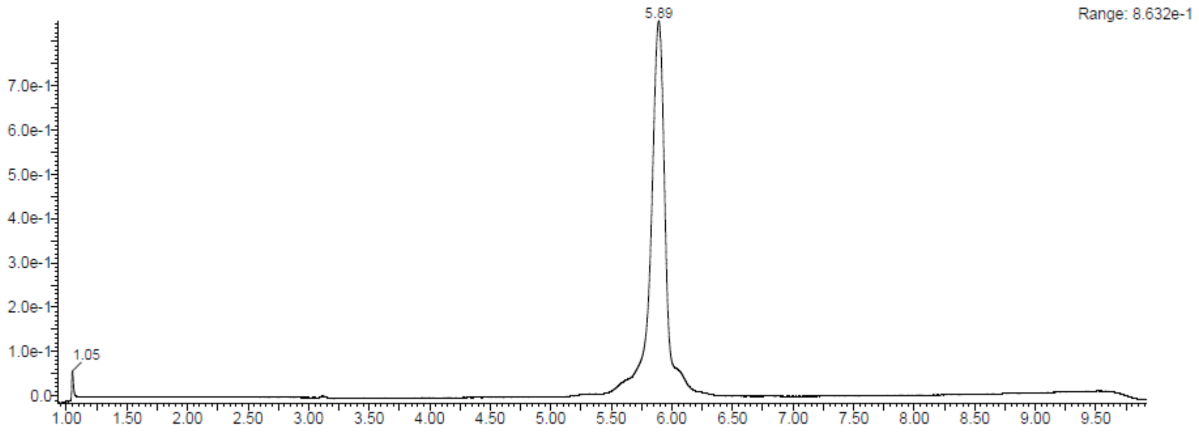

Figure S5: Reverse-phase UPLC of **OMe4** (UV absorbance at 260 nm vs time in min).

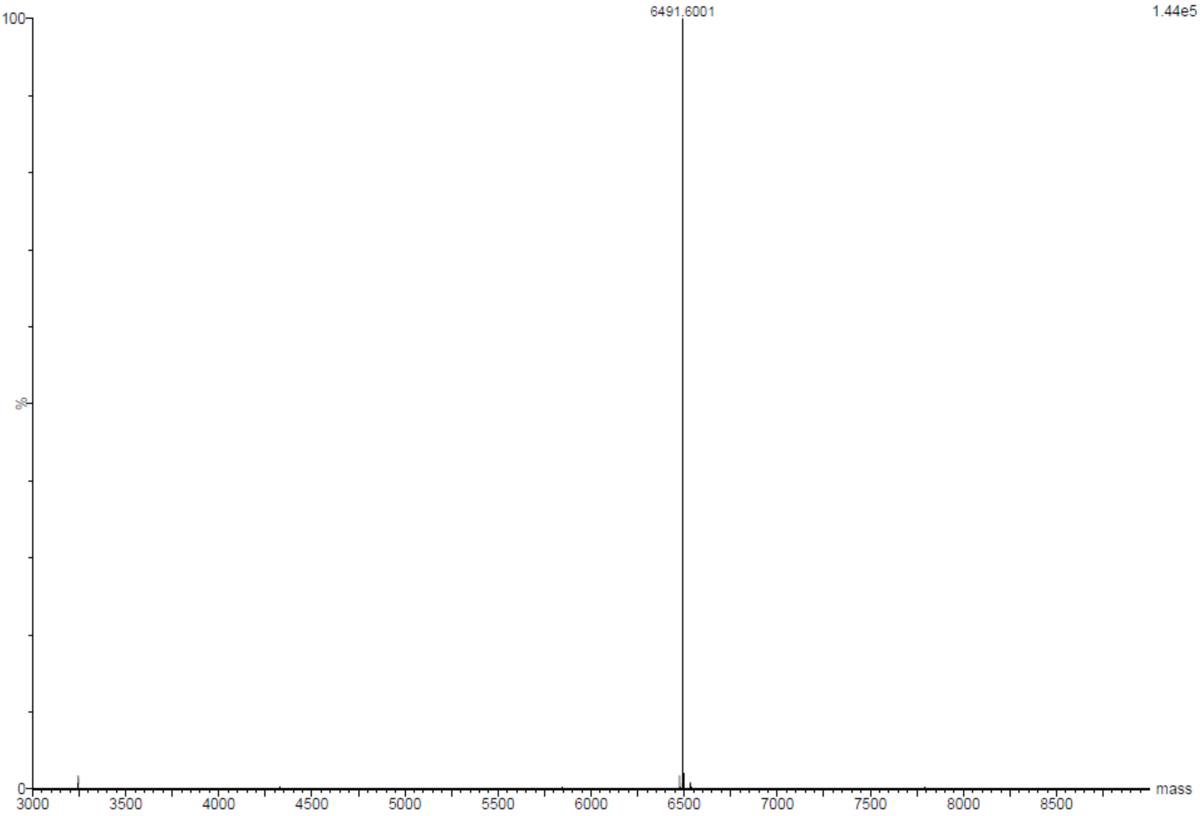

Figure S6: Mass spectrum (ES-) of **OMe4**. Required **6491.46** Da, found **6491.60** Da. y-axis = relative intensity (%), x-axis = mass in Da.

**OMe6**	CC**T** C**TT** ACC **T**CA G**TT** ACA	

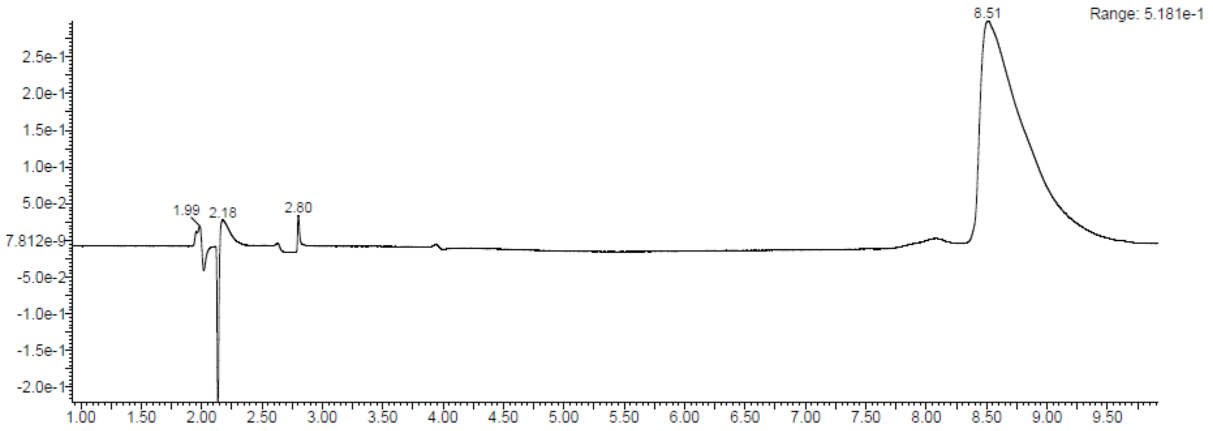

Figure S7: Reverse-phase UPLC of **OMe6** (UV absorbance at 260 nm vs time in min).

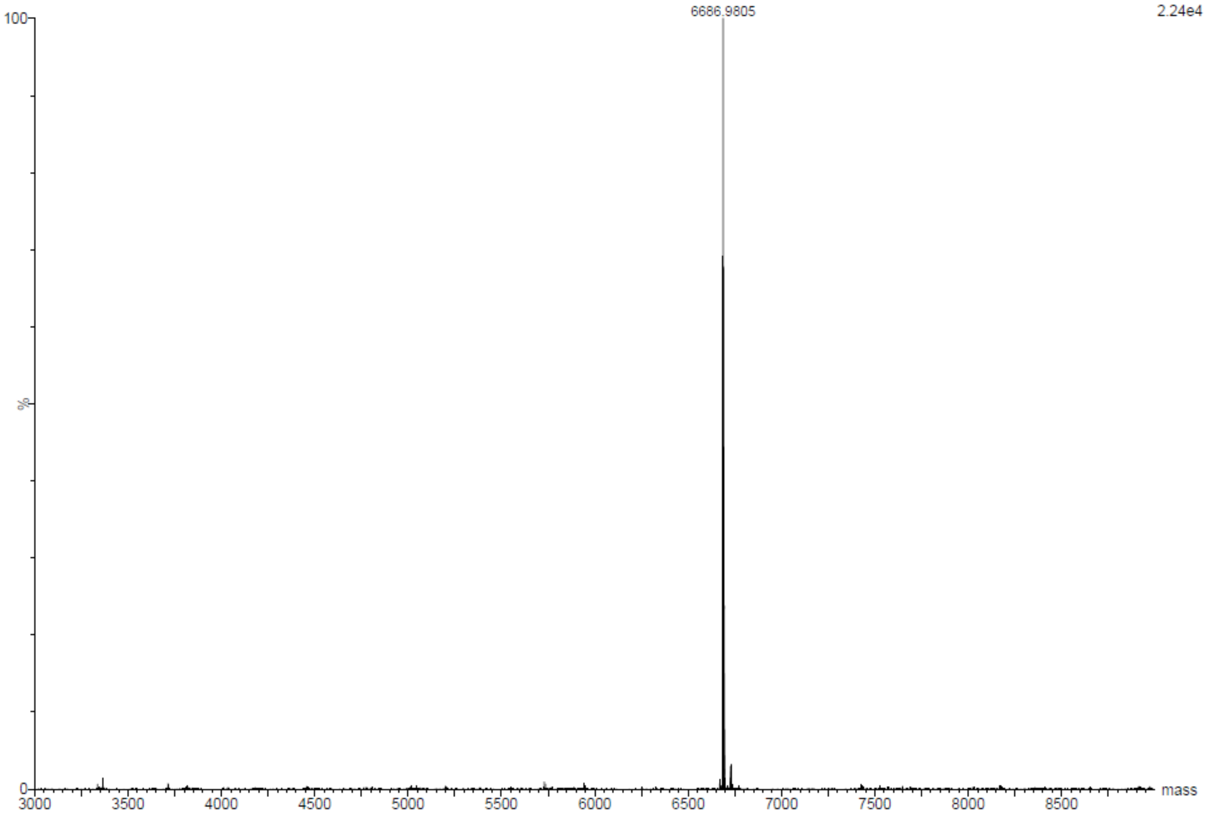

Figure S8: Mass spectrum (ES-) of **OMe6**. Required **6687.75** Da, found **6686.98** Da. y-axis = relative intensity (%), x-axis = mass in Da.

2.5.2 Mixed 2´-OMe and 2´-MOE chimeric oligonucleotides

**MOE2**	CCU CU**T** ACC UCA GU**T** ACA	

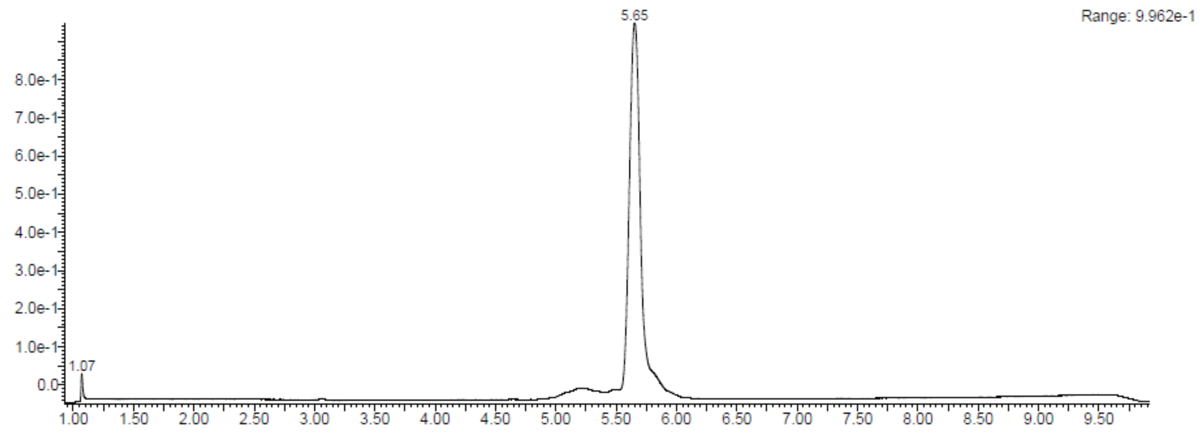

Figure S9: Reverse-phase UPLC of **MOE2** (UV absorbance at 260 nm vs time in min).

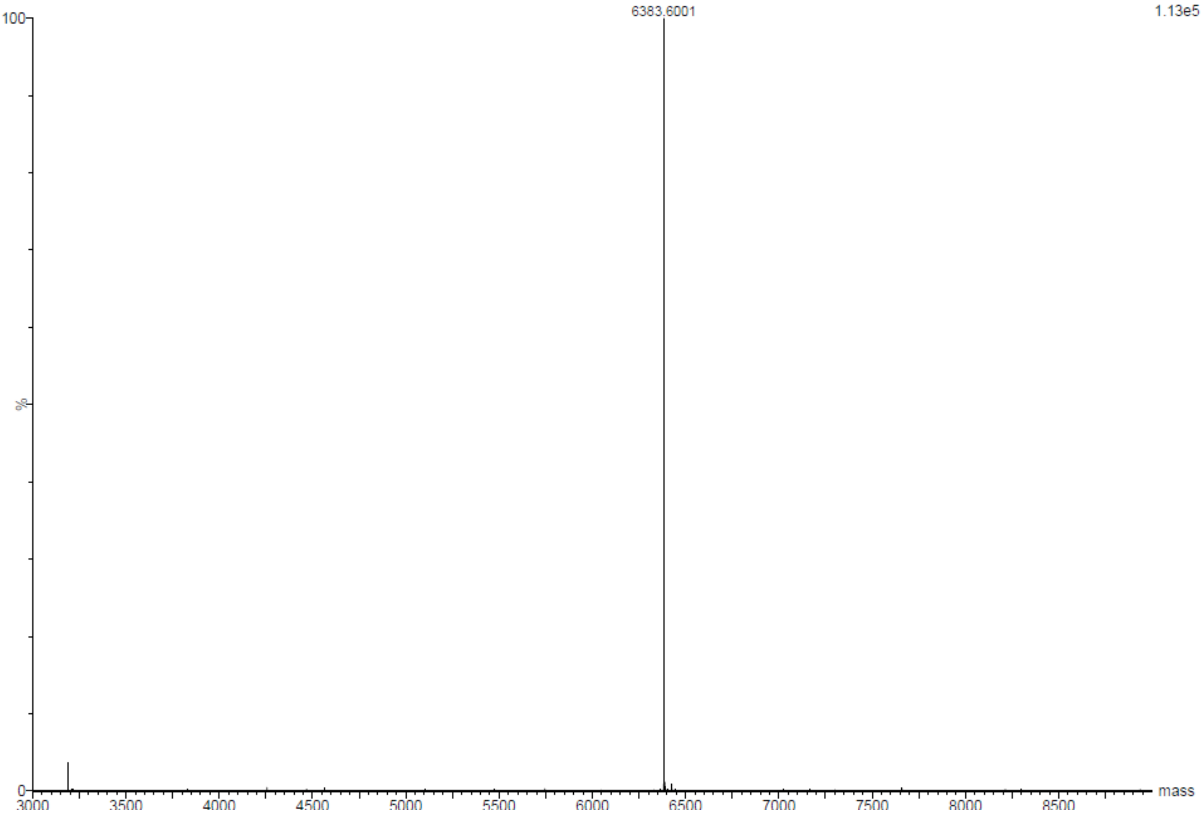

Figure S10: Mass spectrum (ES-) of **MOE2**. Required **6383.27** Da, found **6383.60** Da. y-axis = relative intensity (%), x-axis = mass in Da.

**MOE$_4$**	CC<u>T</u> CU<u>T</u> ACC <u>T</u>CA GU<u>T</u> ACA	

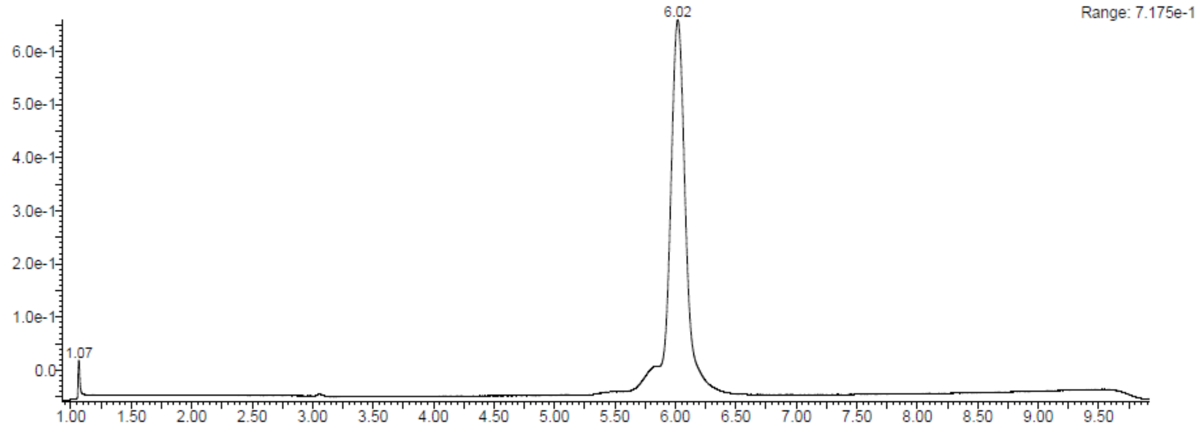

Figure S11: Reverse-phase UPLC of **MOE$_4$** (UV absorbance at 260 nm vs time in min).

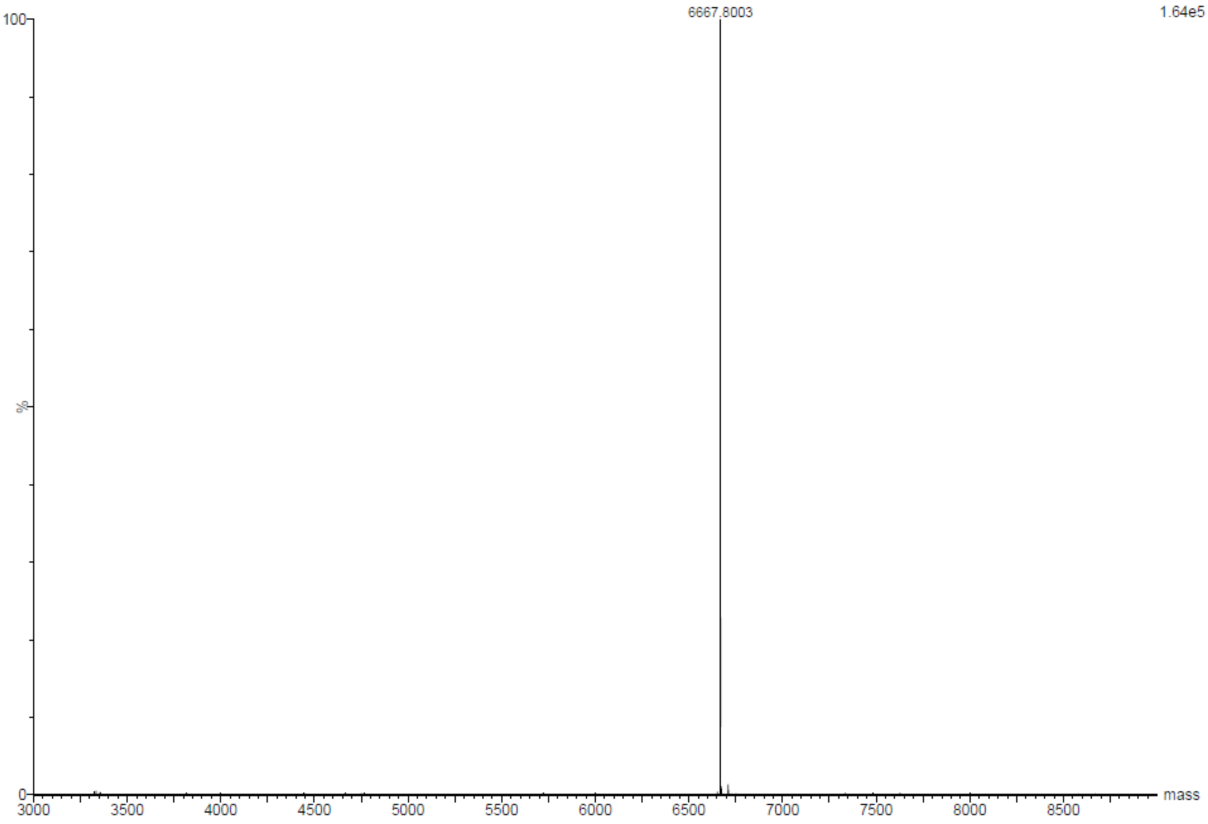

Figure S12: Mass spectrum (ES-) of **MOE$_4$**. Required **6667.67** Da, found **6667.80** Da. y-axis = relative intensity (%), x-axis = mass in Da.

**MOE$_6$**	CC**T** C**TT** ACC **T**CA G**TT** ACA	

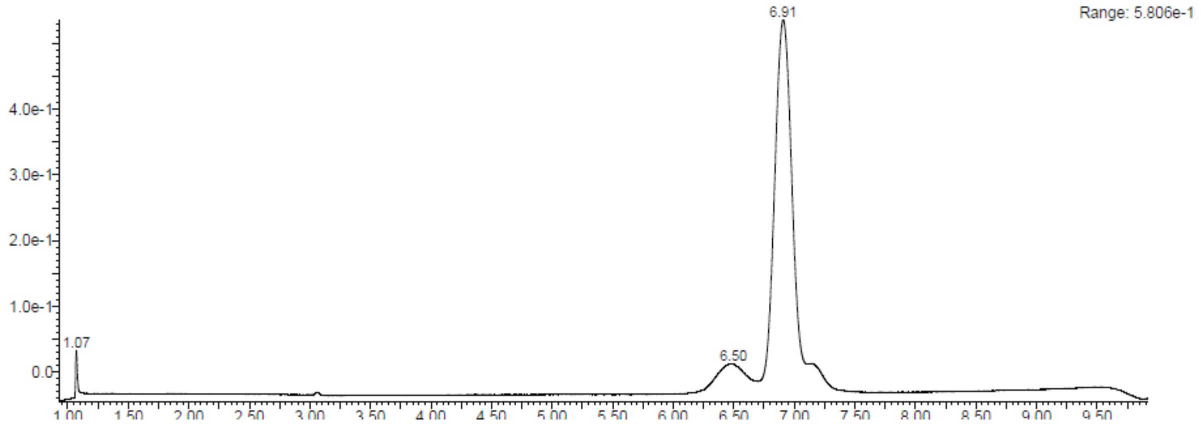

Figure S13: Reverse-phase UPLC of **MOE$_6$** (UV absorbance at 260 nm vs time in min).

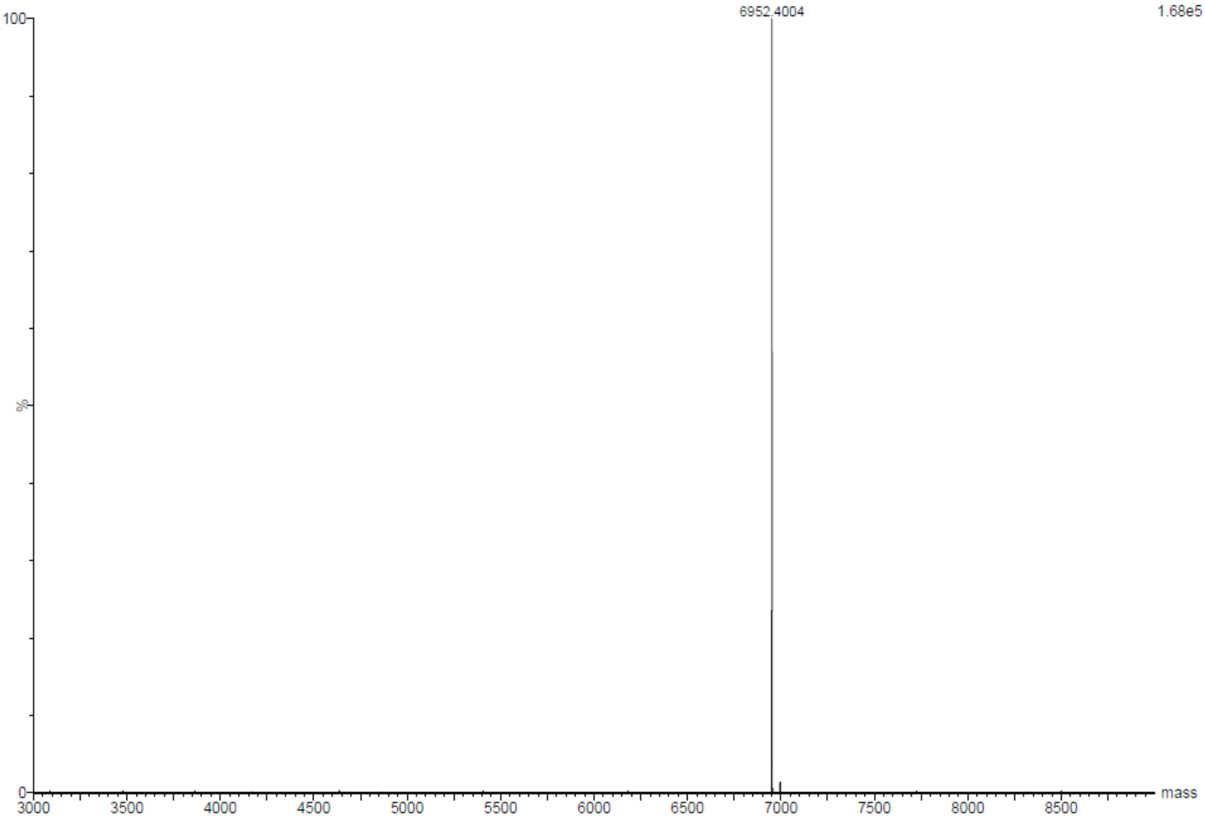

Figure S14: Mass spectrum (ES-) of **MOE$_6$**. Required **6952.07** Da, found **6952.40** Da. y-axis = relative intensity (%), x-axis = mass in Da.

2.5.3 Mixed 2´-OMe, 2´-MOE, and LNA chimeric oligonucleotides

**LNA$_2$-OMe$_2$**	CCU CUT ACC UCA GUT ACA	

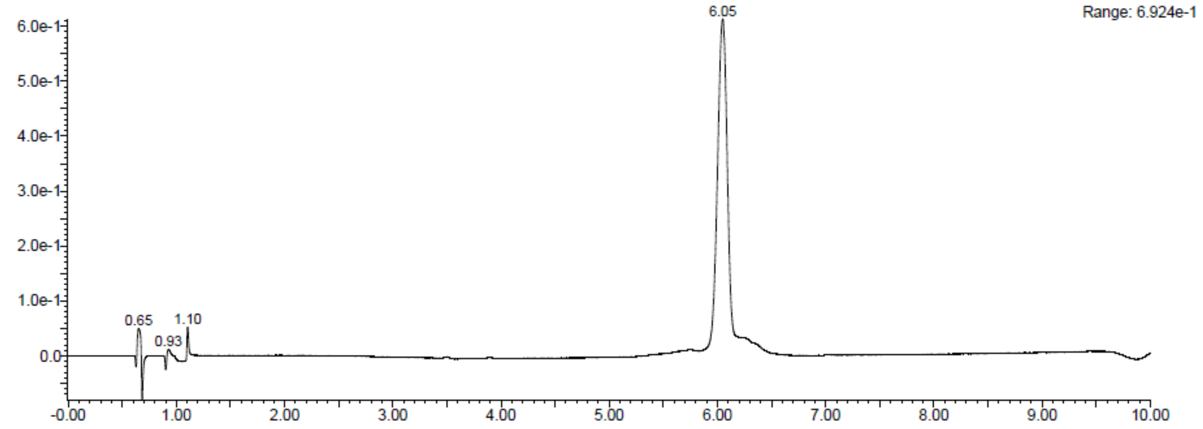

Figure S15: Reverse-phase UPLC of **LNA$_2$-OMe$_2$** (UV absorbance at 260 nm vs time in min).

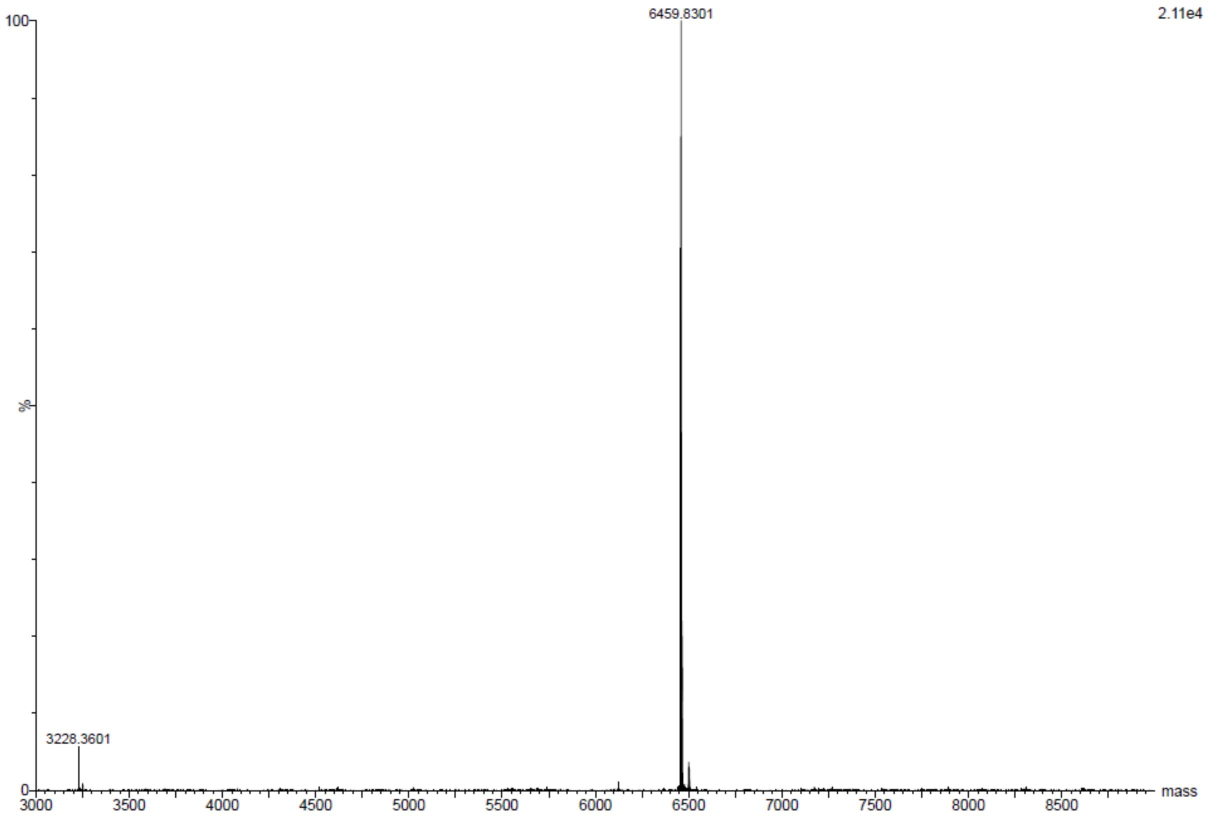

Figure S16: Mass spectrum (ES-) of **LNA$_2$-OMe$_2$.** Required **6459.37** Da, found **6459.83** Da. y-axis = relative intensity (%), x-axis = mass in Da.

**LNA_4-OMe_4**	CCT CUT ACC TCA GUT ACA	

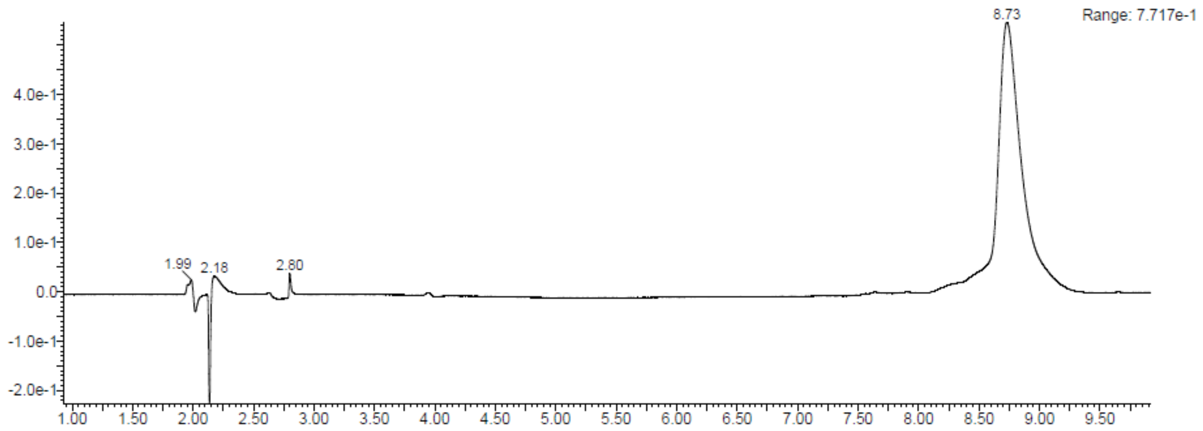

Figure S17: Reverse-phase UPLC of **LNA_4-OMe_4** (UV absorbance at 260 nm vs time in min).

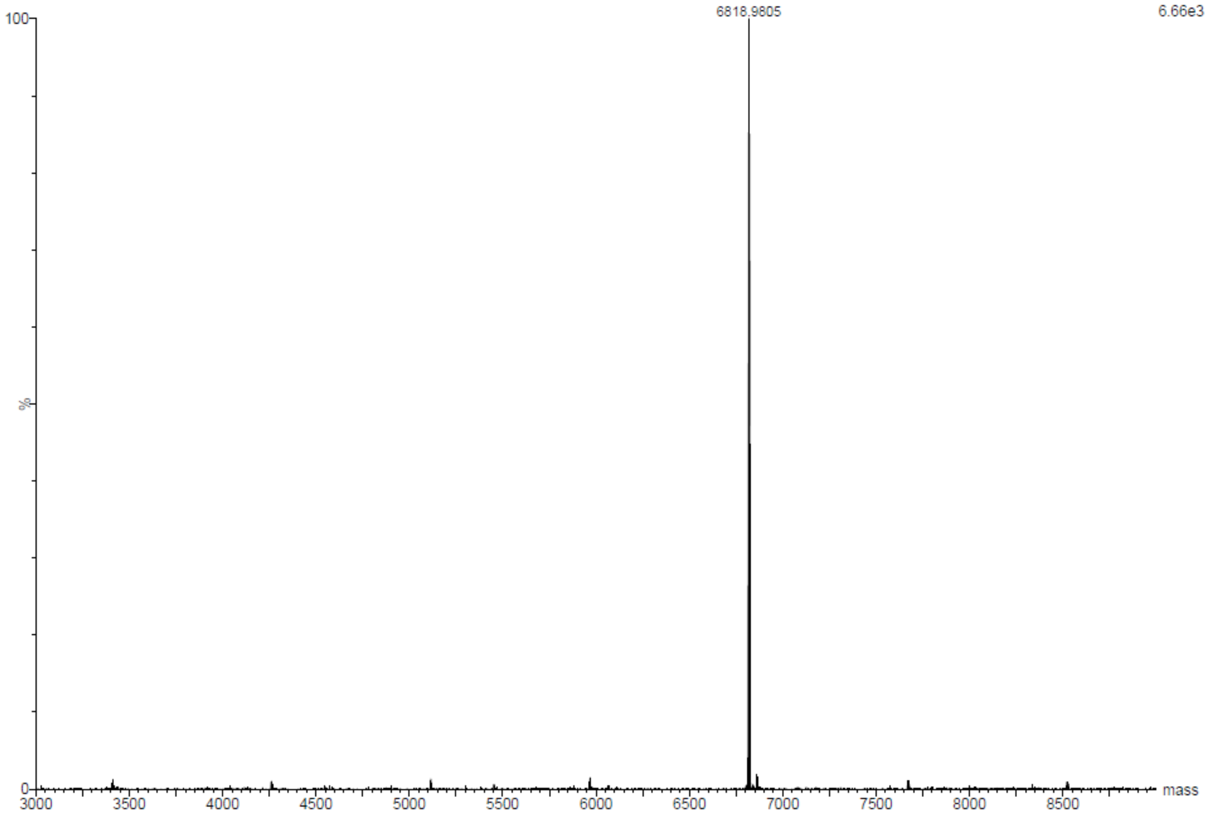

Figure S18: Mass spectrum (ES-) of **LNA_4-OMe_4.** Required **6819.87** Da, found **6818.98** Da. y-axis = relative intensity (%), x-axis = mass in Da.

**LNA7-OMe6**	CCT CTT ACC TCA GTT ACA	

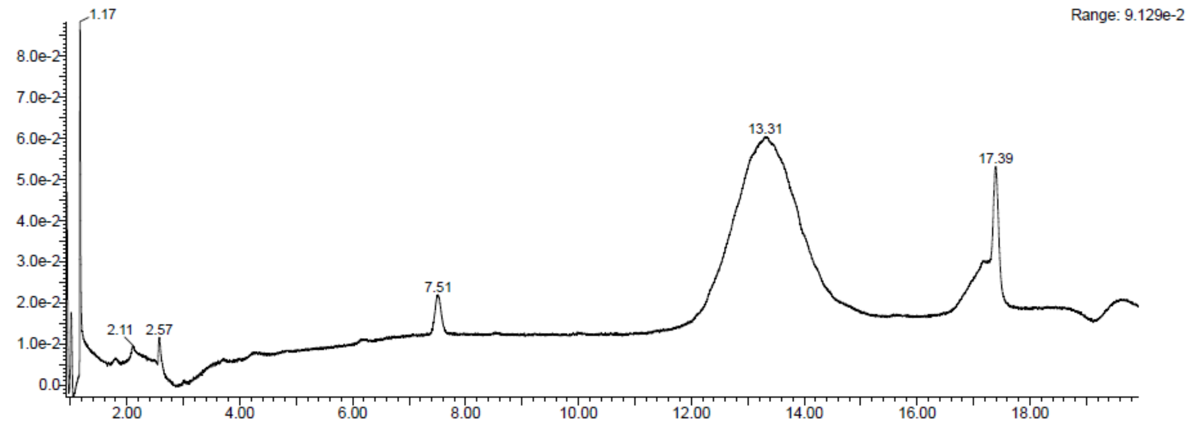

Figure S19: Reverse-phase UPLC of **LNA7-OMe6** (UV absorbance at 260 nm vs time in min).

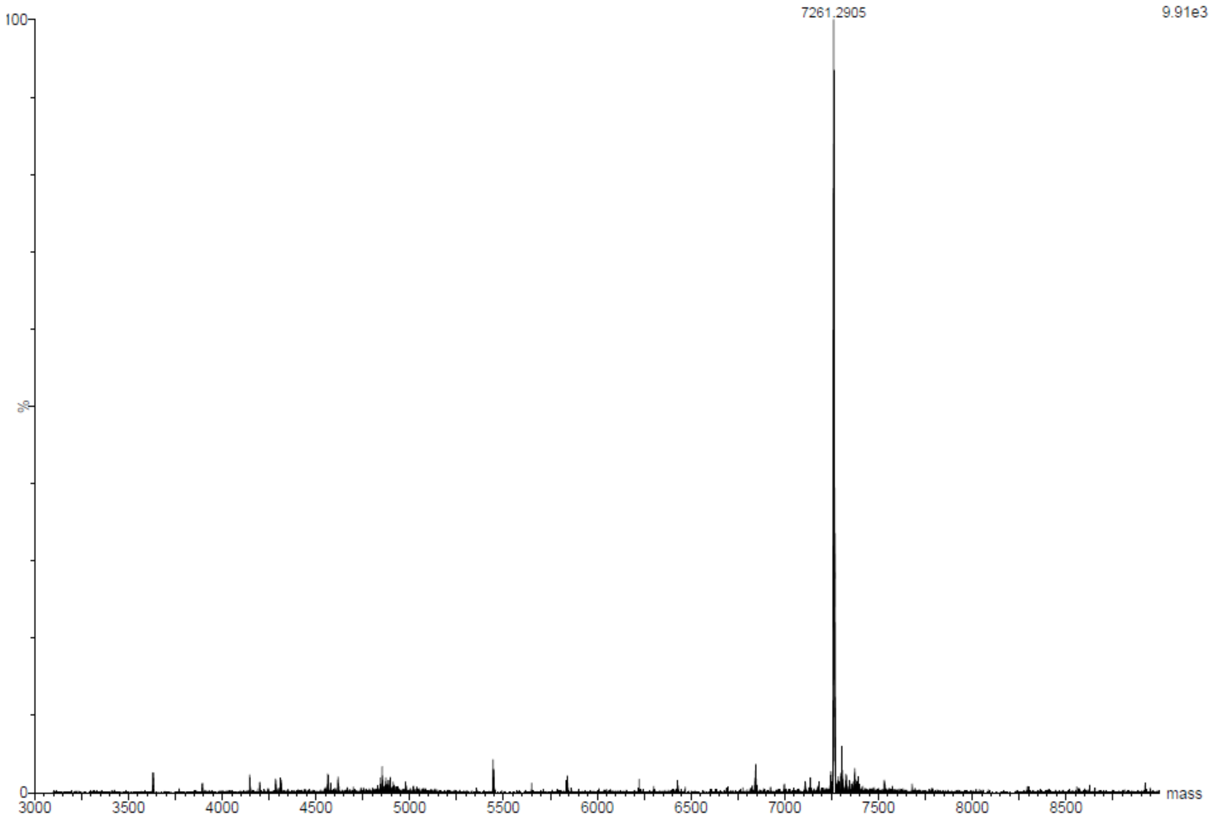

Figure S20: Mass spectrum (ES-) of **LNA7-OMe6.** Required **7262.46** Da, found **7261.29** Da. y-axis = relative intensity (%), x-axis = mass in Da.

**2´MOEPS+**	CCT CTT ACC TCA GTT ACA	

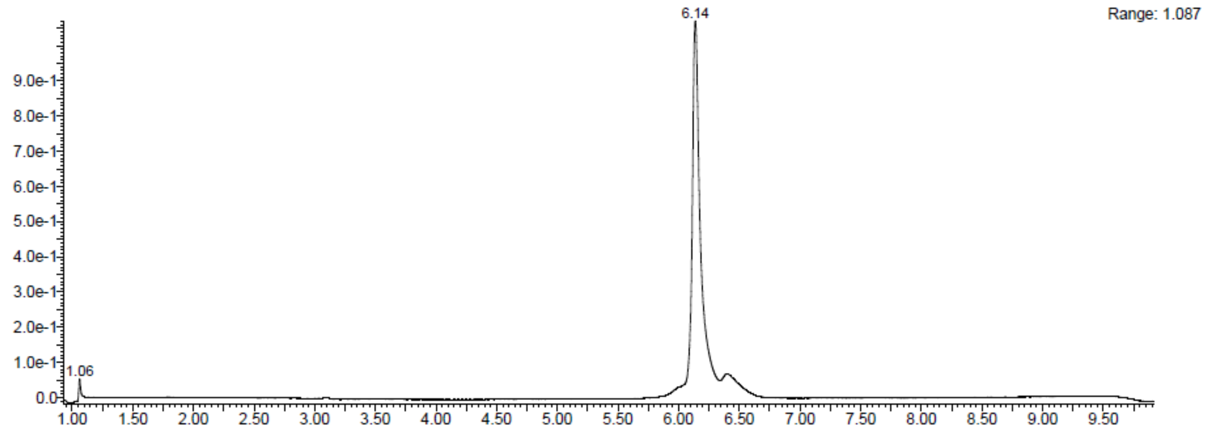

Figure S21: Reverse-phase UPLC of **2´MOEPS+** (UV absorbance at 260 nm vs time in min).

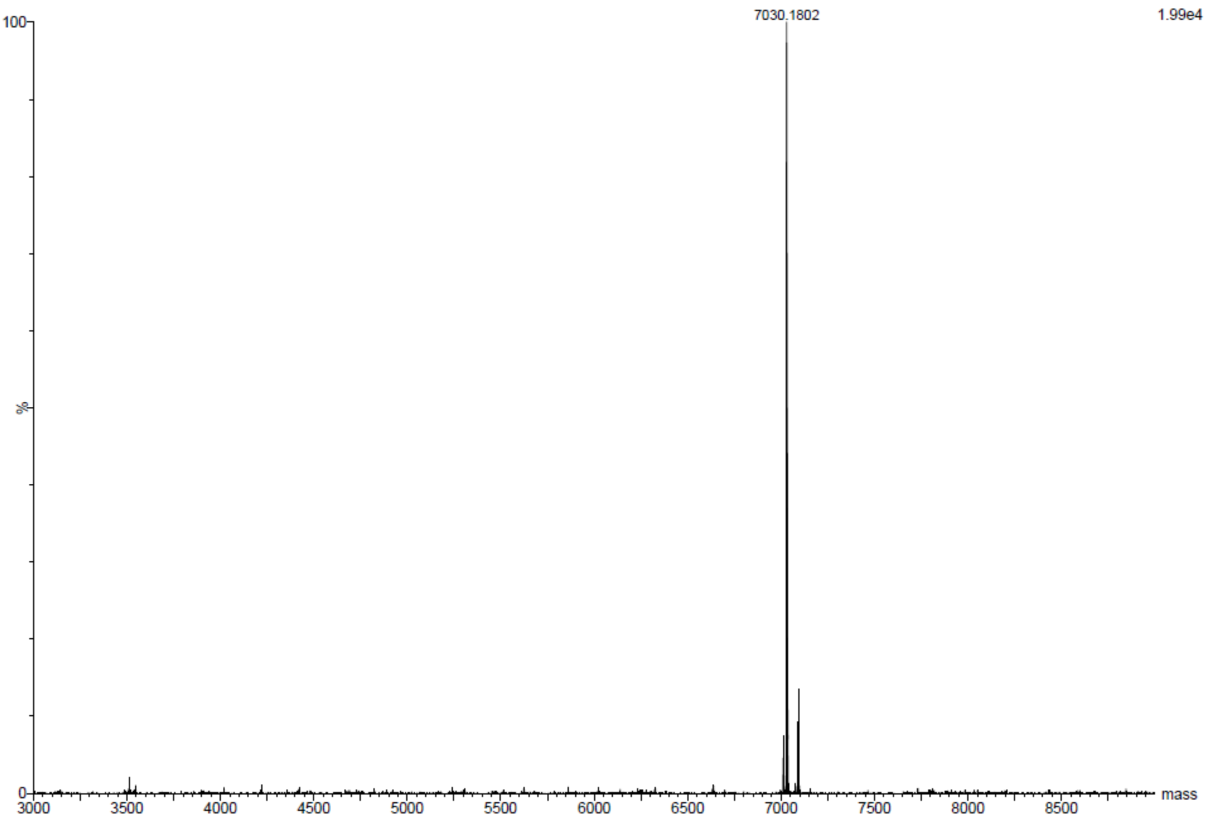

Figure S22: Mass spectrum (ES-) of **2´MOEPS+.** Required **7030.13** Da, found **7030.18** Da. y-axis = relative intensity (%), x-axis = mass in Da.

LNA2-MOE2	CCT CTT ACC TCA GTT ACA	

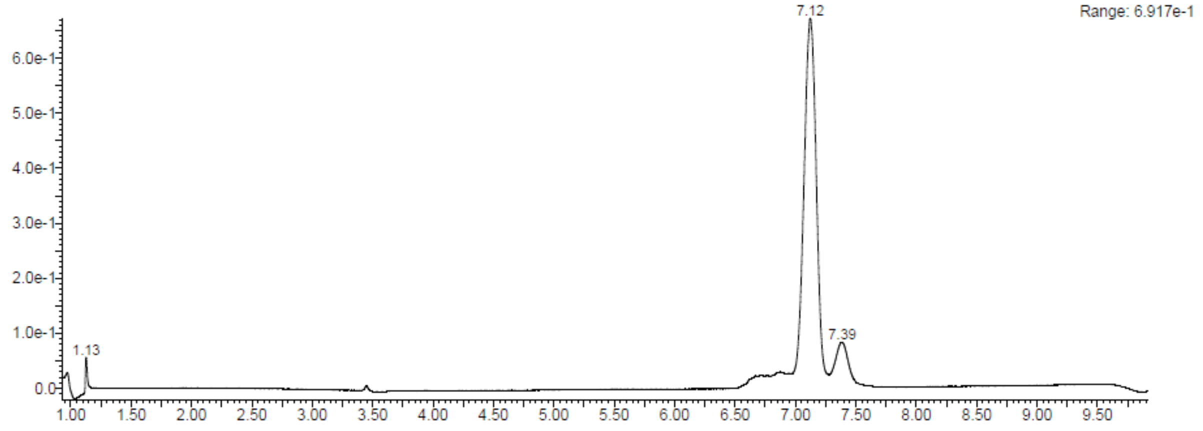

Figure S23: Reverse-phase UPLC of **LNA2-MOE2** (UV absorbance at 260 nm vs time in min).

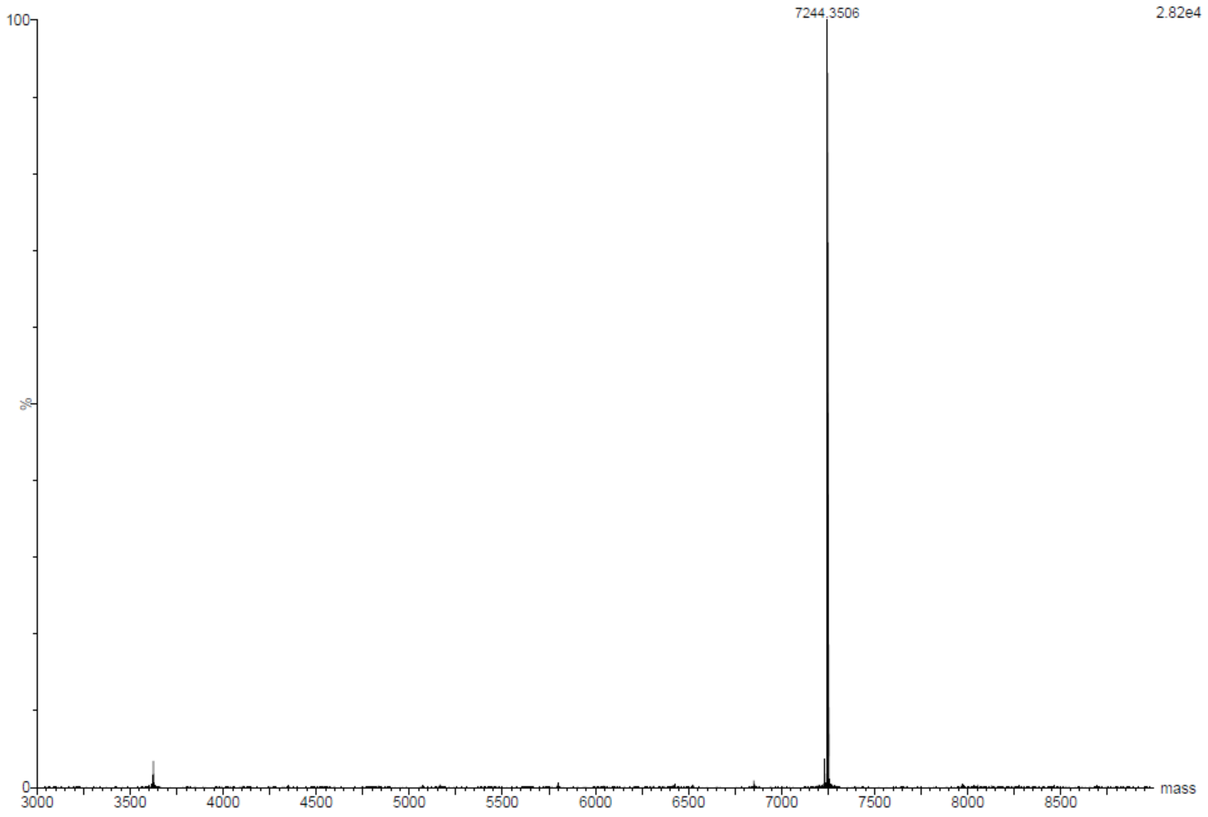

Figure S24: Mass spectrum (ES-) of **LNA2-MOE2.** Required **7246.41** Da, found **7244.35** Da. y-axis = relative intensity (%), x-axis = mass in Da.

**LNA4-MOE4**	CCT CTT ACC TCA GTT ACA	

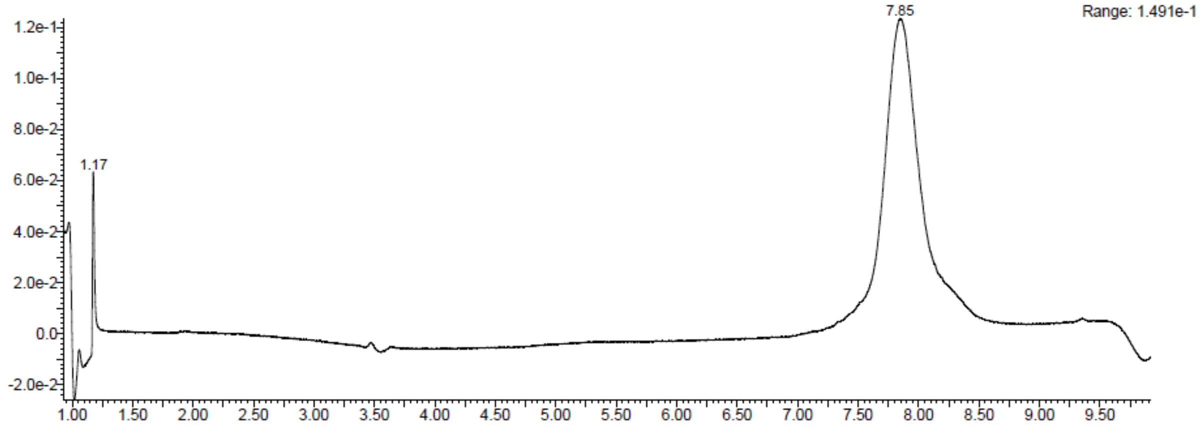

Figure S25: Reverse-phase UPLC of **LNA4-MOE4** (UV absorbance at 260 nm vs time in min).

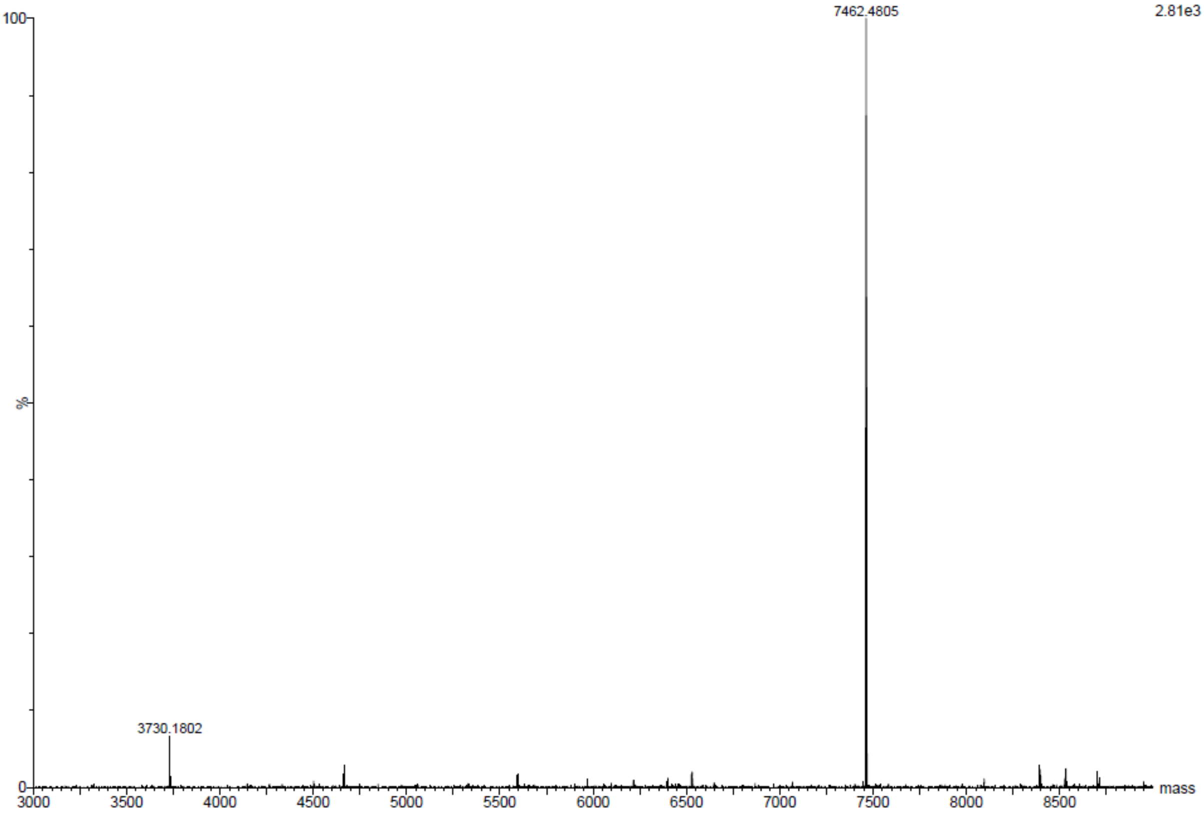

Figure S26: Mass spectrum (ES-) of **LNA4-MOE4.** Required **7462.69** Da, found **7462.48** Da. y-axis = relative intensity (%), x-axis = mass in Da.

LNA7-MOE6	CCT CTT ACC TCA GTT ACA	

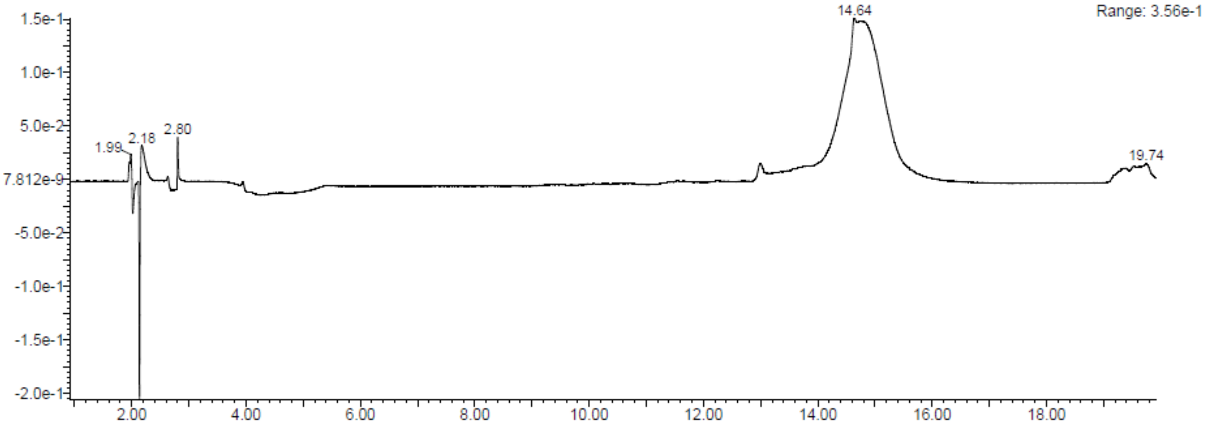

Figure S27: Reverse-phase UPLC of **LNA$_7$-MOE$_6$** (UV absorbance at 260 nm vs time in min).

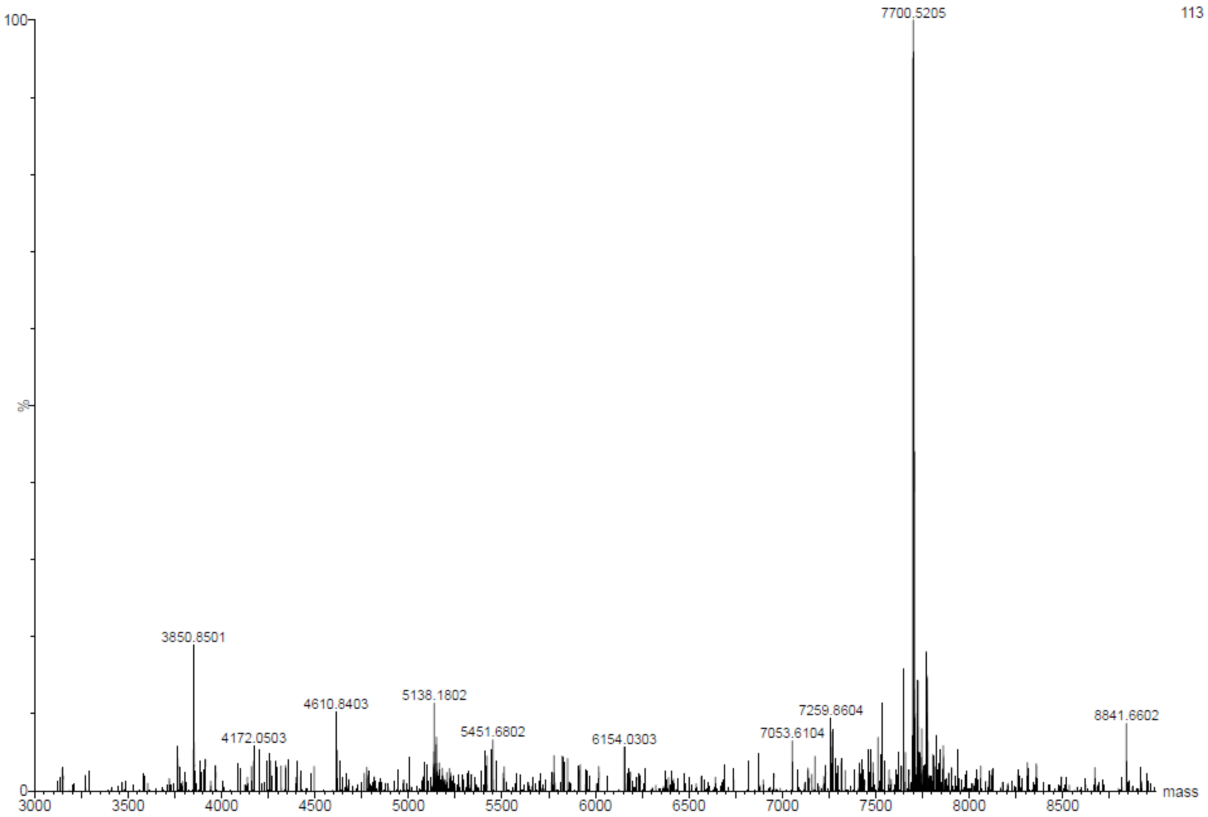

Figure S28: Mass spectrum (ES-) of **LNA$_7$-MOE$_6$.** Required **7702.99** Da, found **7700.52** Da. y-axis = relative intensity (%), x-axis = mass in Da.

**LNA$_{11}$-OMe$_6$-PO**	$C_oC_oT_oC_oT_oT_oA_oC_oC_oT_oC_oA_oG_oT_oT_oA_oC_oA$	

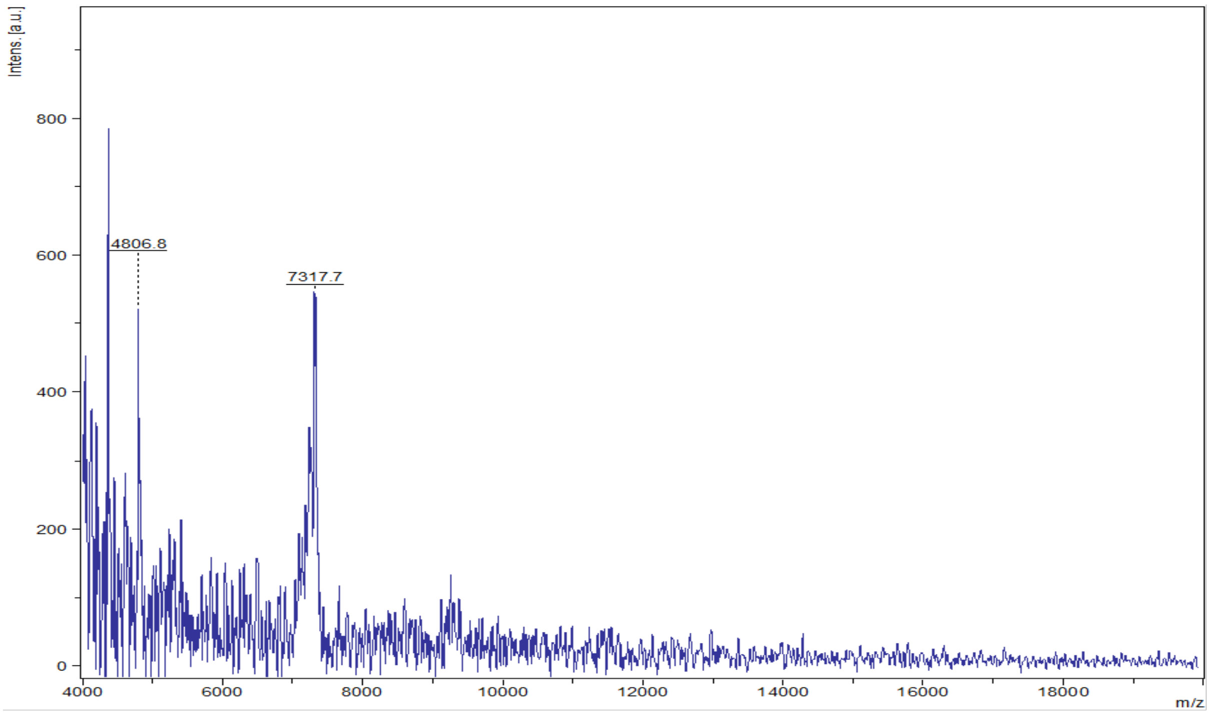

Figure S29: MALDI-MS of **LNA$_{11}$-OMe$_6$.** Required **7317.8** Da, found **7317.7** Da. y-axis = relative intensity (%), x-axis = mass in Da.

2.5.4 2´-F oligonucleotides

**F2**	CCU CU<u>T</u> ACC UCA GU<u>T</u> ACA	

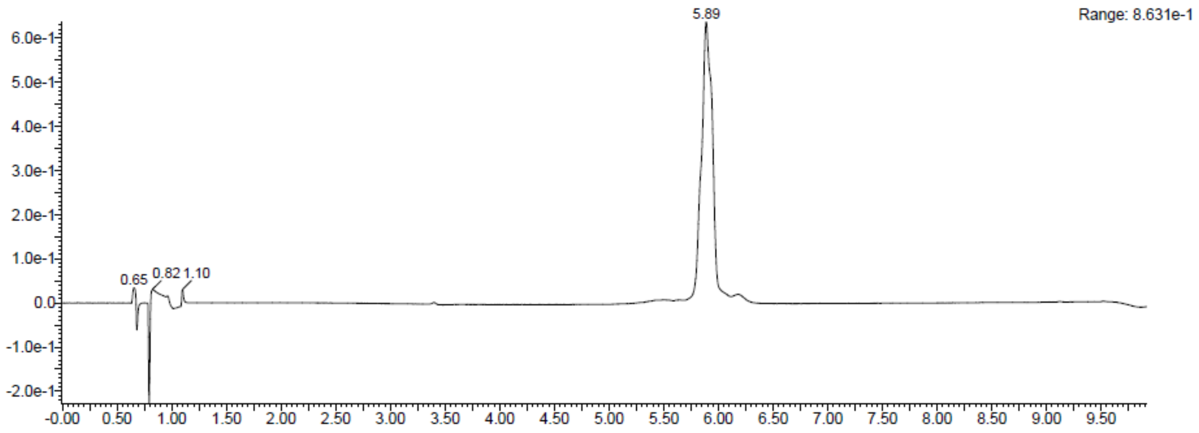

Figure S30: Reverse-phase UPLC of **F2** (UV absorbance at 260 nm vs time in min).

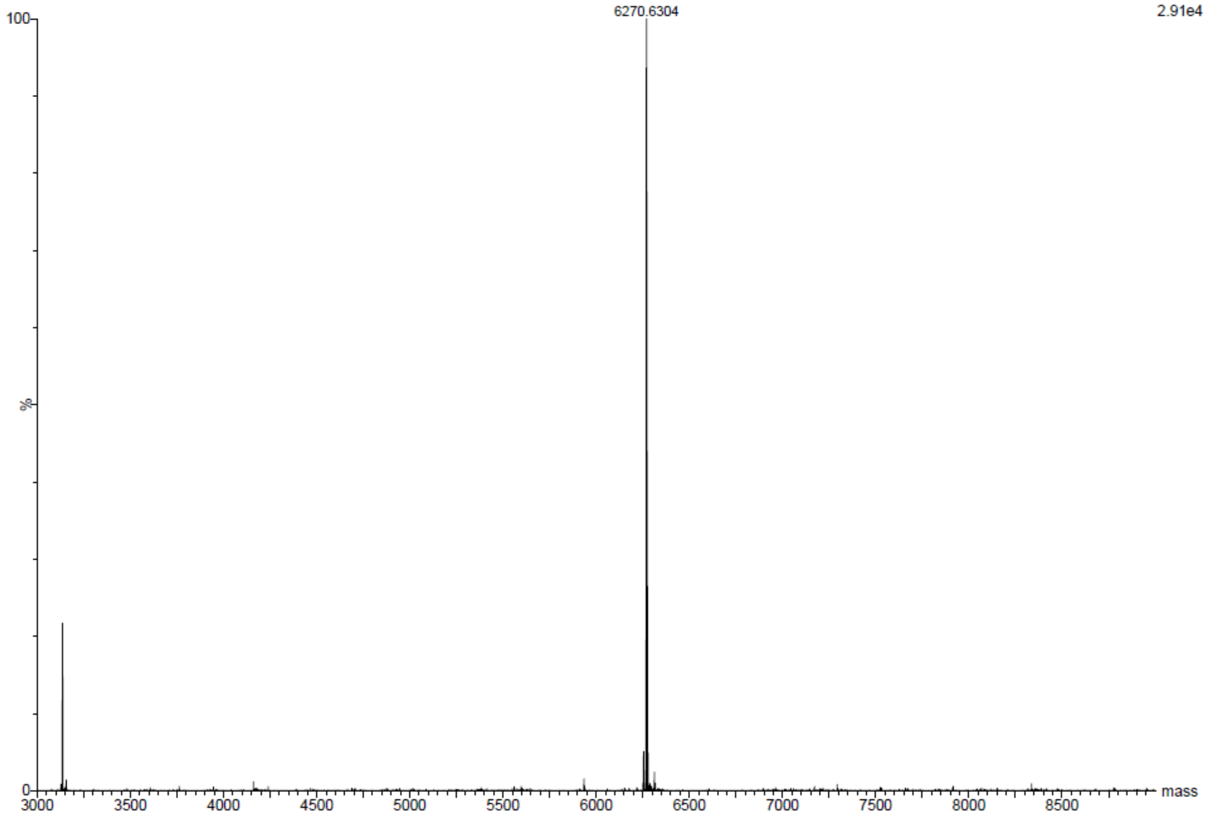

Figure S31: Mass spectrum (ES-) of **F2**. Required **6271.10** Da, found **6270.63** Da. y-axis = relative intensity (%), x-axis = mass in Da.

**F_4**	CCT CUT ACC TCA GUT ACA	

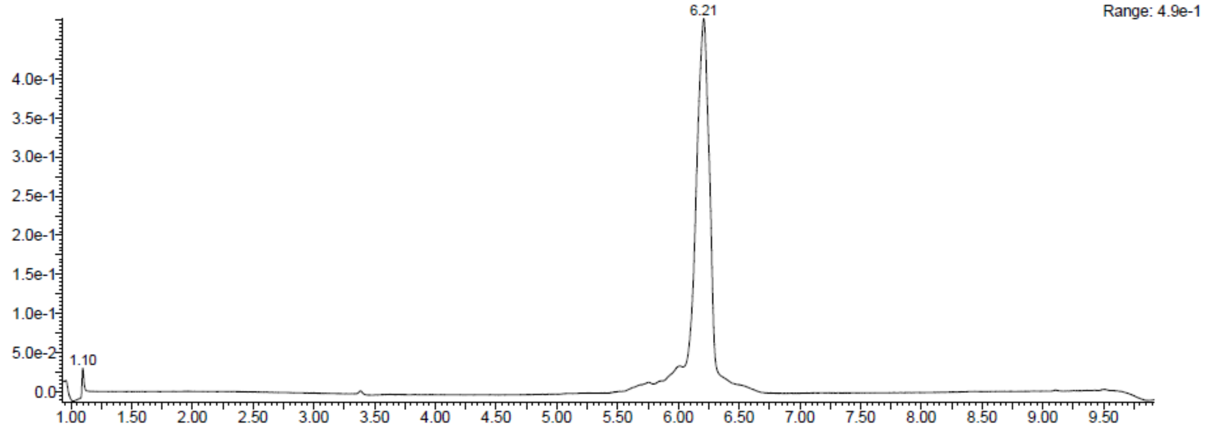

Figure S32: Reverse-phase UPLC of **F_4** (UV absorbance at 260 nm vs time in min).

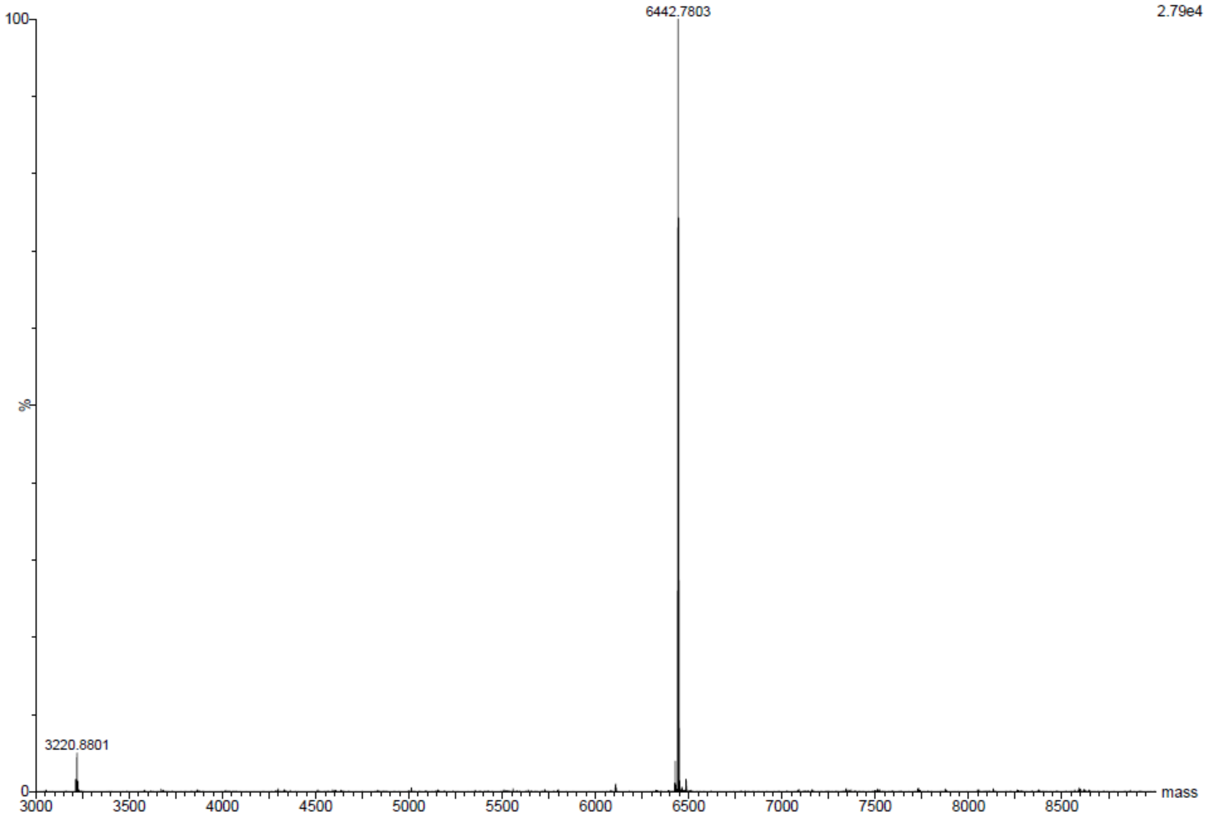

Figure S33: Mass spectrum (ES-) of **F_4**. Required **6443.31** Da, found **6442.78** Da. y-axis = relative intensity (%), x-axis = mass in Da.

**F_6**	CCT CTT ACC TCA GTT ACA	

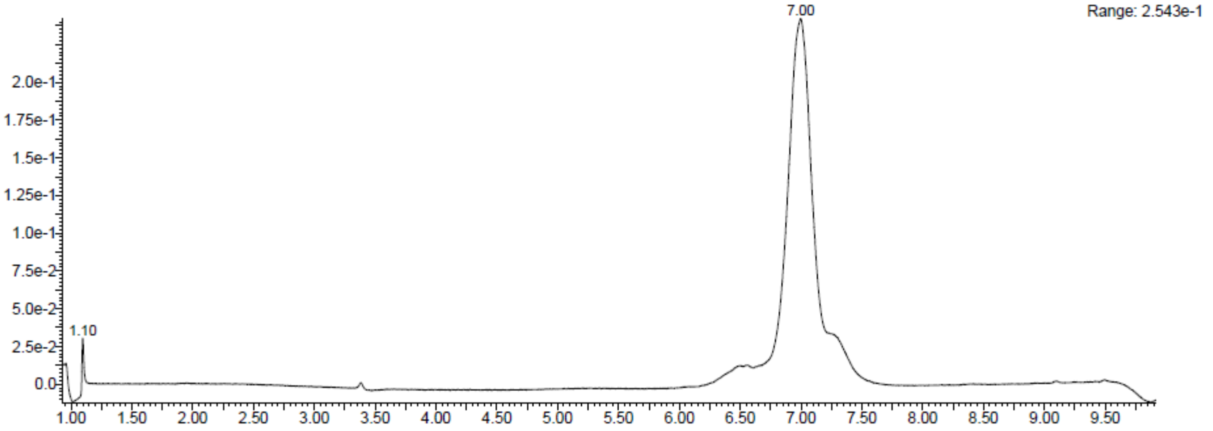

Figure S34: Reverse-phase UPLC of **F_6** (UV absorbance at 260 nm vs time in min).

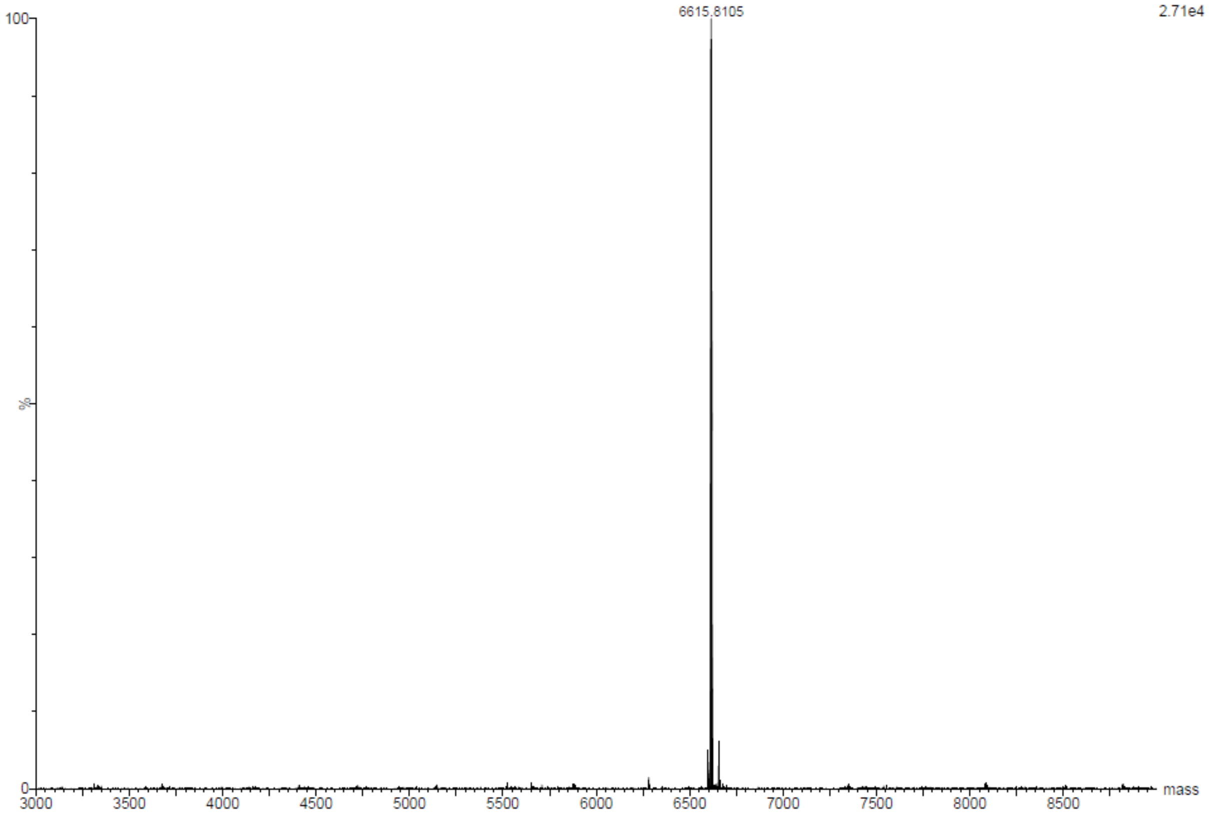

Figure S35: Mass spectrum (ES-) of **F_6**. Required **6615.53** Da, found **6615.81** Da. y-axis = relative intensity (%), x-axis = mass in Da.

**LNA2-F2**	CCT CUT ACC TCA GUT ACA	

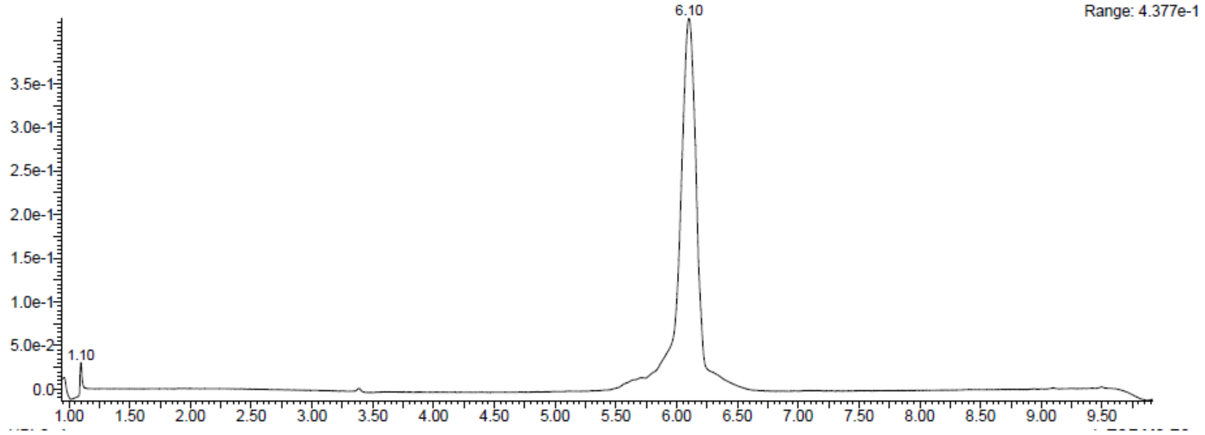

Figure S36: Reverse-phase UPLC of **LNA2-F2** (UV absorbance at 260 nm vs time in min).

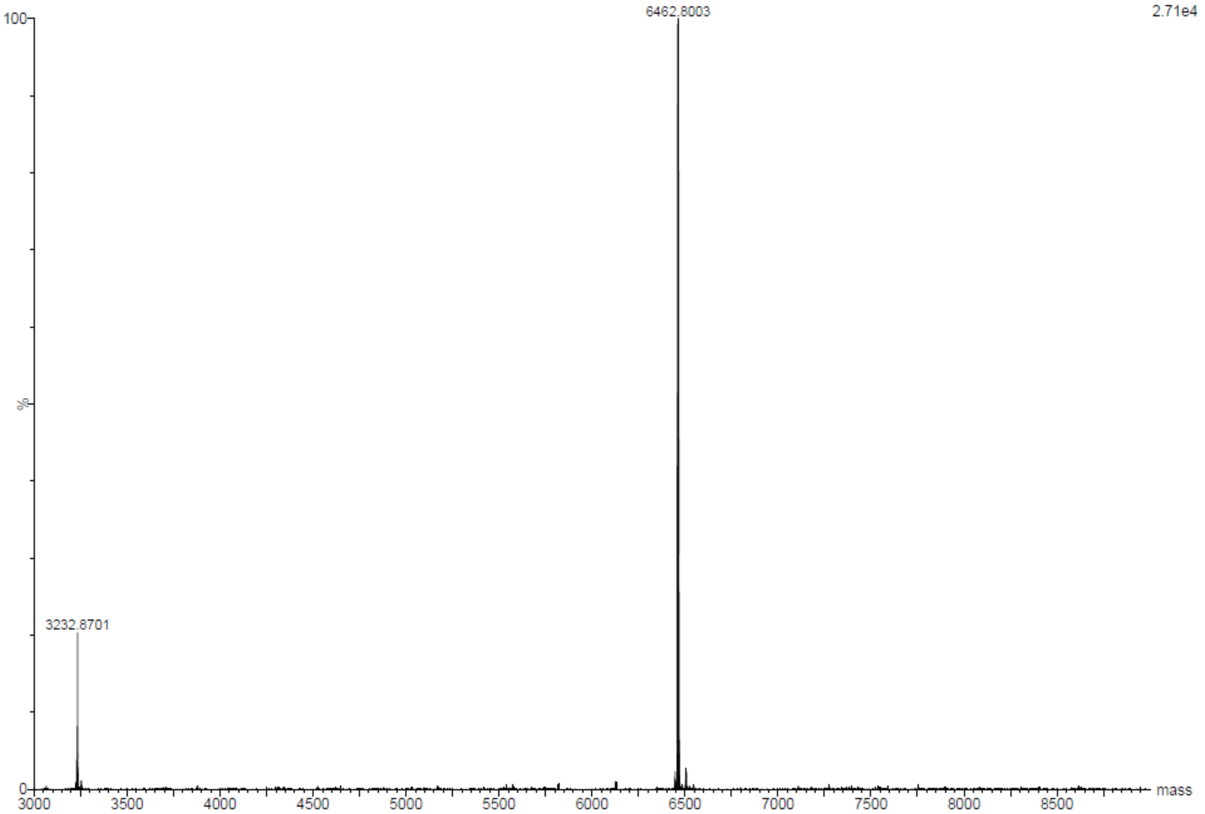

Figure S37: Mass spectrum (ES-) of **LNA2-F2**. Required **6463.35** Da, found **6462.80** Da. y-axis = relative intensity (%), x-axis = mass in Da.

**LNA2-F4**	CCT CTT ACC TCA GTT ACA	

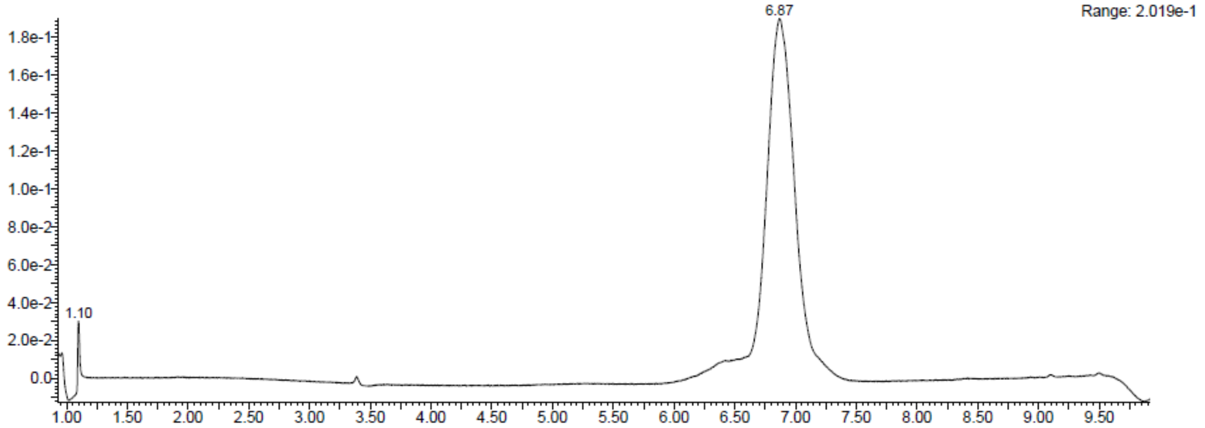

Figure S38: Reverse-phase UPLC of **LNA2-F4** (UV absorbance at 260 nm vs time in min).

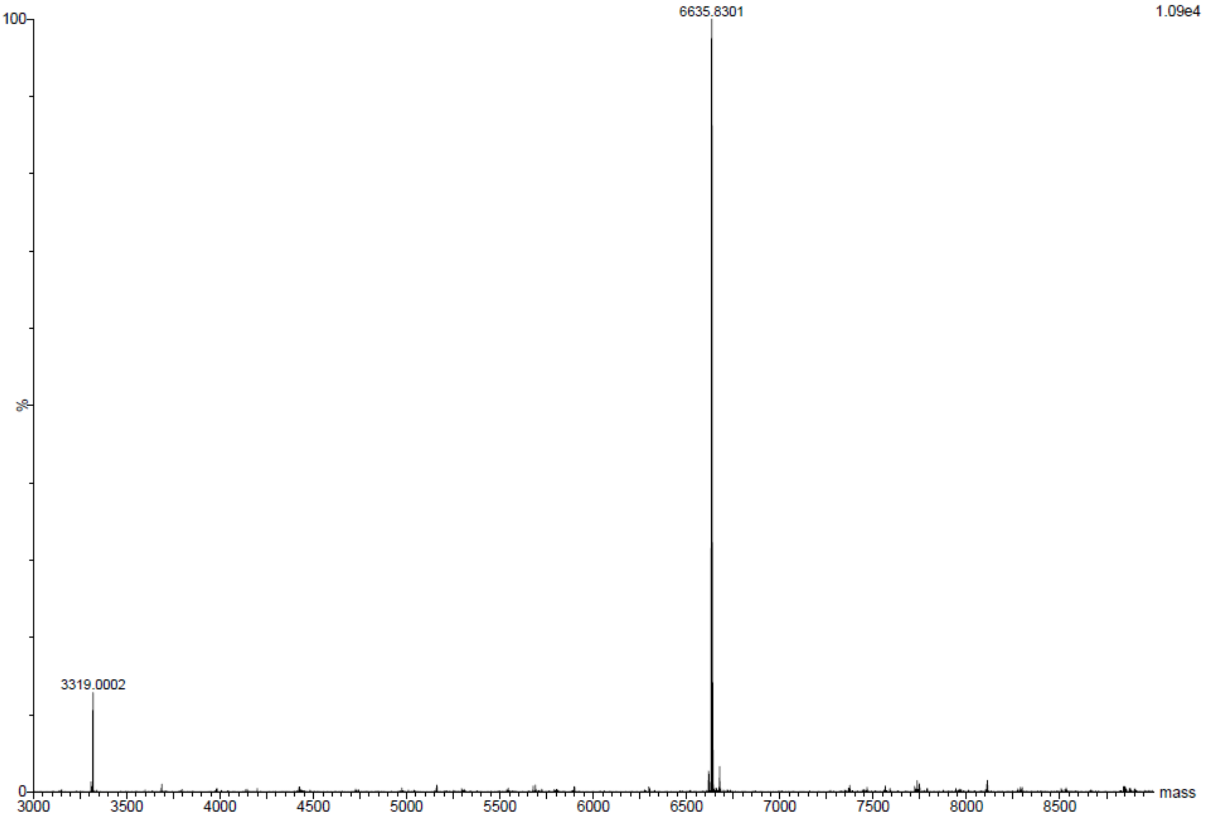

Figure S39: Mass spectrum (ES-) of **LNA2-F4**. Required **6635.57** Da, found **6635.83** Da. y-axis = relative intensity (%), x-axis = mass in Da.

LNA3-F3	CCT CTT ACC TCA GTT ACA	

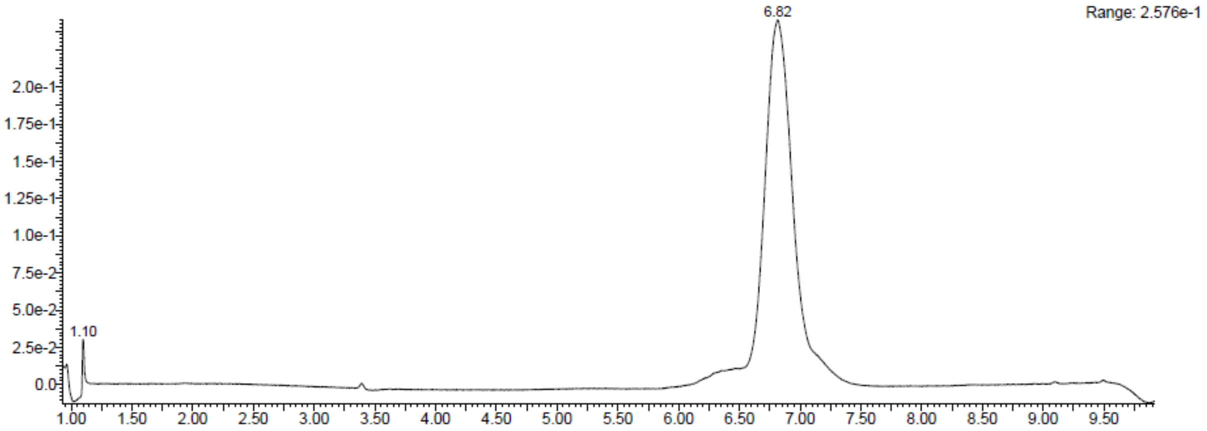

Figure S40: Reverse-phase UPLC of **LNA3-F3** (UV absorbance at 260 nm vs time in min).

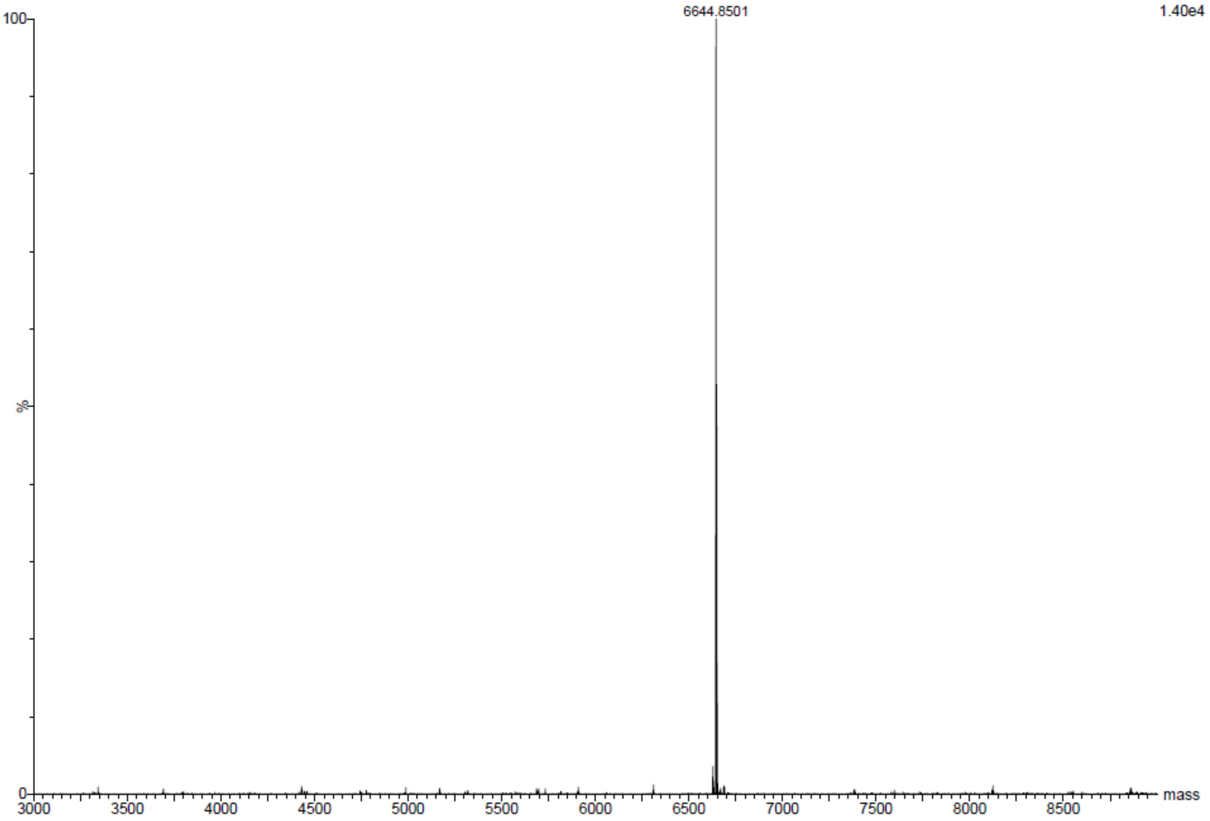

Figure S41: Mass spectrum (ES-) of **LNA3-F3**. Required **6645.59** Da, found **6644.85** Da. y-axis = relative intensity (%), x-axis = mass in Da.

**LNA$_4$-F$_2$**	CCT CTT ACC TCA GTT ACA	

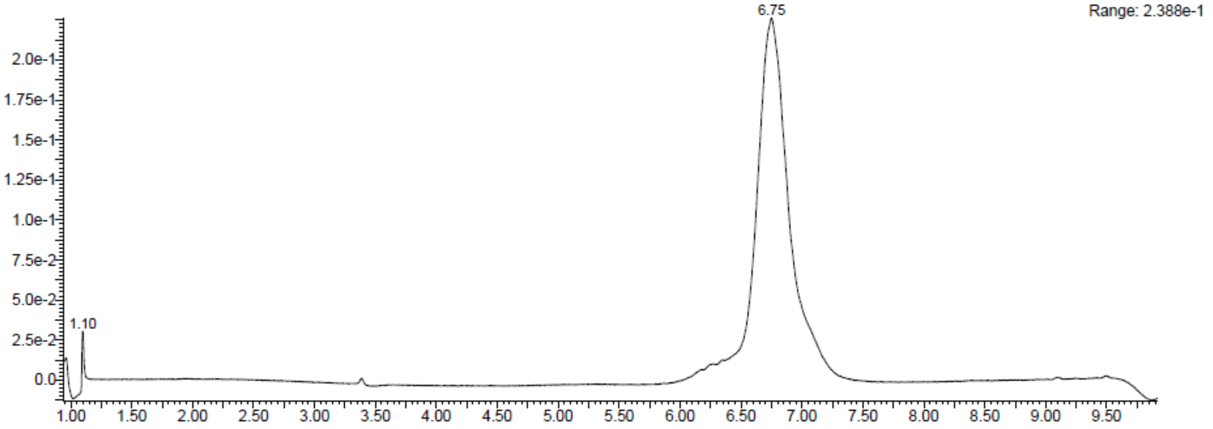

Figure S42: Reverse-phase UPLC of **LNA$_4$-F$_2$** (UV absorbance at 260 nm vs time in min).

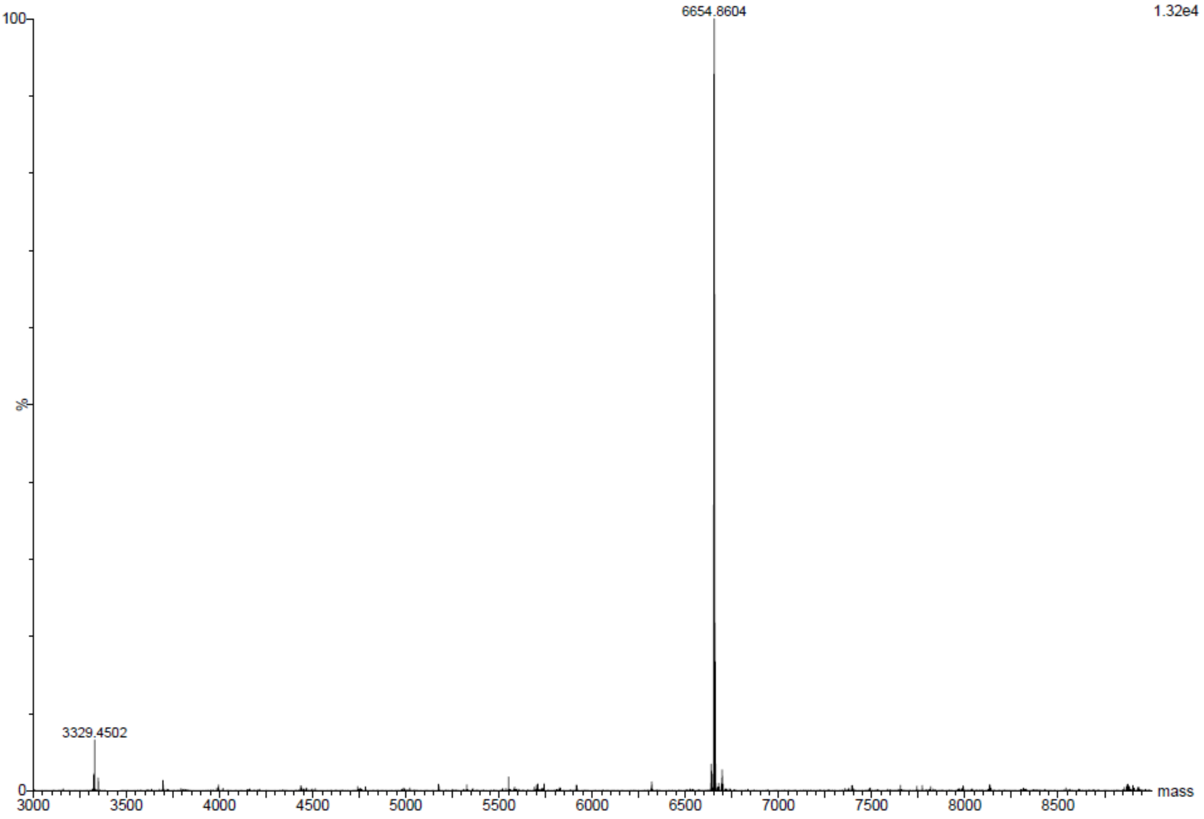

Figure S43: Mass spectrum (ES-) of **LNA$_4$-F$_2$**. Required **6655.61** Da, found **6654.86** Da. y-axis = relative intensity (%), x-axis = mass in Da.

2.5.4 Piperid-4-yl PTTE oligonucleotides

**Pip-LNA2**	CCU CUt ACC UCA GUt ACA	

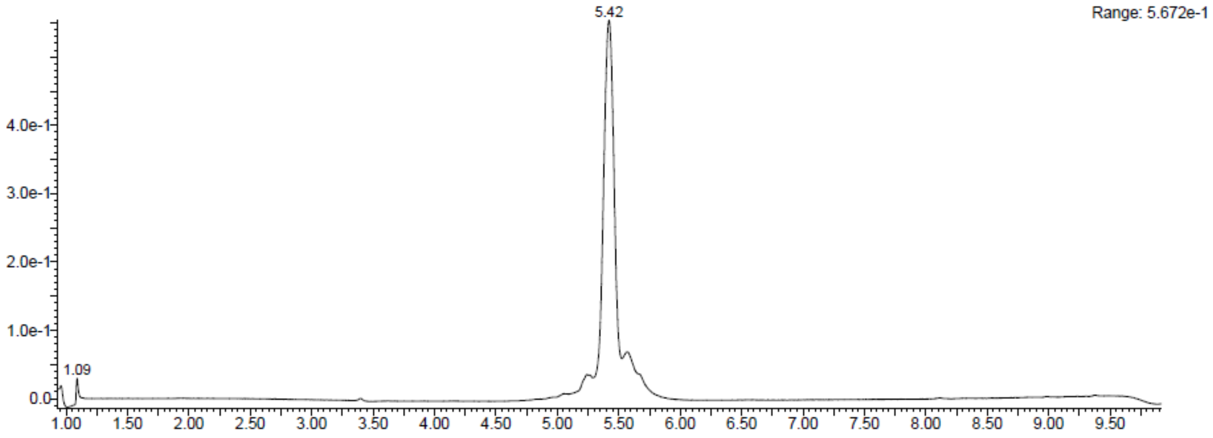

Figure S44: Reverse-phase UPLC of **Pip-LNA2** (UV absorbance at 260 nm vs time in min).

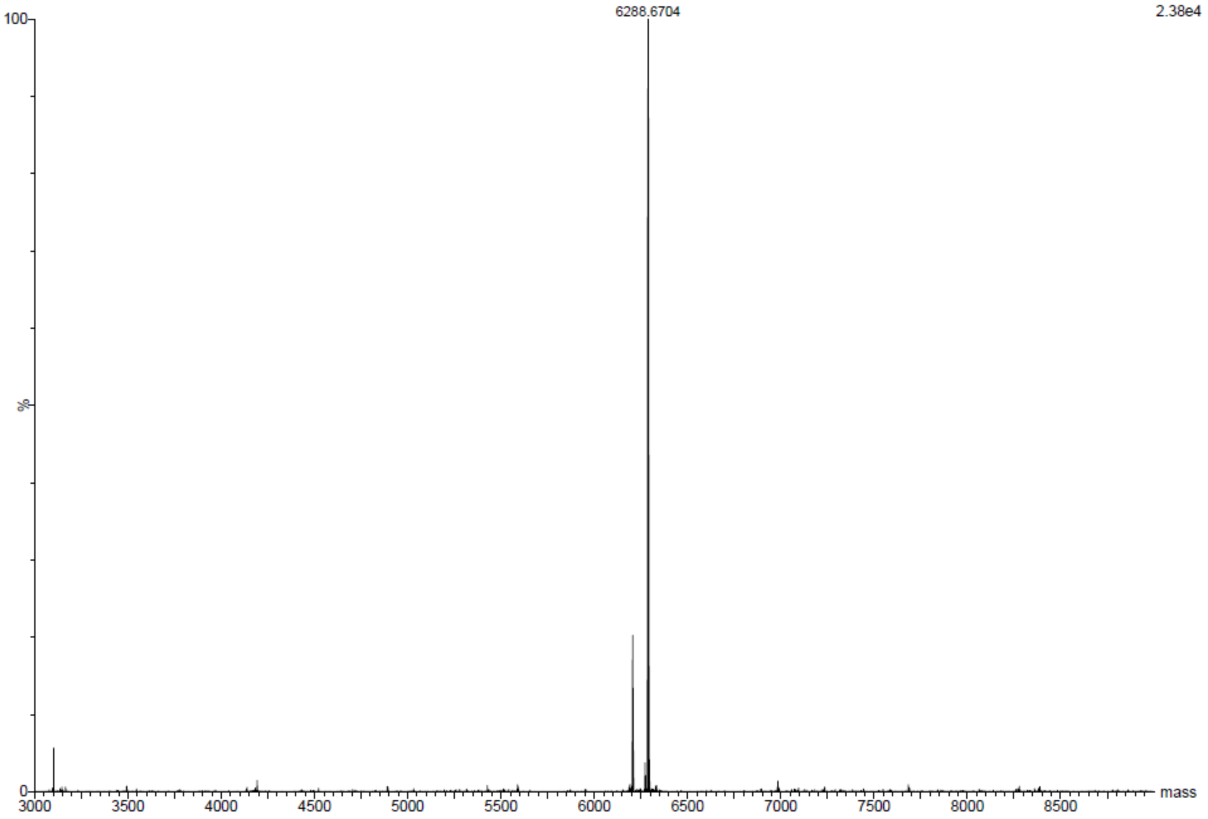

Figure S45: Mass spectrum (ES-) of **Pip-LNA2**. Required **6289.17** Da, found **6288.67** Da. y-axis = relative intensity (%), x-axis = mass in Da.

Pip-LNA4	CCt CUt ACC tCA GUt ACA	

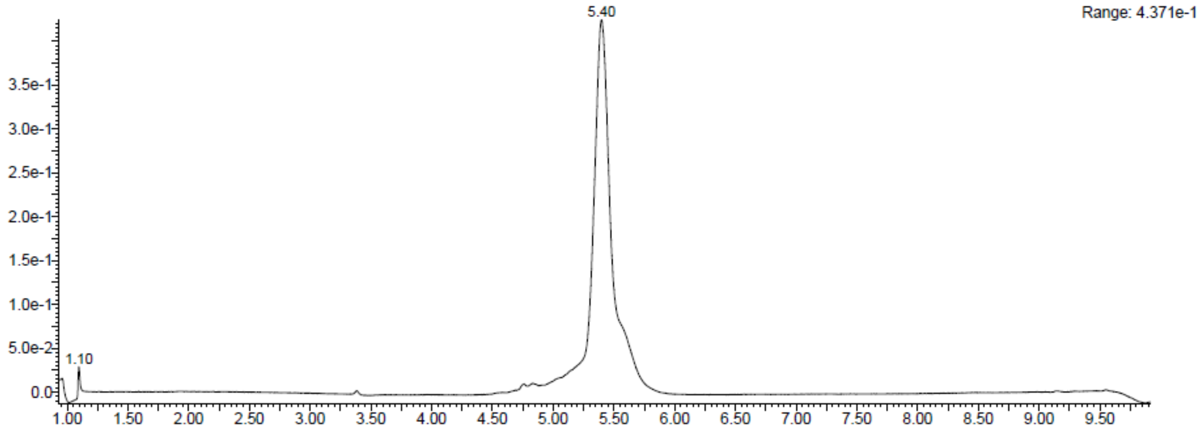

Figure S46: Reverse-phase UPLC of **Pip-LNA4** (UV absorbance at 260 nm vs time in min).

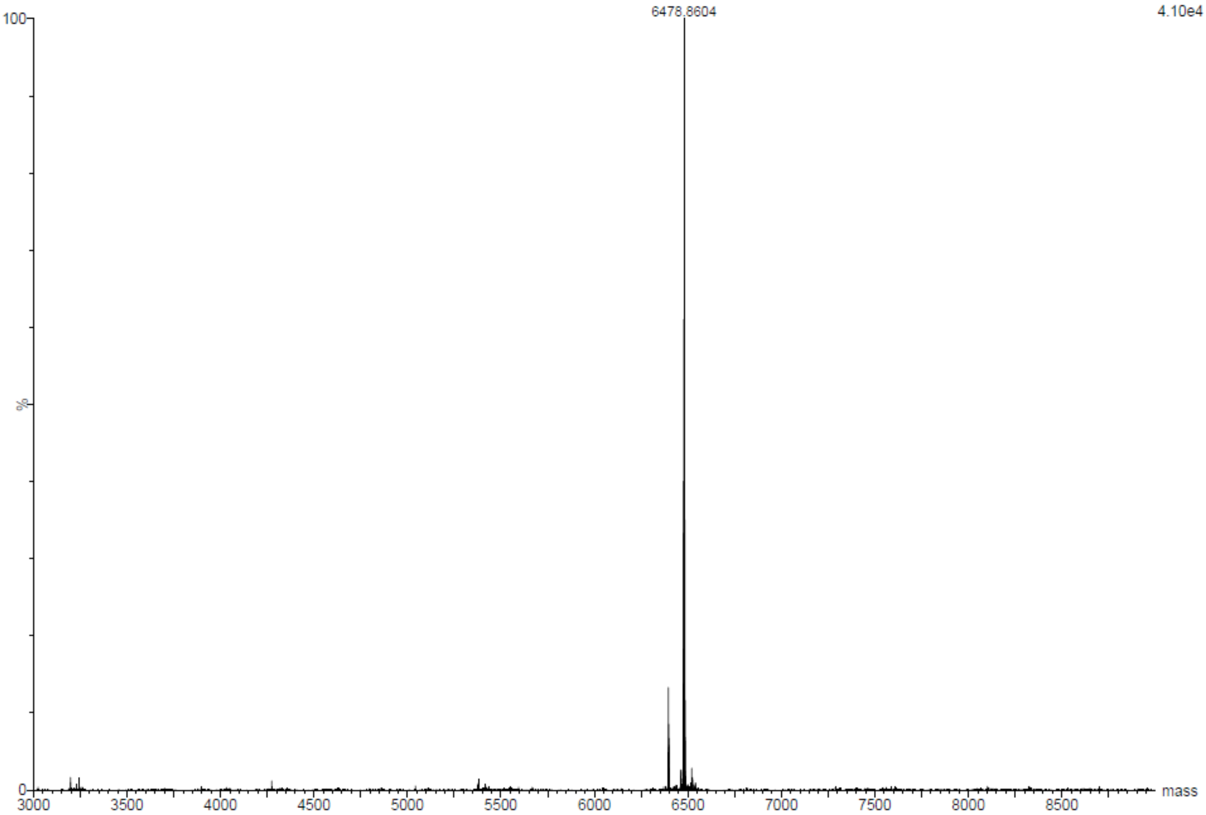

Figure S47: Mass spectrum (ES-) of **Pip-LNA4**. Required **6479.46** Da, found **6478.86** Da. y-axis = relative intensity (%), x-axis = mass in Da.

**Pip-LNA$_6$**	CCt Ctt ACC tCA Gtt ACA	

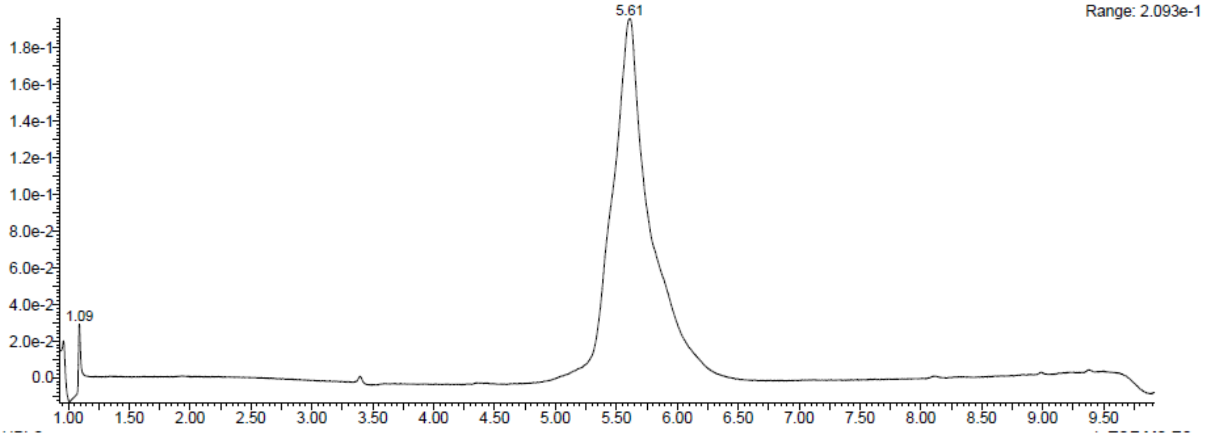

Figure S48: Reverse-phase UPLC of **Pip-LNA$_6$** (UV absorbance at 260 nm vs time in min).

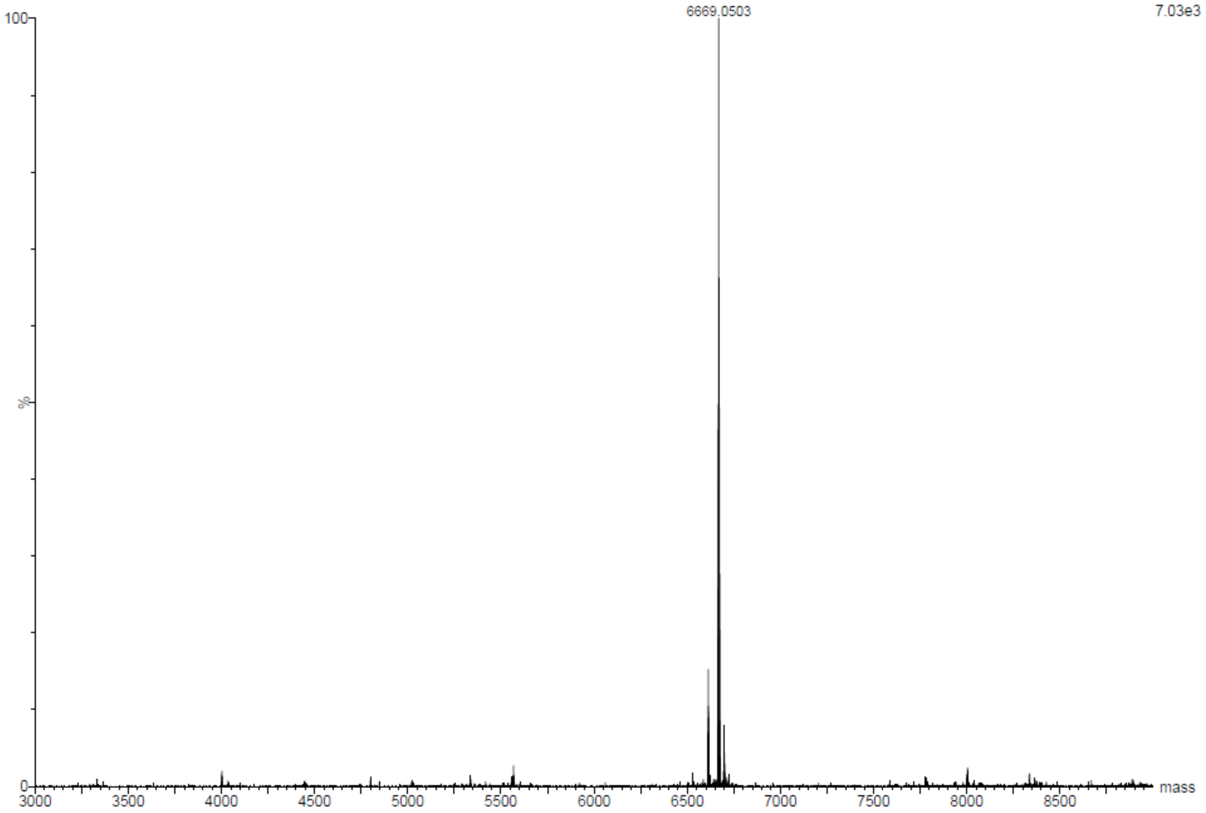

Figure S49: Mass spectrum (ES-) of **Pip-LNA$_6$**. Required **6669.75** Da, found **6669.05** Da. y-axis = relative intensity (%), x-axis = mass in Da.

**Pip-F2**	CCU CUt ACC UCA GUt ACA	

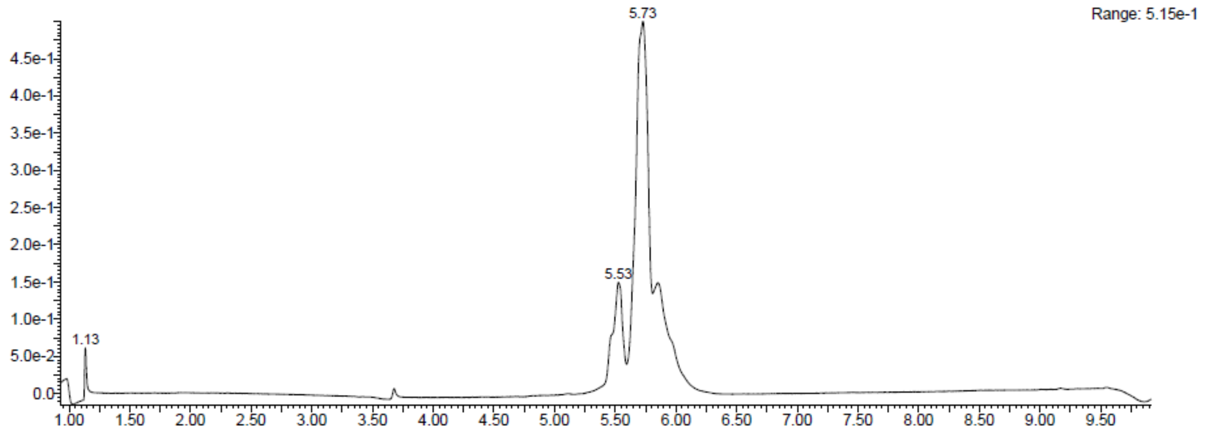

Figure S50: Reverse-phase UPLC of **Pip-F$_2$** (UV absorbance at 260 nm vs time in min).

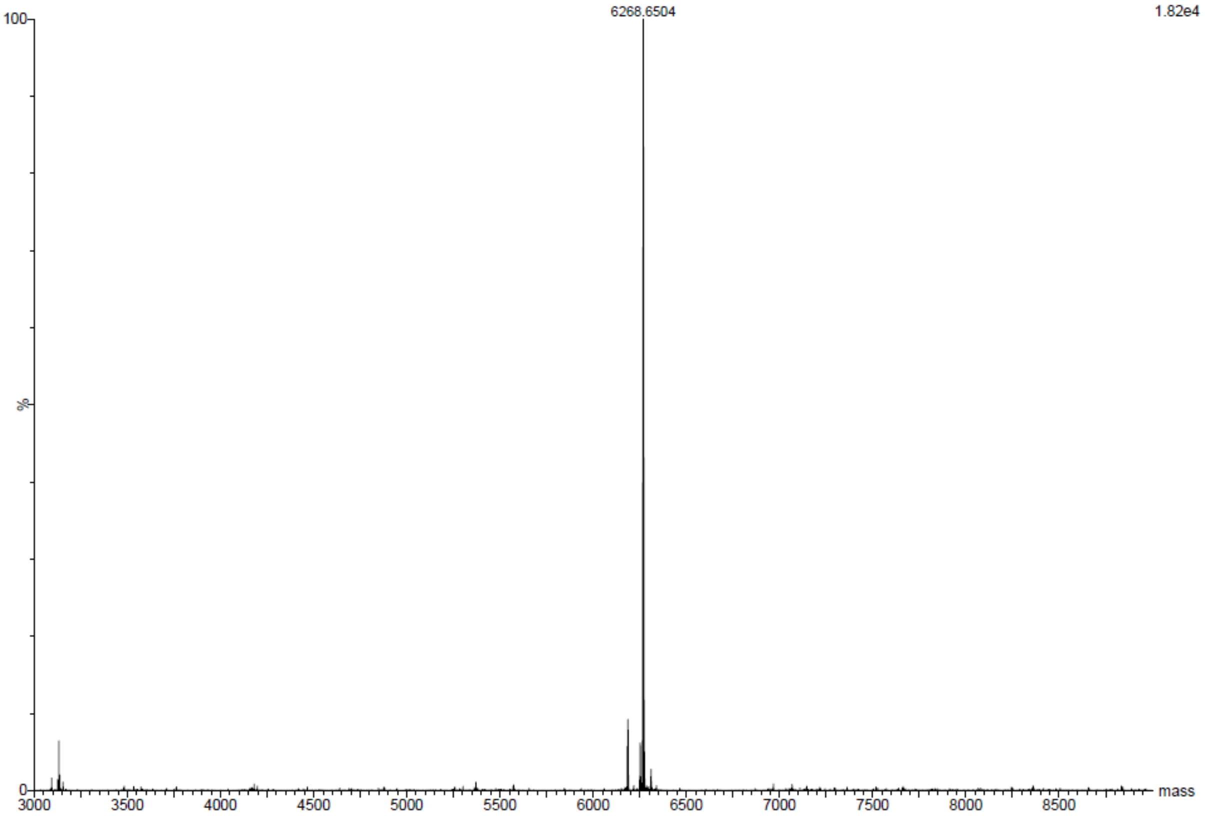

Figure S51: Mass spectrum (ES-) of **Pip-F$_2$**. Required **6269.13** Da, found **6268.65** Da. y-axis = relative intensity (%), x-axis = mass in Da.

**Pip-F4**	CCt CUt ACC tCA GUt ACA	

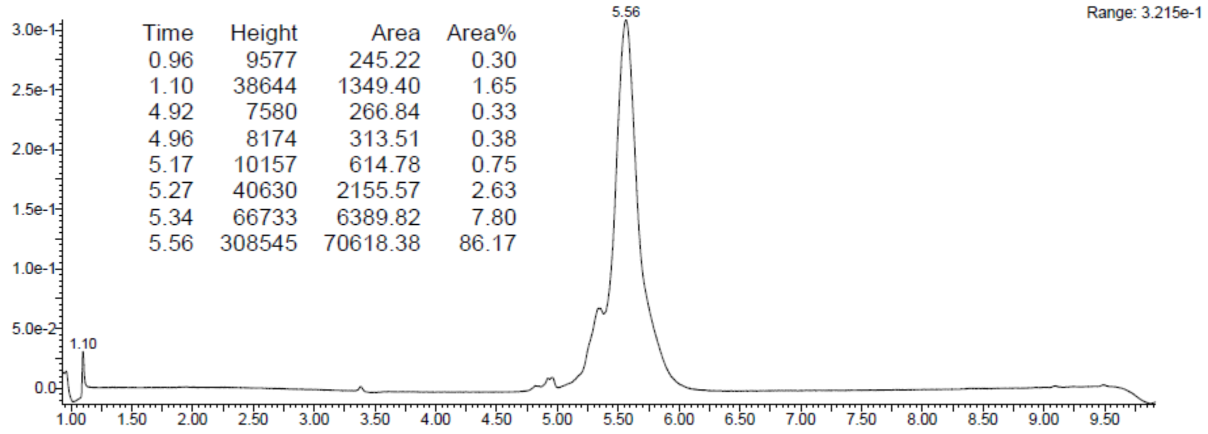

Figure S52: Reverse-phase UPLC of **Pip-F4** (UV absorbance at 260 nm vs time in min).

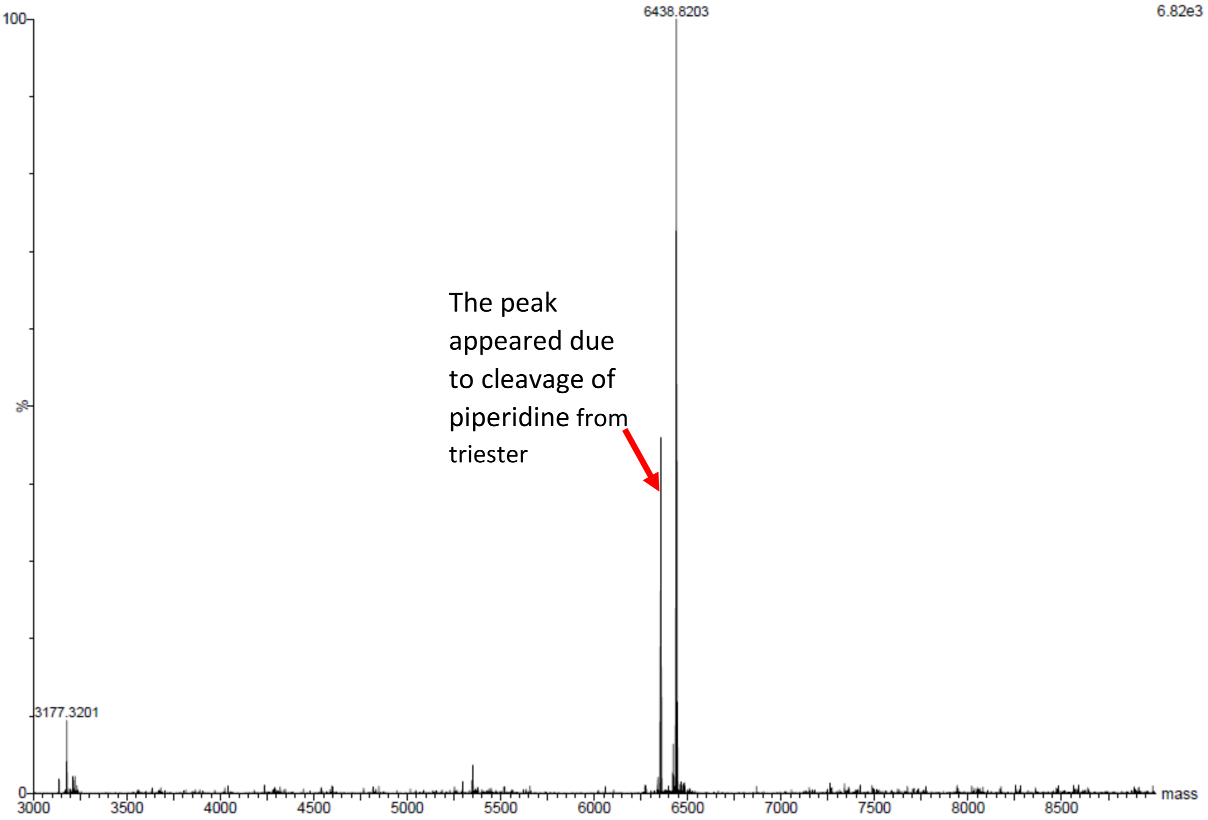

Figure S53: Mass spectrum (ES-) of **Pip-F4**. Required **6439.38** Da, found **6438.82** Da. y-axis = relative intensity (%), x-axis = mass in Da. The compound was used without further purification (~86% pure). The smaller peak corresponds to cleavage of one piperidine triester.

**Pip-F_6**	CCt Ctt ACC tCA Gtt ACA	

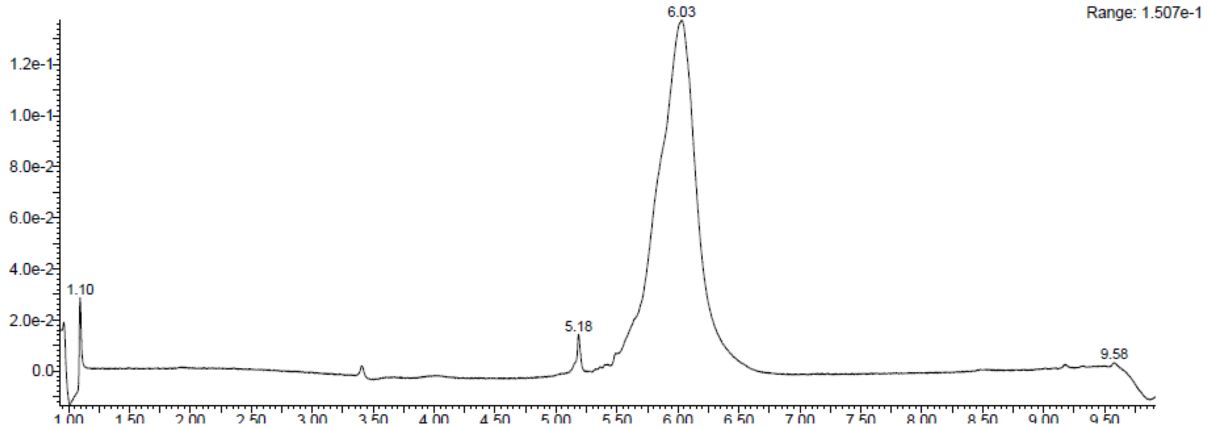

Figure S54: Reverse-phase UPLC of **Pip-F_6** (UV absorbance at 260 nm vs time in min).

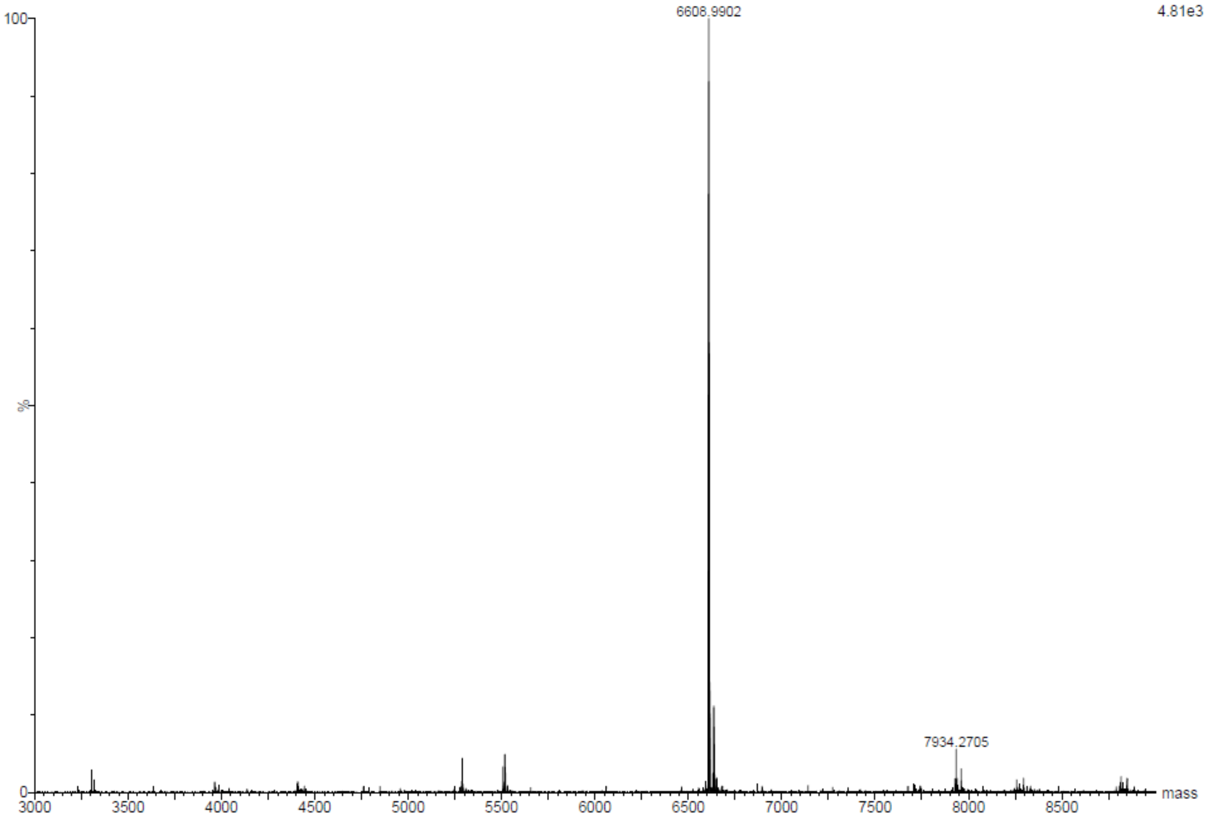

Figure S55: Mass spectrum (ES-) of **Pip-F_6**. Required **6609.63** Da, found **6608.99** Da. y-axis = relative intensity (%), x-axis = mass in Da.

2.5.4 Lipid conjugated oligonucleotides

**Pal-OMe4**	Pal-CC**T** CU**T** ACC **T**CA GU**T** ACA	

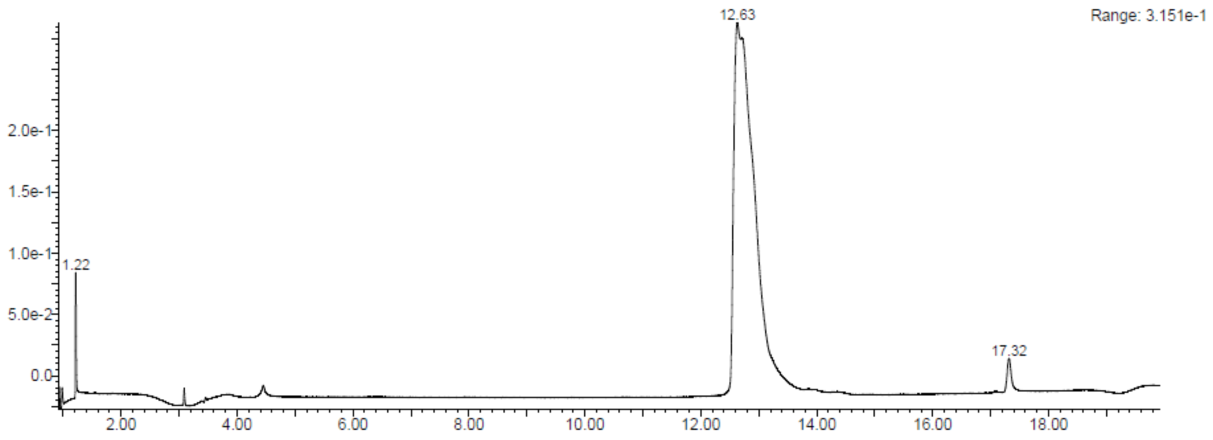

Figure S56: Reverse-phase UPLC of **Pal-OMe4** (UV absorbance at 260 nm vs time in min).

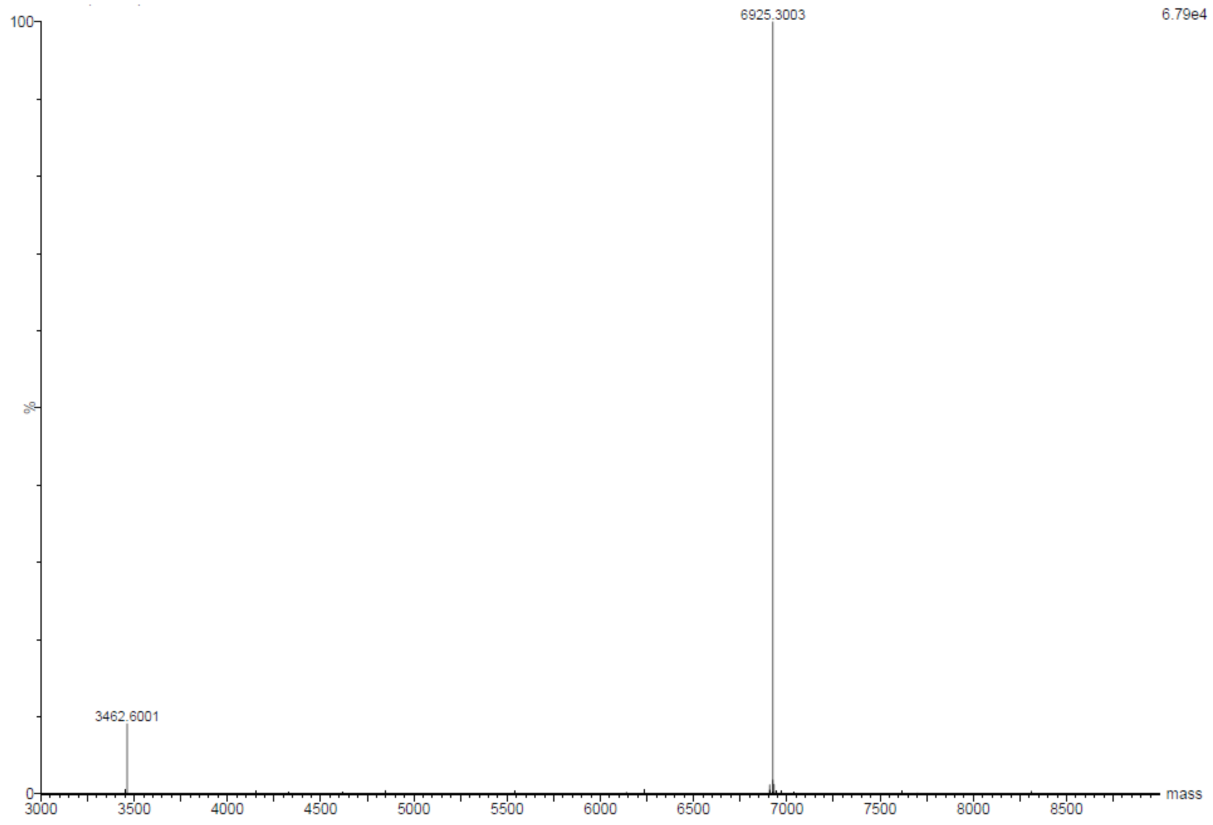

Figure S57: Mass spectrum (ES-) of **Pal-OMe4**. Required **6925.09** Da, found **6925.30** Da. y-axis = relative intensity (%), x-axis = mass in Da.

**Pal-MOE4**	Pal-CC<u>T</u> CU<u>T</u> ACC <u>T</u>CA GU<u>T</u> ACA	

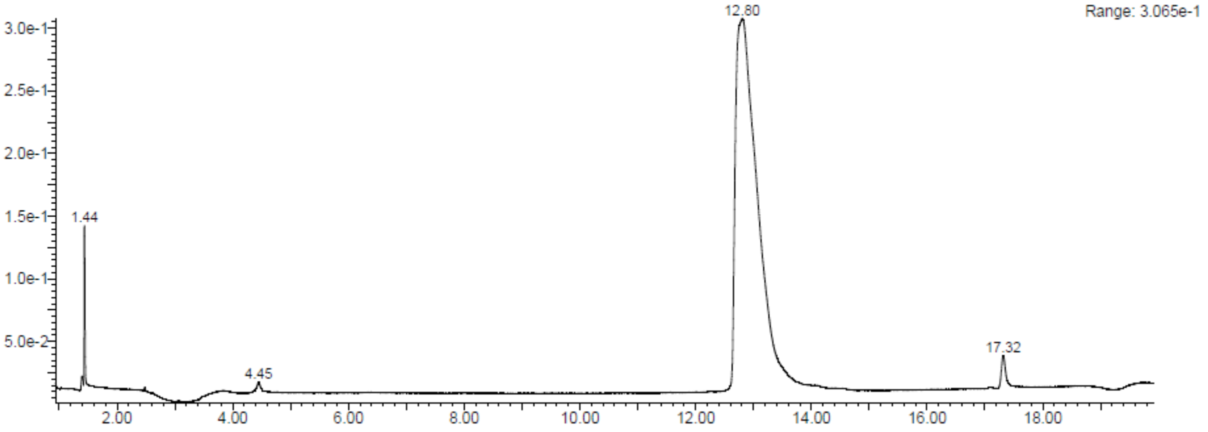

Figure S58: Reverse-phase UPLC of **Pal-MOE4** (UV absorbance at 260 nm vs time in min).

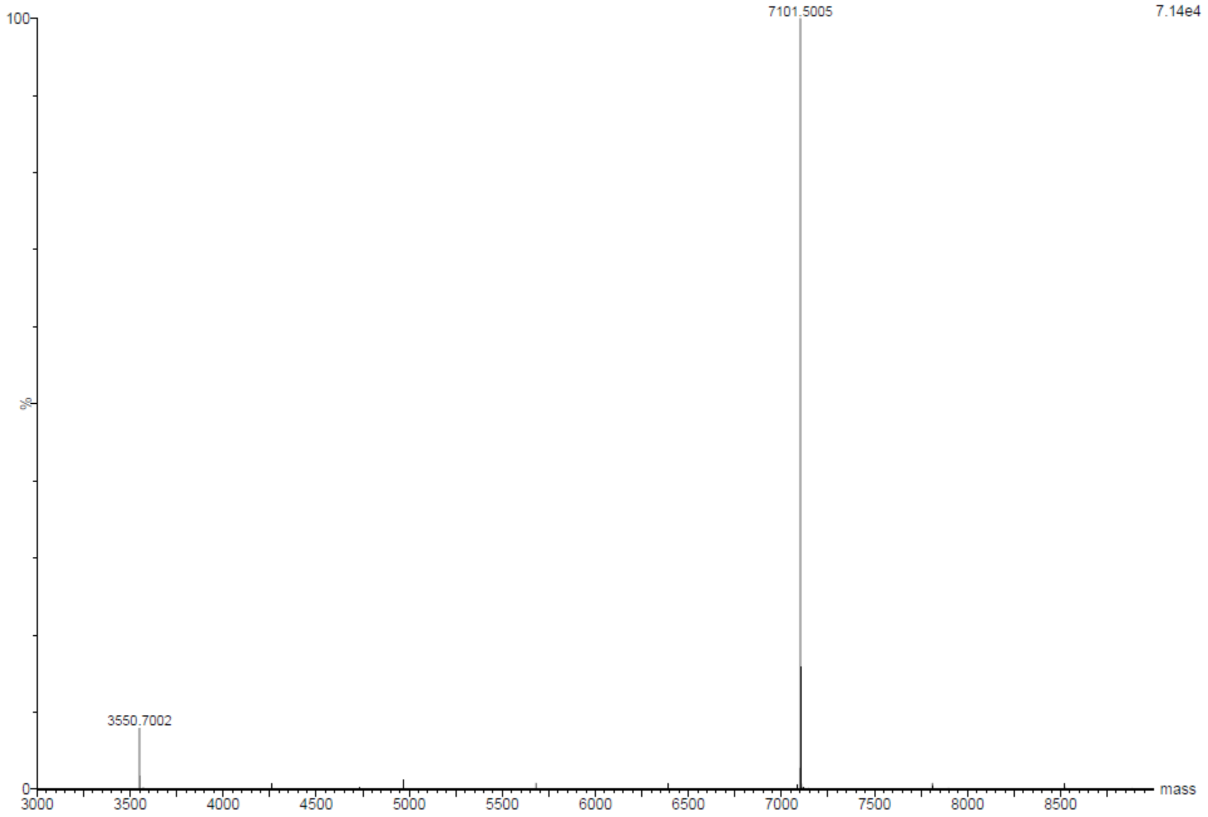

Figure S59: Mass spectrum (ES-) of **Pal-MOE4**. Required **7101.30** Da, found **7101.50** Da. y-axis = relative intensity (%), x-axis = mass in Da.

**Pal-LNA4**	Pal-CC**T** CU**T** ACC **T**CA GU**T** ACA	

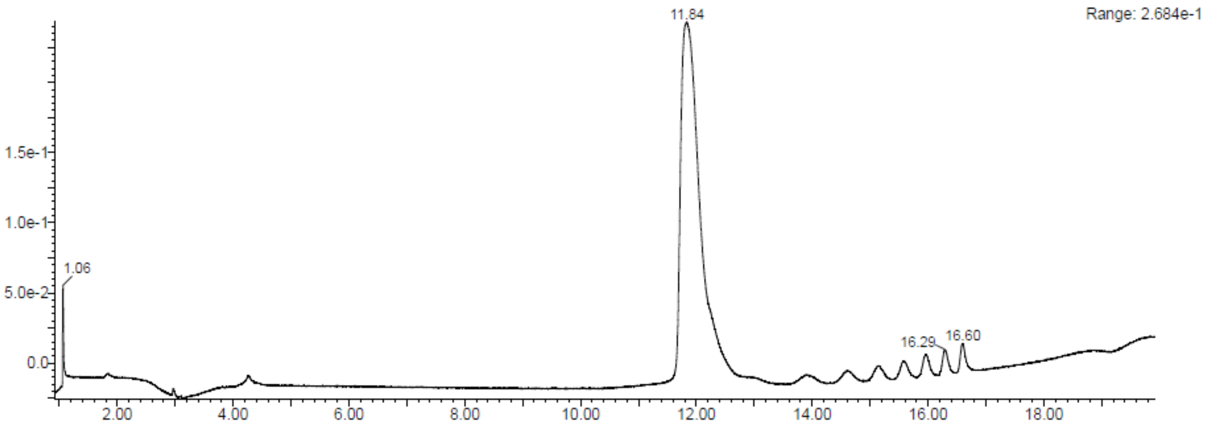

Figure S60: Reverse-phase UPLC of **Pal-LNA4** (UV absorbance at 260 nm vs time in min).

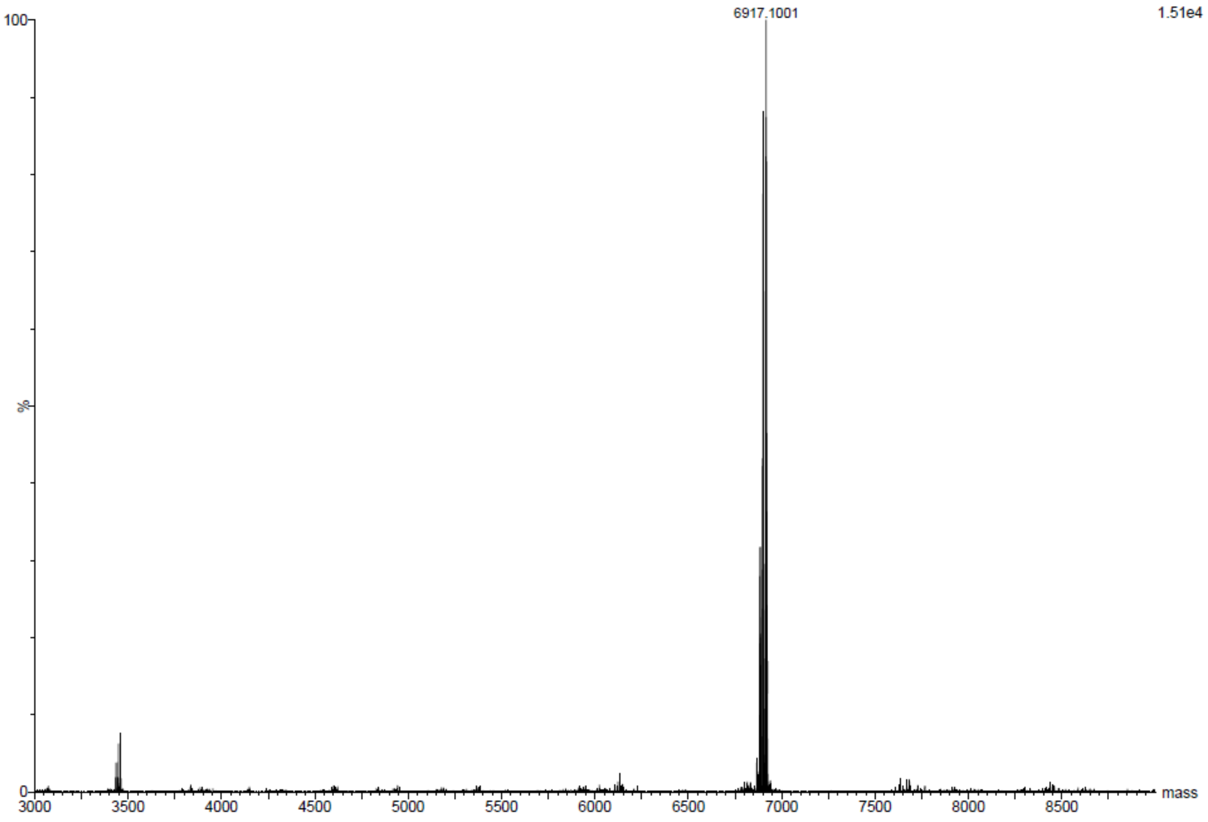

Figure S61: Mass spectrum (ES-) of **Pal-LNA4**. Required **6917.02** Da, found **6917.10** Da. y-axis = relative intensity (%), x-axis = mass in Da. The smaller peaks close to desired peak (M-16) are corresponding to the S→O exchange due to conversion of phosphorothioate (PS) to phosphodiester

**Pal-LNA4-PS**	Pal-CCT CUT ACC TCA GUT ACA	

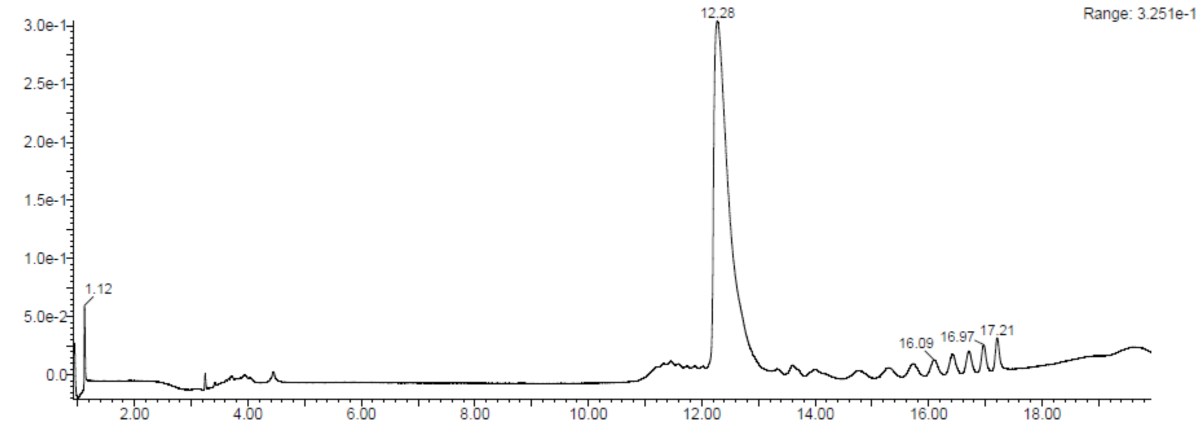

Figure S62: Reverse-phase UPLC of **Pal-LNA4-PS** (UV absorbance at 260 nm vs time in min). The small peaks are likely due to aggregation or micelle formation.

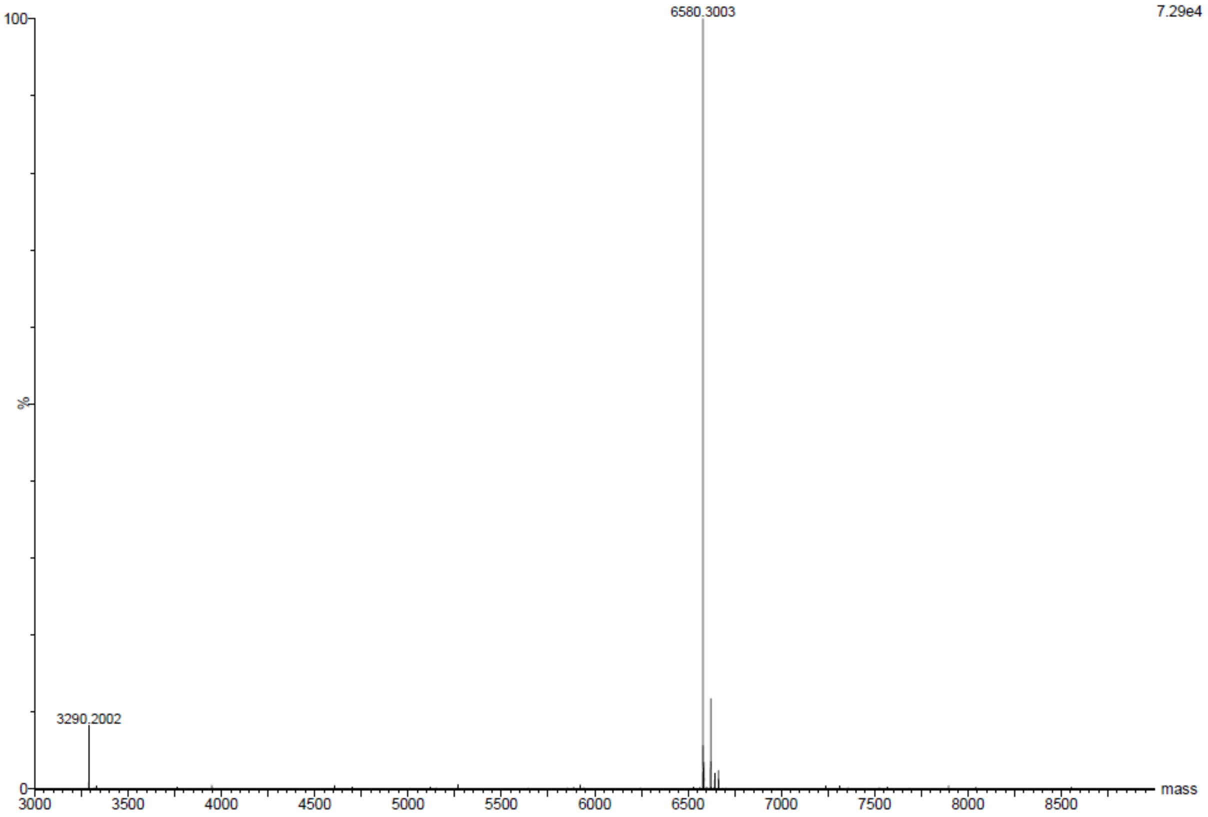

Figure S63: Mass spectrum (ES-) of **Pal-LNA4-PS**. Required **6580.55** Da, found **6580.30** Da. y-axis = relative intensity (%), x-axis = mass in Da.

Pal-Pip-LNA4	Pal-CCt CUt ACC tCA GUt ACA	

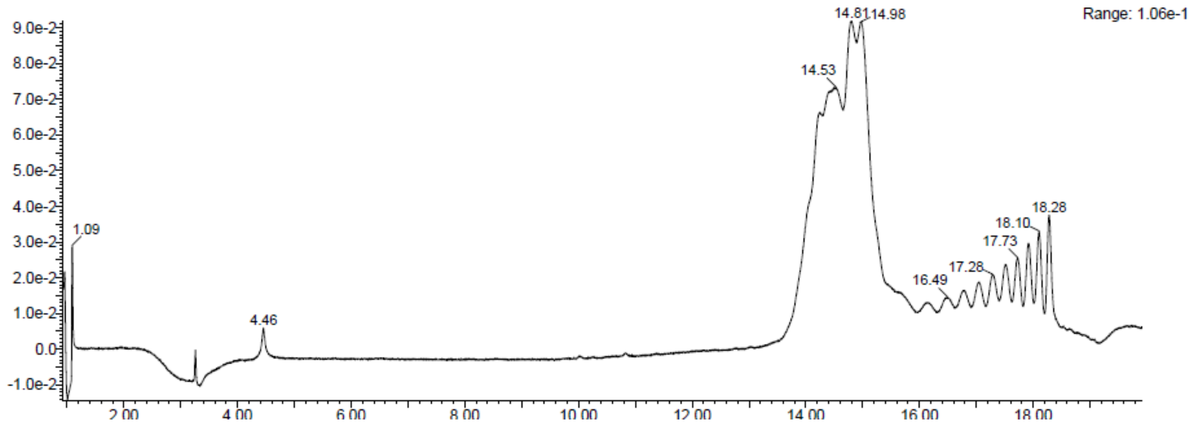

Figure S64: Reverse-phase UPLC of **Pal-Pip-LNA4** (UV absorbance at 260 nm vs time in min). Peaks eluting after16.00 min are likely from aggregation or hydrophobic adduct formation. A later peak at ~18.00 min is attributed to a system-related impurity, also observed in the blank (no sample) run.

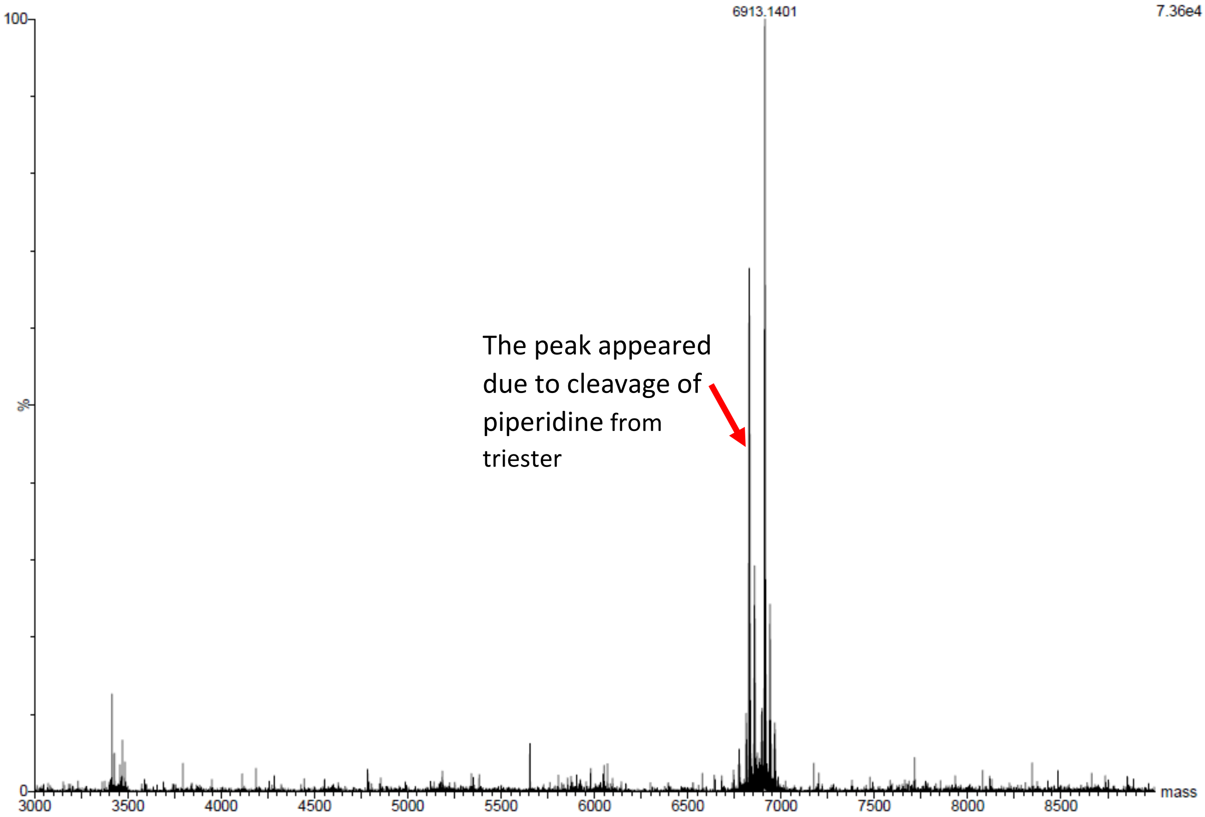

Figure S65: Mass spectrum (ES-) of **Pal-Pip-LNA4**. Required **6913.09** Da, found **6913.14** Da. y-axis = relative intensity (%), x-axis = mass in Da. Presence of 3 x and 4 x Pip-PTTE

**Pal-Pip-F4**	Pal-CCt CUt ACC tCA GUt ACA	

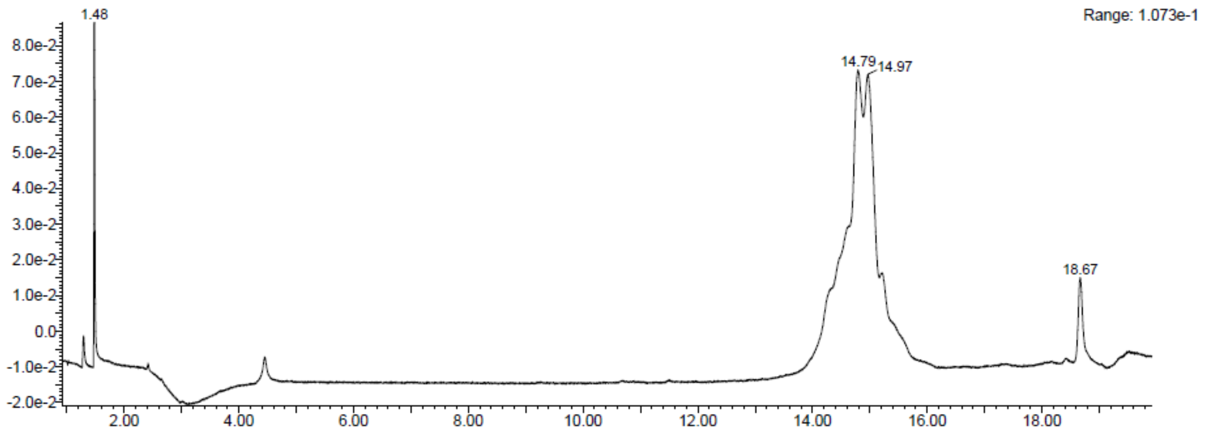

Figure S66: Reverse-phase UPLC of **Pal-Pip-F4** (UV absorbance at 260 nm vs time in min). A later peak at ~18.67 min is attributed to the system-related impurity, also observed in the blank (no sample) run.

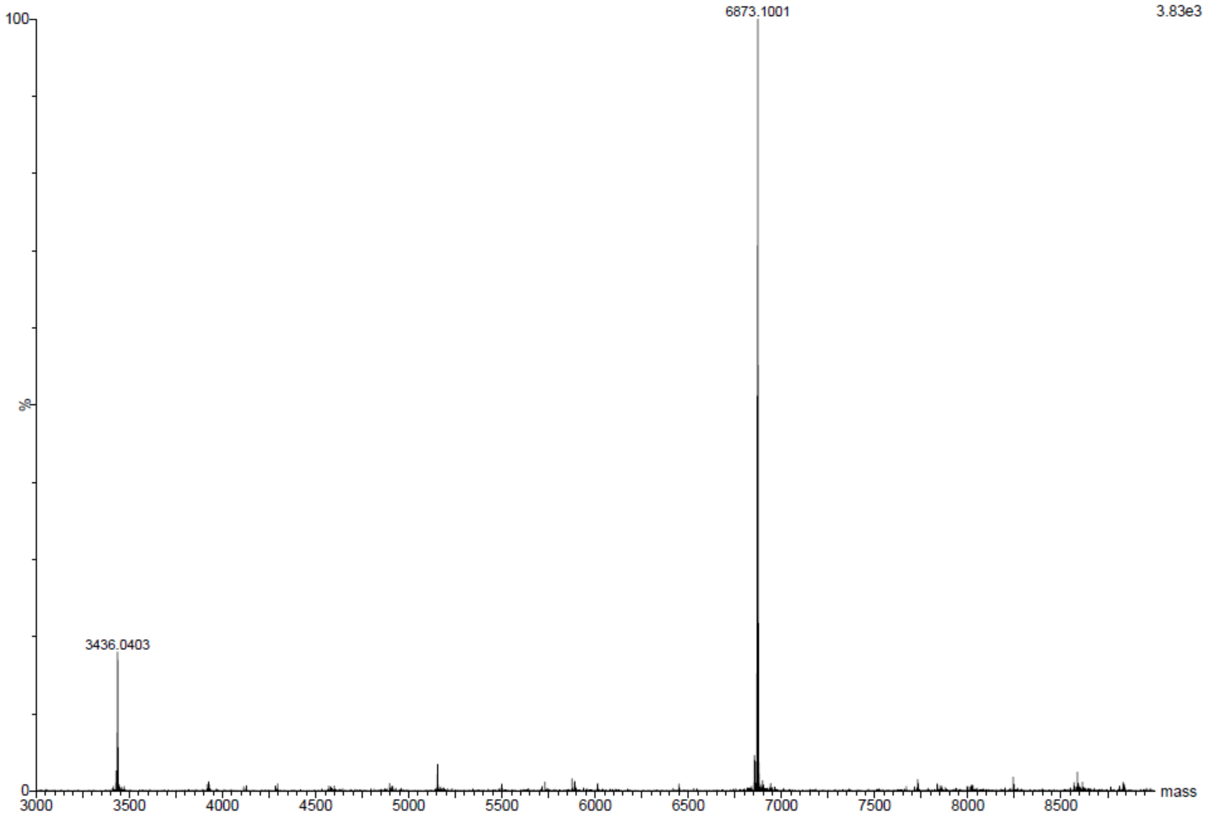

Figure S67: Mass spectrum (ES-) of **Pal-Pip-F4**. Required **6873.01** Da, found **6873.10** Da. y-axis = relative intensity (%), x-axis = mass in Da.

**Pal_2-LNA4**	Pal_2-CCT CUT ACC TCA GUT ACA	

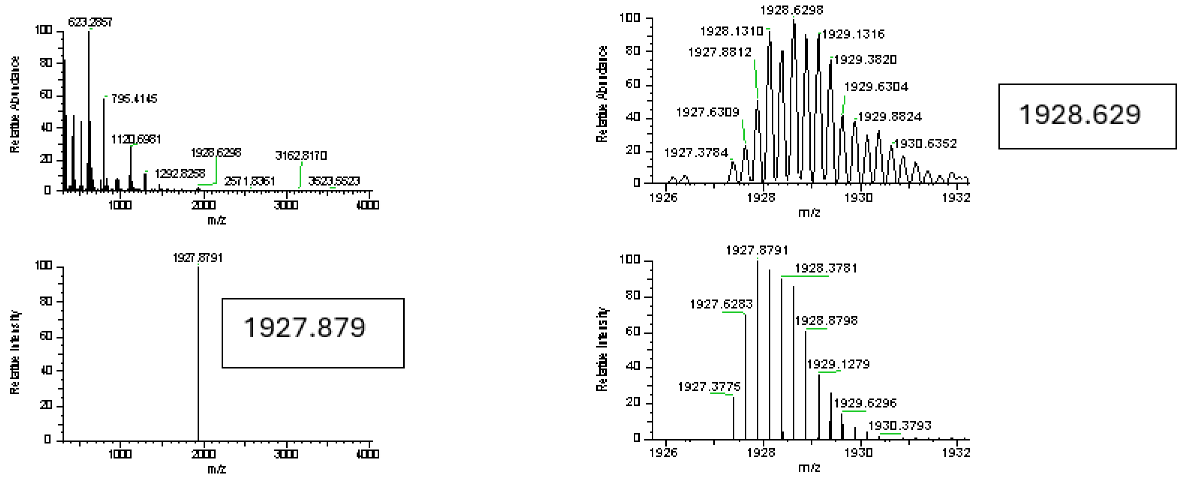

Figure S68: Mass spectrum (ES-) of **Pal_2-LNA4** Required **7719.04** Da. y-axis = relative intensity (%), x-axis = mass in Da, m/z corresponds to $(M-4H)^{4-}$ ion. Mass accuracy 0.5 ppm. Left: full mass spectrum and right: zoomed in mass spectrum.

**Pal-SS**	Pal-SS-CCU CUU ACC UCA GUU ACA	

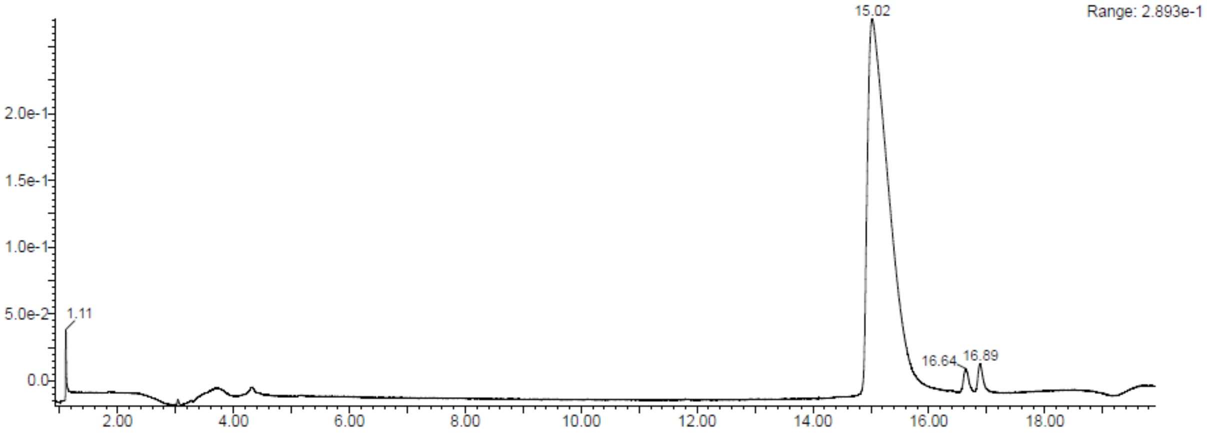

Figure S69: Reverse-phase UPLC of **Pal-SS** (UV absorbance at 260 nm vs time in min).

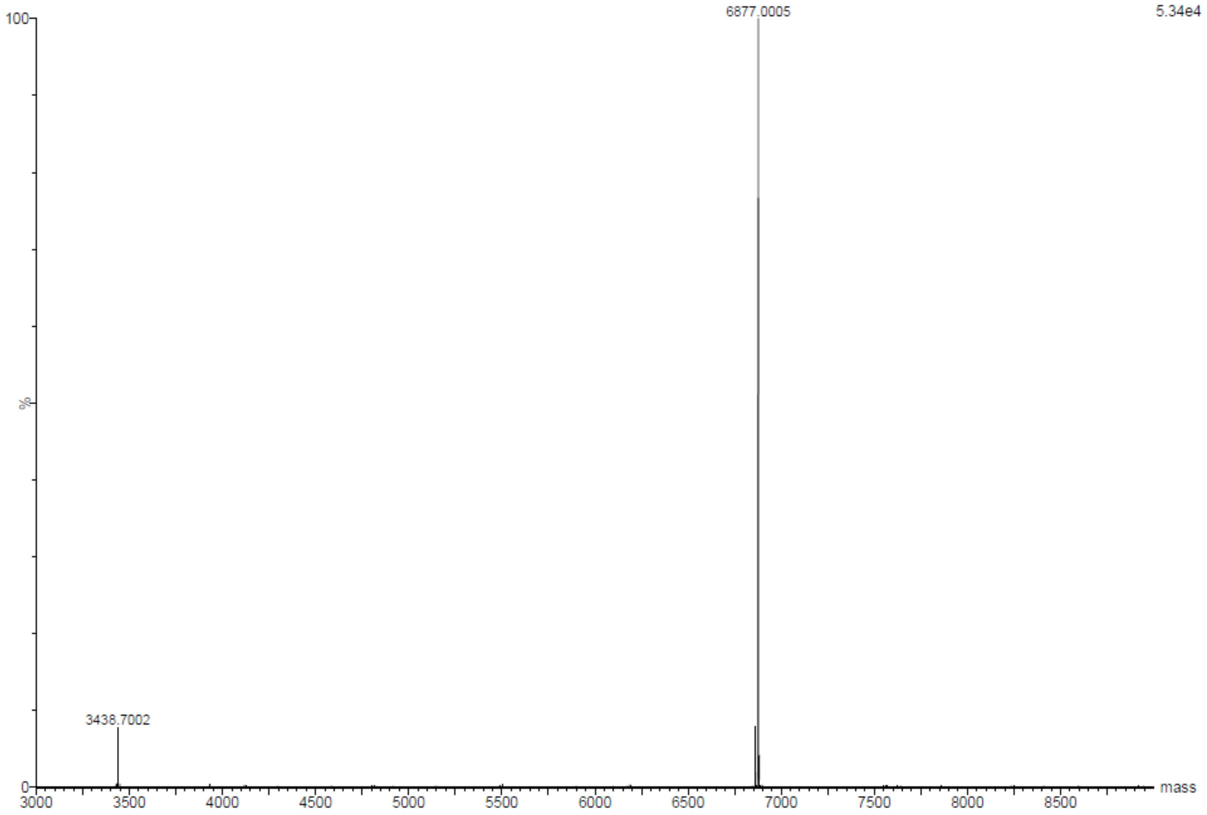

Figure S70: Mass spectrum (ES-) of **Pal-SS**. Required **6876.99** Da, found **6877.00** Da. y-axis = relative intensity (%), x-axis = mass in Da.

C16-SS	C16-SS-CCU CUU ACC UCA GUU ACA	

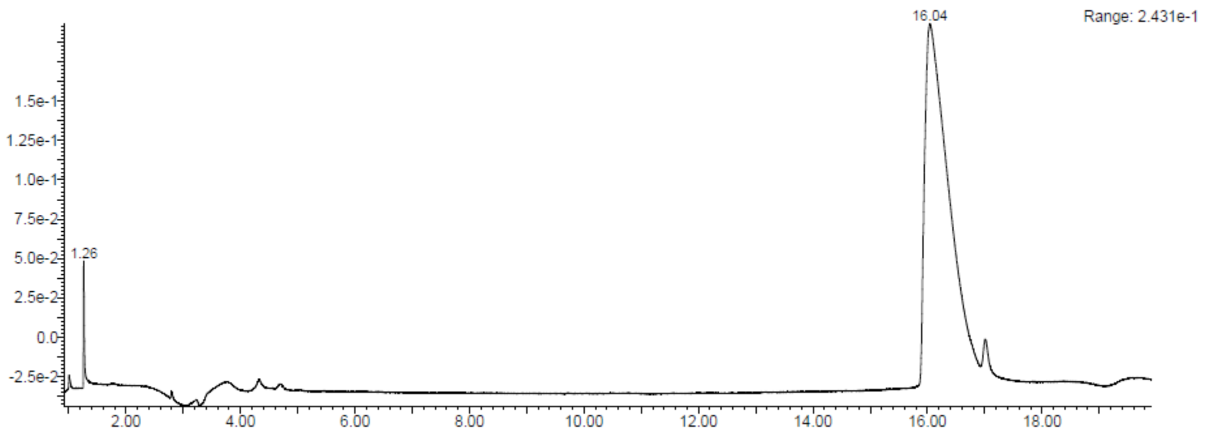

Figure S71: Reverse-phase UPLC of **C16-SS** (UV absorbance at 260 nm vs time in min).

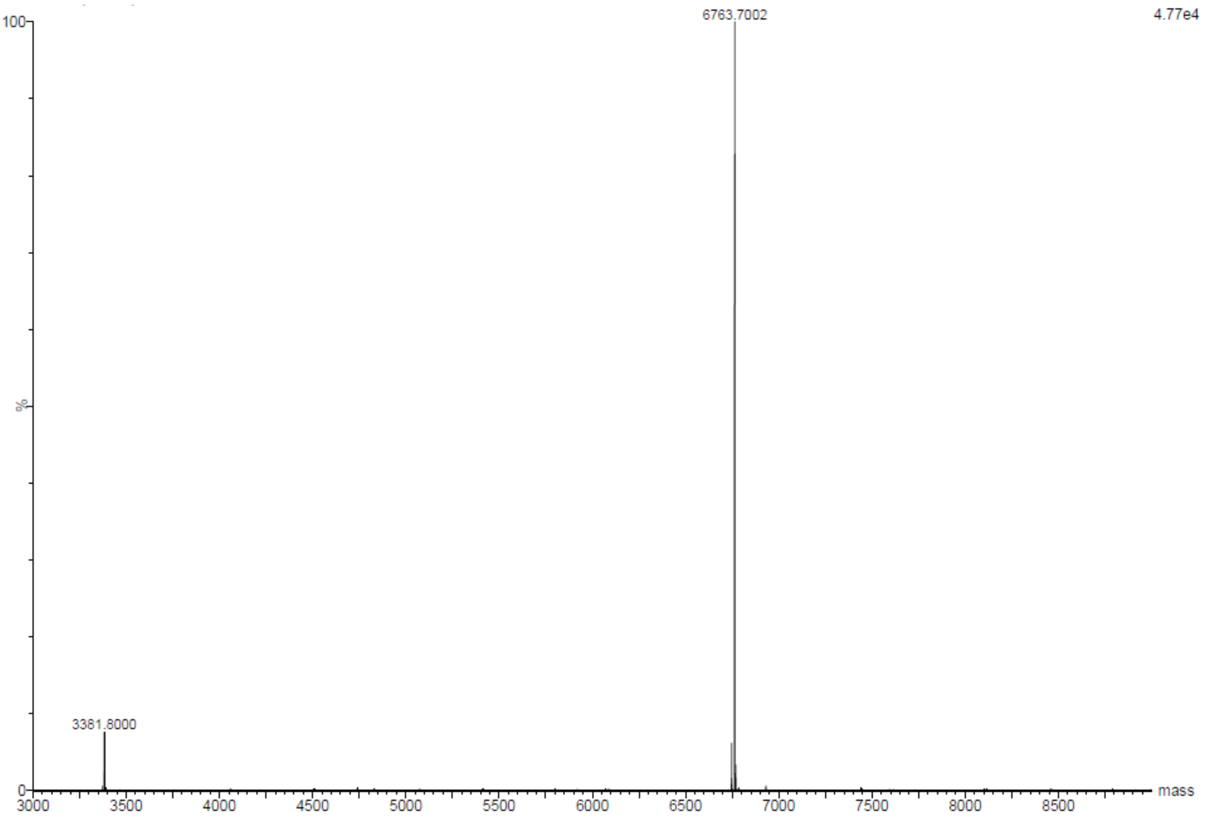

Figure S72: Mass spectrum (ES-) of **C16-SS**. Required **6763.83** Da, found **6763.70** Da. y-axis = relative intensity (%), x-axis = mass in Da.

**Pal-HEG**	Pal-HEG-CCU CUU ACC UCA GUU ACA	

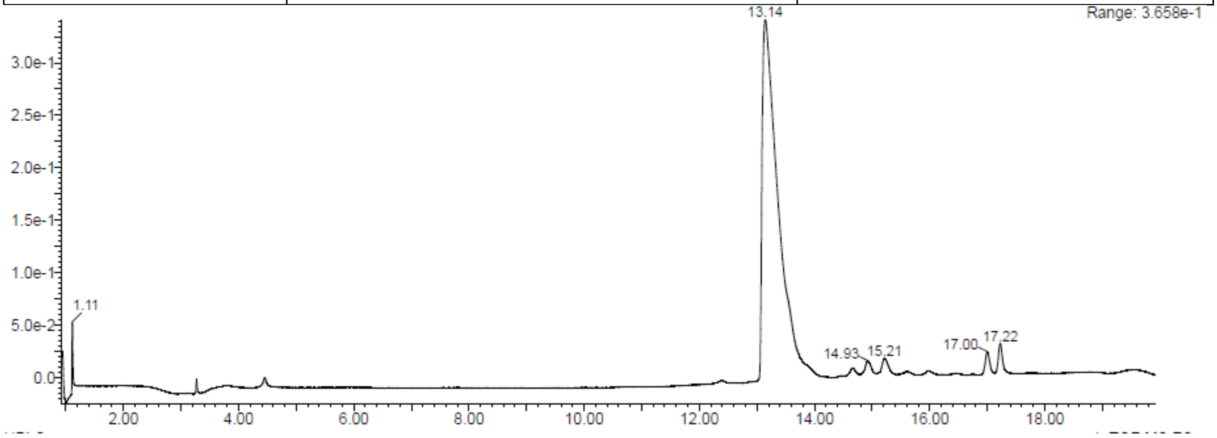

Figure S73: Reverse-phase UPLC of **Pal-HEG** (UV absorbance at 260 nm vs time in min).

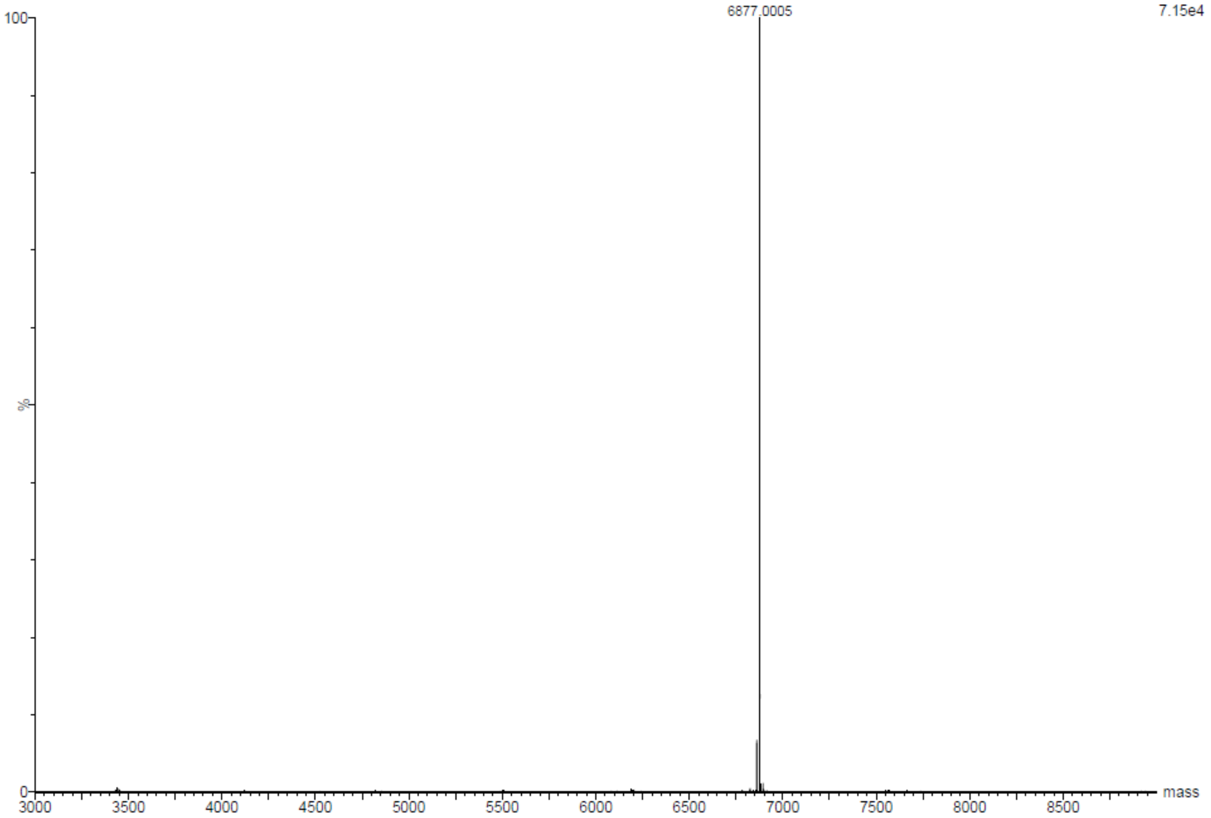

Figure S74: Mass spectrum (ES-) of **Pal-HEG**. Required **6876.81** Da, found **6877.00** Da. y-axis = relative intensity (%), x-axis = mass in Da.

**Pal-HEG-PO**	Pal_o-HEG_o-CCU CUU ACC UCA GUU ACA	

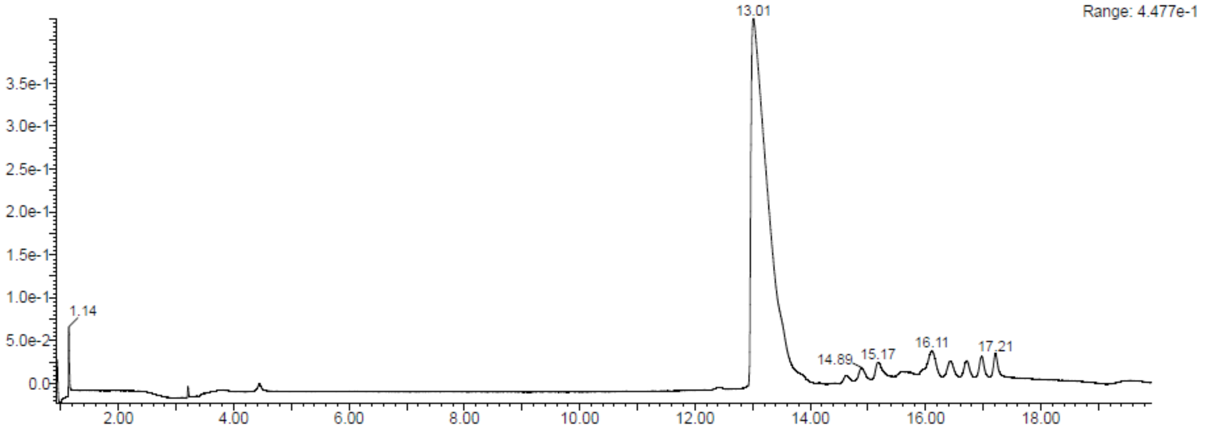

Figure S75: Reverse-phase UPLC of **Pal-HEG-PO** (UV absorbance at 260 nm vs time in min).

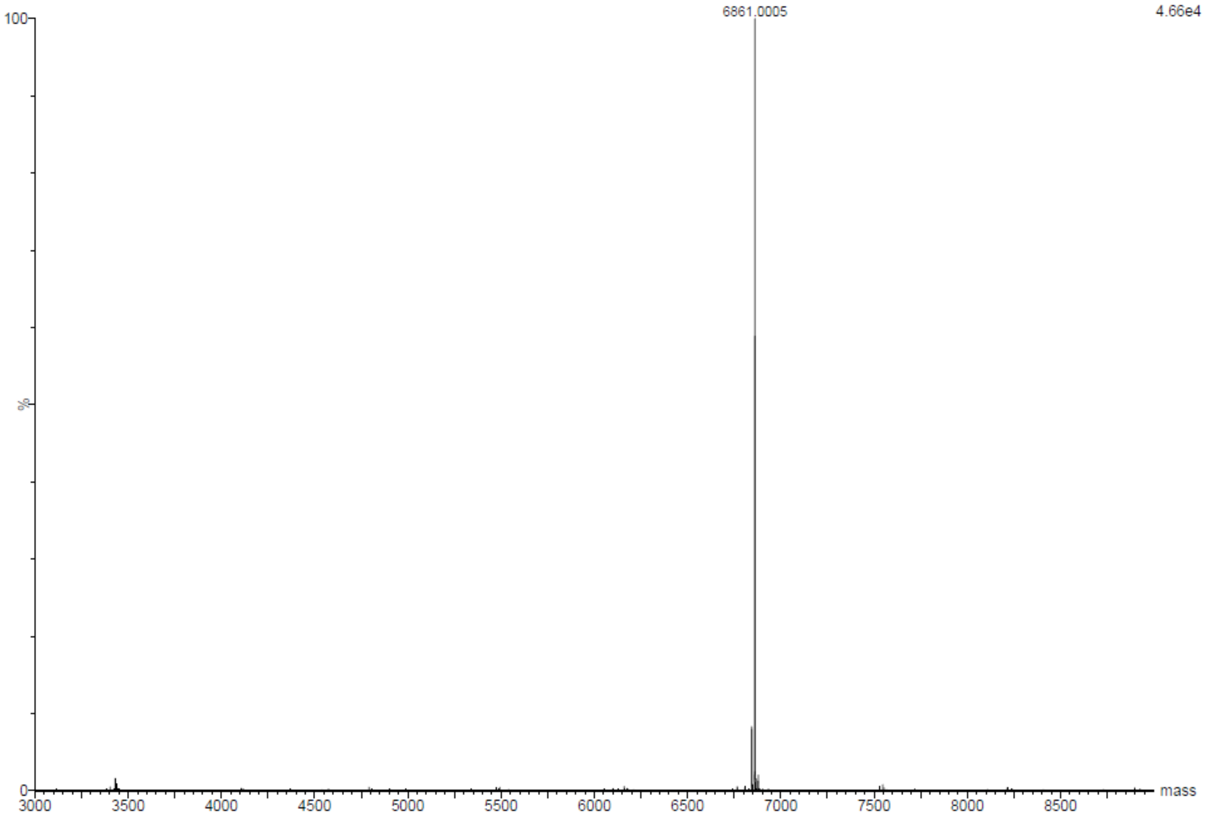

Figure S76: Mass spectrum (ES-) of **Pal-HEG-PO**. Required **6860.74** Da, found **6861.00** Da. y-axis = relative intensity (%), x-axis = mass in Da.

+Control-Pal	Pal-CCU CUU ACC UCA GUU ACA	

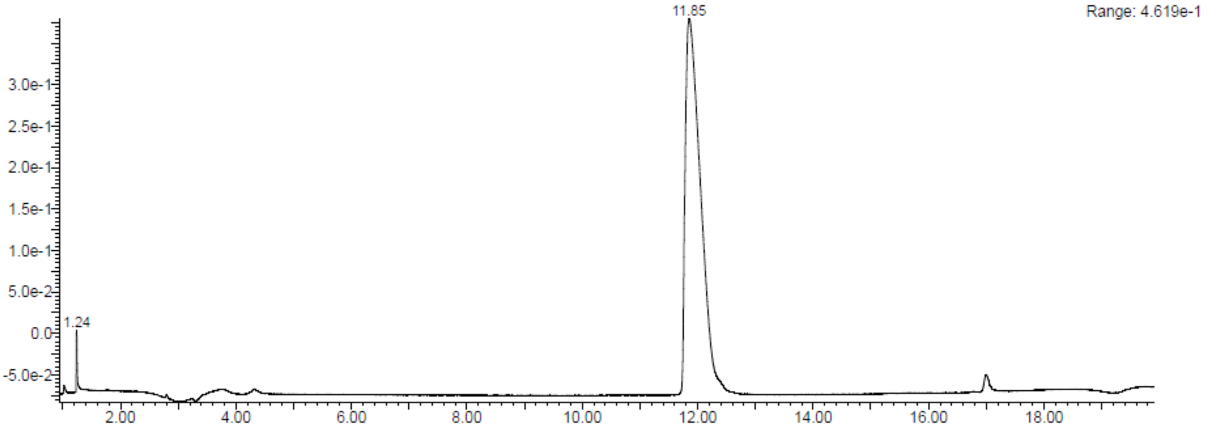

Figure S77: Reverse-phase UPLC of **+Control-Pal** (UV absorbance at 260 nm vs time in min).

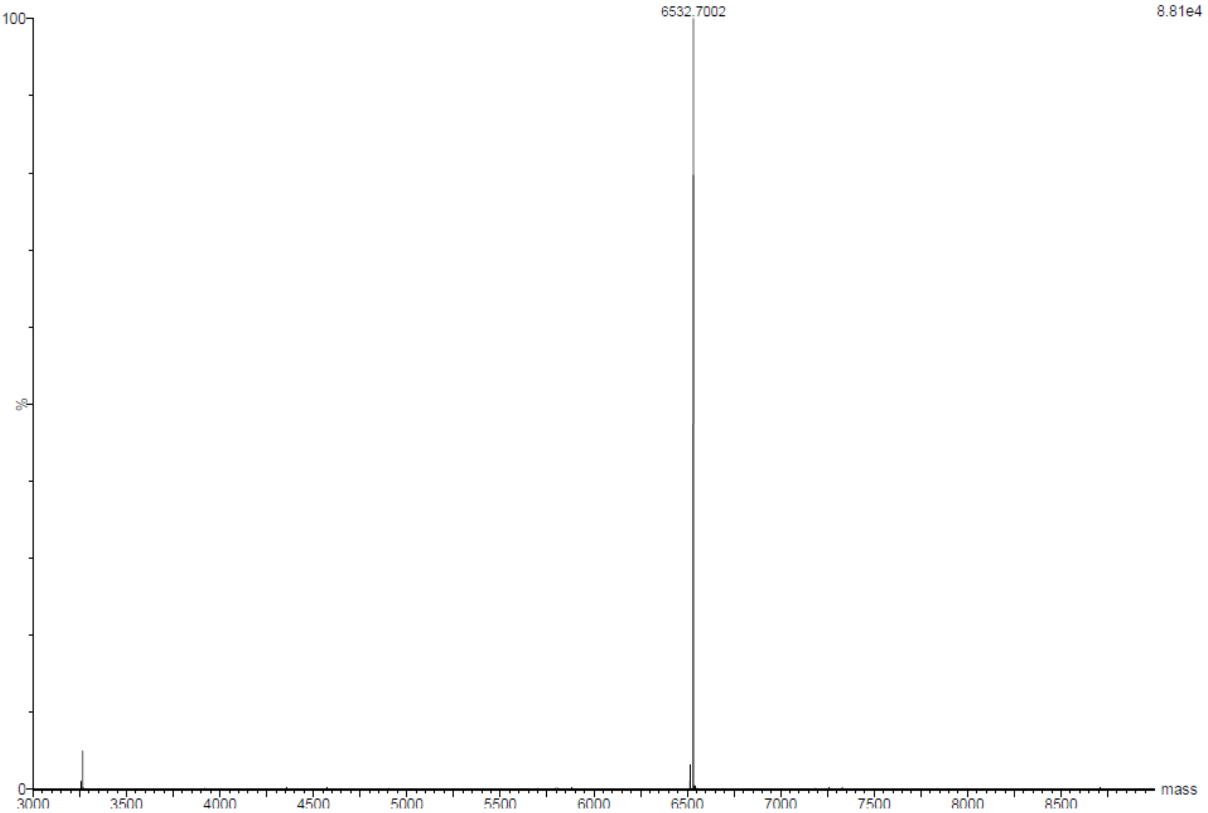

Figure S78: Mass spectrum (ES-) of **+Control-Pal**. Required **6532.51** Da, found **6532.70** Da. y-axis = relative intensity (%), x-axis = mass in Da.

+Control-Pal2	Pal_2-CCU CUU ACC UCA GUU ACA	

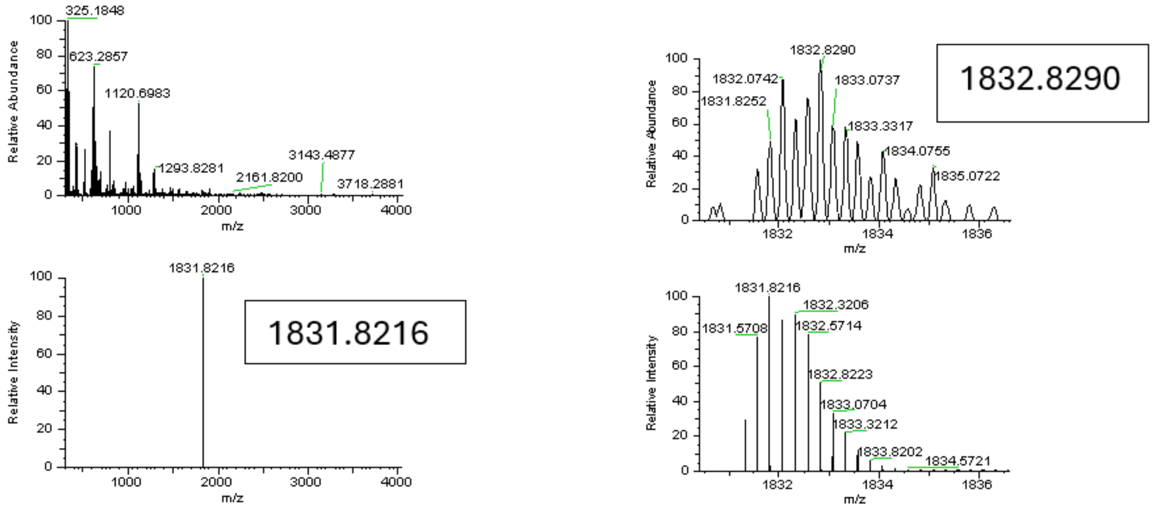

Figure S79: Mass spectrum (ES-) of **+Control-Pal2**. Requires **7332.51** Da, found **7331.316** Da. y-axis = relative intensity (%), x-axis = mass in Da. m/z corresponds to $(M\text{-}3H)^{4-}$ ion. Mass accuracy 2 ppm. Stoichiometry is different by one hydrogen atom. *Left:* Full mass spectrum and *right:* zoomed in spectrum

**-Control-Pal**	Pal-CCU CAU UCA CUC GAU UCA	

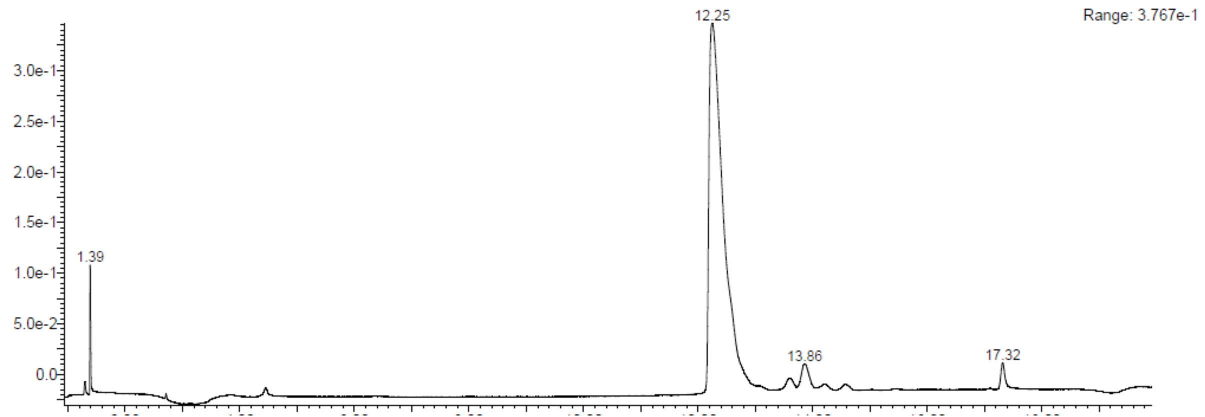

Figure S80: Reverse-phase UPLC of **-Control-Pal** after HPLC purification (UV absorbance at 260 nm vs time in min).

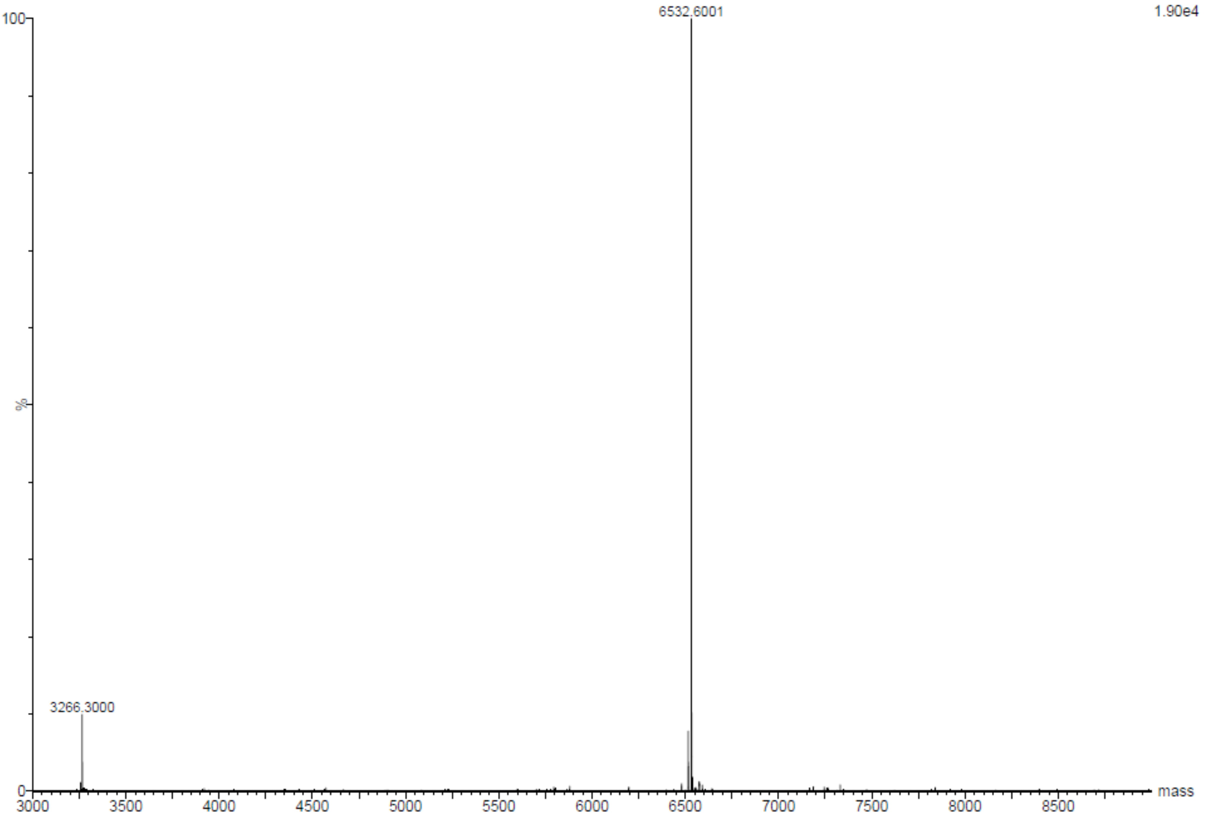

Figure S81: Mass spectrum (ES-) of **-Control-Pal**. Required **6532.51** Da, found **6532.60** Da. y-axis = relative intensity (%), x-axis = mass in Da.

**Pal-LNA2-OMe2**	Pal-CCUCUTACCUCAGUTACA	

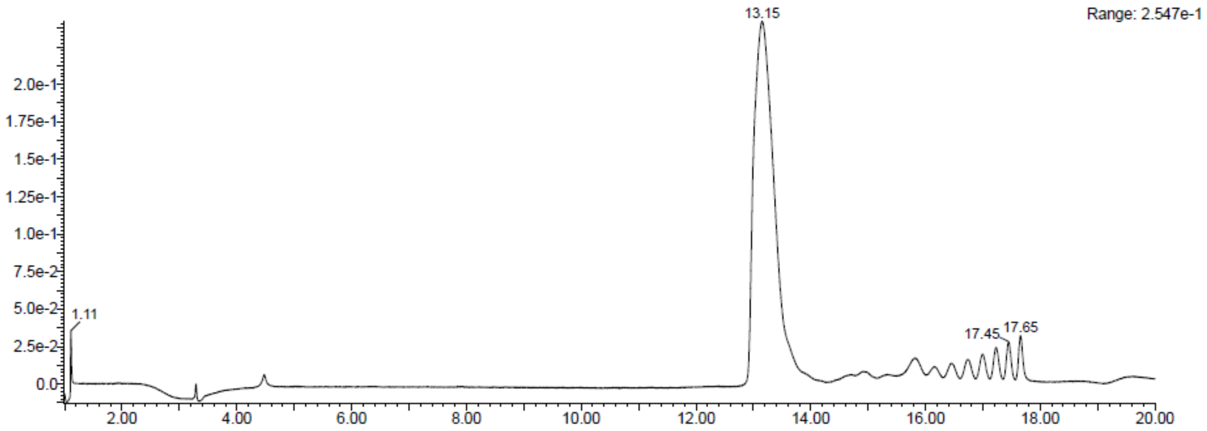

Figure S82: Reverse-phase UPLC of **Pal-LNA2-OMe2** after HPLC purification (UV absorbance at 260 nm vs time in min).

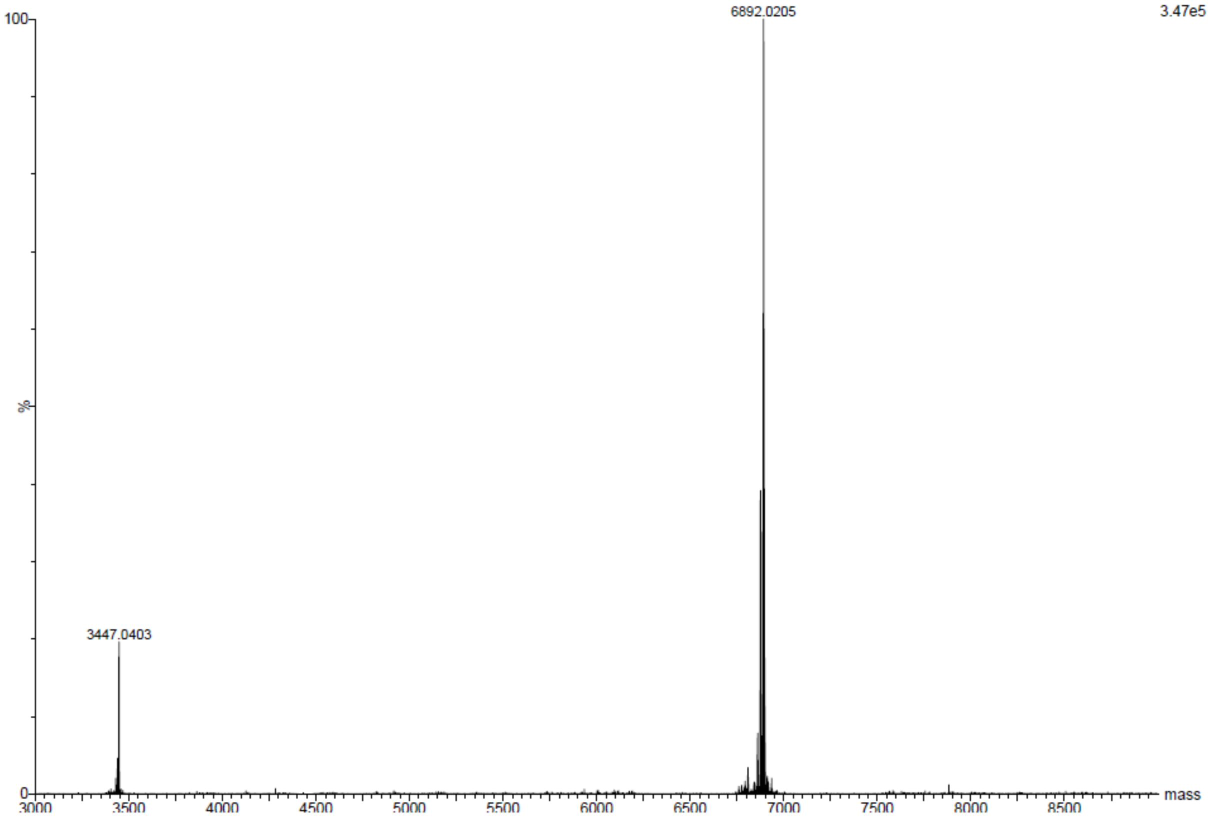

Figure S83: Mass spectrum (ES-) of **Pal-LNA2-OMe2**. Required **6893.01** Da, found **6892.02** Da. y-axis = relative intensity (%), x-axis = mass in Da.

**Pal-LNA2(C)-PS**	Pal-CCU CUU ACC UCA GUUACA	

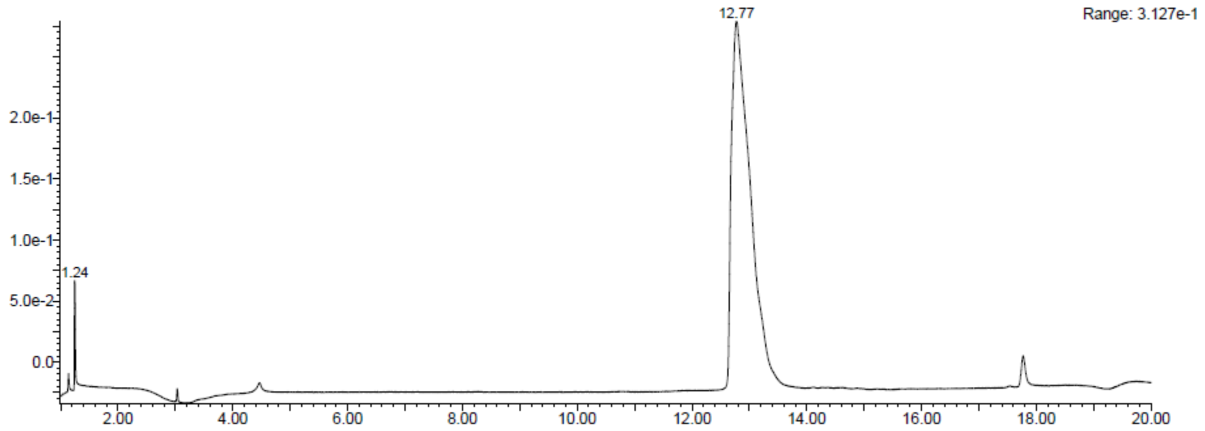

Figure S84: Reverse-phase UPLC of **Pal-LNA2(C)-PS** after HPLC purification (UV absorbance at 260 nm vs time in min).

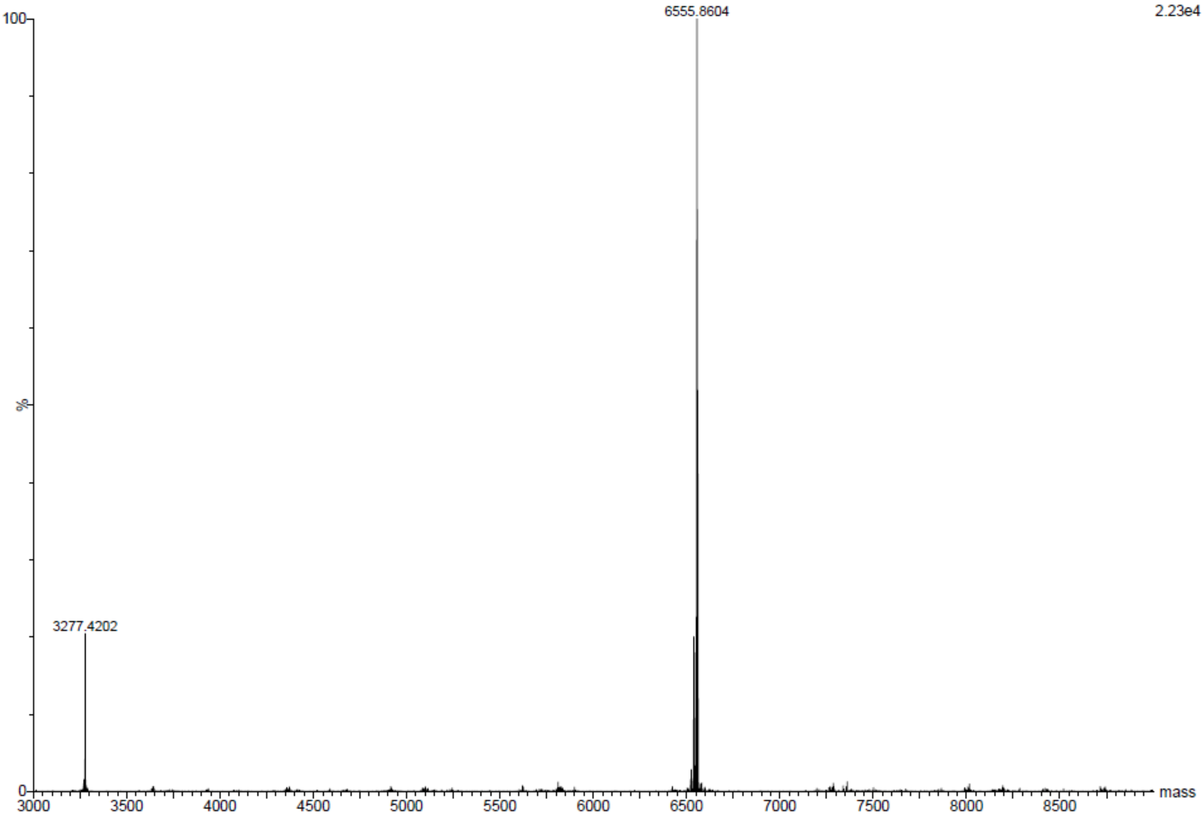

Figure S85: Mass spectrum (ES-) of **Pal-LNA2(C)-PS**. Required **6556.54** Da, found **6555.86** Da. y-axis = relative intensity (%), x-axis = mass in Da. The smaller peaks close to desired peak (M-16) correspond to the S→O exchange due to conversion of phosphorothioate (PS) to phosphodiester.

**Pal-LNA2-F2**	Pal-CCTCUTACCTCAGUTACA	

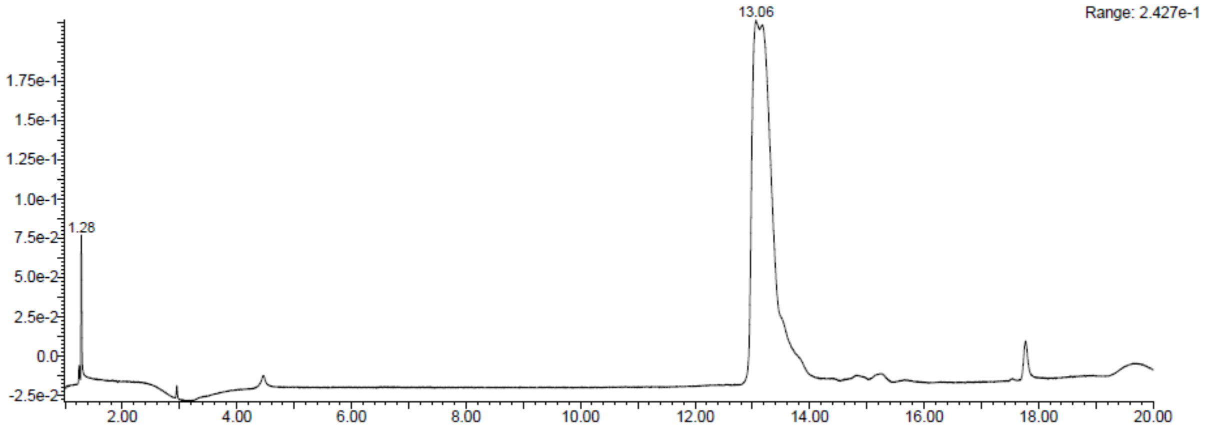

Figure S86: Reverse-phase UPLC of **Pal-LNA2-F2** after HPLC purification (UV absorbance at 260 nm vs time in min).

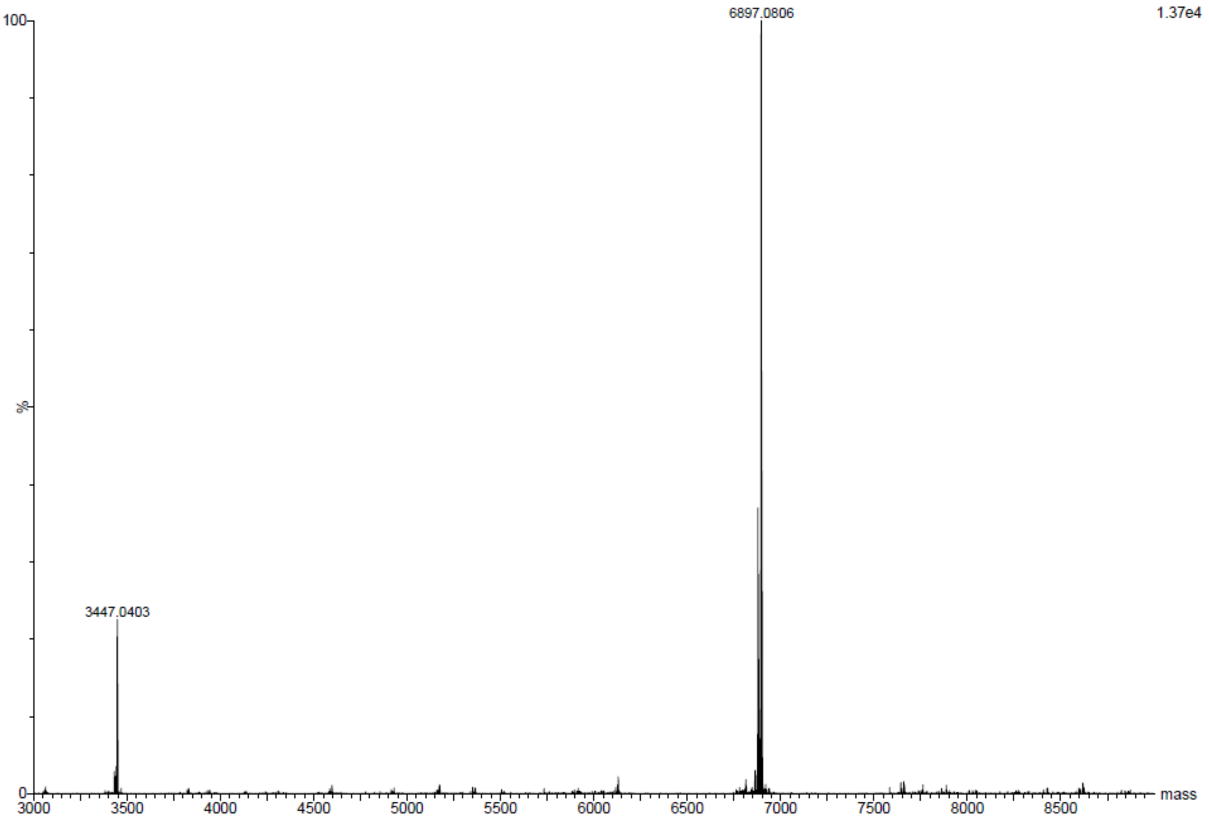

Figure S87: Mass spectrum (ES-) of **Pal-LNA2-F2**. Required **6896.99** Da, found **6897.08** Da. y-axis = relative intensity (%), x-axis = mass in Da.

**Pal-LNA2-F2-PS**	Pal-CCTCUTACCTCAGUTACA	

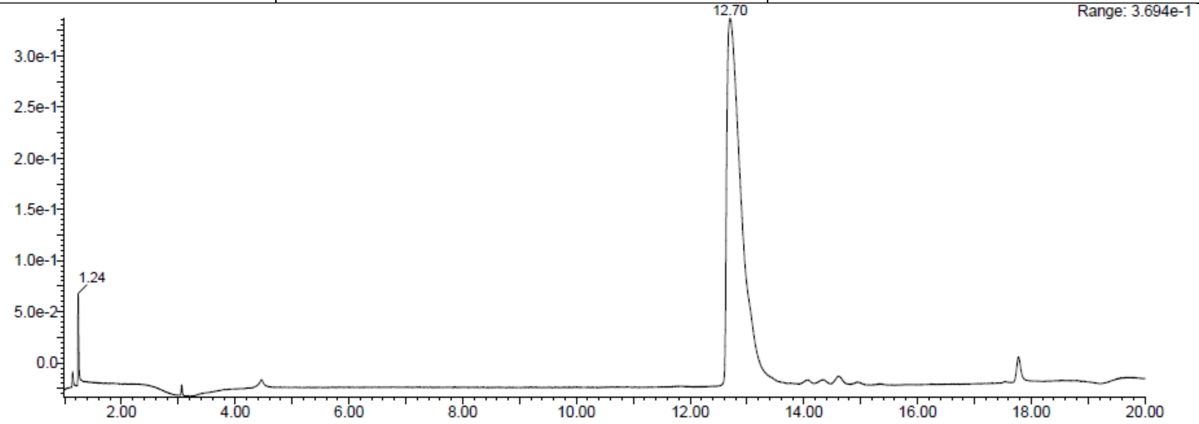

Figure S88: Reverse-phase UPLC of **Pal-LNA2-F2-PS** after HPLC purification (UV absorbance at 260 nm vs time in min).

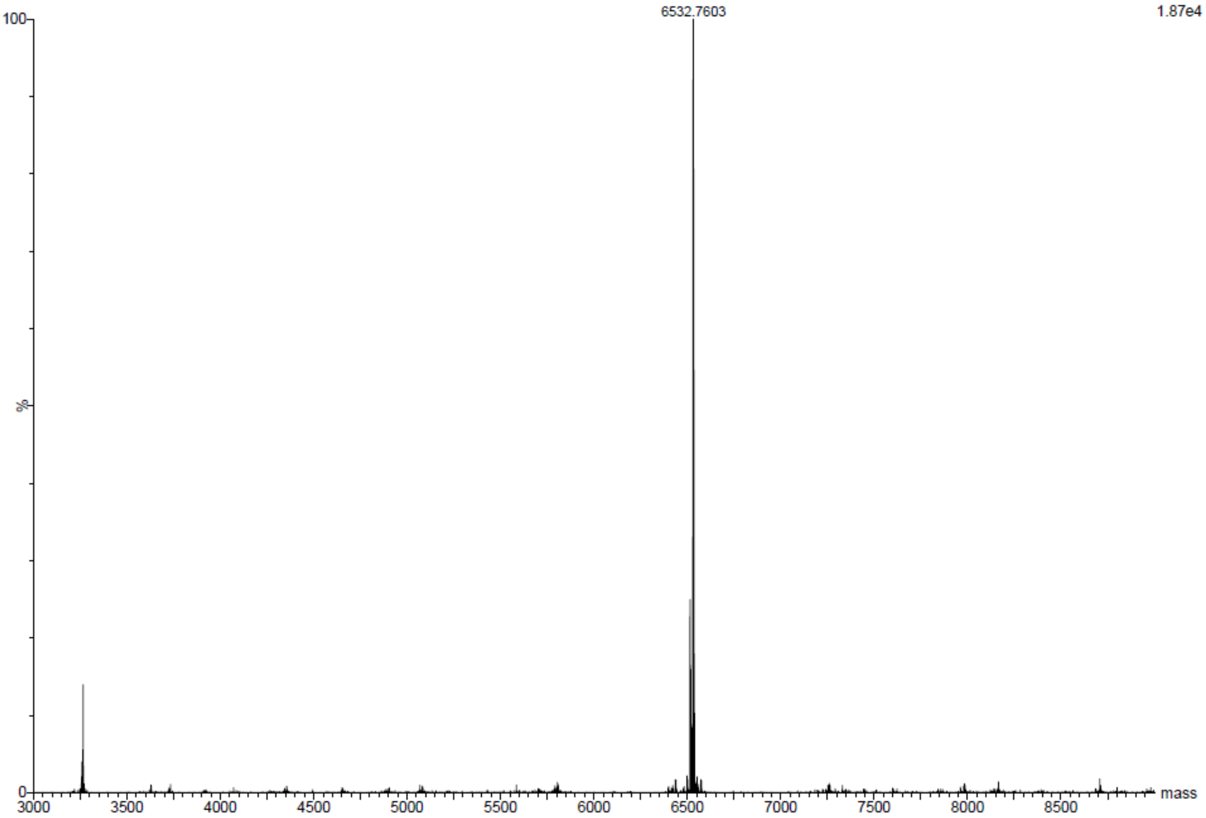

Figure S89: Mass spectrum (ES-) of **Pal-LNA2-F2-PS**. Required **6532.46** Da, found **6532.76** Da. y-axis = relative intensity (%), x-axis = mass in Da.

**Pal-Pip-LNA2**	Pal-CCU CUt ACC UCA GUt ACA	

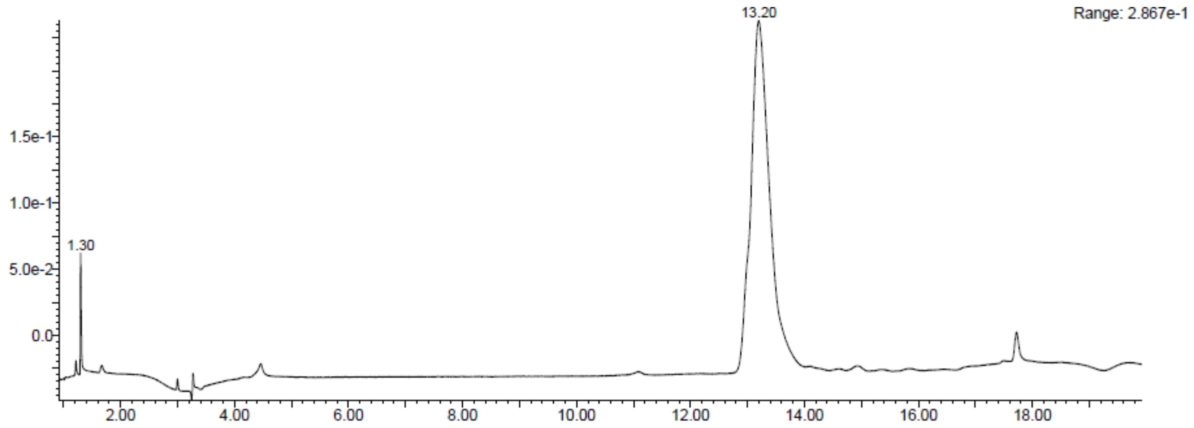

Figure S90: Reverse-phase UPLC of **Pal-Pip-LNA2** after HPLC purification (UV absorbance at 260 nm vs time in min).

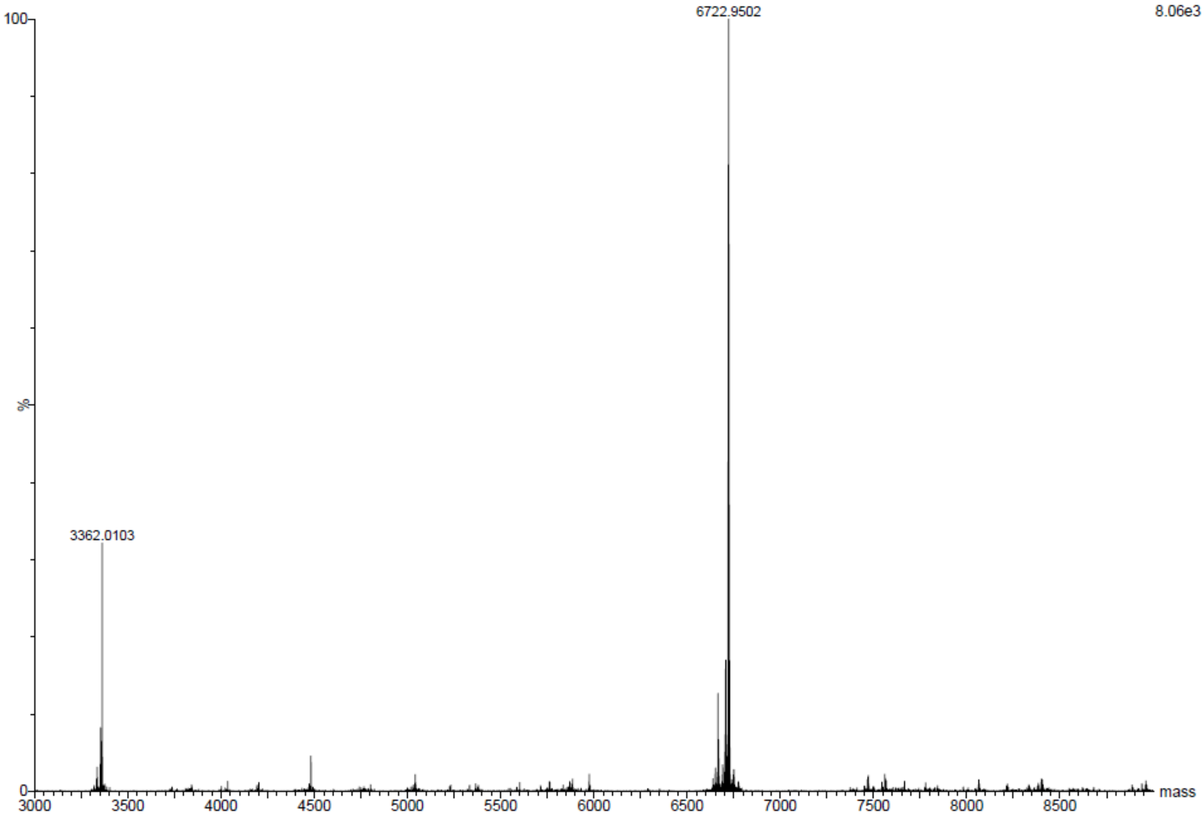

Figure S91: Mass spectrum (ES-) of **Pal-Pip-LNA2**. Required **6722.80** Da, found **6722.95** Da. y-axis = relative intensity (%), x-axis = mass in Da.

Pal-LNA2-PS	Pal-CCU CUT ACC UCA GUT ACA	

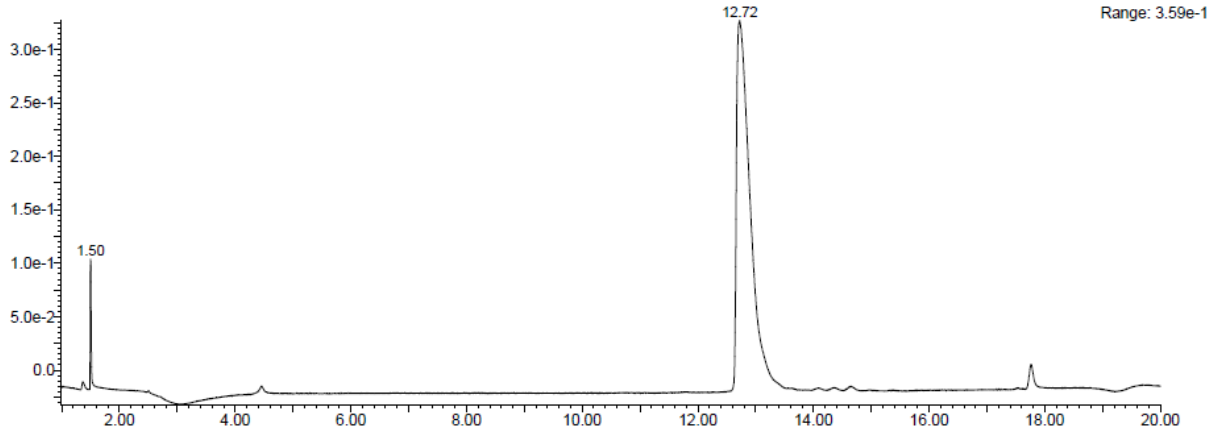

Figure S92: Reverse-phase UPLC of **Pal-LNA2-PS** after HPLC purification (UV absorbance at 260 nm vs time in min).

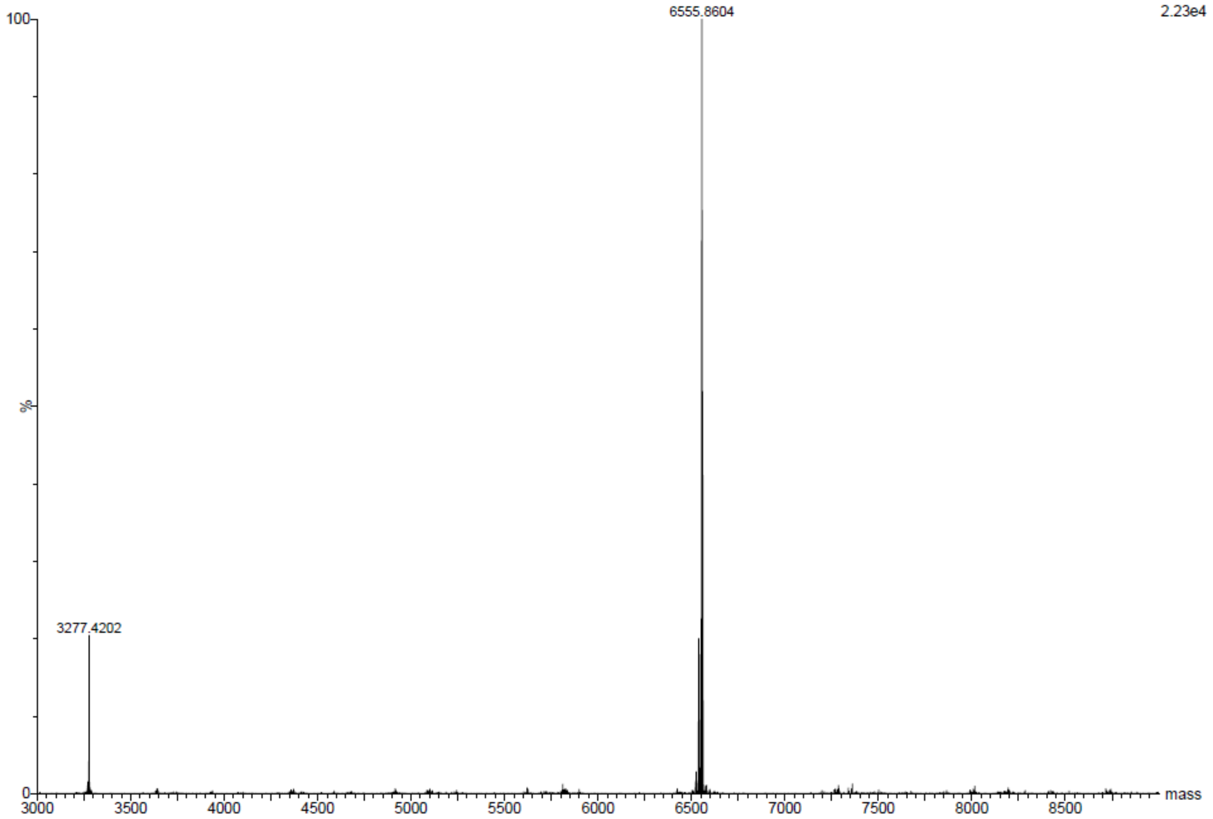

Figure S93: Mass spectrum (ES-) of **Pal-LNA2-PS**. Required **6556.54** Da, found **6555.86** Da. y-axis = relative intensity (%), x-axis = mass in Da.

Pal-F2-PS	Pal-CCU CUT ACC UCA GUT ACA	

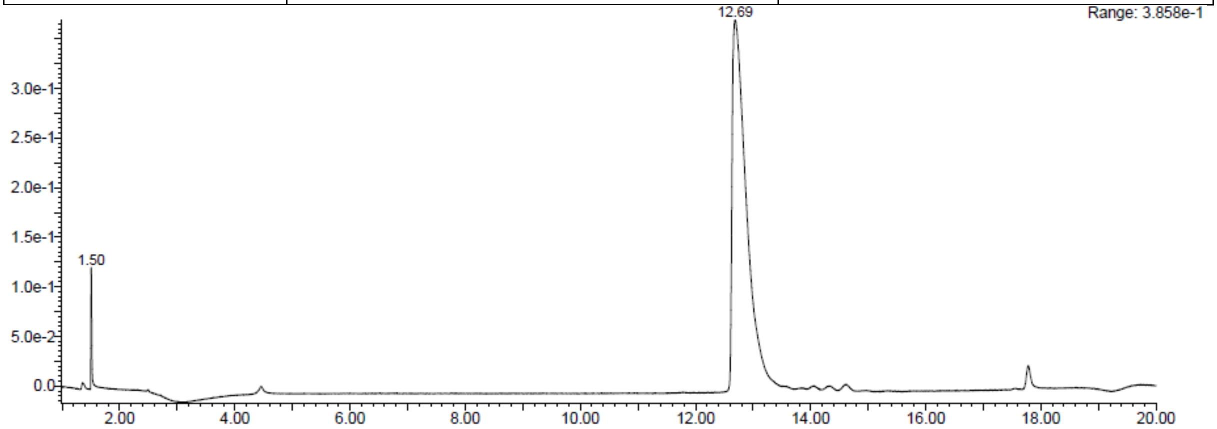

Figure S94: Reverse-phase UPLC of **Pal-F$_2$-PS** after HPLC purification (UV absorbance at 260 nm vs time in min).

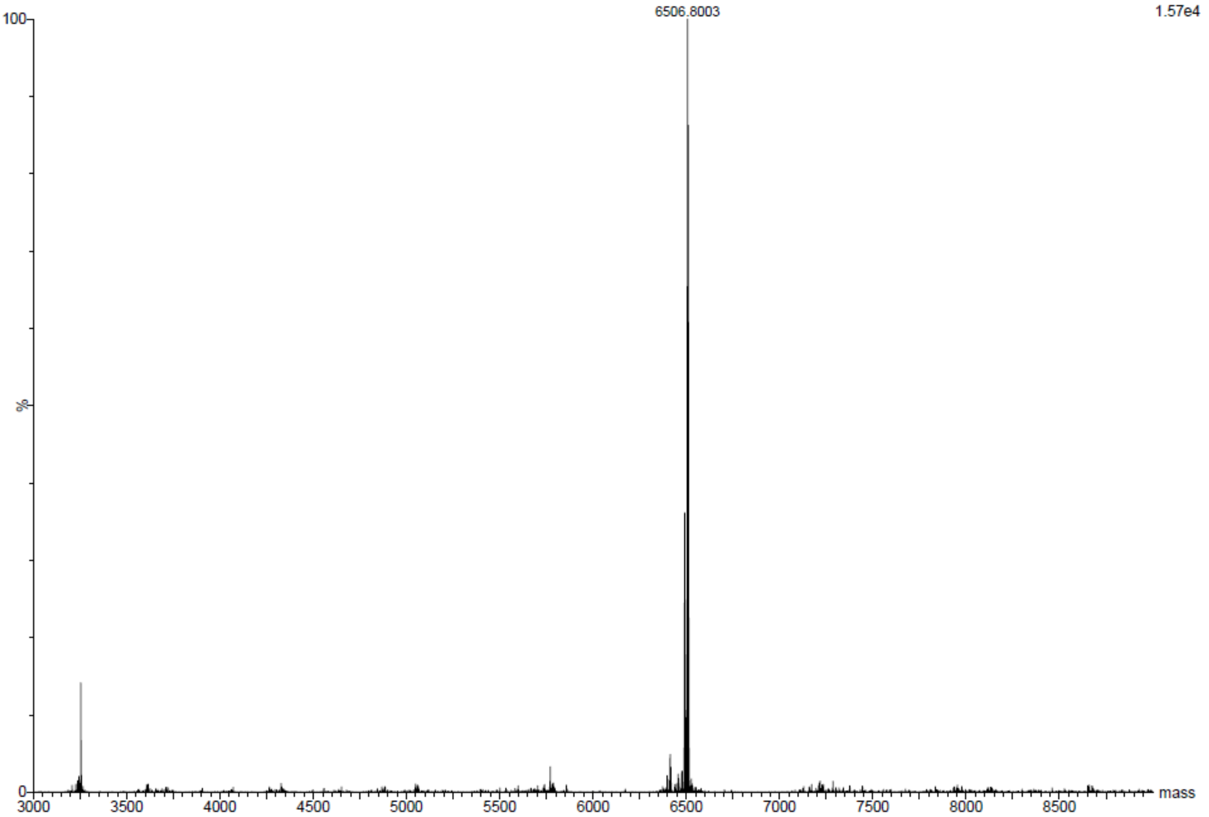

Figure S95: Mass spectrum (ES-) of **Pal-F$_2$-PS**. Required **6508.44** Da, found **6506.80** Da. y-axis = relative intensity (%), x-axis = mass in Da.

Pal-MOE$_2$-PS	Pal-CCU CUT ACC UCA GUT ACA	

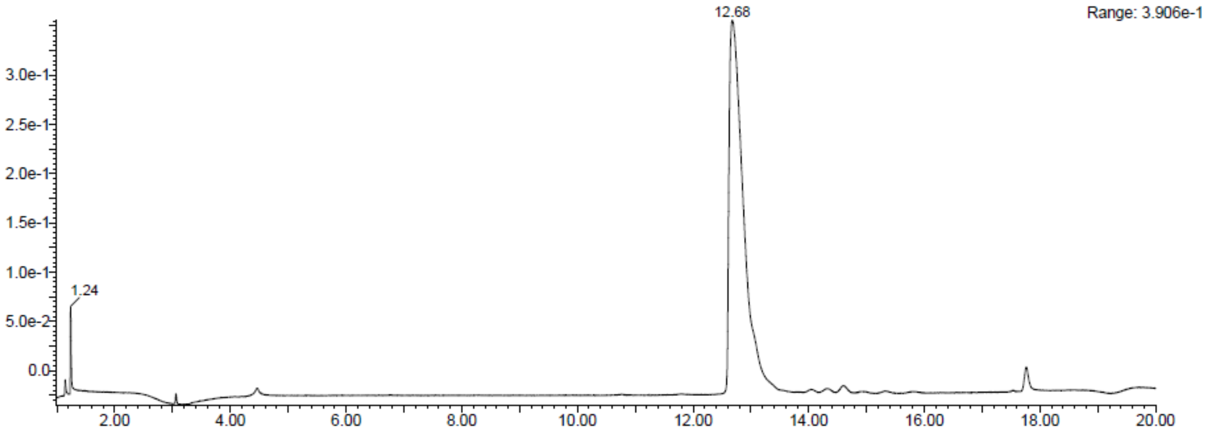

Figure S96: Reverse-phase UPLC of **Pal-MOE$_2$-PS** after HPLC purification (UV absorbance at 260 nm vs time in min).

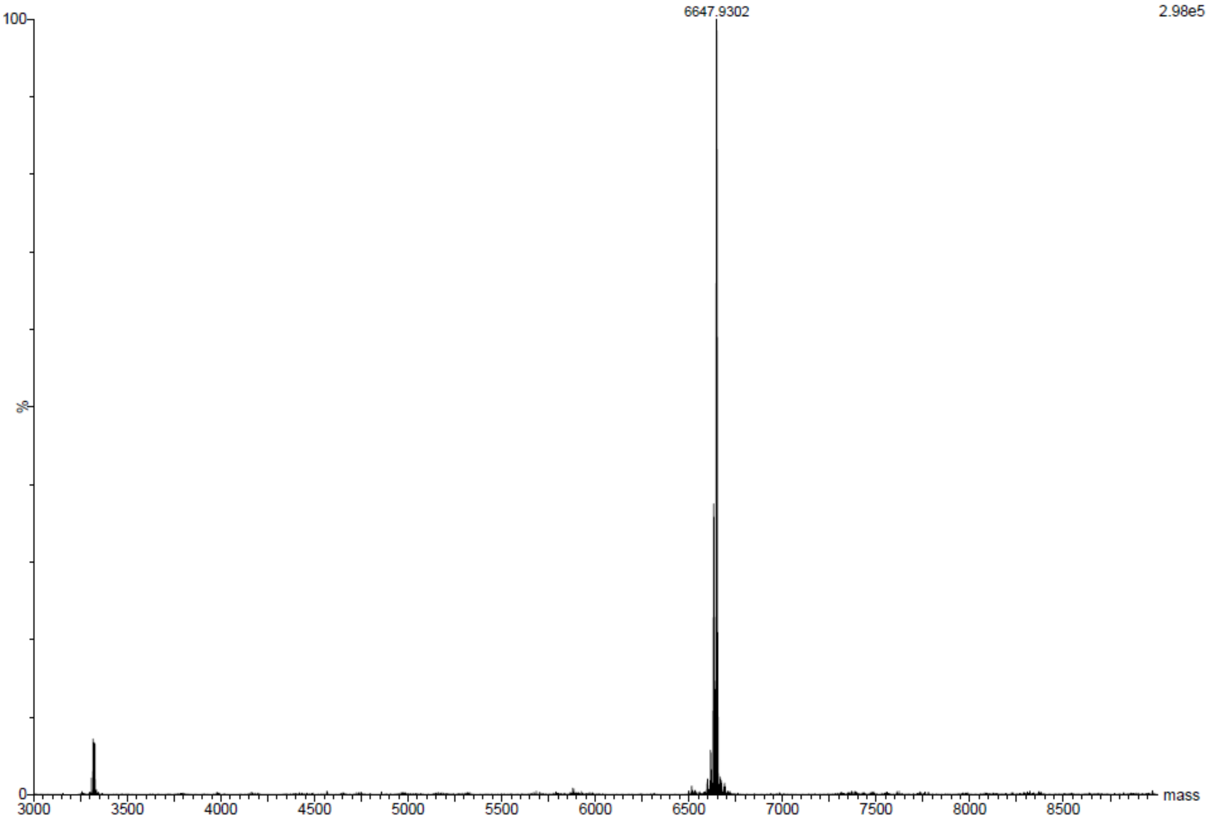

Figure S97: Mass spectrum (ES-) of **Pal-MOE$_2$-PS**. Required **6648.67** Da, found **6647.93** Da. y-axis = relative intensity (%), x-axis = mass in Da.

Pal-2´MOEPS+	Pal-CCU CUU ACC UCA GUU ACA	

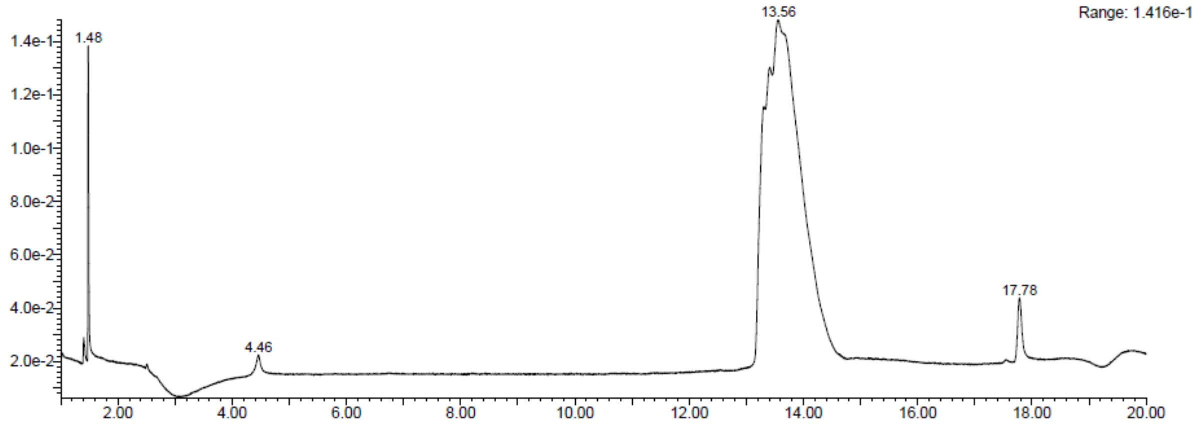

Figure S98: Reverse-phase UPLC of **Pal-2´MOEPS+** after HPLC purification (UV absorbance at 260 nm vs time in min).

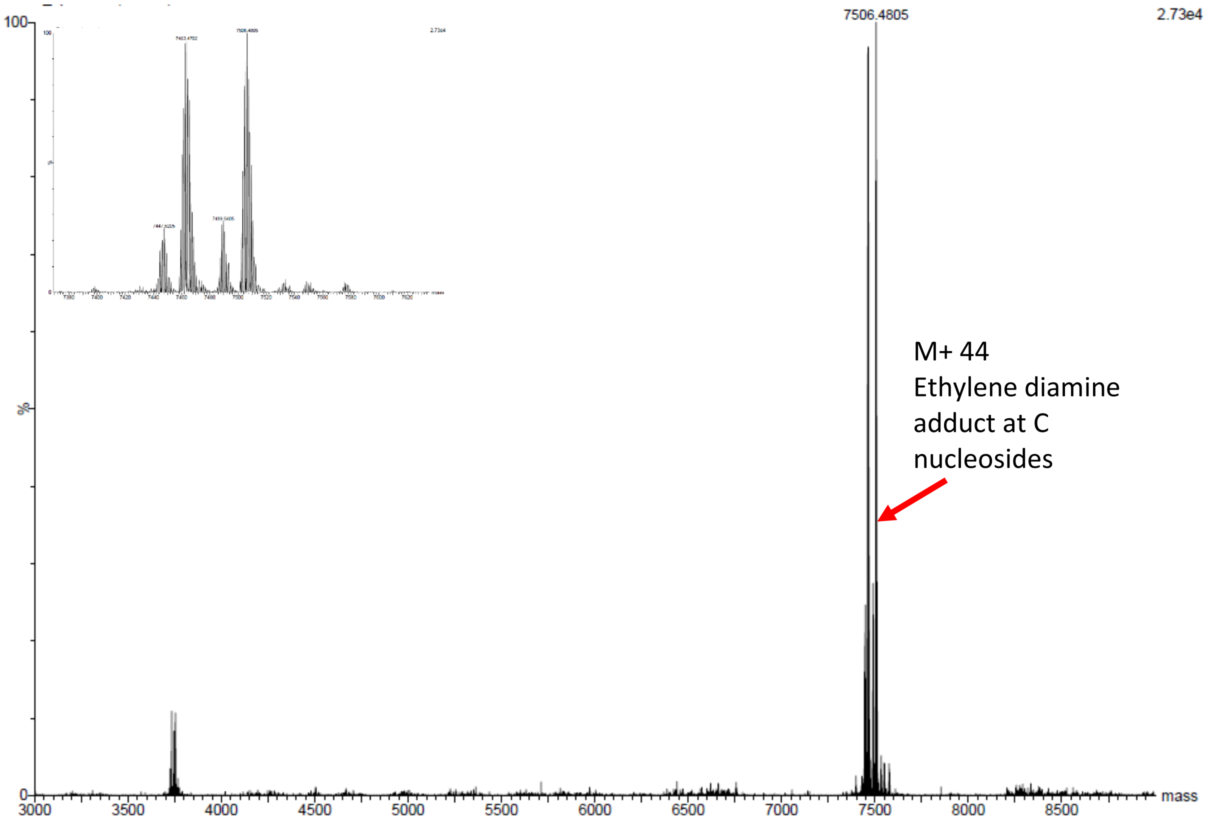

Figure S99: Mass spectrum (ES-) of **Pal-2´MOEPS+**. Required **7463.77** Da, found **7463.47** Da. y-axis = relative intensity (%), x-axis = mass in Da.

3.0 Thermal Stability Studies

UV melting experiments were performed using a Cary 4000 scan UV-Vis spectrophotometer. 2 nmol of each oligonucleotide was dissolved in 1 mL of 10 mM phosphate buffer containing 100 mM NaCl (for DNA Target) and 25 mM NaCl (for RNA target) at pH 7.0. The samples were first denatured by heating to 85 °C (2-10 °C/min) and then annealed by slowly cooling to 20 °C. (1 °C/min). Six successive cycles of heating and cooling were performed at a gradient of 1 °C/min whilst recording the change in UV absorbance at 260 nm. The built-in Cary WinUV 3.0 software was then used to calculate the melting temperature from the first derivative of the melting curves. The curves shown are representative of three independent repeats, each consisting of at least two technical repeats. Tm values are an average of three experiments with an error of ±0.50 ºC

Table T3: Duplex melting temperatures (Tm) in °C of **OMe$_2$, OMe$_4$ and OMe$_6$** oligonucleotides with complementary DNA and RNA. Nucleotides marked in black have 2´-OMe ribose sugars and phosphorothioate internucleoside linkages. Nucleotides marked in black-bold-underlined are 2´-OMe tetrahydropyran-4-yl phosphothiotriesters.

**Oligonucleotide**	**Sequence (5´→3´)**	**DNA target Tm (ΔTm)**	**RNA target Tm (ΔTm)**
**OMe$_2$**	CCU CU**T** ACC UCA GU**T** ACA	44.8 (-3.7)	58.4 (-3.1)
**OMe$_4$**	CC**T** CU**T** ACC **T**CA GU**T** ACA	41.8 (-6.7)	56.8 (-4.5)
**OMe$_6$**	CC**T** C**TT** ACC **T**CA G**TT** ACA	38.2 (-10.3)	55.0 (-6.3)

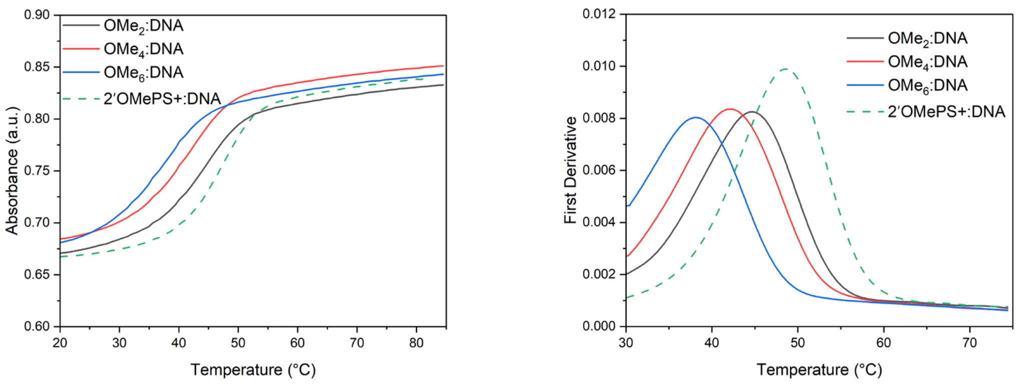

Figure S100: UV melting studies of **OMe$_2$, OMe$_4$ and OMe$_6$** oligonucleotides with complementary DNA. *Left:* Representative UV melting curves measured using 2 nmol of each oligonucleotide (final concentration 2 µM) in 10 mM Na-phosphate buffer, 100 mM NaCl, pH = 7.0; *Right:*1st derivative of melting curves.

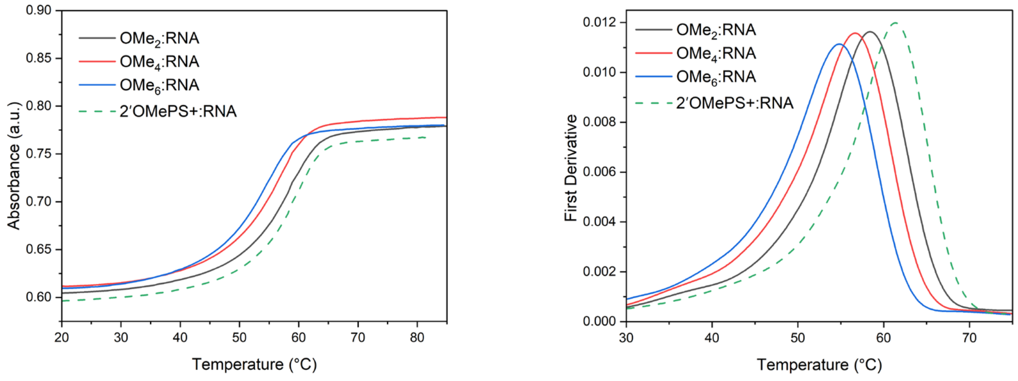

Figure S101: UV melting studies of **OMe$_2$, OMe$_4$ and OMe$_6$** oligonucleotides with complementary RNA. *Left:* Representative UV melting curves measured using 2 nmol of each oligonucleotide (final concentration 2 µM) in 10 mM Na-phosphate buffer, 25 mM NaCl, pH = 7.0; *Right:* 1st derivative of melting curves.

Table T4: Duplex melting temperatures (Tm) in °C of **MOE$_2$, MOE$_4$ and MOE$_6$** oligonucleotides with complementary DNA and RNA. Nucleotides marked in black have 2′-OMe ribose sugars and phosphorothioate internucleoside linkages. Nucleotides marked in blue-bold-underlined are 2′-MOE tetrahydropyran-4-yl phosphothiotriesters.

Oligonucleotide	Sequence (5′→3′)	DNA target Tm (ΔTm)	RNA target Tm (ΔTm)
**MOE$_2$**	CCU CU**T** ACC UCA GU**T** ACA	44.0 (-4.5)	58.1 (-3.2)
**MOE$_4$**	CC**T** CU**T** ACC **T**CA GU**T** ACA	41.5 (-6.8)	55.8 (-5.5)
**MOE$_6$**	CC**T** C**TT** ACC **T**CA G**TT** ACA	37.7 (-10.8)	54.6 (-6.5)

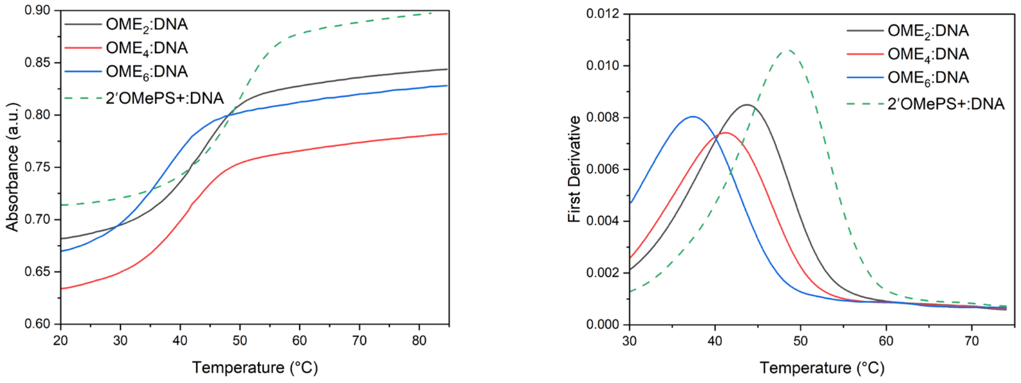

Figure S102: UV melting studies of **MOE$_2$, MOE$_4$ and MOE$_6$** oligonucleotides with complementary DNA. *Left:* Representative UV melting curves measured using 2 nmol of each oligonucleotide (final concentration 2 µM) in 10 mM Na-phosphate buffer, 100 mM NaCl, pH = 7.0; *Right:* 1st derivative of melting curves.

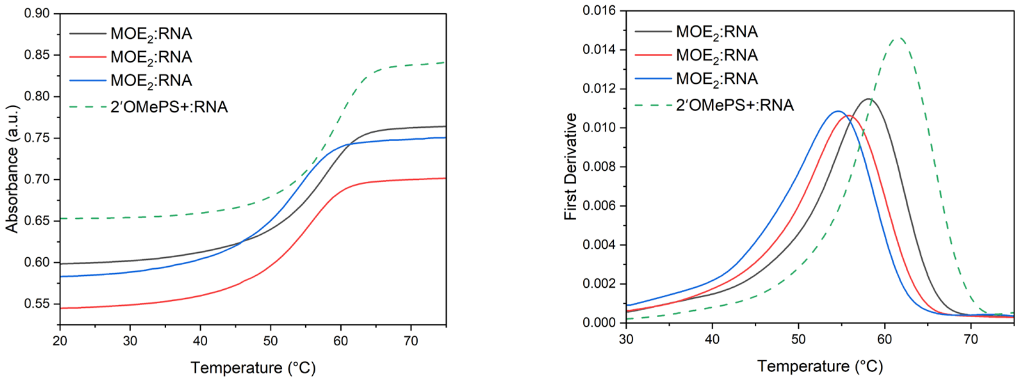

Figure S103: UV melting studies of **MOE$_2$, MOE$_4$ and MOE$_6$** oligonucleotides with complementary RNA. *Left:* Representative UV melting curves measured using 2 nmol of each oligonucleotide (final concentration 2 µM) in 10 mM Na-phosphate buffer, 25 mM NaCl, pH = 7.0; *Right:* 1st derivative of melting curves.

Table T5: Duplex melting temperatures (Tm) in °C of **LNA2-OMe2, LNA4-OMe4 and LNA7-OMe6** oligonucleotides with complementary DNA and RNA. Nucleotides marked in black have 2′-OMe ribose sugars and phosphorothioate internucleoside linkages. Nucleotides marked in black-bold-underlined are 2′-OMe tetrahydropyran-4-yl phosphothiotriesters. Nucleotides marked in red-bold-underlined are LNA tetrahydropyran-4-yl phosphothiotriesters.

Oligonucleotide	Sequence (5′→3′)	DNA target Tm (ΔTm)	RNA target Tm (ΔTm)
**LNA2-OMe2**	C**C**U CU**T** ACC U**C**A GU**T** ACA	50.1 (+1.6)	63.0 (+1.7)
**LNA4-OMe4**	C**CT** CU**T** A**C**C **TC**A GU**T** A**C**A	45.9 (-2.6)	61.5 (+0.2)
**LNA7-OMe6**	**CCT** **CTT** A**CC** **TC**A G**TT** A**C**A	37.2 (-11.1)	60.3 (-1.0)

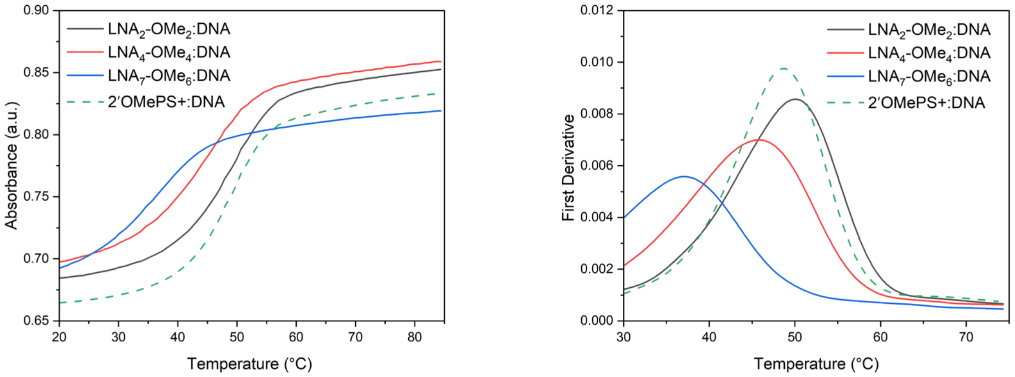

Figure S104: UV melting studies of **LNA2-OMe2, LNA4-OMe4 and LNA7-OMe6** oligonucleotides with complementary DNA. *Left:* Representative UV melting curves measured using 2 nmol of each oligonucleotide (final concentration 2 µM) in 10 mM Na-phosphate buffer, 100 mM NaCl, pH = 7.0; *Right:*1st derivative of melting curves.

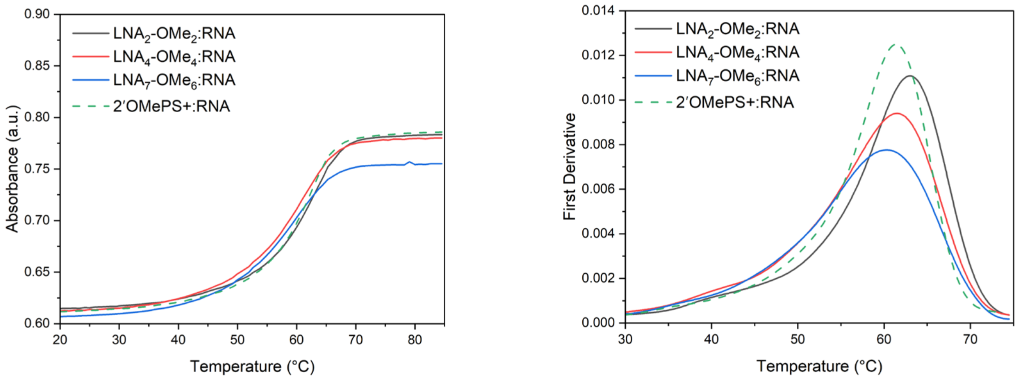

Figure S105: UV melting studies of **LNA2-OMe2, LNA4-OMe4 and LNA7-OMe6** oligonucleotides with complementary RNA. *Left:* Representative UV melting curves measured using 2 nmol of each oligonucleotide (final concentration 2 µM) in 10 mM Na-phosphate buffer, 25 mM NaCl, pH = 7.0; *Right:* 1st derivative of melting curves.

Table T6: Duplex melting temperatures (Tm) in °C of **LNA2-MOE2, LNA4-MOE4 and LNA7-MOE6** oligonucleotides with complementary DNA and RNA. Nucleotides marked in black have 2´-OMe ribose sugars and phosphorothioate internucleoside linkages. Nucleotides marked in blue have 2´-MOE ribose sugars and phosphorothioate internucleoside linkages. Nucleotides marked in blue-bold-underlined are 2´-MOE tetrahydropyran-4-yl phosphothiotriesters. Nucleotides marked in red-bold-underlined are LNA tetrahydropyran-4-yl phosphothiotriesters.

Oligonucleotide	Sequence (5´→3´)	DNA target Tm (ΔTm)	RNA target Tm (ΔTm)
**2´MOEPS+**	CCT CTT ACC TCA GTT ACA	56.0 (7.5)	67.9 (6.6)
**LNA2-MOE2**	C**C**T CT**T** ACC T**C**A GT**T** ACA	56.4 (7.9)	69.5 (8.2)
**LNA4-MOE4**	C**CT** CT**T** A**C**C **TC**A GT**T** A**C**A	50.2 (1.7)	64.4 (3.1)
**LNA7-MOE6**	**CCT** **CTT** A**CC** **TC**A G**TT** A**C**A	38.0 (-10.5)	59.0 (-2.3)

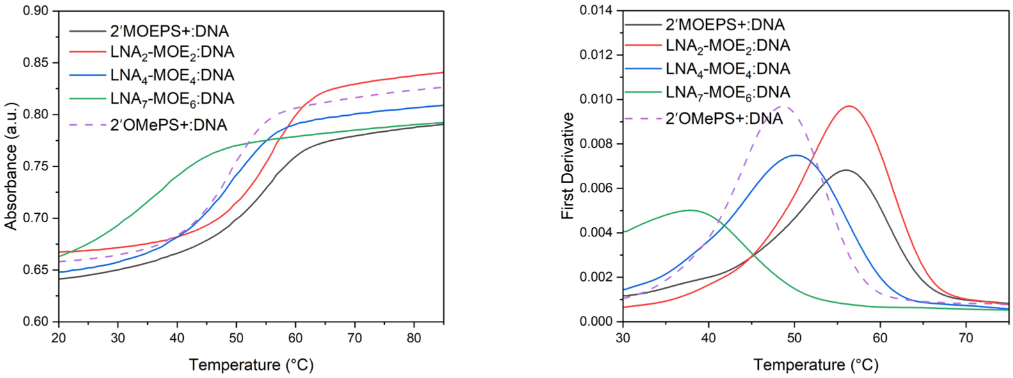

Figure S106: UV melting studies of **LNA2-MOE2, LNA4-MOE4 and LNA7-MOE6** oligonucleotides with complementary DNA. *Left:* Representative UV melting curves measured using 2 nmol of each oligonucleotide (final concentration 2 µM) in 10 mM Na-phosphate buffer, 100 mM NaCl, pH = 7.0; *Right:* 1st derivative of melting curves.

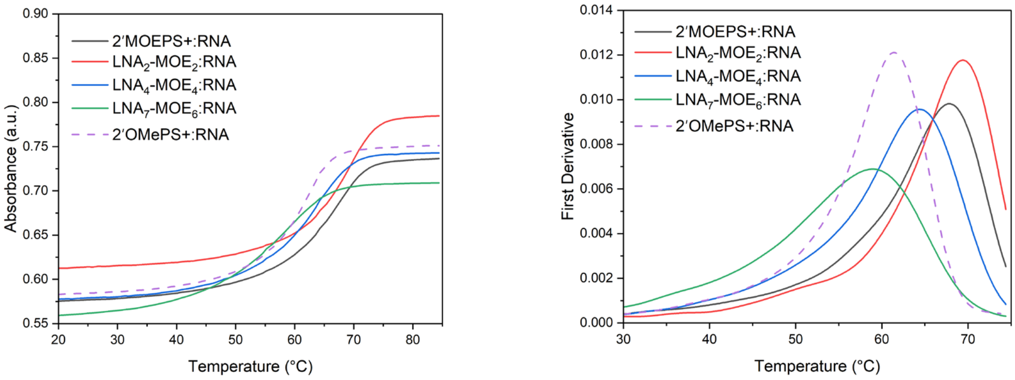

Figure S107: UV melting studies of **LNA2-MOE2, LNA4-MOE4 and LNA7-MOE6** oligonucleotides with complementary RNA. *Left:* Representative UV melting curves measured using 2 nmol of each oligonucleotide (final concentration 2 µM) in 10 mM Na-phosphate buffer, 25 mM NaCl, pH = 7.0; *Right:* 1st derivative of melting curves.

Table T7: Duplex melting temperatures (Tm) in °C of **F2, F4 and F6** oligonucleotides with complementary DNA and RNA. Nucleotides marked in black have 2´-OMe ribose sugars and phosphorothioate internucleoside linkages. Nucleotides marked in purple-bold-underlined are 2´-F tetrahydropyran-4-yl phosphothiotriesters.

**Oligonucleotide**	**Sequence (5´→3´)**	**DNA target Tm (ΔTm)**	**RNA target Tm (ΔTm)**
**F2**	CCU CU**T** ACC UCA GU**T** ACA	43.8 (-4.7)	58.2 (-2.9)
**F4**	CC**T** CU**T** ACC **T**CA GU**T** ACA	40.7 (-7.8)	56.0 (-5.3)
**F6**	CC**T** C**TT** ACC **T**CA G**TT** ACA	33.8 (-14.7)	52.2 (-9.1)

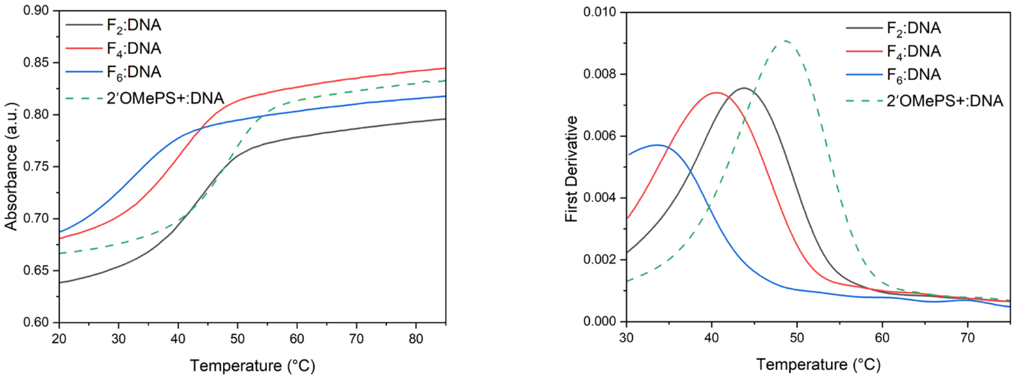

Figure S108: UV melting studies of **F2, F4 and F6** oligonucleotides with complementary DNA. *Left:* Representative UV melting curves measured using 2 nmol of each oligonucleotide (final concentration 2 µM) in 10 mM Na-phosphate buffer, 100 mM NaCl, pH = 7.0; *Right:* 1st derivative of melting curves.

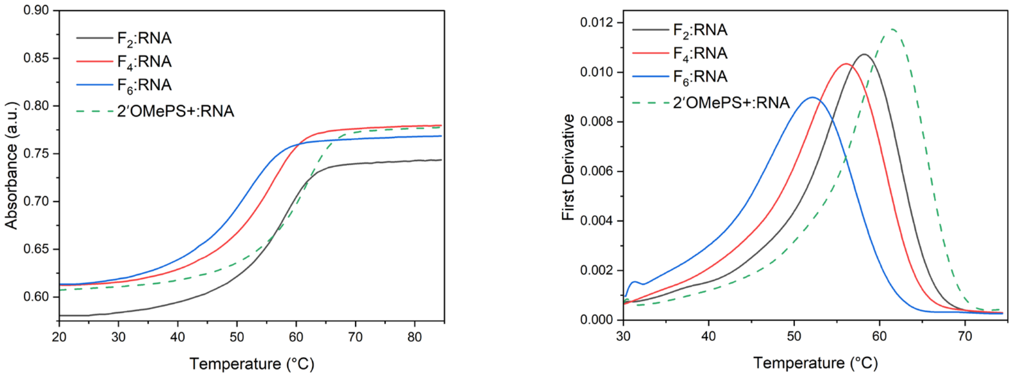

Figure S109: UV melting studies of **F2, F4 and F6** oligonucleotides with complementary RNA. *Left:* Representative UV melting curves measured using 2 nmol of each oligonucleotide (final concentration 2 µM) in 10 mM Na-phosphate buffer, 25 mM NaCl, pH = 7.0; *Right:* 1st derivative of melting curves.

Table T8: Duplex melting temperatures (Tm) in °C of **LNA$_2$-F$_2$, LNA$_2$-F$_4$ LNA$_3$-F$_3$ and LNA$_4$-F$_2$** oligonucleotides with complementary DNA and RNA. Nucleotides marked in black have 2´-OMe ribose sugars and phosphorothioate internucleoside linkages. Nucleotides marked in red-bold-underlined are LNA tetrahydropyran-4-yl phosphothiotriesters. Nucleotides marked in purple-bold-underlined are 2´-F tetrahydropyran-4-yl phosphothiotriesters.

Oligonucleotide	Sequence (5´→3´)	DNA target Tm (ΔTm)	RNA target Tm (ΔTm)
**LNA$_2$-F$_2$**	CC**T** CU**T** ACC **T**CA GU**T** ACA	52.0 (+3.5)	65.7 (+4.2)
**LNA$_2$-F$_4$**	CC**T** C**TT** ACC **T**CA G**TT** ACA	47.6 (-0.9)	63.0 (+1.7)
**LNA$_3$-F$_3$**	CC**T** C**TT** ACC **T**CA G**TT** ACA	52.6 (+4.1)	67.2 (+5.9)
**LNA$_4$-F$_2$**	CC**T** C**TT** ACC **T**CA G**TT** ACA	57.4 (+8.9)	70.6 (+9.3)

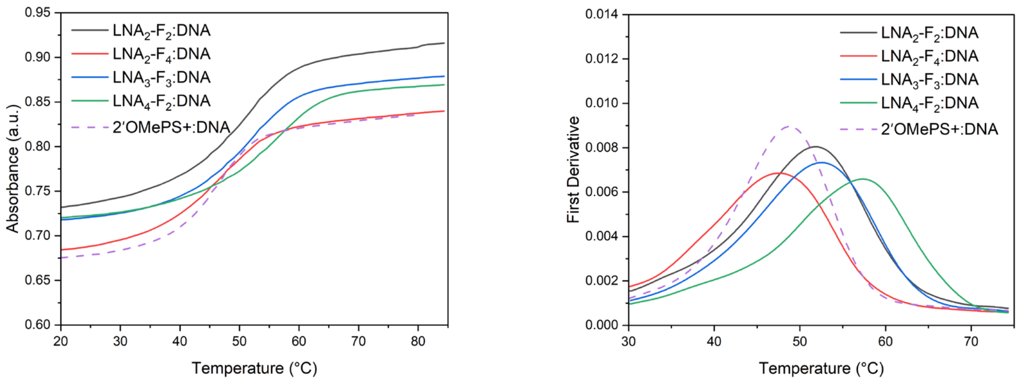

Figure S110: UV melting studies of **LNA$_2$-F$_2$, LNA$_2$-F$_4$ LNA$_3$-F$_3$ and LNA$_4$-F$_2$** oligonucleotides with complementary DNA. *Left:* Representative UV melting curves measured using 2 nmol of each oligonucleotide (final concentration 2 µM) in 10 mM Na-phosphate buffer, 100 mM NaCl, pH = 7.0; *Right:* 1st derivative of melting curves.

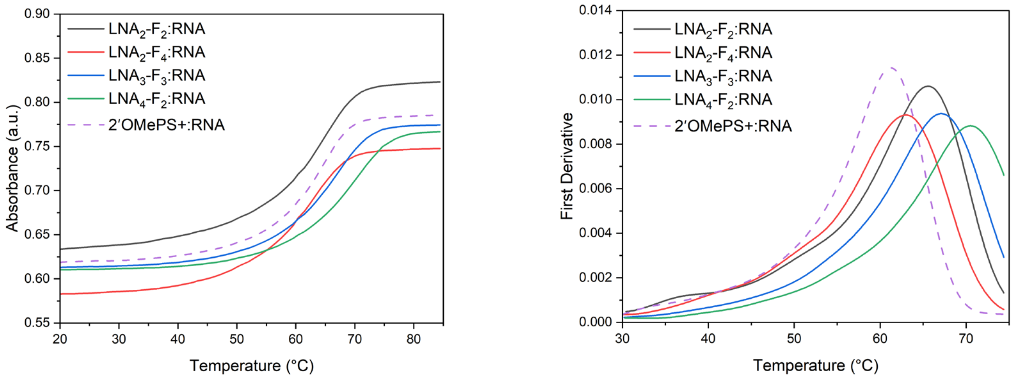

Figure S111: UV melting studies of **LNA$_2$-F$_2$, LNA$_2$-F$_4$ LNA$_3$-F$_3$ and LNA$_4$-F$_2$** oligonucleotides with complementary RNA. *Left:* Representative UV melting curves measured using 2 nmol of each oligonucleotide (final concentration 2 µM) in 10 mM Na-phosphate buffer, 25 mM NaCl, pH = 7.0; *Right:* 1st derivative of melting curves.

Table T9: Duplex melting temperatures (Tm) in °C of **Pip-LNA2, Pip-LNA4 and Pip-LNA6** oligonucleotides with complementary DNA and RNA. Nucleotides marked in black have 2´-OMe ribose sugars and phosphorothioate internucleoside linkages. Nucleotides marked in red-bold-lowercase are LNA piperid-4-yl phosphothiotriesters. *0 mM NaCl.

Oligonucleotide	Sequence (5´→3´)	DNA target Tm (ΔTm)	RNA target Tm (ΔTm)
**Pip-LNA2**	CCU CU**t** ACC UCA GU**t** ACA	55.0 (+6.5)	68.5 (+7.2)
**Pip-LNA4**	CC**t** CU**t** ACC **t**CA GU**t** ACA	61.5 (+12.0)	74.4, 68.6* (+11.1)
**Pip-LNA6**	CC**t** C**tt** ACC **t**CA G**tt** ACA	67.2 (+18.7)	Nd (>+15.0)

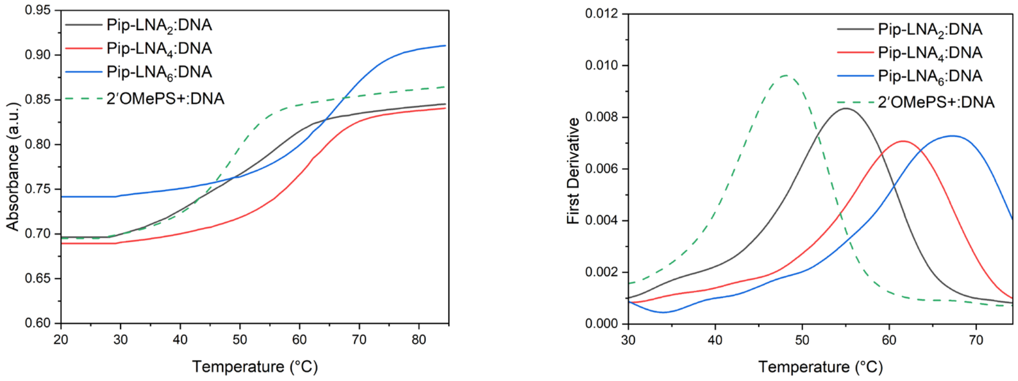

Figure S112: UV melting studies of **Pip-LNA2, Pip-LNA4 and Pip-LNA6** oligonucleotides with complementary DNA. *Left:* Representative UV melting curves measured using 2 nmol of each oligonucleotide (final concentration 2 µM) in 10 mM Na-phosphate buffer, 100 mM NaCl, pH = 7.0; *Right:* 1st derivative of melting curves.

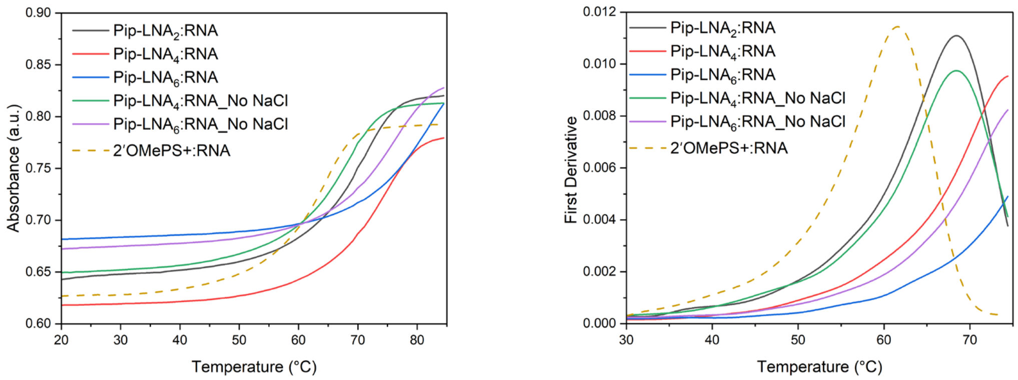

Figure S113: UV melting studies of **Pip-LNA2, Pip-LNA4 and Pip-LNA6** oligonucleotides with complementary RNA. *Left:* Representative UV melting curves measured using 2 nmol of each oligonucleotide (final concentration 2 µM) in 10 mM Na-phosphate buffer, 25 mM NaCl, pH = 7.0; *Right:* 1st derivative of melting curves.

Table T10: Duplex melting temperatures (Tm) in °C of **Pip-F2, Pip-F4 and Pip-F6** oligonucleotides with complementary DNA and RNA. Nucleotides marked in black have 2´-OMe ribose sugars and phosphorothioate internucleoside linkages. Nucleotides marked in purple-bold-lowercase are 2´-F piperid-4-yl phosphothiotriesters.

Oligonucleotide	Sequence (5´→3´)	DNA target Tm (ΔTm)	RNA target Tm (ΔTm)
**Pip-F2**	CCU CU**t** ACC UCA GU**t** ACA	44.2 (-4.1)	58.5 (-2.8)
**Pip-F4**	CC**t** CU**t** ACC **t**CA GU**t** ACA	41.5 (-6.8)	57.6 (-3.7)
**Pip-F6**	CC**t** C**tt** ACC **t**CA G**tt** ACA	35.2 (-13.1)	55.0 (-6.3)

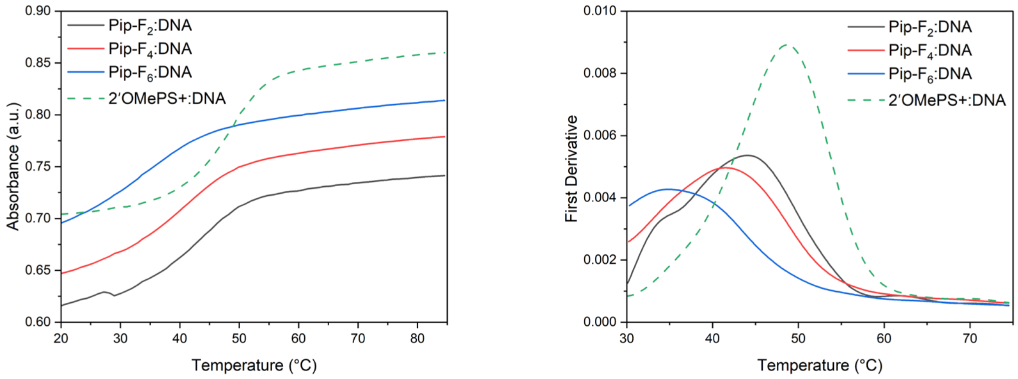

Figure S114: UV melting studies of **Pip-F2, Pip-F4 and Pip-F6** oligonucleotides with complementary DNA. *Left:* Representative UV melting curves measured using 2 nmol of each oligonucleotide (final concentration 2 μM) in 10 mM Na-phosphate buffer, 100 mM NaCl, pH = 7.0; *Right:*1st derivative of melting curves.

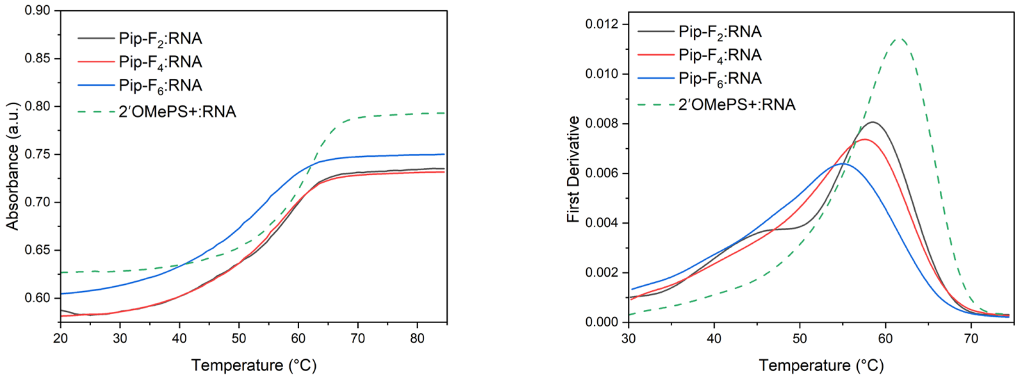

Figure S115: UV melting studies of **Pip-F2, Pip-F4 and Pip-F6** oligonucleotides with complementary RNA. *Left:* Representative UV melting curves measured using 2 nmol of each oligonucleotide (final concentration 2 μM) in 10 mM Na-phosphate buffer, 25 mM NaCl, pH = 7.0; *Right:* 1st derivative of melting curves.

Table T11: Duplex melting temperatures (Tm) in °C of phosphotriester **LNA11-OMe6-PO** oligonucleotide with complementary DNA and RNA. Nucleotides marked in black have 2´-OMe ribose sugars and phosphorothioate internucleoside linkages. Nucleotides marked in red-bold-underlined are LNA tetrahydropyran-4-yl phosphothiotriesters. Nucleotides marked in black-bold-underlined are 2´-OMe tetrahydropyran-4-yl phosphothiotriesters.

Oligonucleotide	Sequence (5´→3´)	DNA target Tm (ΔTm)	RNA target Tm (ΔTm)
**LNA11-OMe6-PO**	$C_oC_oT_oC_oT_oT_oA_oC_oC_oT_oC_oA_oG_oT_oT_oA_oC_oA$	62.7 (+14.2)	Nd

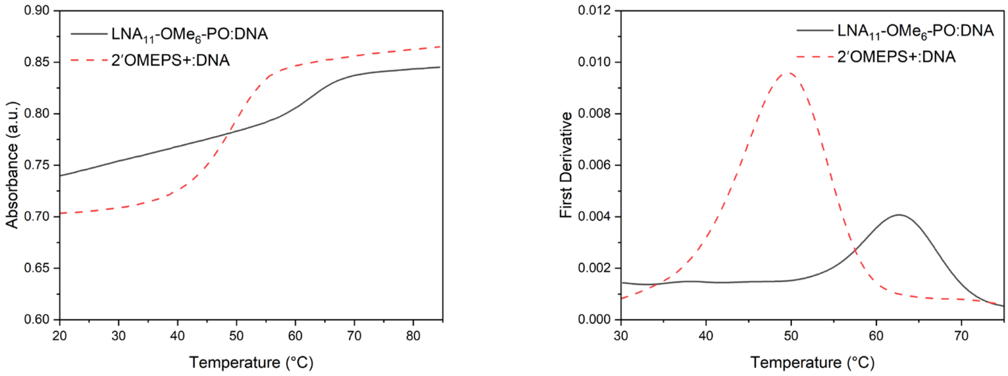

Figure S116: UV melting studies of **LNA11-OMe6-PO** oligonucleotide with complementary DNA. *Left:* Representative UV melting curves measured using 2 nmol of each oligonucleotide (final concentration 2 µM) in 10 mM Na-phosphate buffer, 100 mM NaCl, pH = 7.0; *Right:* 1st derivative of melting curves.

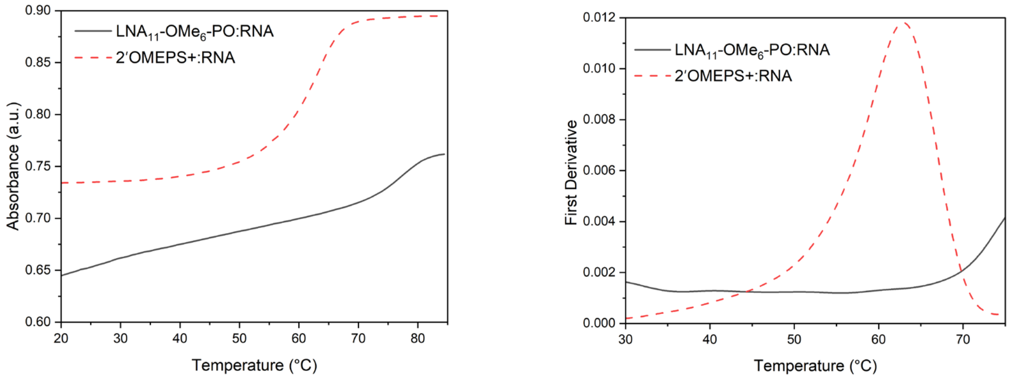

Figure S117: UV melting studies of **LNA11-OMe6-PO** oligonucleotide with complementary RNA. *Left:* Representative UV melting curves measured using 2 nmol of each oligonucleotide (final concentration 2 µM) in 10 mM Na-phosphate buffer, 0 mM NaCl, pH = 7.0; *Right:* 1st derivative of melting curves.

Table T12: Duplex melting temperatures (Tm) in °C of **Pip-OMe$_4$, Pal-MOE$_4$, Pal-LNA$_4$, Pal-LNA$_4$-PS and Pal$_2$-LNA$_4$** oligonucleotides with complementary DNA and RNA. Nucleotides marked in black have 2´-OMe ribose sugars and phosphorothioate internucleoside linkages. Nucleotides marked in red have LNA sugars and phosphorothioate internucleoside linkages. Nucleotides marked in black-bold-underlined are 2´-OMe tetrahydropyran-4-yl phosphothiotriesters. Nucleotides marked in blue-bold-underlined are 2´-MOE tetrahydropyran-4-yl phosphothiotriesters. Nucleotides marked in red-bold-underlined are LNA tetrahydropyran-4-yl phosphothiotriesters.

Oligonucleotide	Sequence (5´→3´)	DNA target Tm (ΔTm)	RNA target Tm (ΔTm)
**Pal-OMe$_4$**	Pal-CC**T** CU**T** ACC **T**CA GU**T** ACA	36.8 (-11.7_	53.4
**Pal-MOE$_4$**	Pal-CC**T** CU**T** ACC **T**CA GU**T** ACA	37.2 (-11.3)	53.0
**Pal-LNA$_4$**	Pal-CC**T** CU**T** ACC **T**CA GU**T** ACA	57.4 (8.9)	69.5 (8.2)
**Pal-LNA$_4$-PS**	Pal-CCT CUT ACC TCA GUT ACA	65.9 (17.4)	>75.0 (Nd)
**Pal$_2$-LNA$_4$**	Pal$_2$-CC**T** CU**T** ACC **T**CA GU**T** ACA	57.8 (9.4)	69.3 (8.0)

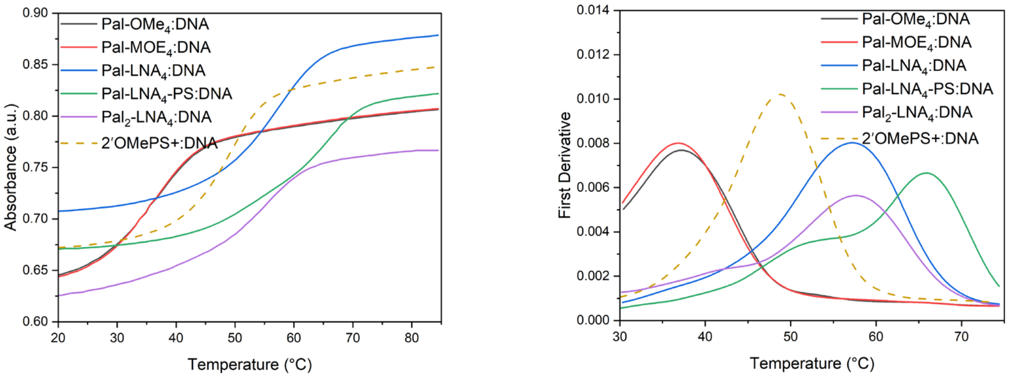

Figure S118: UV melting studies of **Pip-OMe$_4$, Pal-MOE$_4$, Pal-LNA$_4$, Pal-LNA$_4$-PS and Pal$_2$-LNA$_4$** oligonucleotides with complementary DNA. *Left:* Representative UV melting curves measured using 2 nmol of each oligonucleotide (final concentration 2 µM) in 10 mM Na-phosphate buffer, 100 mM NaCl, pH = 7.0; *Right:* 1st derivative of melting curves.

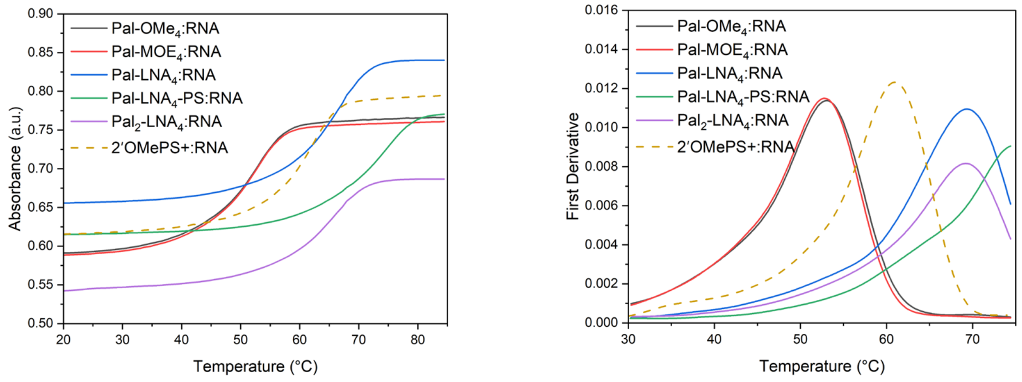

Figure S119: UV melting studies of **Pip-OMe$_4$, Pal-MOE$_4$, Pal-LNA$_4$, Pal-LNA$_4$-PS and Pal$_2$-LNA$_4$** oligonucleotides with complementary RNA. *Left:* Representative UV melting curves measured using 2 nmol of each oligonucleotide (final concentration 2 µM) in 10 mM Na-phosphate buffer, 25 mM NaCl, pH = 7.0; *Right:* 1st derivative of melting curves.

Table T13: Duplex melting temperatures (Tm) in °C of **Pal-Pip-LNA4 and Pal-Pip-F4** oligonucleotides with complementary DNA and RNA. Nucleotides marked in black have 2´-OMe ribose sugars and phosphorothioate internucleoside linkages. Nucleotides marked in purple-bold-lowercase are 2´-F piperid-4-yl phosphothiotriesters. Nucleotides marked in Red-bold-lowercase are LNA piperid-4-yl phosphothiotriesters.

Oligonucleotide	Sequence (5´→3´)	DNA target Tm (ΔTm)	RNA target Tm (ΔTm)
**Pal-Pip-LNA4**	Pal-CC**t** CU**t** ACC **t**CA GU**t** ACA	60.0 (+11.5)	73.2 (+11.9)
**Pal-Pip-F4**	Pal-CC**t** CU**t** ACC **t**CA GU**t** ACA	37.2 (-11.2)	54.4 (-6.9)

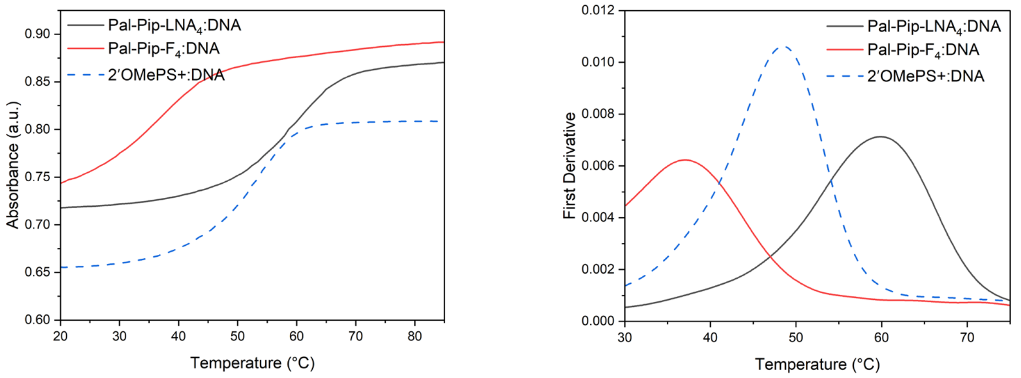

Figure S120: UV melting studies of **Pal-Pip-LNA4 and Pal-Pip-F4** oligonucleotides with complementary DNA. *Left:* Representative UV melting curves measured using 2 nmol of each oligonucleotide (final concentration 2 µM) in 10 mM Na-phosphate buffer, 100 mM NaCl, pH = 7.0; *Right:*1st derivative of melting curves.

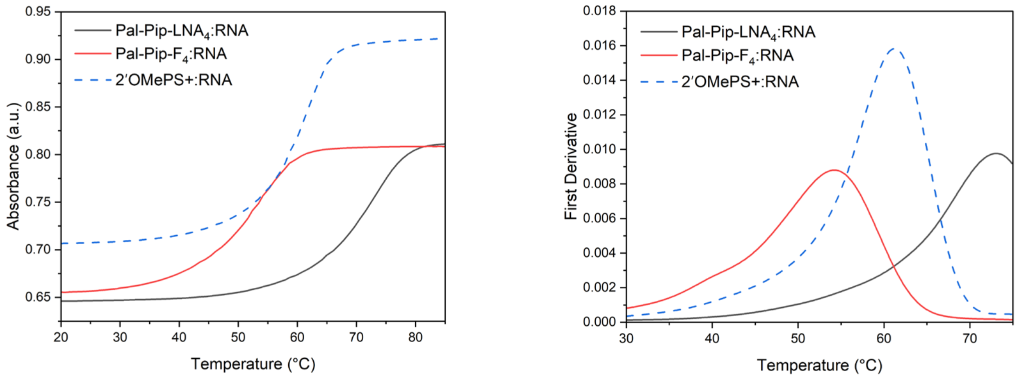

Figure S121: UV melting studies of **Pal-Pip-LNA4 and Pal-Pip-F4** oligonucleotides with complementary RNA. *Left:* Representative UV melting curves measured using 2 nmol of each oligonucleotide (final concentration 2 µM) in 10 mM Na-phosphate buffer, 25 mM NaCl, pH = 7.0; *Right:* 1st derivative of melting curves.

Table T14: Duplex melting temperatures (Tm) in °C of **Pal-SS, C16-SS, Pal-HEG, Pal-HEG-PO and +Control-Pal and +pal-Control-Pal$_2$** oligonucleotides with complementary DNA and RNA. Nucleotides marked in black have 2´-OMe ribose sugars and phosphorothioate internucleoside linkages.

Oligonucleotide	Sequence (5´→3´)	DNA target Tm (ΔTm)	RNA target Tm (ΔTm)
**Pal-SS**	Pal-SS-CCU CUU ACC UCA GUU ACA	48.3 (-0.2)	61.0 (-0.3)
**C16-SS**	C16-SS-CCU CUU ACC UCA GUU ACA	48.3 (-0.2)	61.2 (-0.1)
**Pal-HEG**	Pal-HEG-CCU CAU UCA CUC GAU UCA	48.3 (-0.2)	60.8 (-0.5)
**Pal-HEG-PO**	Pal$_o$-HEG$_o$-CCU CAU UCA CUC GAU UCA	48.5 (0.0)	60.6 (-0.7)
**+Control-Pal**	Pal-CCU CUU ACC UCA GUU ACA	48.2 (-0.3)	61.2 (-0.1)
**+Control-Pal$_2$**	Pal$_2$-CCU CAU UCA CUC GAU UCA	48.8 (0.3)	61.2 (-0.1)

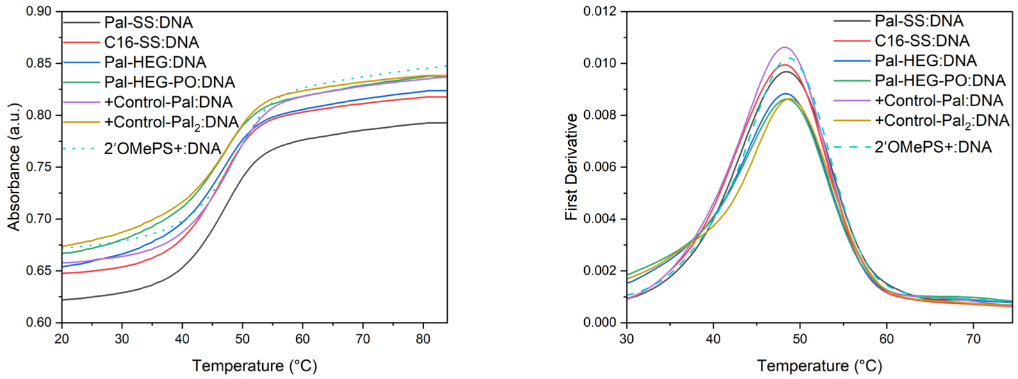

Figure S122: UV melting studies of **Pal-SS, C16-SS, Pal-HEG, Pal-HEG-PO and +Control-Pal and +pal-Control-Pal$_2$** oligonucleotides with complementary DNA. *Left:* Representative UV melting curves measured using 2 nmol of each oligonucleotide (final concentration 2 µM) in 10 mM Na-phosphate buffer, 100 mM NaCl, pH = 7.0; *Right:*1st derivative of melting curves.

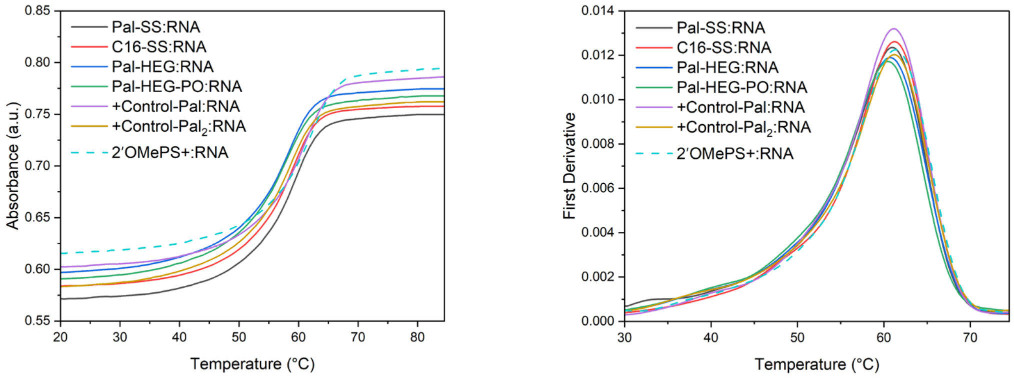

Figure S123: UV melting studies of **Pal-SS, C16-SS, Pal-HEG, Pal-HEG-PO and +Control-Pal and +pal-Control-Pal$_2$** oligonucleotides with complementary RNA. *Left:* Representative UV melting curves measured using 2 nmol of each oligonucleotide (final concentration 2 µM) in 10 mM Na-phosphate buffer, 25 mM NaCl, pH = 7.0; *Right:* 1st derivative of melting curves.

Table T15: Duplex melting temperatures (Tm) in °C of **Pal-LNA$_2$-OMe$_2$, Pal-LNA$_2$(C)-PS, Pal-LNA$_2$-F$_2$, Pal-LNA$_2$-F$_2$-PS, Pal-Pip-LNA$_2$, Pal-LNA$_2$-PS, Pal-F$_2$-PS, Pal-MOE$_2$-PS and Pal-2´MOEPS+** oligonucleotides with complementary DNA and RNA. Nucleoside in black have 2´-OMe sugars and phosphorothioate internucleoside linkages. Nucleoside in blue have 2´-MOE sugars and phosphorothioate internucleoside linkages. Nucleosides in red are locked nucleic acids and phosphorothioate internucleoside linkages. Nucleosides in purple have 2´-F sugars and phosphorothioate internucleoside linkages. Nucleosides in bold and under-lined are tetrahydropyran-4-yl phosphothiotriesters. Nucleosides in red lowercase are LNA-piperid-4-yl phosphothiotriesters. The LNA-C bases in Pal-LNA2(C)-PS are methylated at position 5.

Oligonucleotide	Sequence (5´→3´)	DNA target Tm (ΔTm)	RNA target Tm (ΔTm)
**Pal-LNA$_2$-OMe$_2$**	Pal-C**C**U CU**T** ACC U**C**A GU**T** ACA	47.0 (-1.5)	60.7 (-0.6)
**Pal-LNA$_2$(C)-PS**	Pal-CCU CUU ACC UCA GUU ACA	59.0 (+10.5)	68.3 (+7.0)
**Pal-LNA$_2$-F$_2$**	Pal-CC**T** CU**T** ACC **T**CA GU**T** ACA	48.6 (+0.1)	62.3 (+1.0)
**Pal-LNA$_2$-F$_2$-PS**	Pal-CCT CUT ACC TCA GUT ACA	58.9 (+10.4)	68.7 (+7.4)
**Pal-Pip-LNA$_2$**	Pal-CCU CU**t** ACC UCA GU**t** ACA	55.2 (+6.7)	66.8 (+5.5)
**Pal-LNA$_2$-PS**	Pal-CCU CUT ACC UCA GUT ACA	57.5 (9.0)	68.6 (+7.3)
**Pal-F$_2$-PS**	Pal-CCU CUT ACC UCA GUT ACA	48.5 (+0.0)	60.7 (-0.6)
**Pal-MOE$_2$-PS**	Pal-CCU CUT ACC UCA GUT ACA	47.7 (-0.8)	60.3 (-1.0)
**Pal-2´MOEPS+**	Pal-CCU CUU ACC UCA GUU ACA	55.2 (+6.7)	60.8 (-0.5)

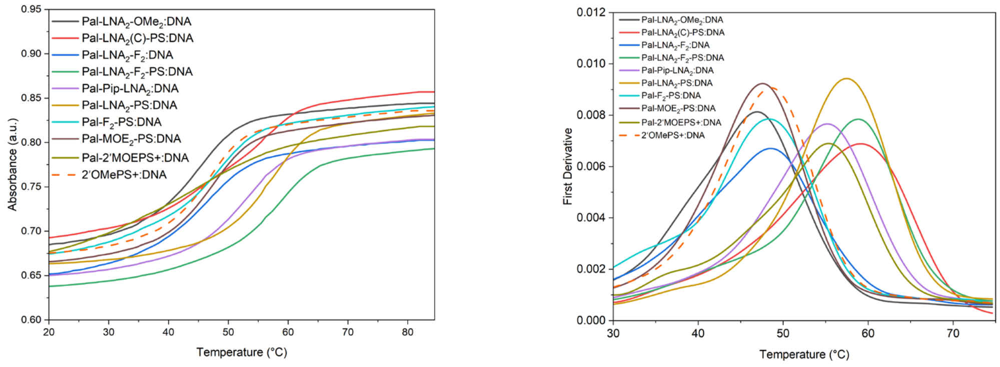

Figure S124: UV melting studies of **Pal-LNA$_2$-OMe$_2$, Pal-LNA$_2$(C)-PS, Pal-LNA$_2$-F$_2$, Pal-LNA$_2$-F$_2$-PS, Pal-Pip-LNA$_2$, Pal-LNA$_2$-PS, Pal-F$_2$-PS, Pal-MOE$_2$-PS and Pal-2´MOEPS+** oligonucleotides with complementary DNA. *Left:* Representative UV melting curves measured using 2 nmol of each oligonucleotide (final concentration 2 μM) in 10 mM Na-phosphate buffer, 100 mM NaCl, pH = 7.0; *Right:* 1st derivative of melting curves.

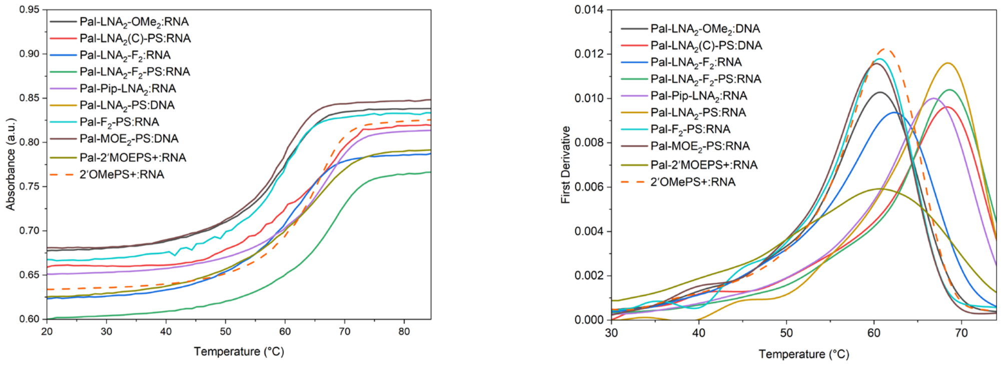

Figure S125: UV melting studies of **Pal-LNA$_2$-OMe$_2$, Pal-LNA$_2$(C)-PS, Pal-LNA$_2$-F$_2$, Pal-LNA$_2$-F$_2$-PS, Pal-Pip-LNA$_2$, Pal-LNA$_2$-PS, Pal-F$_2$-PS, Pal-MOE$_2$-PS and Pal-2´MOEPS+** oligonucleotides with complementary RNA. *Left:* Representative UV melting curves measured using 2 nmol of each oligonucleotide (final concentration 2 μM) in 10 mM Na-phosphate buffer, 25 mM NaCl, pH = 7.0; *Right:* 1st derivative of melting curves.

3.1 Thermal Stability Studies in Acidic pH

Table T16: Duplex melting temperatures (Tm and ΔTm) in °C of **Piperidine PTTE** oligonucleotides with complementary DNA and RNA. Tm values are averages of three experiments with an error of ±0.50 °C. ΔTm = difference in duplex melting temperature against RNA compared to the **2´OMePS+**. Nd: not determined.

[a] Condition; 100 mM NaOAc-AcOH buffer, pH 5.5, 25 mM NaCl
[b] Condition; 100 mM NaOAc-AcOH buffer, pH 5.5, no NaCl
[c] Condition; 50 mM NaOAc-AcOH buffer, pH 5.5, no NaCl
[d] Condition; 10 mM NaOAc-AcOH buffer, pH 5.5, no NaCl

**Oligonucleotides**	**DNA target[a] Tm (ΔTm)**	**RNA target[a] Tm (ΔTm)**	**RNA target[b] Tm (ΔTm)**	**RNA target[c] Tm (ΔTm)**	**RNA target[d] Tm (ΔTm)**
**2´OMePS+**	48.1	69.1	66.4	61.0	53.0
**+Control-Pal**	47.6 (-0.5)	67.6 (-1.5)	65.2 (-1.2)	59.6 (-1.4)	45.6 (-7.4)
**Pip-LNA$_2$**	56.1 (+8.0)	74.3 (+5.2)	72.0 (+5.6)	66.6 (+5.6)	57.5 (+4.5)
**Pip-LNA$_4$**	61.7 (+13.6)	>+75.0 (Nd)	>+75.0 (Nd)	74.0 (+13.0)	64.5 (+11.5)
**Pip-LNA$_6$**	67.5 (+19.4)	>+75.0 (Nd)	>+75.0 (Nd)	>+75.0 (Nd)	72.2 (+19.2)
**Pal-Pip-LNA$_2$**	54.1 (6.0)	73.8 (+4.7)	71.6 (+5.2)	65.2 (+4.2)	54.4 (+1.4)
**Pal-Pip-LNA$_4$**	59.8 (11.7)	>+75.0 (Nd)	>+75.0	72.1 (+11.1)	60.0 (+7.0)
**Pip-F$_2$**	42.8 (-5.3)	65.6 (-3.5)	62.4 (-4.0)	57.0 (-4.0)	46.4 (-6.6)
**Pip-F$_4$**	39.6 (-8.5)	63.4 (-5.7)	59.9 (-6.5)	56.5 (-3.5)	46.3 (-6.7)
**Pip-F$_6$**	34.8 (-13.3)	59.5 (-9.6)	59.0 (-7.4)	54.0 (-7.0)	47.3 (-5.7)
**Pal-Pip-F$_4$**	35.4 (-12.7)	61.2 (-7.9)	58.4 (-8.0)	52.8 (-8.2)	42.0 (-11.0)

4.0 Circular Dichroism Studies

CD spectra were recorded on a Chirascan spectropolarimeter (Applied Photophysics Ltd). Duplexes were prepared by annealing 2 nmol of the modified oligonucleotide with an equimolar amount of complementary DNA or RNA in 100 mM sodium phosphate buffer (pH 7.0), supplemented with 100 mM NaCl for DNA duplexes or 25 mM NaCl for RNA duplexes. Measurements were carried out in 2 mm path-length quartz cuvettes at 25 °C. Spectra were collected over the range of 200–330 nm with a bandwidth of 1 nm, at a scanning speed of 20 nm/min. Each final spectrum represents the average of three consecutive scans.

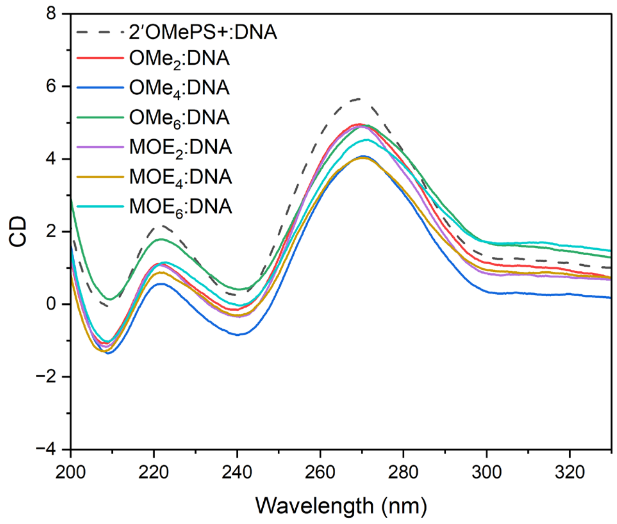

Figure S126: CD spectra of modified oligonucleotides (**OMe$_2$, OMe$_4$, OMe$_6$, MOE$_2$, MOE$_4$, and MOE$_6$**) with complementary DNA duplex in 10 mM Na-Phosphate buffer, 100 mM NaCl, pH 7.0. Y-axis is ellipticity θ, (10^{-3} deg.cm^2/dmol).

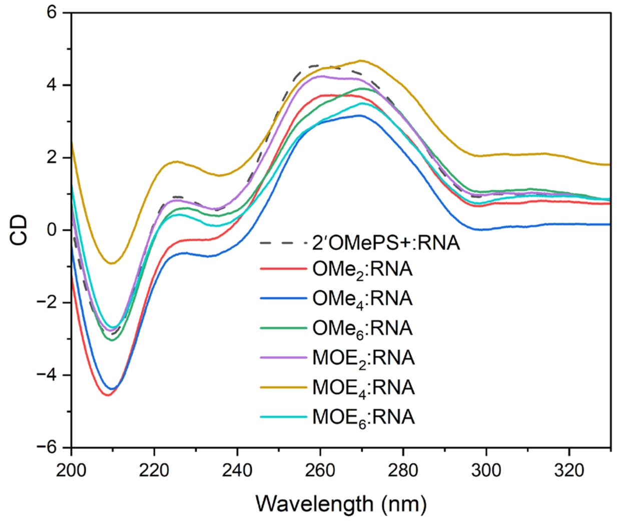

Figure S127: CD spectra of modified oligonucleotides (**OMe$_2$, OMe$_4$, OMe$_6$, MOE$_2$, MOE$_4$, and MOE$_6$**) with complementary RNA duplex in 10 mM Na-Phosphate buffer, 25 mM NaCl, pH 7.0. Y-axis is ellipticity θ, (10^{-3} deg.cm^2/dmol).

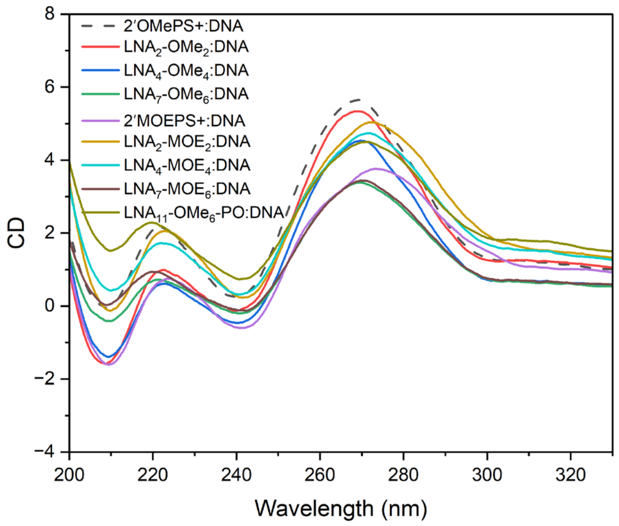

Figure S128: CD spectra of modified oligonucleotides (**LNA$_{2}$-OMe$_{2}$, LNA$_{4}$-OMe$_{4}$, LNA$_{7}$-OMe$_{6}$, 2´MOEPS+, LNA$_{2}$-MOE$_{2}$, LNA$_{4}$-MOE$_{4}$, LNA$_{7}$-MOE$_{6}$ and LNA$_{11}$-OME$_{6}$-PO**) with complementary DNA duplex in 10 mM Na-Phosphate buffer, 100 mM NaCl, pH 7.0. Y-axis is ellipticity θ, (10^{-3} deg.cm^{2}/dmol).

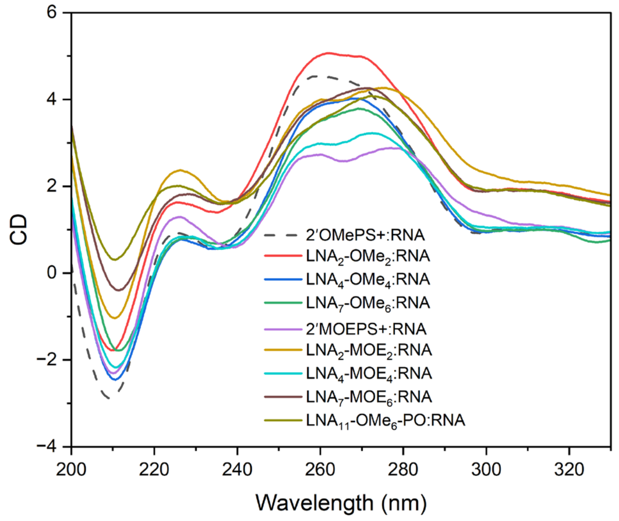

Figure S129: CD spectra of modified oligonucleotides (**LNA$_{2}$-OMe$_{2}$, LNA$_{4}$-OMe$_{4}$, LNA$_{7}$-OMe$_{6}$, 2´MOEPS+, LNA$_{2}$-MOE$_{2}$, LNA$_{4}$-MOE$_{4}$, LNA$_{7}$-MOE$_{6}$ and LNA$_{11}$-OME$_{6}$-PO**) with complementary RNA duplex in 10 mM Na-Phosphate buffer, 25 mM NaCl, pH 7.0. Y-axis is ellipticity θ, (10^{-3} deg.cm^{2}/dmol).

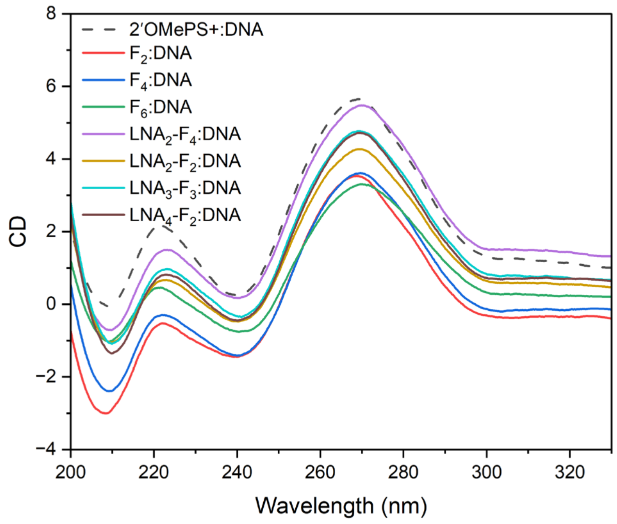

Figure S130: CD spectra of modified oligonucleotides (**F_2, F_4, F_6, LNA$_2$-F_2, LNA$_2$-F_4, LNA$_3$-MOE$_3$ and LNA$_4$-F_2**) with complementary DNA duplex in 10 mM Na-Phosphate buffer, 100 mM NaCl, pH 7.0. Y-axis is ellipticity θ, (10^{-3} deg.cm^2/dmol).

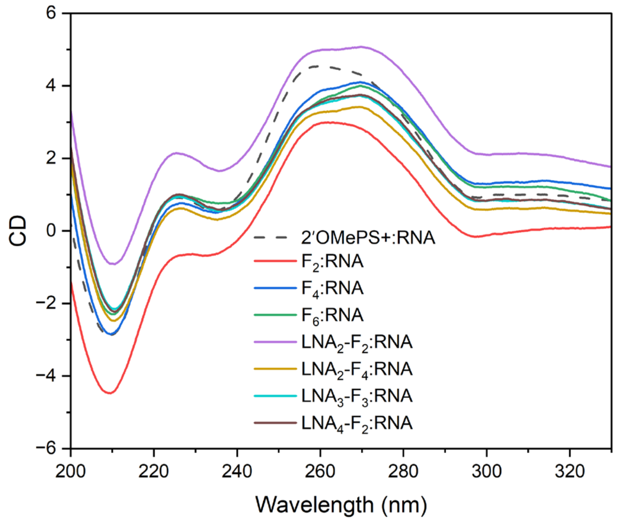

Figure S131: CD spectra of modified oligonucleotides (**F_2, F_4, F_6, LNA$_2$-F_2, LNA$_2$-F_4, LNA$_3$-MOE$_3$ and LNA$_4$-F_2**) with complementary RNA duplex in 10 mM Na-Phosphate buffer, 25 mM NaCl, pH 7.0. Y-axis is ellipticity θ, (10^{-3} deg.cm^2/dmol).

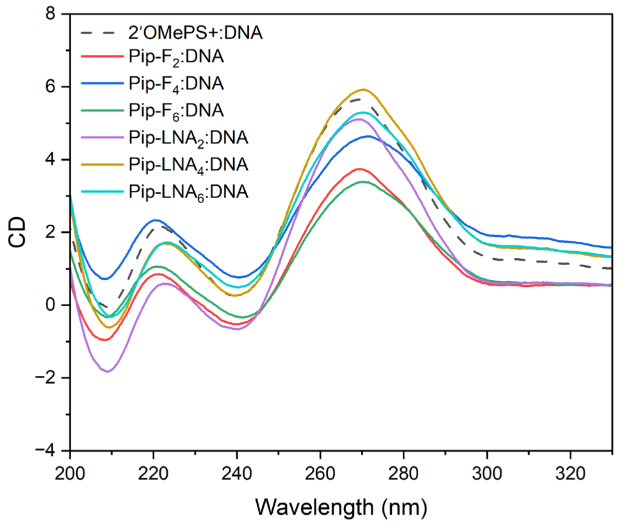

Figure S132: CD spectra of modified oligonucleotides (**Pip-F2, Pip-F4, Pip-F6, Pip-LNA2, Pip-LNA4, and Pip-LNA6**) with complementary DNA duplex in 10 mM Na-Phosphate buffer, 100 mM NaCl, pH 7.0. Y-axis is ellipticity θ, (10^{-3} deg.cm^2/dmol).

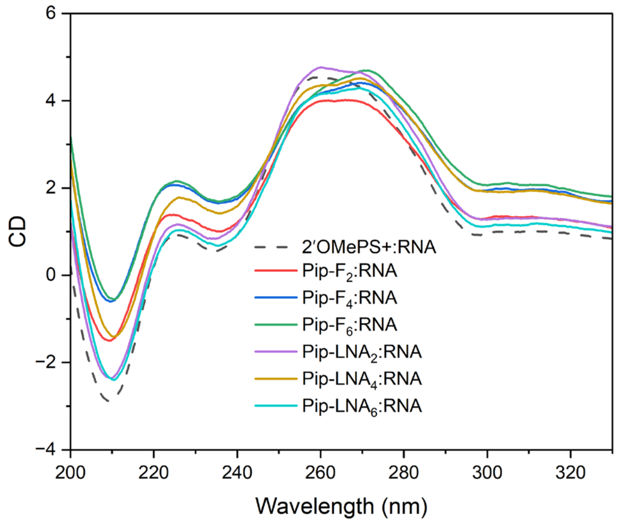

Figure S133: CD spectra of modified oligonucleotides (**Pip-F2, Pip-F4, Pip-F6, Pip-LNA2, Pip-LNA4, and Pip-LNA6**) with complementary RNA duplex in 10 mM Na-Phosphate buffer, 25 mM NaCl, pH 7.0. Y-axis is ellipticity θ, (10^{-3} deg.cm^2/dmol).

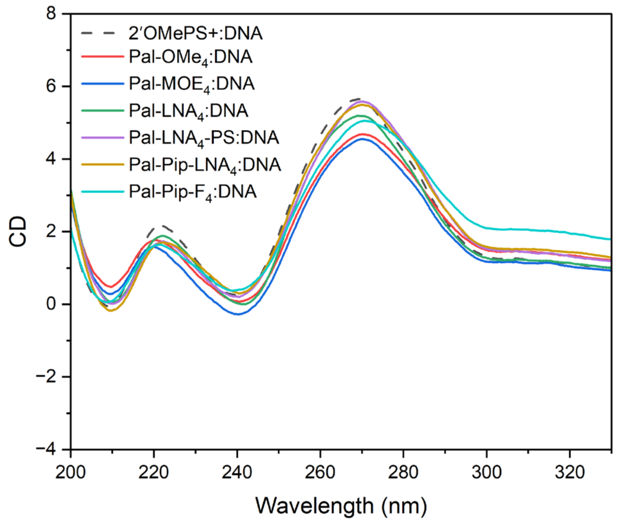

Figure S134: CD spectra of modified oligonucleotides (**Pal-OMe4, Pal-MOE4, Pal-LNA4, Pal-LNA4-PS, Pal-Pip-LNA4 and Pal-Pip-F4**) with complementary DNA duplex in 10 mM Na-Phosphate buffer, 100 mM NaCl, pH 7.0. Y-axis is ellipticity θ, (10^{-3} deg.cm^2/dmol).

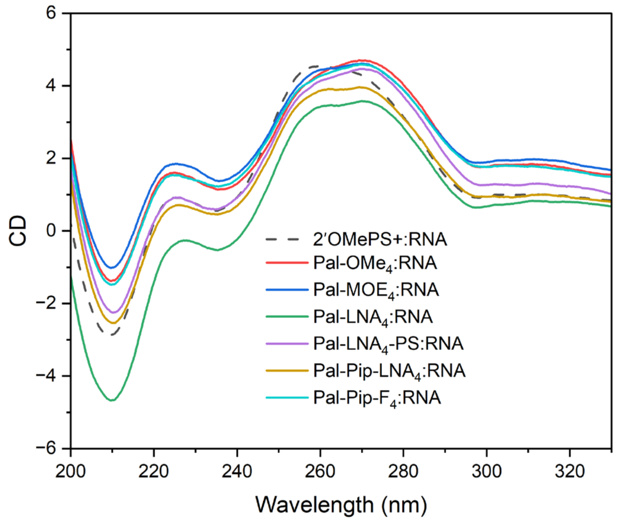

Figure S135: CD spectra of modified oligonucleotides (**Pal-OMe4, Pal-MOE4, Pal-LNA4, Pal-LNA4-PS, Pal-Pip-LNA4 and Pal-Pip-F4**) with complementary RNA duplex in 10 mM Na-Phosphate buffer, 25 mM NaCl, pH 7.0. Y-axis is ellipticity θ, (10^{-3} deg.cm^2/dmol).

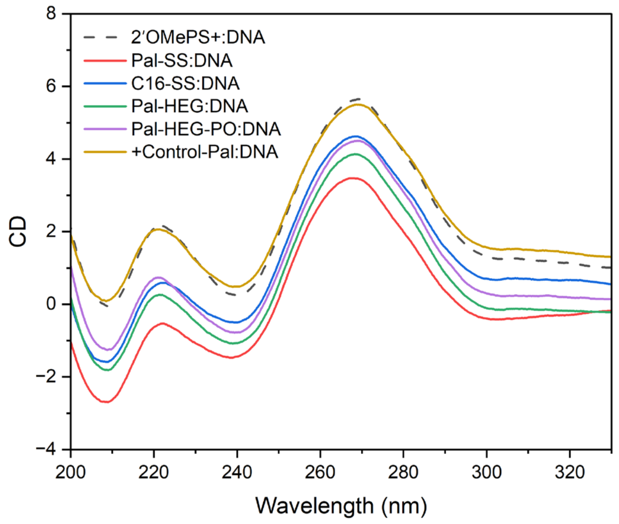

Figure S136: CD spectra of modified oligonucleotides (**Pal-SS, C16-SS, Pal-HEG, Pal-HEG-PO and +Control-Pal**) with complementary DNA duplex in 10 mM Na-Phosphate buffer, 100 mM NaCl, pH 7.0. Y-axis is ellipticity θ, (10^{-3} deg.cm^2/dmol).

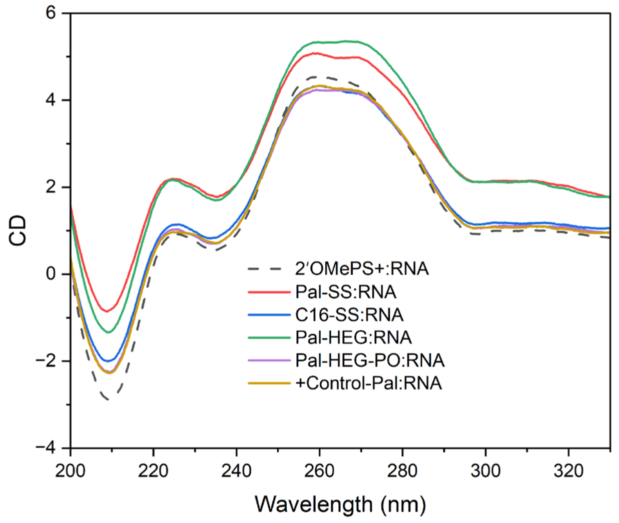

Figure S137: CD spectra of modified oligonucleotides (**Pal-SS, C16-SS, Pal-HEG, Pal-HEG-PO and +Control-Pal**) with complementary RNA duplex in 10 mM Na-Phosphate buffer, 25 mM NaCl, pH 7.0. Y-axis is ellipticity θ, (10^{-3} deg.cm^2/dmol).

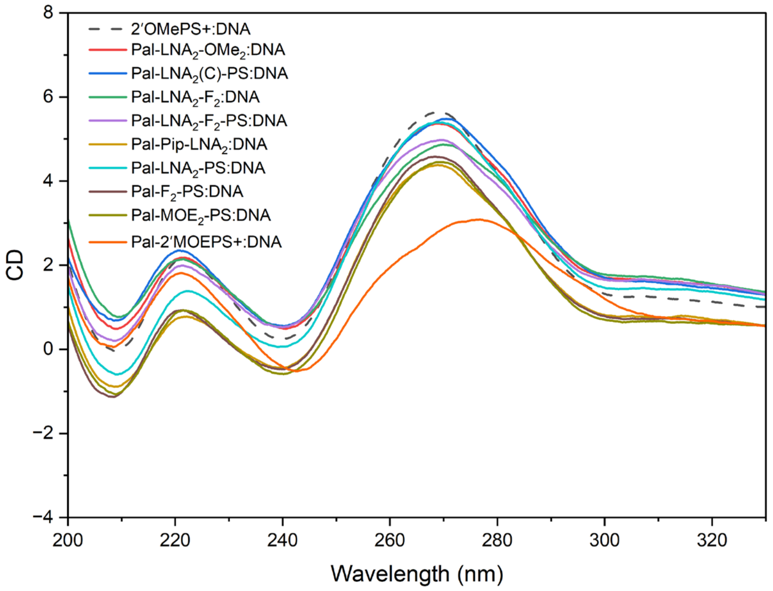

Figure S138: CD spectra of modified oligonucleotides (**Pal-LNA2-OMe2, Pal-LNA2(C)-PS, Pal-LNA2-F2, Pal-LNA2-F2-PS, Pal-Pip-LNA2, Pal-LNA2-PS, Pal-F2-PS, Pal-MOE2-PS and Pal-2´MOEPS+**) with complementary DNA duplex in 10 mM Na-Phosphate buffer, 100 mM NaCl, pH 7.0. Y-axis is ellipticity θ, (10^{-3} deg.cm^2/dmol).

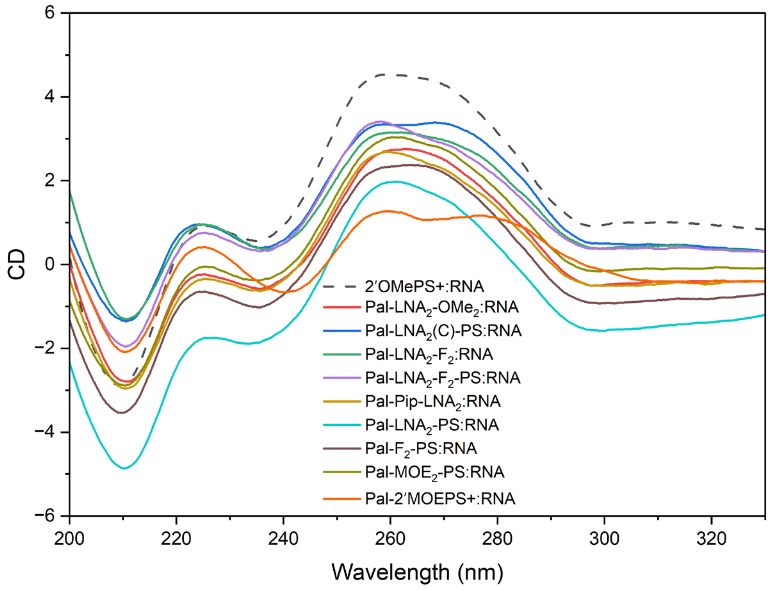

Figure S139: CD spectra of modified oligonucleotides (**Pal-LNA2-OMe2, Pal-LNA2(C)-PS, Pal-LNA2-F2, Pal-LNA2-F2-PS, Pal-Pip-LNA2, Pal-LNA2-PS, Pal-F2-PS, Pal-MOE2-PS and Pal-2´MOEPS+**) with complementary RNA duplex in 10 mM Na-Phosphate buffer, 25 mM NaCl, pH 7.0. Y-axis is ellipticity θ, (10^{-3} deg.cm^2/dmol).

5.0 Biological Experiments

Cell culture: HeLa pLuc/705 cells(2) were maintained in Dulbecco's Modified Eagle Medium (DMEM; Gibco, 31966) supplemented with 10% fetal bovine serum (FBS) and 1X Antibiotic-Antimycotic (Gibco, 15240) at 37 °C and 5% CO_2 in a humidified incubator.

Transfection experiments: HeLa pLuc/705 cells were seeded at a density of 10,000 cells/well in 100 µL DMEM supplemented with 10% FBS and 1X Antibiotic-Antimycotic in a 96-well plate (Greiner, 655098) and incubated for 16 h at 37 °C and 5% CO_2 in a humidified incubator. Oligonucleotides in aqueous solution were diluted to 100 nM in 300 µL Opti-MEM (Gibco, 31985). Separately, 2.00 µL of Lipofectamine 2000 Transfection Reagent (Invitrogen, 11668) were diluted to a final volume of 300 µL in Opti-MEM and incubated for 10 min at room temperature. After 10 min, the diluted oligonucleotide and diluted transfection reagent were mixed and incubated for 20 min at room temperature to allow complex formation. After 20 min, the complexes were serially diluted in Opti-MEM to achieve final oligonucleotide concentrations of 50, 12.5, 3.125, and 0 nM. At 18 h post-seeding, the complete media was aspirated from the cells and replaced with the diluted complexes or Opti-MEM alone (i.e., the 0 nM treatment or untreated cells). The cells were incubated for 4 h at 37 °C and 5% CO_2 in a humidified incubator. At 4 h post-transfection, the complexes were aspirated from the cells and replaced with complete DMEM media with 10% FBS and 1X Antibiotic-Antimycotic, and the cells were incubated for a further 44 h at 37 °C and 5% CO_2 in a humidified incubator.

Gymnosis experiments: Oligonucleotides were diluted to 40 µM in 400 µL Opti-MEM and then serially diluted in Opti-MEM to achieve oligonucleotide concentrations of 40, 20, 10, and 0 µM or 40, 20, 10, 5, 2.5, 1.25, 0.625, and 0 µM. Then, 50 µL of diluted oligonucleotide or Opti-MEM alone (i.e., the 0 µM treatment or untreated cells) and 50 µL HeLa pLuc/705 cells in DMEM supplemented with 6% FBS and 2X Antibiotic-Antimycotic were added to each well of a 96-well plate (Greiner, 655098) to achieve final oligonucleotide concentrations of 20, 10, 5, and 0 µM or 20, 10, 5, 2.5, 1.25, 0.625, 0.3125, and 0 µM and a seeding density of 10,000 cells/well in 100 µL DMEM supplemented with 3% FBS and 1X Antibiotic-Antimycotic. The cells were incubated for 72 h at 37 °C and 5% CO_2 in a humidified incubator.

Luciferase assays

At the end of the transfection or gymnosis experiments described above, the complete media was aspirated from the cells, and the cells were washed with phosphate buffered saline (PBS; Gibco, 10010, 3 x 100 µL). Lysis was performed in 100 µL of Glo Lysis Buffer (Promega, E2661) for 10 min at room temperature with shaking. Then, 50 µL of lysate and 50 µL of Bright-Glo Reagent (Promega, E2620) were added to each well of a 96-well white plate (Greiner, 655075), and after 2 min, luminescence was measured using a CLARIOstar microplate reader (BMG Labtech, software version 5.21.R2). Total protein quantification was carried out using a detergent compatible (DC) Protein Assay (Bio-Rad, 5000111) according to the manufacturer's instructions. Briefly, a bovine serum albumin (BSA) standard (Thermo Scientific, 23209) was prepared at a concentration range of 20-20,000 µg/mL. Then, 5 µL of lysate or BSA standard were treated with 15 µL of Reagent A and 120 µL of Reagent B and incubated for 20 min at room temperature. Absorbance at 750 nm was measured using a

CLARIOstar microplate reader (BMG Labtech, software version 5.21.R2). Total protein quantities were calculated from the measured absorbances using the equation of the linear-fit standard curve in Microsoft Excel.

Both the transfection and gymnosis experiments described above were performed at a minimum in biological triplicate, where each biological replicate was performed in technical triplicate. To calculate the final fold increase over untreated value shown in the plots, the three biological replicates were averaged. Data in the plots are means ± standard deviations for three biological replicates (n = 3). Statistical analyses were performed in GraphPad Prism 10 for macOS Version 10.2.2 (341).

Data presented in this paper, in all cases, luminescence values were normalized to both total protein content and untreated controls. Results represent means ± standard deviations from three independent biological replicates (n = 3), each performed in technical triplicate.

Data are means ± standard deviations for three biological replicates (n=3), where each biological replicate was performed in technical triplicate. Statistics are two-way analysis of variance (ANOVA) with Dunnett's multiple comparisons test against **2´OMePS+** (Figure 5 and Figure 6). Oligonucleotides used in this study did not display any significant toxicity as observed by cell growth.

5.1 Cell Viability Assay

Cell seeding and treatment: HeLa pLuc/705 cells were seeded in 96-well tissue culture plates (clear bottom, polystyrene) at a density of 10,000 cells per well in a final volume of 100 µL. Cells were incubated with oligonucleotides at a final concentration of 20 µM, prepared by combining 50 µL oligonucleotide solution in Opti-MEM with 50 µL cells in DMEM supplemented with 6% FBS and 2× antibiotic–antimycotic, resulting in a 1:1 DMEM:Opti-MEM mixture containing 3% FBS and 1× antibiotic–antimycotic. Cells were incubated for 72 h at 37 °C, 5% CO_2.

Protocol 1: CellTiter-Glo® Luminescent ATP assay

After 72h, at the assay end point, 50 µL of media was removed from each well. Then 50 µL of CellTiter-Glo® Reagent (Promega) was added equal to the volume of cell culture medium and reagent in each well. The plates were placed on an orbital shaker to mix the contents for 2 minutes to induce cell lysis. The plates were then incubated at room temperature for 10 minutes to stabilize the luminescent signal. Then Luminescence was recorded using a CLARIOstar microplate reader (BMG Labtech, software version 5.21.R2).

Protocol 2: Resazurin (alamarBlue™ HS) metabolic viability assay

After 72h, at the assay end point, 5 µL of alamarBlue™ HS (Thermo Fischer Scientific) Cell Viability Reagent was added to each well. The plates were incubated for 1h at 37°C. Fluorescence was recorded using a CLARIOstar microplate reader (BMG Labtech, software version 5.21.R2) with excitation at 570 nm and emission at 590 nm.

Results are presented as mean ± standard deviation from three independent biological replicates (n = 3), each performed in technical triplicate. Background signals from cell-free wells were subtracted prior to analysis.

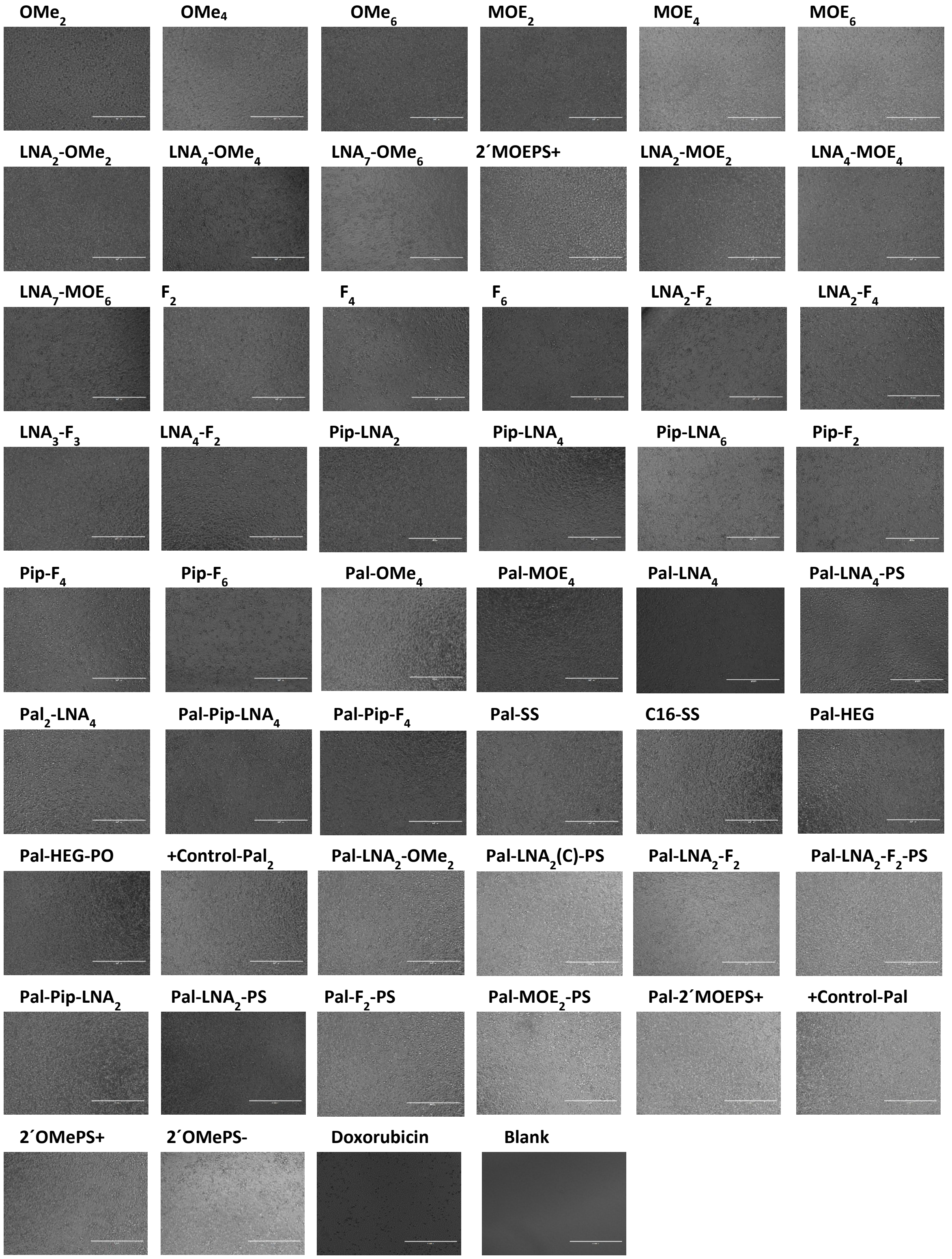

Supplementary Figure S140: Representative micrographs of HeLa pLuc/705 cells treated with oligonucleotides at a concentration 20 μM under gymnosis conditions. Images were captured at the assay endpoint 72 h later. Scale bar = 400 μm.

A.

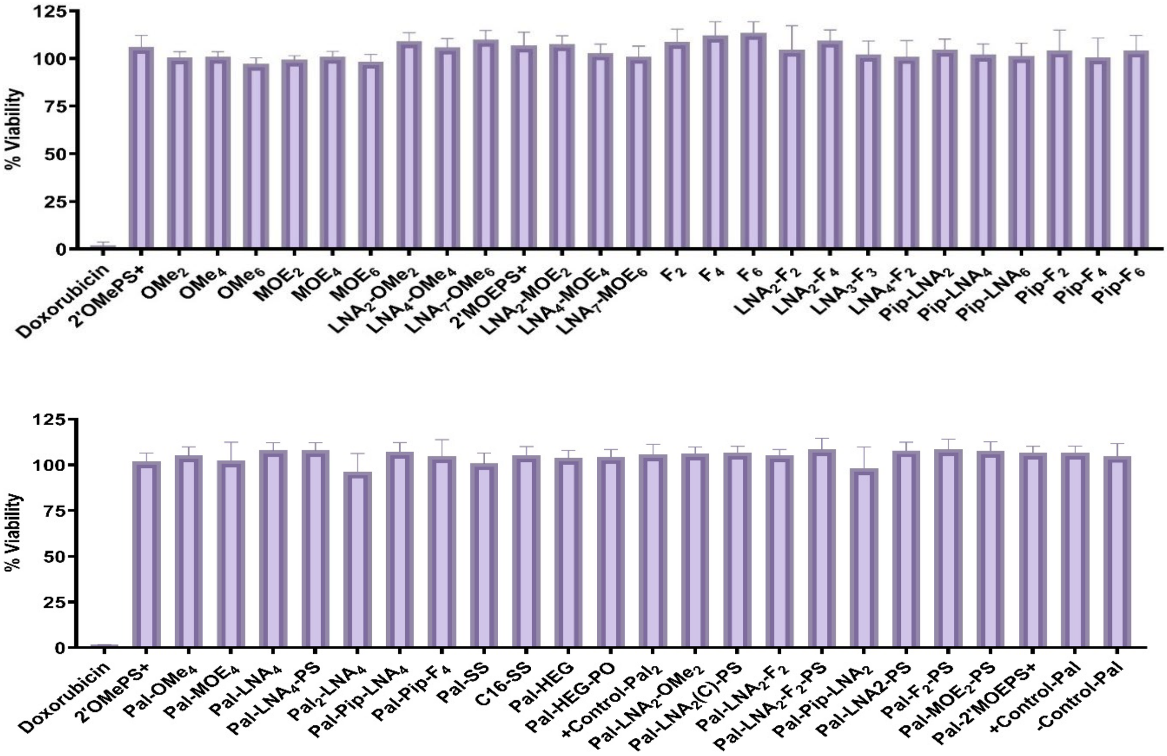

B.

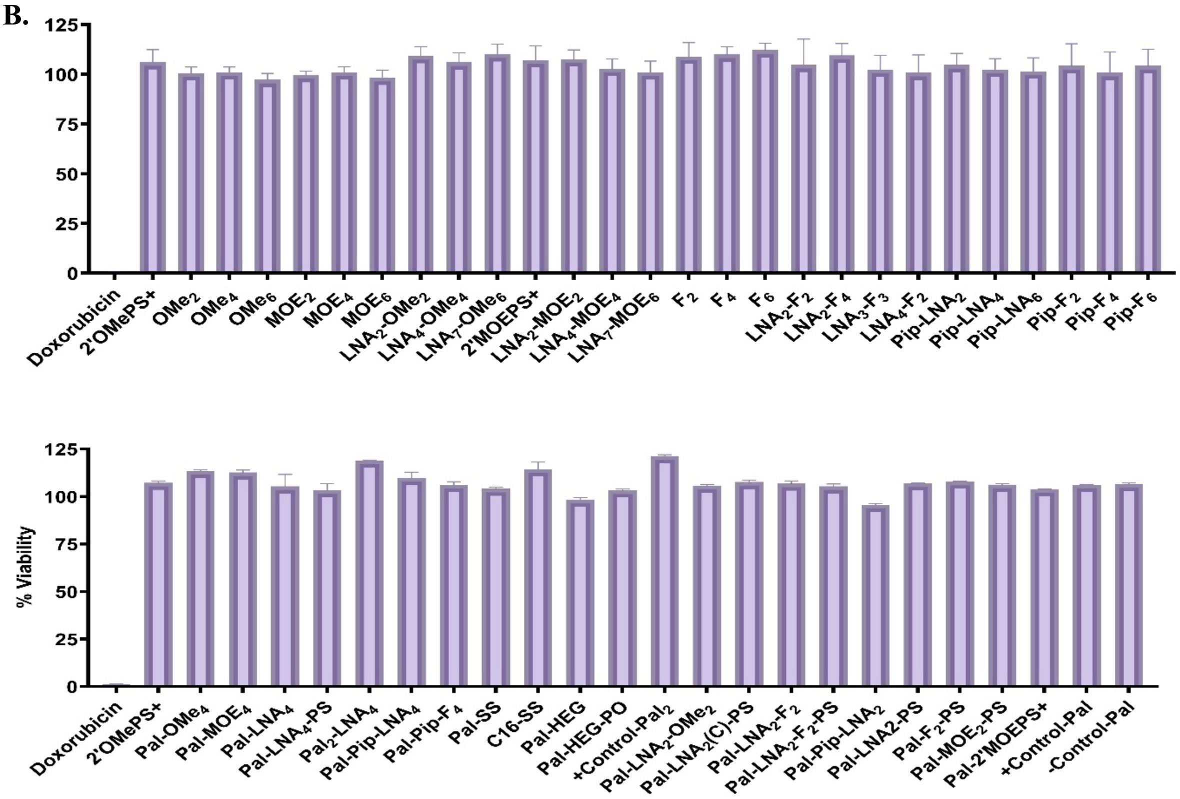

Supplementary Figure S141: % cell viability compared to untreated of HeLa pLuc/705 cells treated with oligonucleotides at a concentration 20 µM under gymnosis conditions. **A.** Measurement based on CellTiter-Glo® Luminescent ATP Assay and **B.** alamarBlue™ HS metabolic viability Assay.

5.2 Gymnosis Data of Pip-LNA2, LNA2 and LNA2-PS

Table T17: Duplex melting temperatures (Tm) in °C of **Pip-LNA2, LNA2** and **LNA2-PS** oligonucleotides with complementary RNA. Nucleotides marked in black have 2′-OMe ribose sugars and phosphorothioate internucleoside linkages. Nucleotides marked in red-bold-underlined are LNA THP phosphothiotriesters. Nucleotides marked in lowercase-red are LNA Piperidine phosphothiotriesters. Nucleotides marked in uppercase red are LNA phosphorothioates

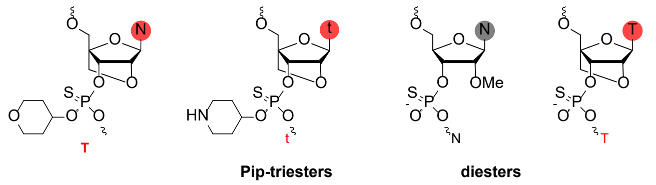

Oligonucleotide	Sequence (5′→3′)	RNA target Tm (ΔTm)
2′OMePS-	CCU CUU ACC UCA GUU ACA	----
2′OMePS+	CCU CAU UCA CUC GAU UCA	61.3 (---)
Pip-LNA2	CCU CUt ACC UCA GUt ACA	67.8 (+6.5)
LNA2	CCU CUT ACC UCA GUT ACA	67.6 (+6.3)*
LNA2-PS	CCU CUT ACC UCA GUT ACA	70.4 (+9.1)*

*Tm were taken from reference 1

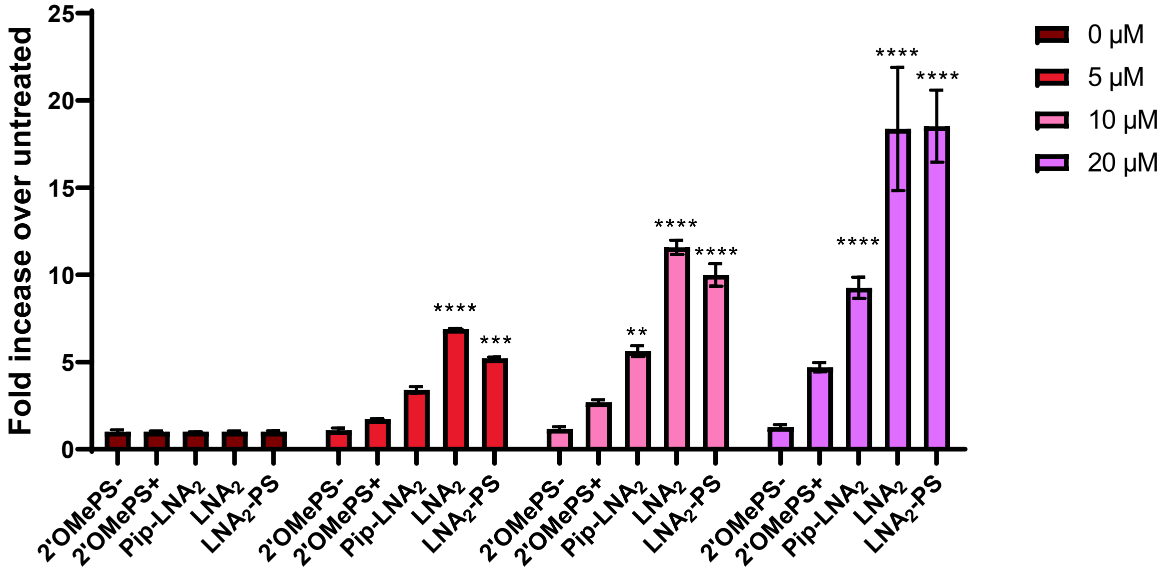

Supplementary Figure S142: Activities under gymnotic condition of ONs in HeLa pLuc/705 cells. ONs were treated to HeLa pLuc/705 cells at the indicated concentrations without transfection reagent, and luciferase activity was measured 72 h later. In all cases, luminescence was normalized to total protein quantity and untreated cells. Data are means ± standard deviations for three biological replicates (n = 3), where each biological replicate was performed in technical triplicate. Statistics are two-way analysis of variance (ANOVA) with Dunnett's multiple comparisons test against 2′OMePS+, α = 0.05: **P ≤ 0.0019, ***P ≤ 0.0002, and **** P ≤ 0.0001.

5.3 Relative Splice-Switching Activity (SSA)

Table T18: Fold increase in splice-switching activity compared to **2′OMePS+** under gymnosis conditions at three different concentrations: 5 µM, 10 µM and 20 µM.

Oligonucleotides	SSA			Oligonucleotides	SSA		
	5 µM	10 µM	20 µM		5 µM	10 µM	20 µM
**OMe$_2$**	0.88	1.06	0.96	**F$_2$**	0.87	0.73	0.63
**OMe$_4$**	0.58	0.54	0.49	**F$_4$**	0.55	0.40	0.25
**OMe$_6$**	0.43	0.29	0.21	**F$_6$**	0.44	0.26	0.15
**MOE$_2$**	1.13	1.10	1.09	**LNA$_2$-F$_2$**	2.72	3.00	2.71
**MOE$_4$**	0.76	0.60	0.45	**LNA$_2$-F$_4$**	0.66	0.44	0.27
**MOE$_6$**	0.62	0.37	0.27	**LNA$_3$-F$_3$**	0.86	0.71	0.51
**LNA$_2$-OMe$_2$**	3.02	3.54	3.30	**LNA$_4$-F$_2$**	0.95	0.82	0.68
**LNA$_4$-OMe$_4$**	0.54	0.40	0.32	**Pip-LNA$_2$**	2.08	2.30	2.68
**LNA$_7$-OMe$_6$**	0.51	0.39	0.27	**Pip-LNA$_4$**	1.73	1.87	1.96
**2´MOEPS+**	1.90	2.24	3.06	**Pip-LNA$_6$**	0.79	0.66	0.63
**LNA$_2$-MOE$_2$**	1.65	1.81	1.94	**Pip-F$_2$**	0.73	0.57	0.50
**LNA$_4$-MOE$_4$**	0.71	0.66	0.54	**Pip-F$_4$**	0.56	0.42	0.28
**LNA$_7$-MOE$_6$**	0.52	0.31	0.25	**Pip-F$_6$**	0.60	0.37	0.18
**Lipid conjugated oligonucleotides**							
**Pal-OMe$_4$**	0.76	0.62	0.53	**+Control-Pal$_2$**	0.98	1.10	1.36
**Pal-MOE$_4$**	0.76	0.61	0.53	**Pal-LNA$_2$-OMe$_2$**	1.83	2.55	2.74
**Pal-LNA$_4$**	4.21	4.48	4.64	**Pal-LNA$_2$(C)-PS**	6.76	6.47	6.30
**Pal-LNA$_4$-PS**	3.90	3.63	3.25	**Pal-LNA$_2$-F$_2$**	1.84	2.28	2.31
**Pal$_2$-LNA$_4$**	2.50	2.78	3.95	**Pal-LNA$_2$-F$_2$-PS**	6.57	8.09	7.05
**Pal-Pip-LNA$_4$**	7.52	7.56	6.49	**Pal-Pip-LNA$_2$**	11.3	11.0	9.0
**Pal-Pip-F$_4$**	0.68	0.60	0.52	**Pal-LNA$_2$-PS**	8.80	9.58	8.30
**Pal-SS**	0.53	0.60	0.79	**Pal-F$_2$-PS**	2.93	3.47	3.52
**C16-SS**	0.51	0.93	2.07	**Pal-MOE$_2$-PS**	4.73	5.19	5.16
**Pal-HEG**	1.93	1.94	1.91	**Pal-2´MOEPS+**	1.32	1.24	1.11
**Pal-HEG-PO**	1.59	1.74	1.71				
**+Control-Pal**	3.21	3.56	3.24				

6.0 NMR Spectra of Compounds

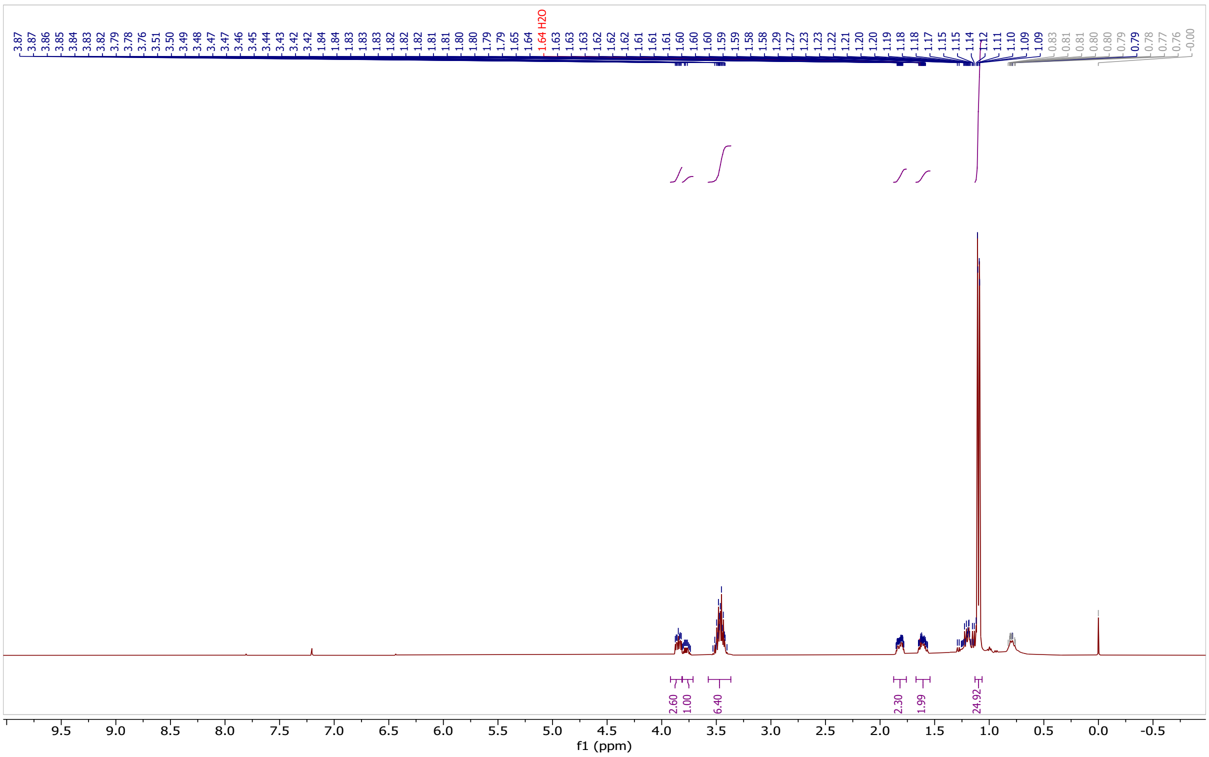

Supplementary Figure S143: ^{1}H NMR (400 MHz, $CDCl_3$) spectrum of **4 (crude).**

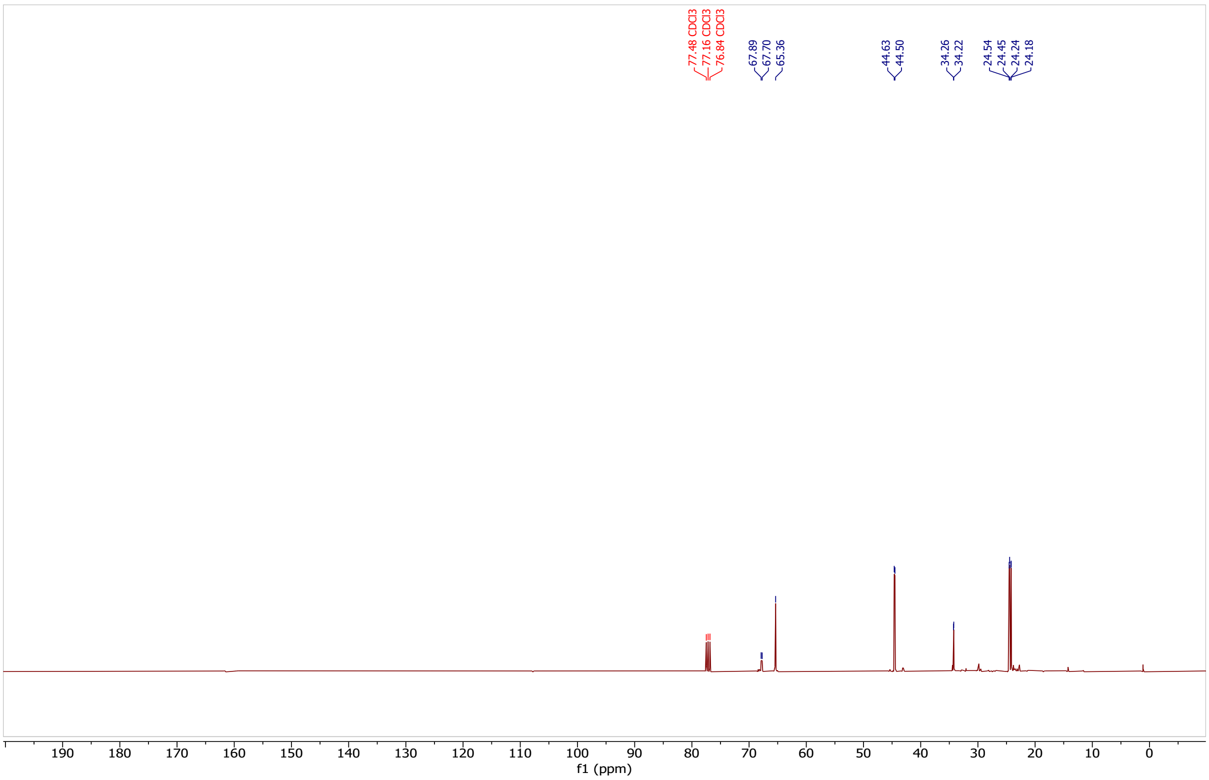

Supplementary Figure S144: ^{31}P NMR (162 MHz, $CDCl_3$) spectrum of **4 (crude).**

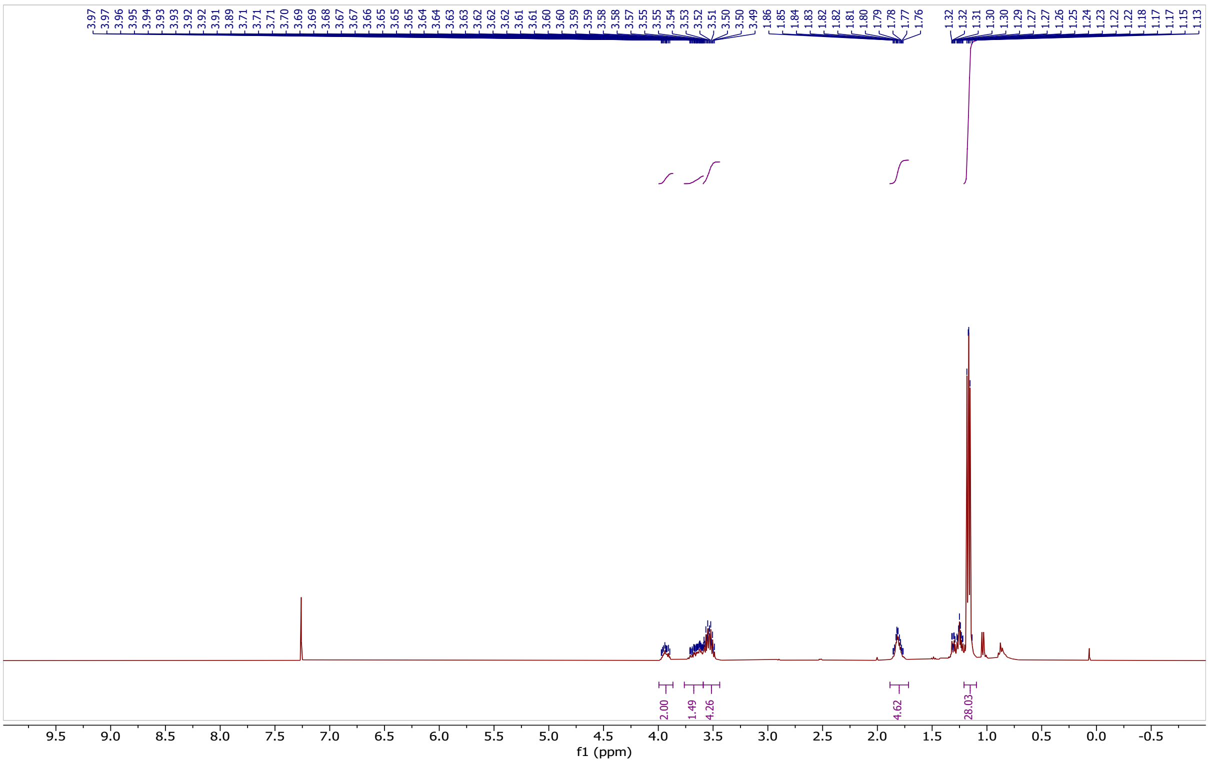

Supplementary Figure S145: ^{1}H NMR (400 MHz, CDCl3) spectrum of **5 (crude).**

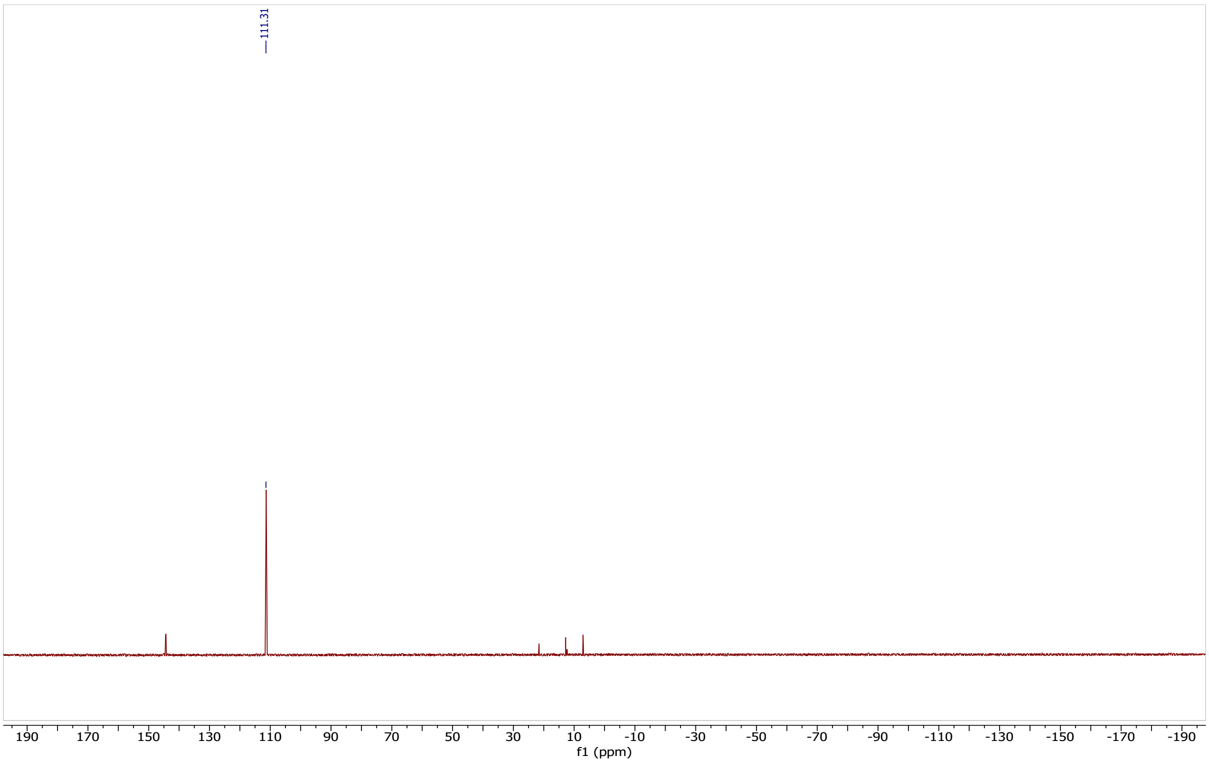

Supplementary Figure 146: ^{31}P NMR (162 MHz, $CDCl_3$) spectrum of **5 (crude).**

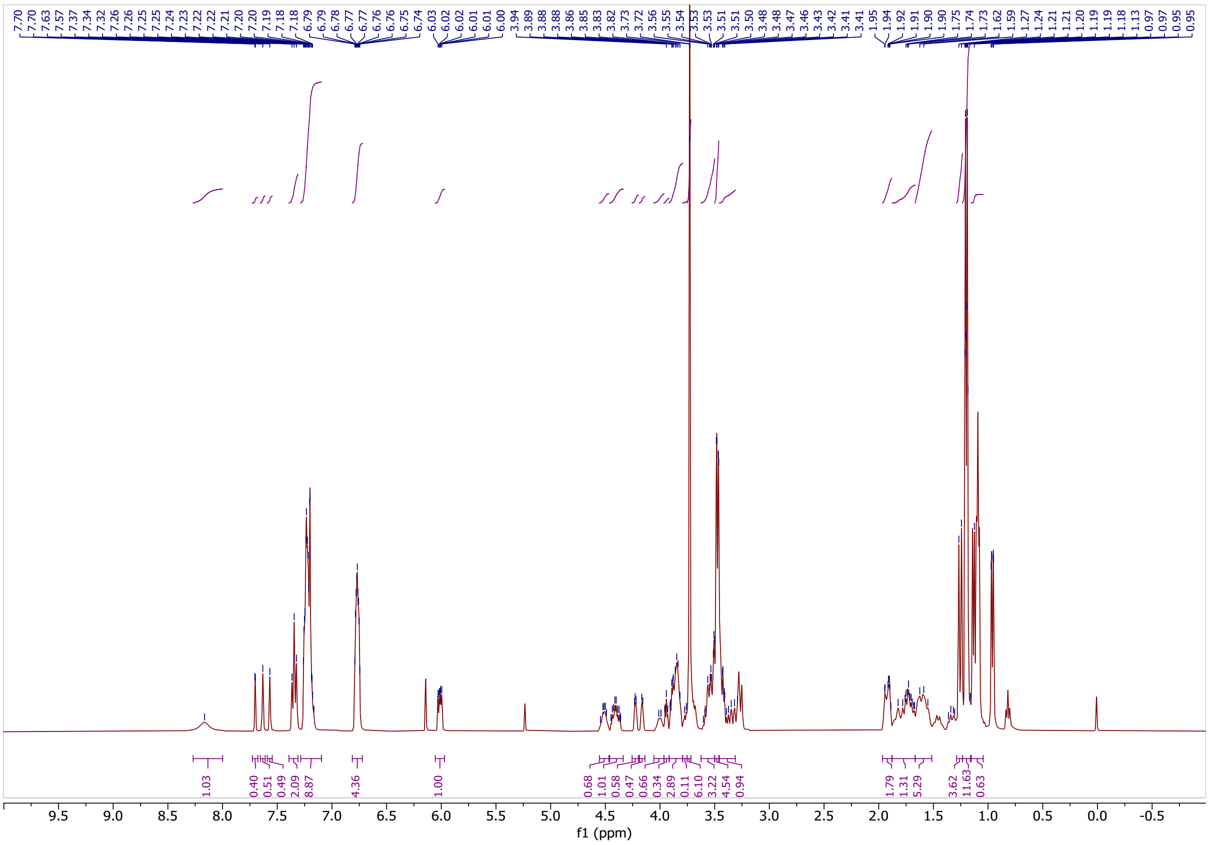

Supplementary Figure S147: ^{1}H NMR (400 MHz, CDCl3) spectrum of **9.**

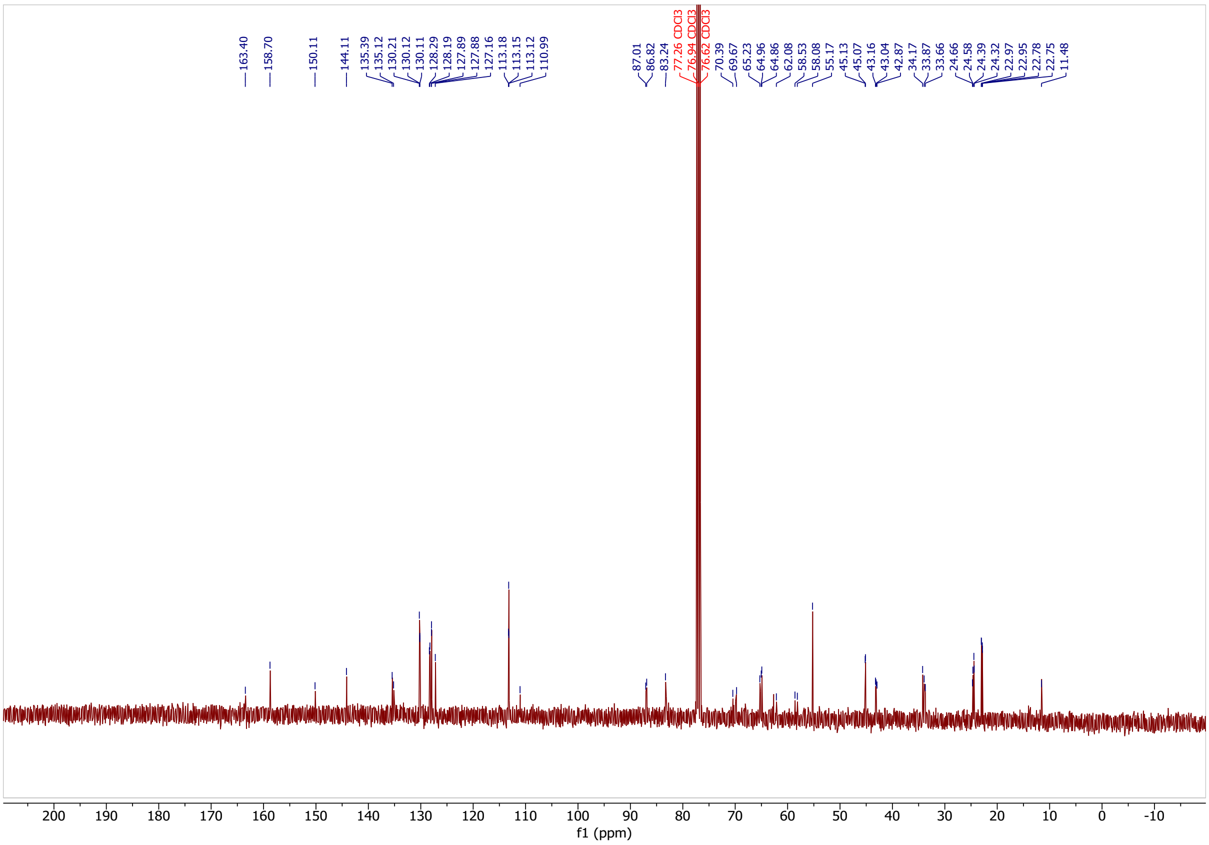

Supplementary Figure S148: ^{13}C NMR (101 MHz, $CDCl_3$) spectrum of **9.**

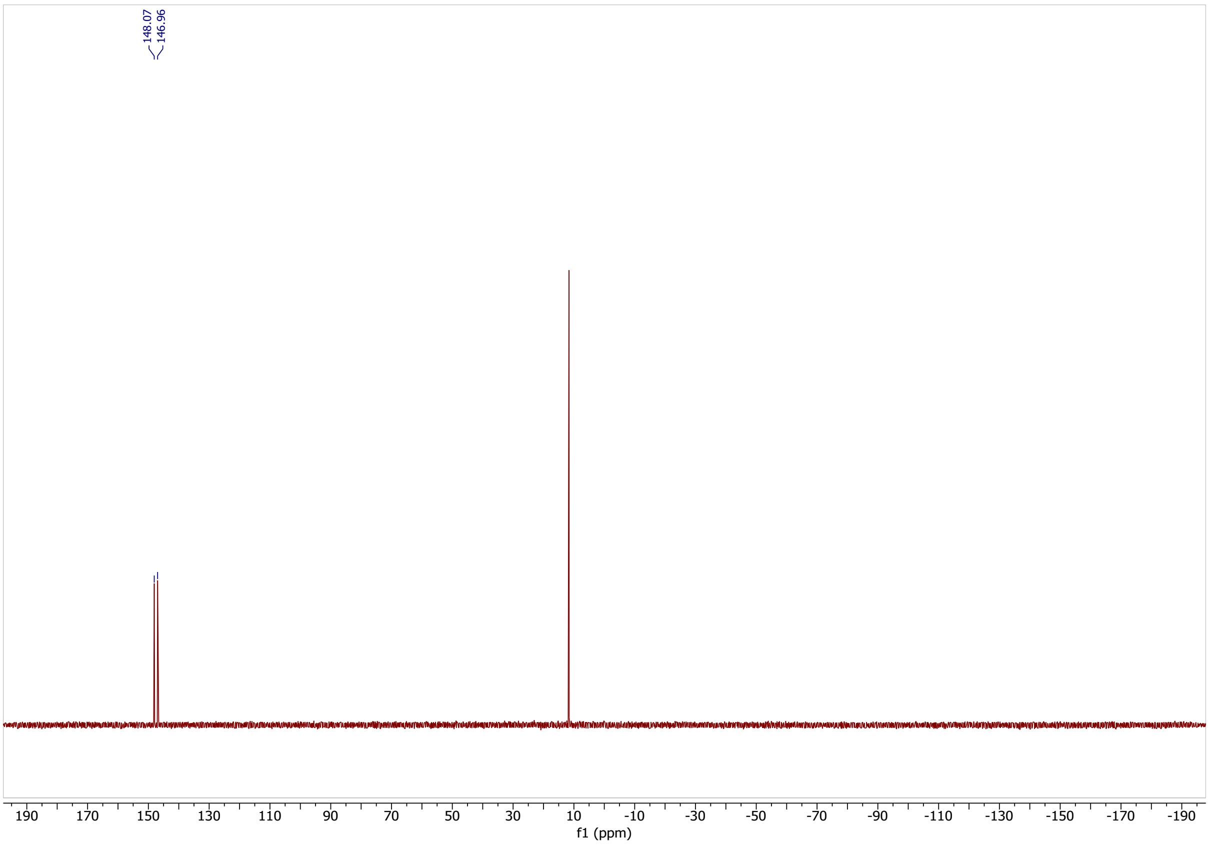

Supplementary Figure S149: ^{31}P NMR (162 MHz, $CDCl_3$) spectrum of **9.**

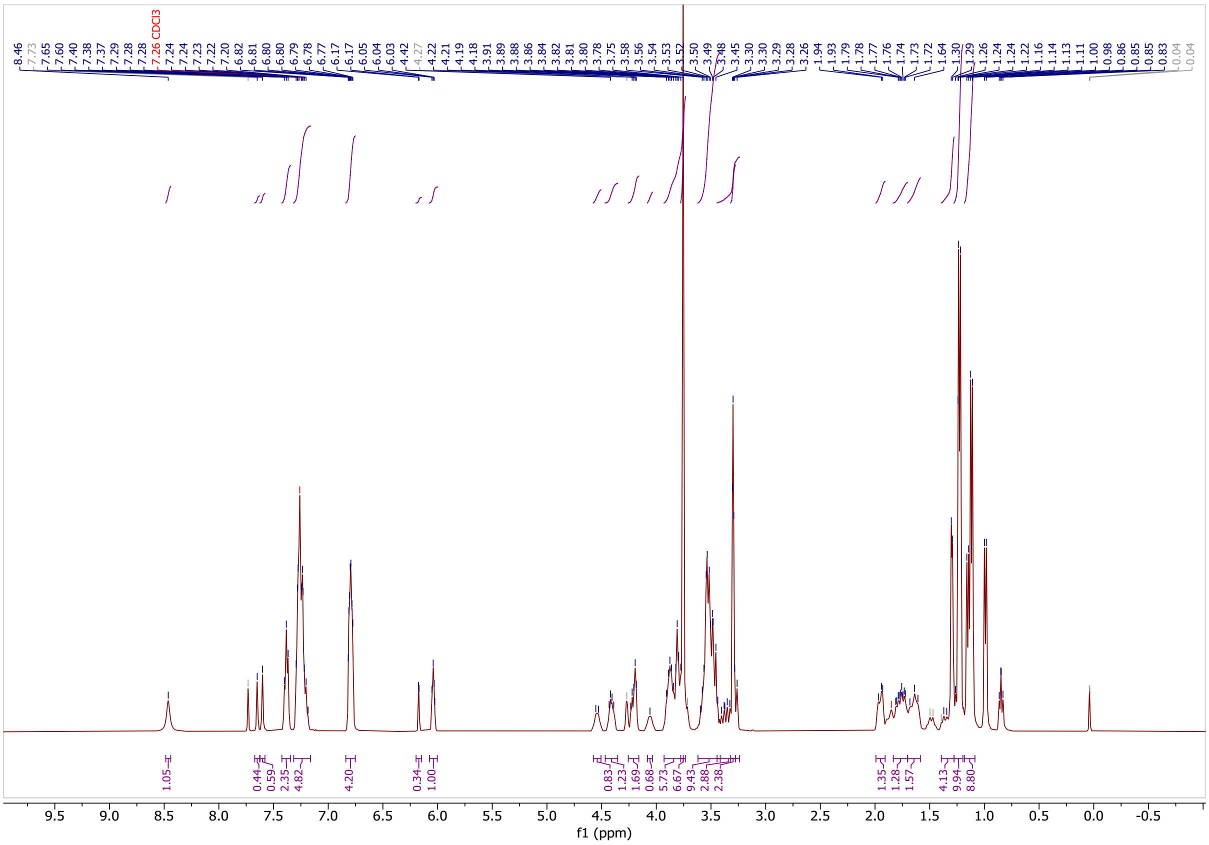

Supplementary Figure S150: ^{1}H NMR (400 MHz, $CDCl_3$) spectrum of **10.**

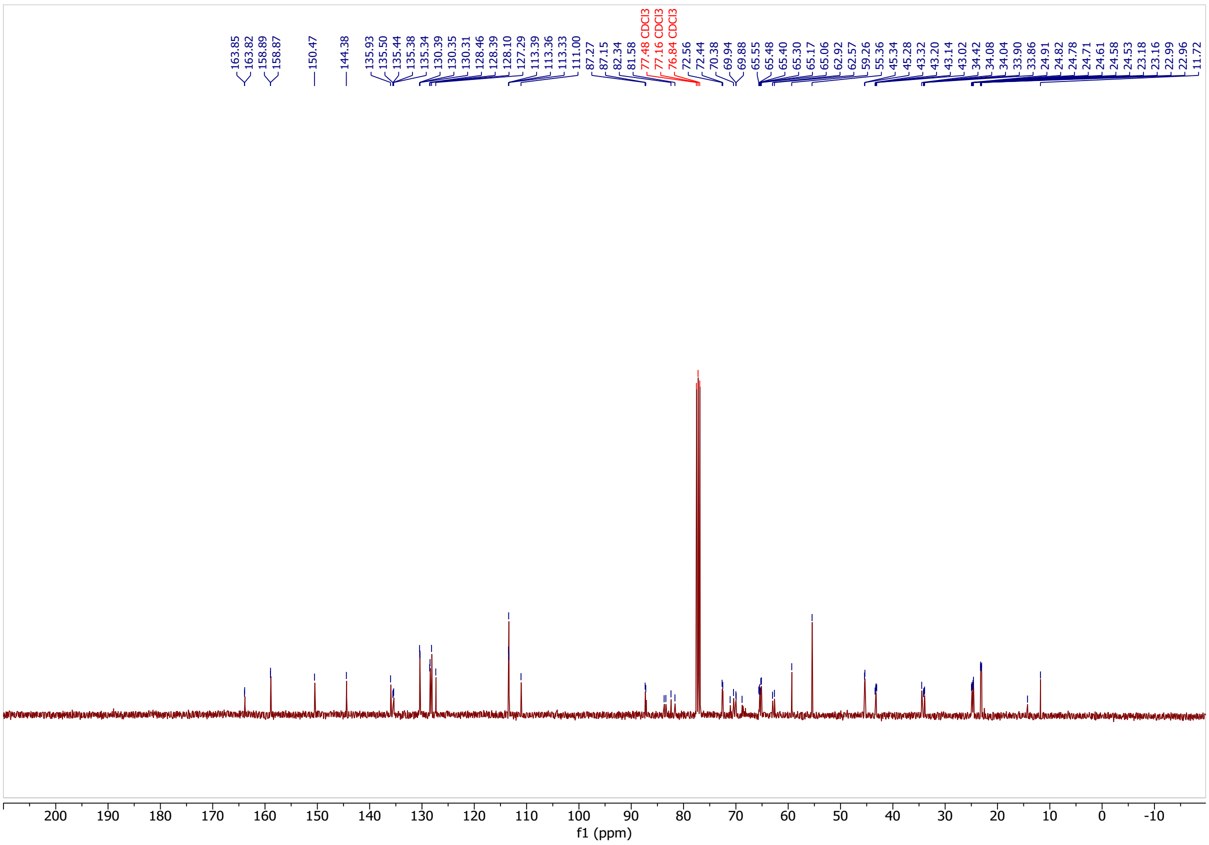

Supplementary Figure S151: $^{13}C\{^{1}H\}$ NMR (101 MHz, $CDCl_3$) spectrum of **10.**

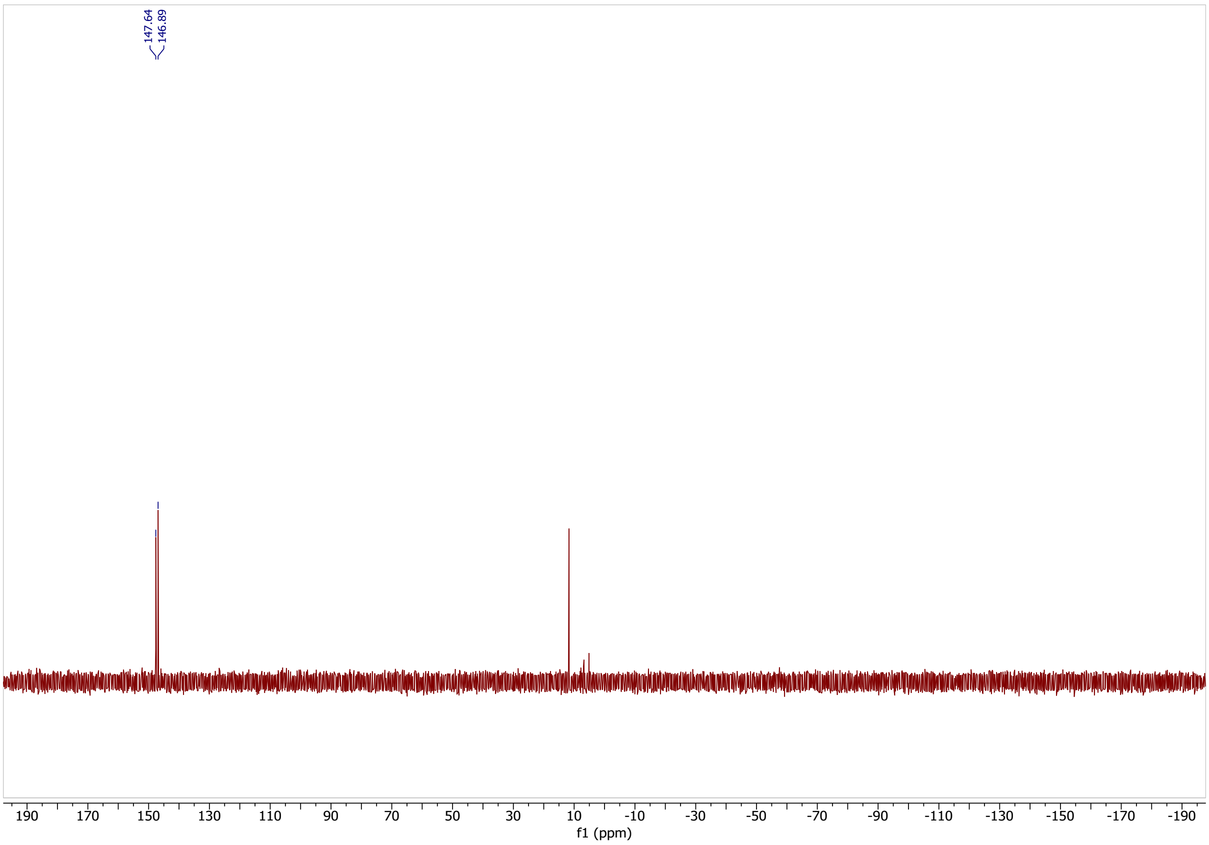

Supplementary Figure S152: ^{31}P NMR (162 MHz, $CDCl_3$) spectrum of **10.**

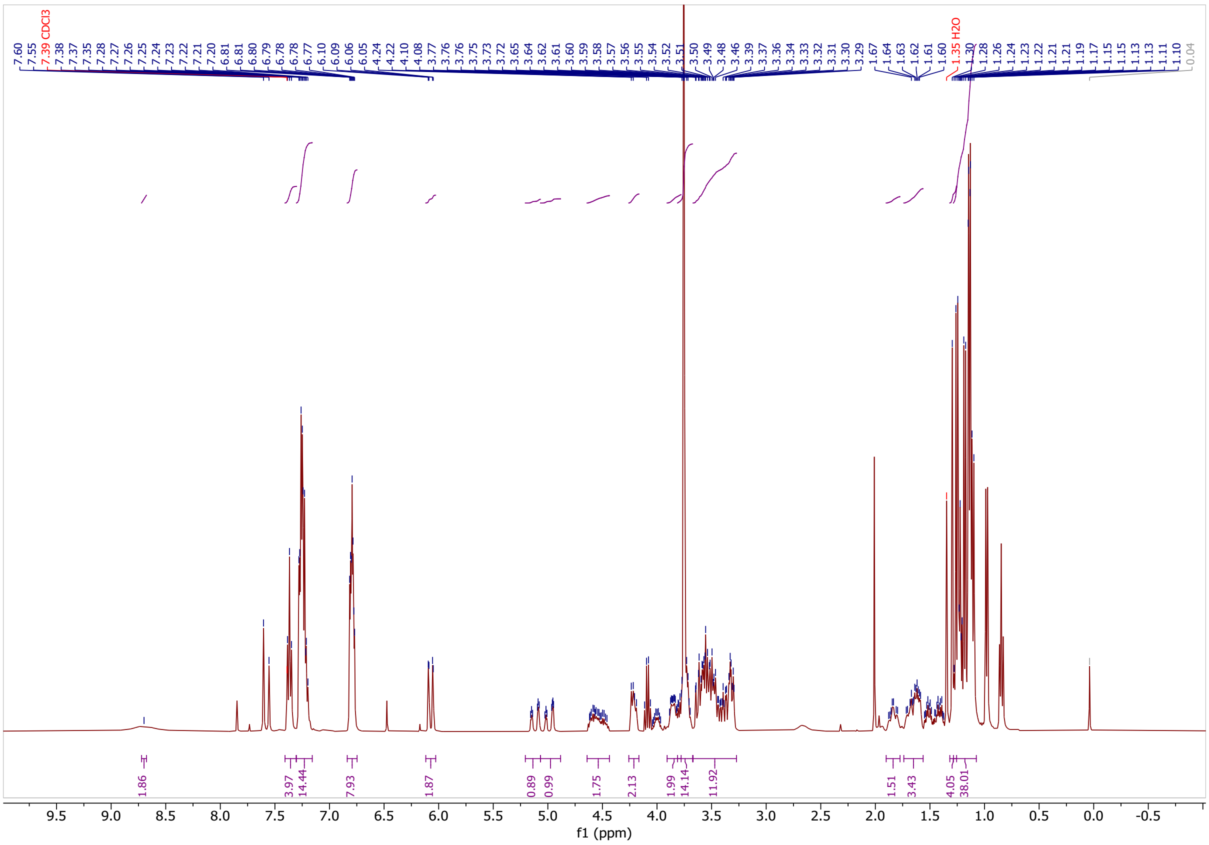

Supplementary Figure S153: ^{1}H NMR (400 MHz, $CDCl_3$) spectrum of **11.**

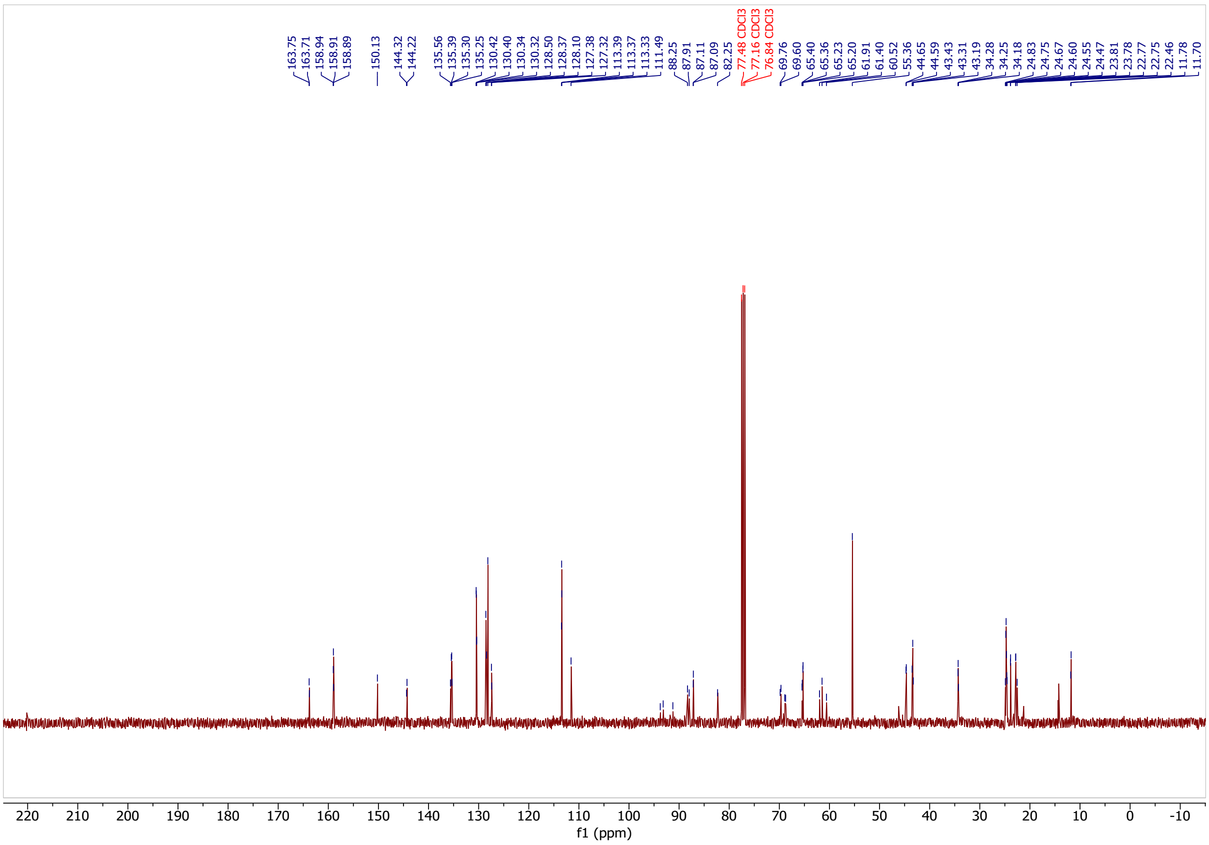

Supplementary Figure S154: $^{13}C\{^{1}H\}$ NMR (101 MHz, $CDCl_3$) spectrum of **11.**

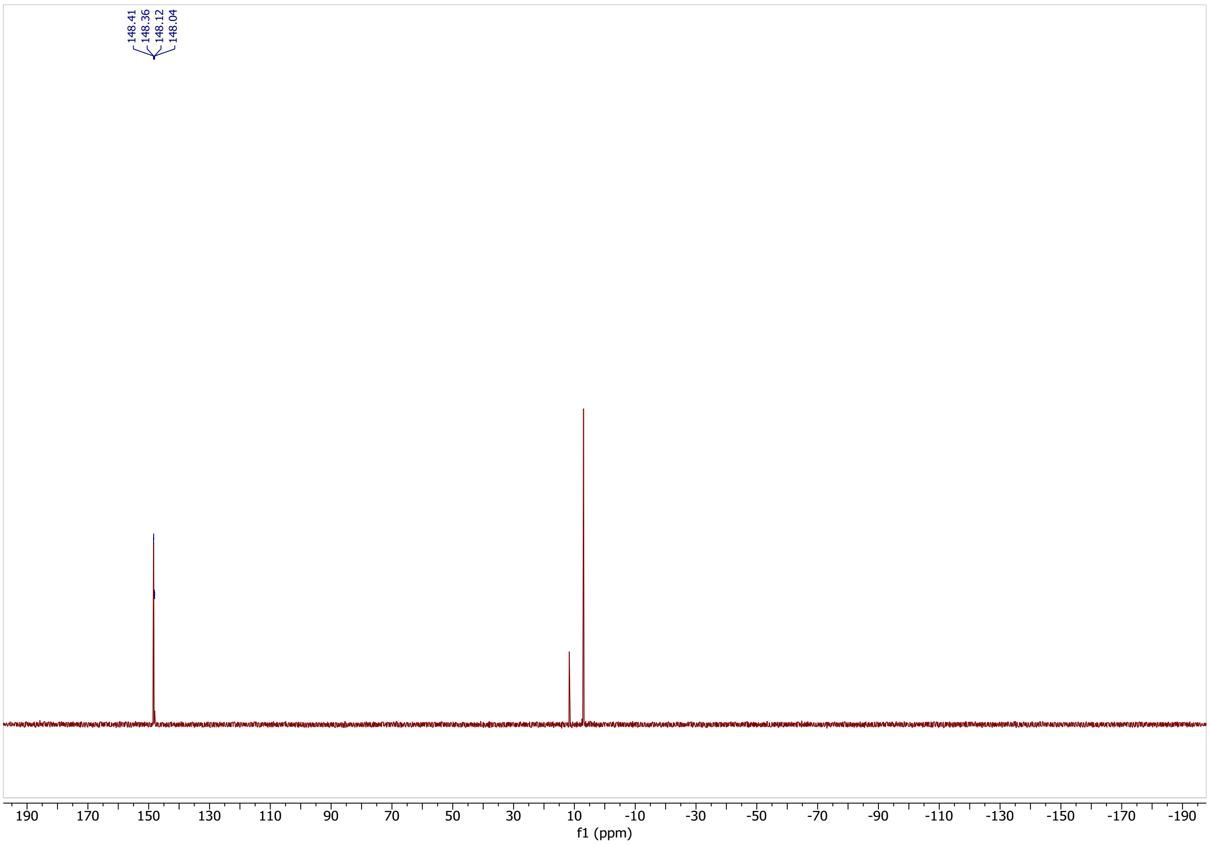

Supplementary Figure S155: ^{31}P NMR (162 MHz, $CDCl_3$) spectrum of **11.**

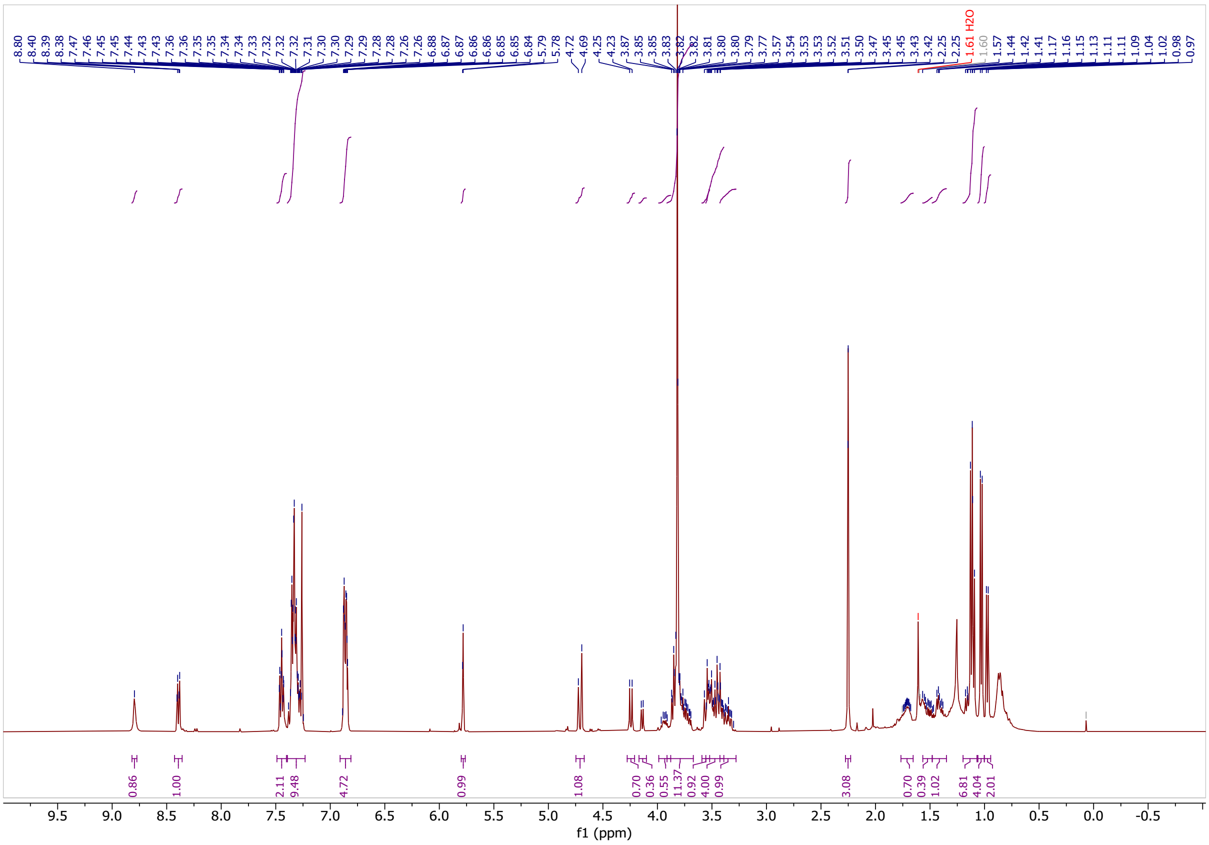

Supplementary Figure S156: ^{1}H NMR (400 MHz, $CDCl_3$) spectrum of **17.**

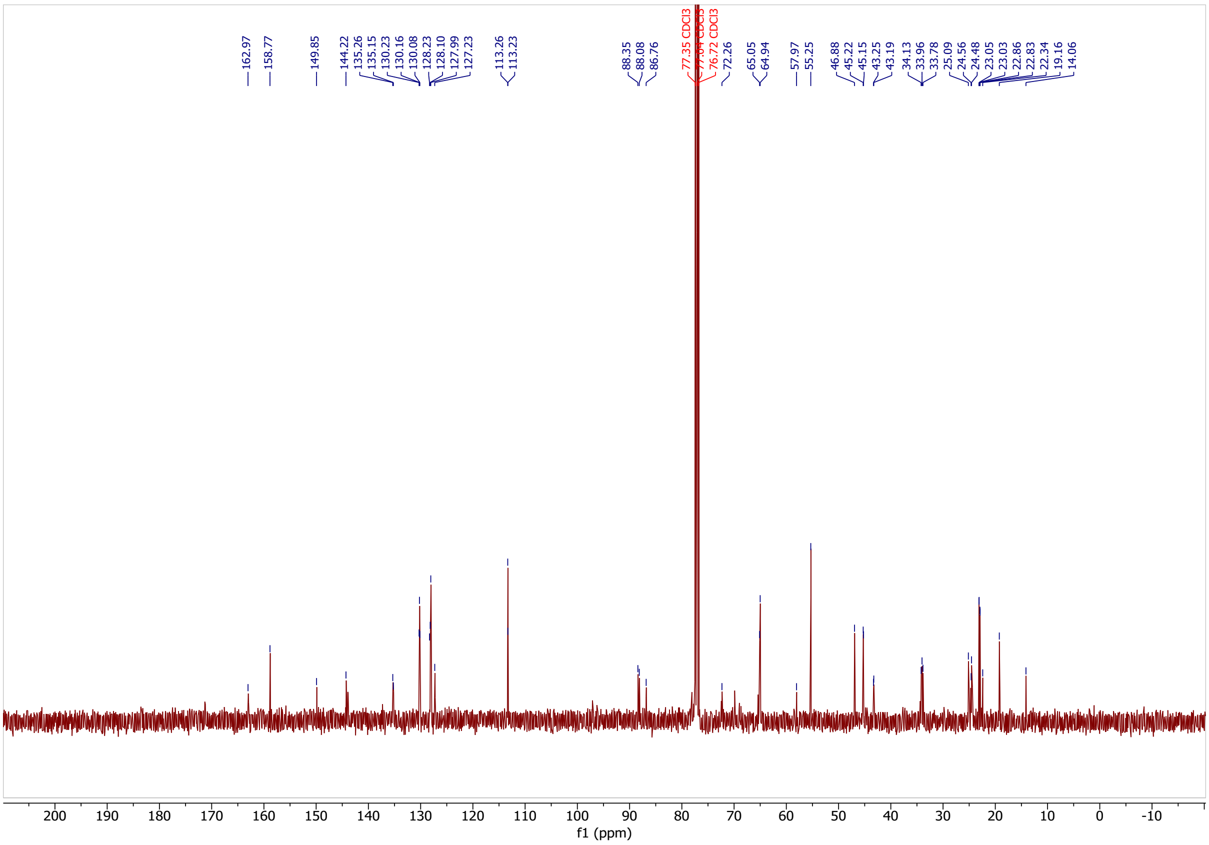

Supplementary Figure S157: $^{13}C\{^{1}H\}$ NMR (101 MHz, $CDCl_3$) spectrum of **17.**

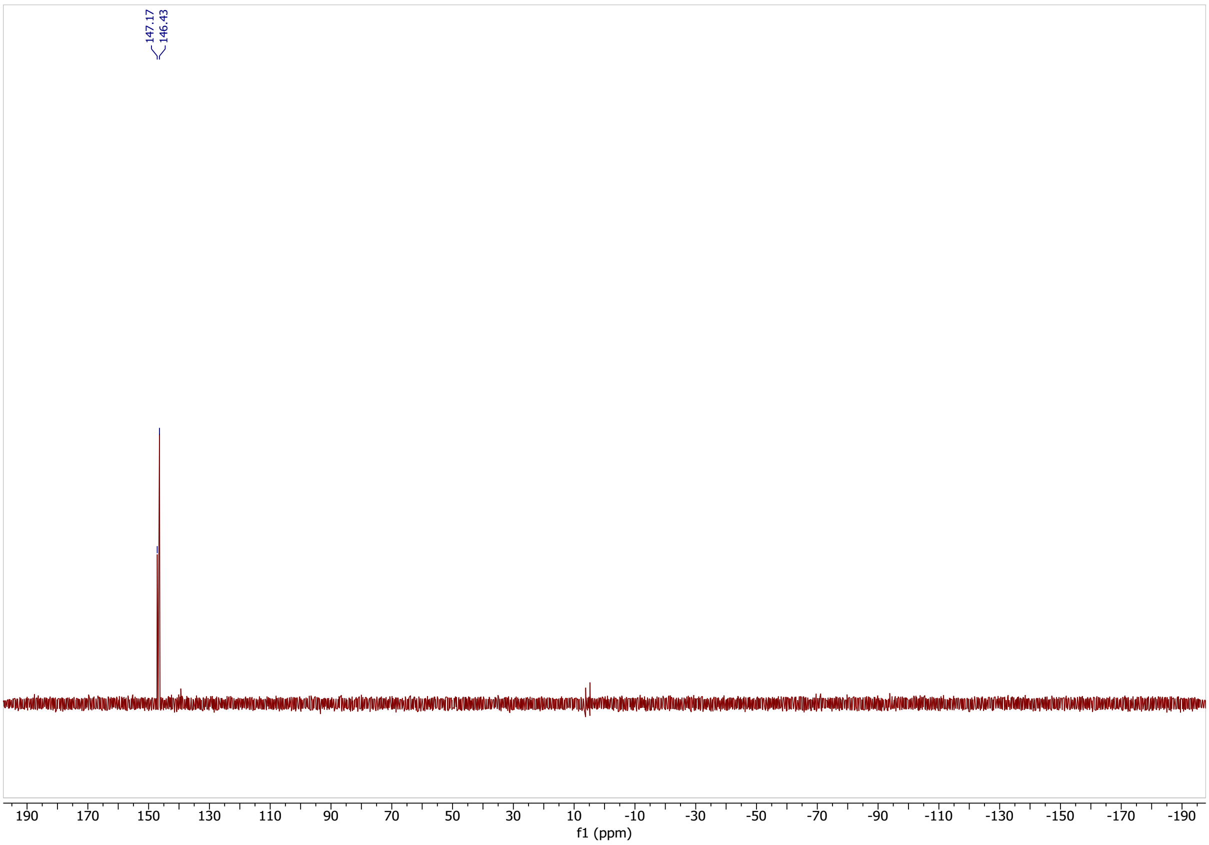

Supplementary Figure S158: ^{31}P NMR (162 MHz, $CDCl_3$) spectrum of **17.**

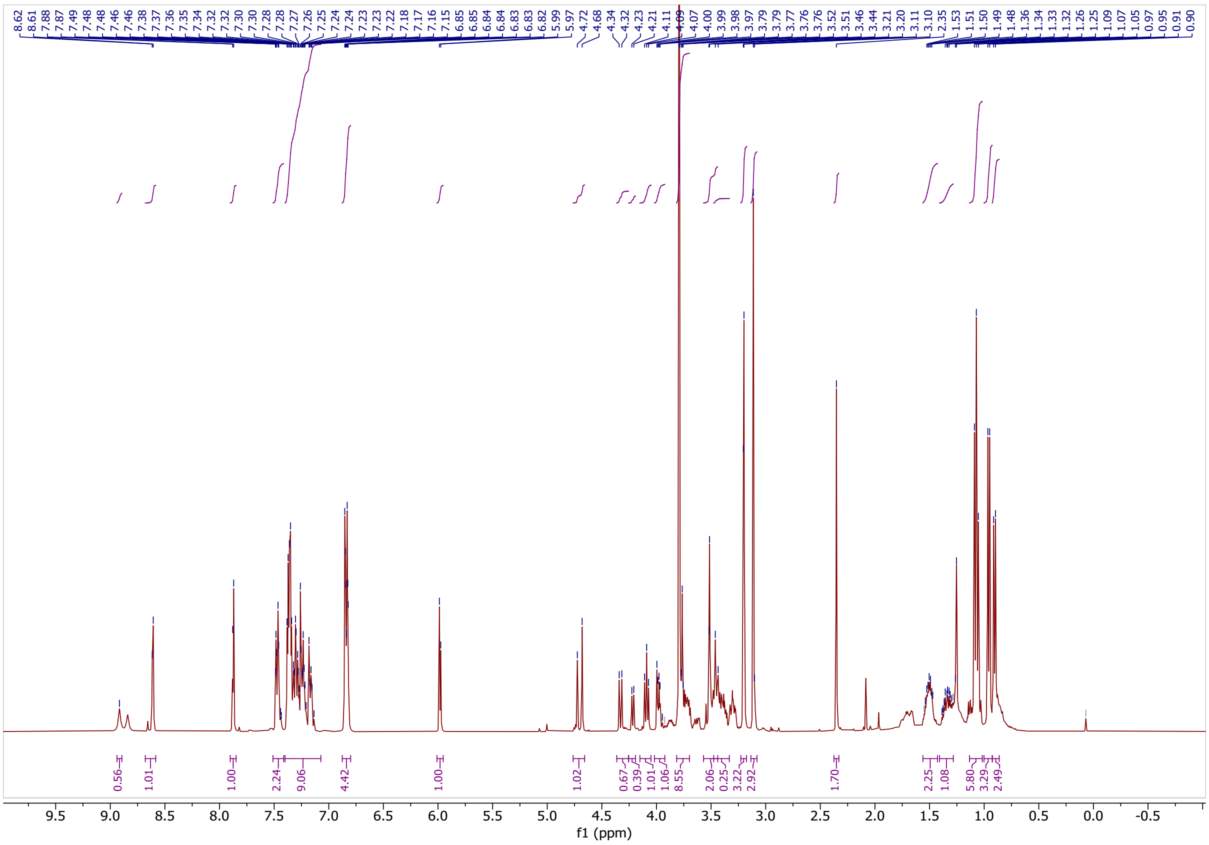

Supplementary Figure S159: ^{1}H NMR (400 MHz, $CDCl_3$) spectrum of **19.**

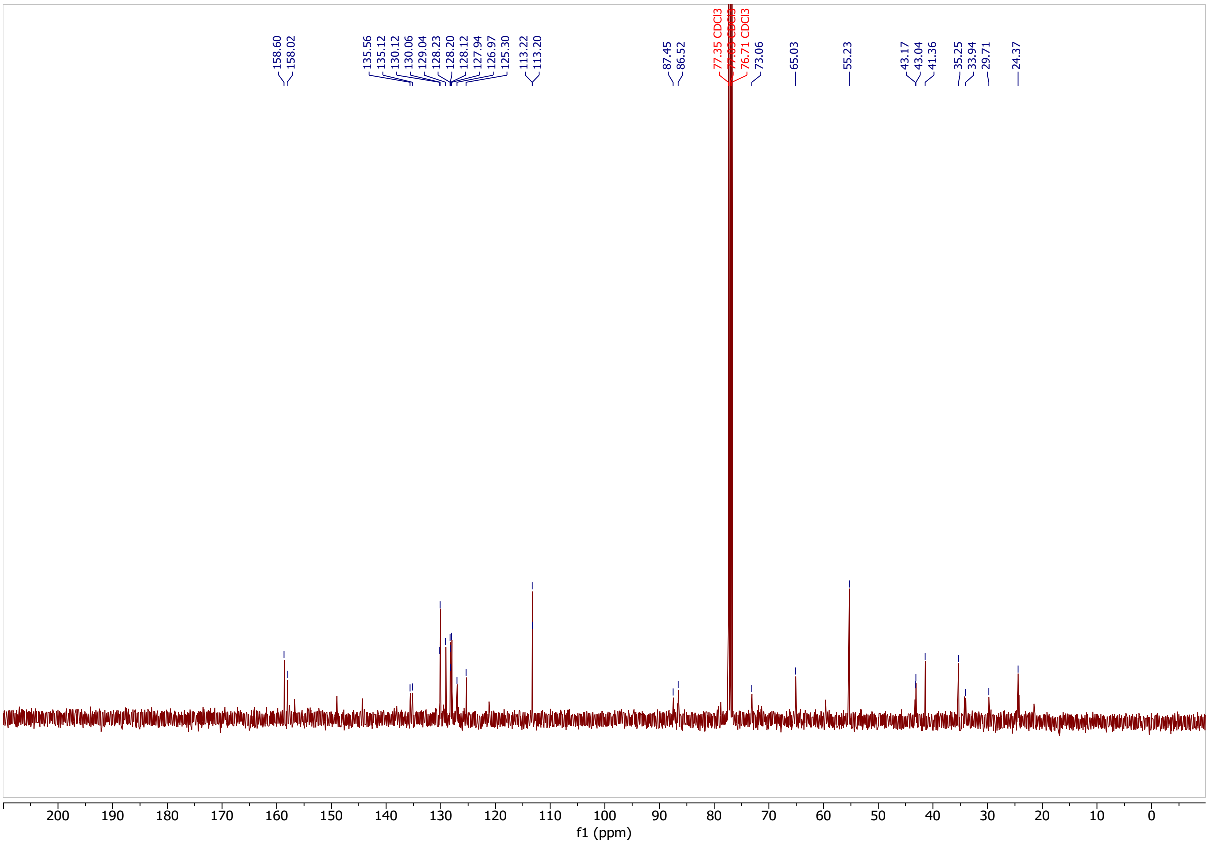

Supplementary Figure S160: $^{13}C\{^{1}H\}$ NMR (101 MHz, $CDCl_3$) spectrum of **19.**

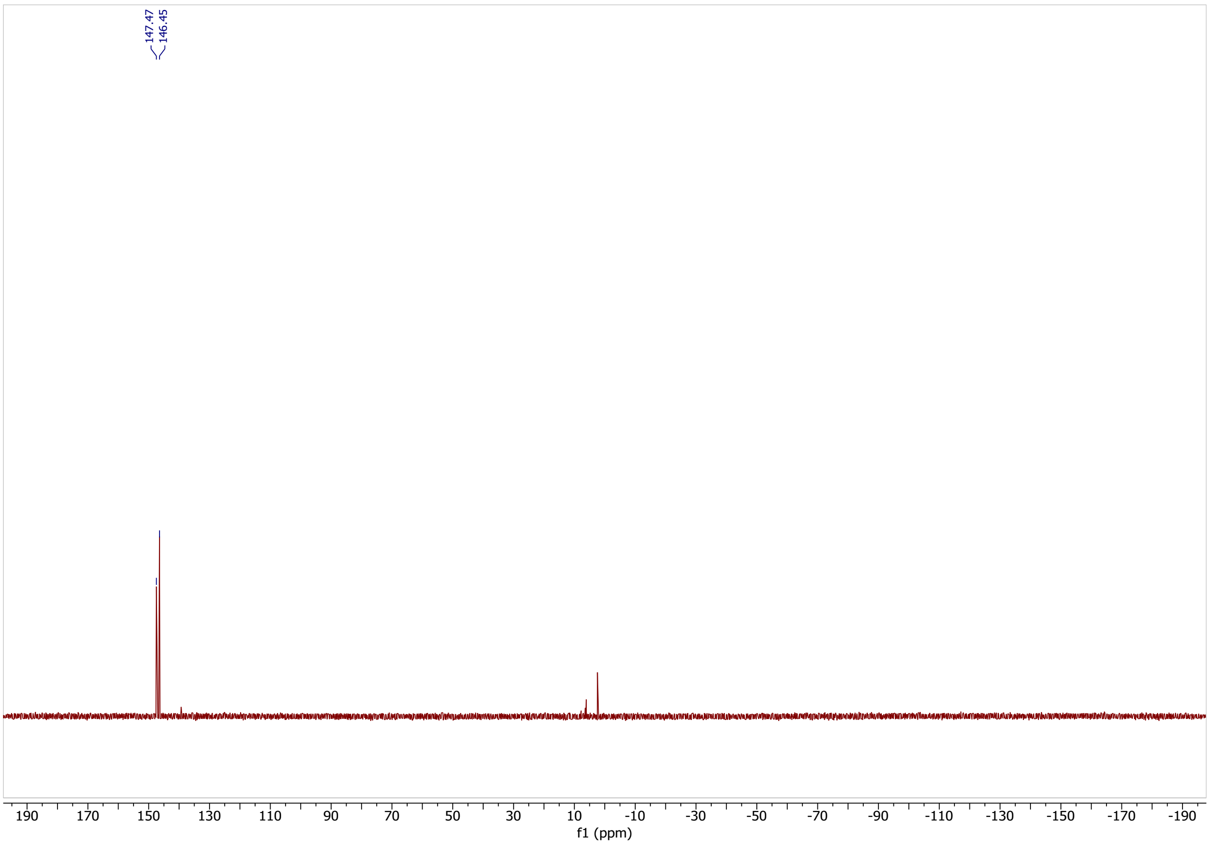

Supplementary Figure S161: ^{31}P NMR (162 MHz, $CDCl_3$) spectrum of **19.**

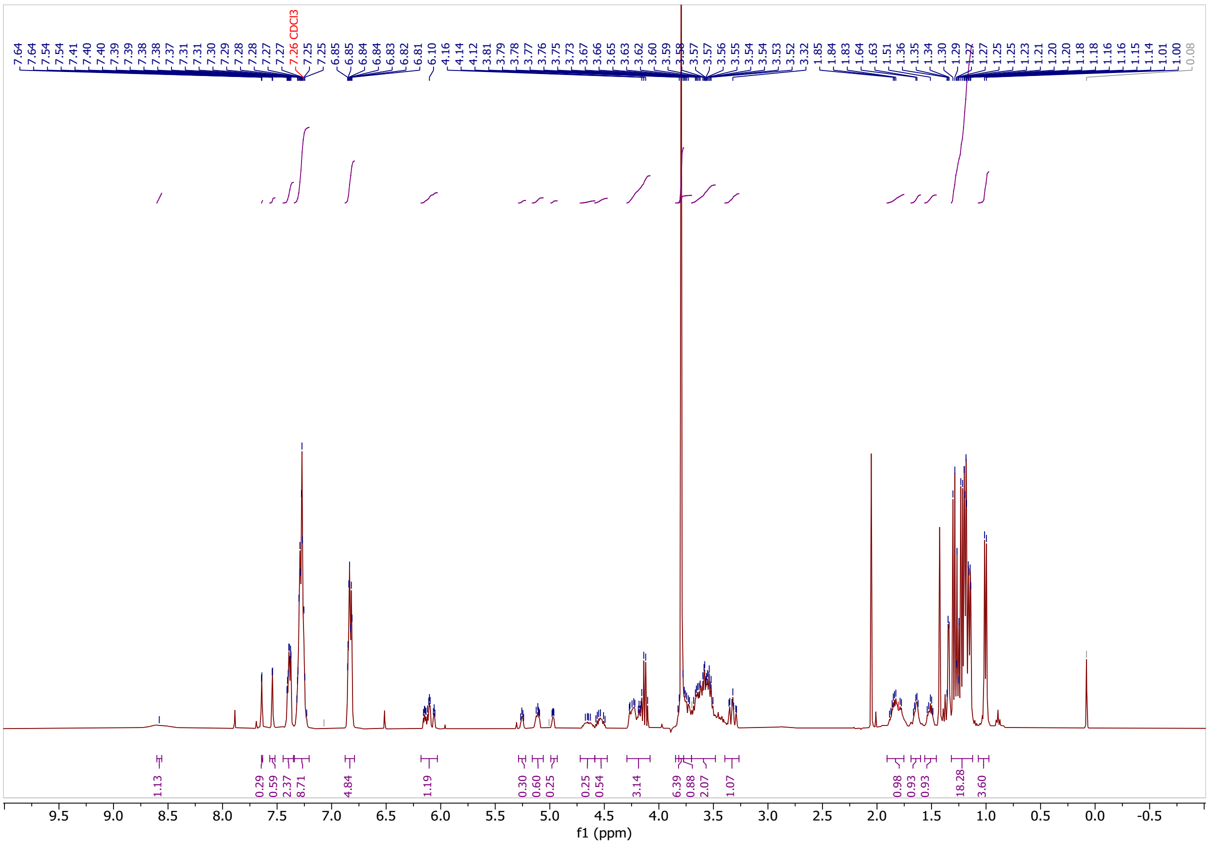

Supplementary Figure S162: ^{1}H NMR (400 MHz, $CDCl_3$) spectrum of **20.**

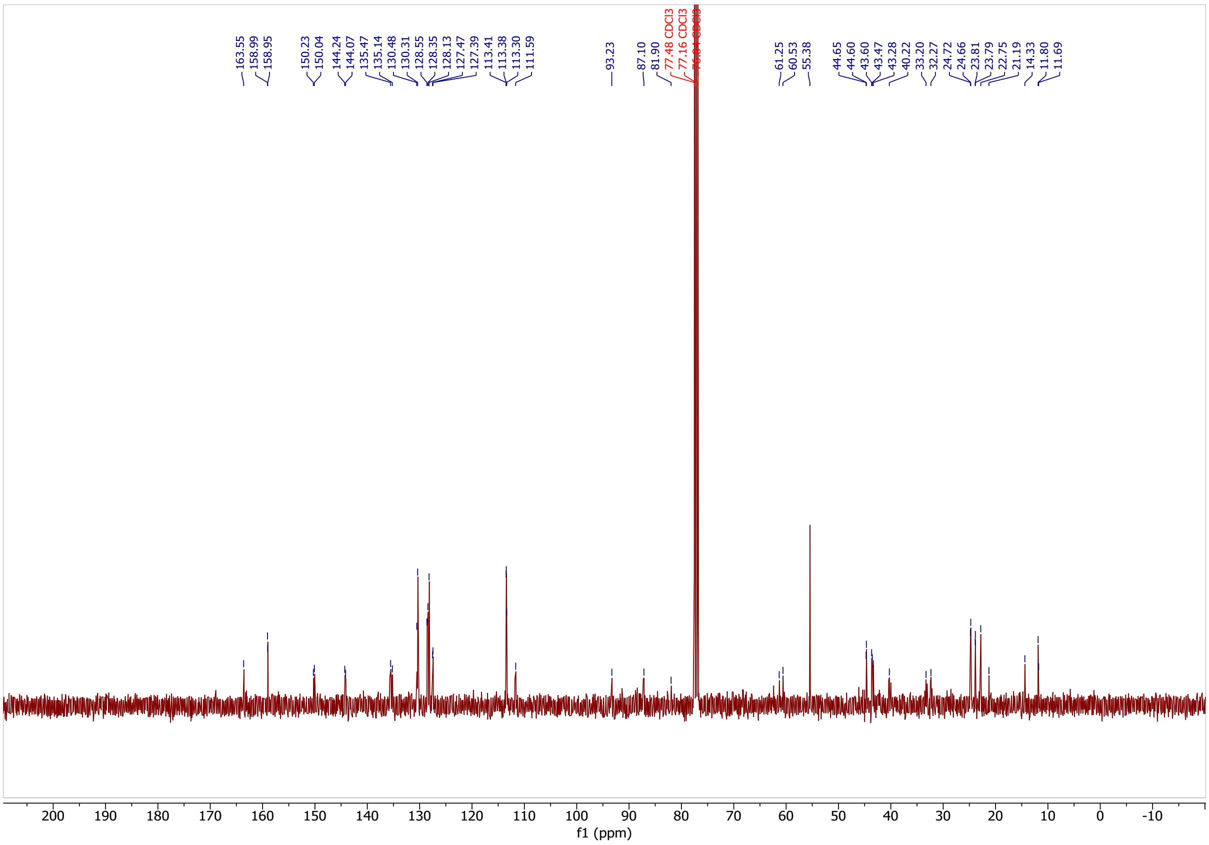

Supplementary Figure S163: $^{13}C\{^{1}H\}$ NMR (101 MHz, $CDCl_3$) spectrum of **20.**

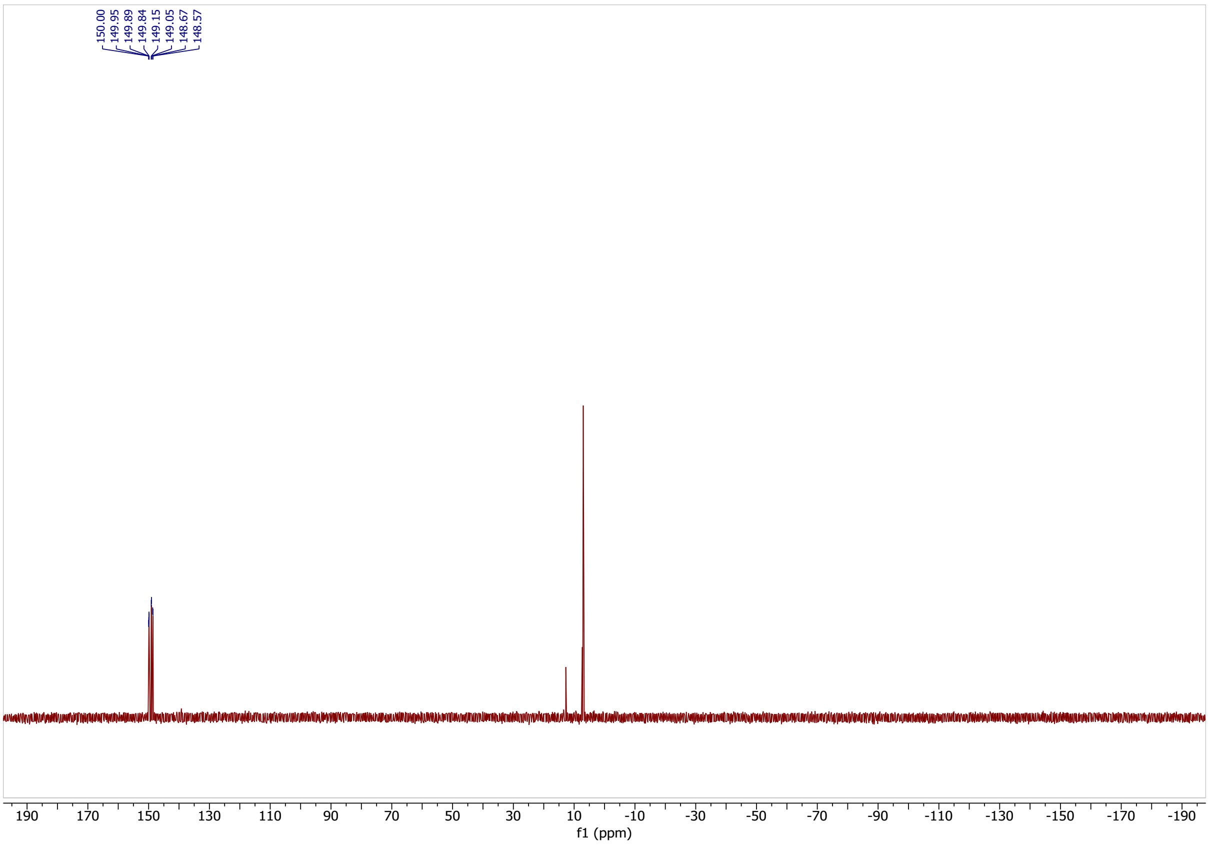

Supplementary Figure S164: ^{31}P NMR (162 MHz, $CDCl_3$) spectrum of **20.**

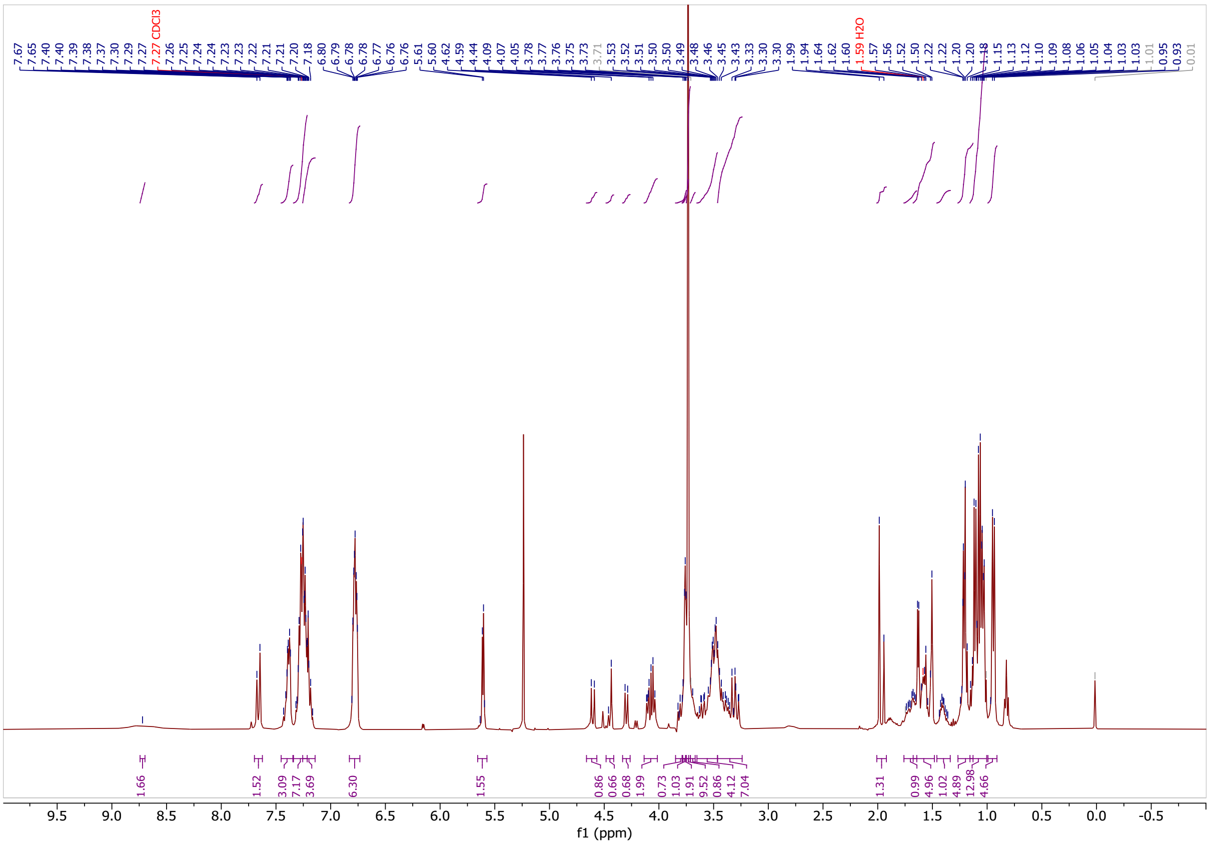

Supplementary Figure S165: ^{1}H NMR (400 MHz, $CDCl_3$) spectrum of **21.**

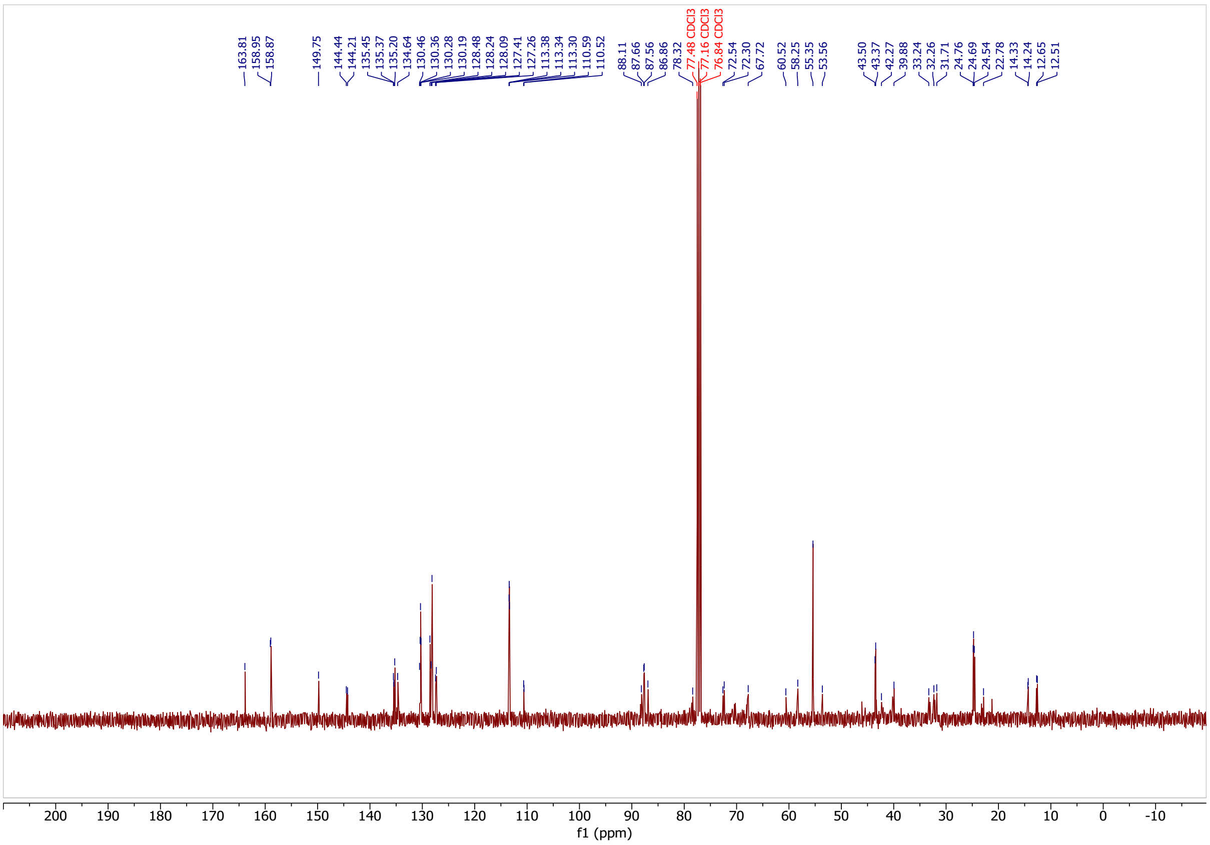

Supplementary Figure S166: $^{13}C\{^{1}H\}$ NMR (101 MHz, $CDCl_3$) spectrum of **21.**

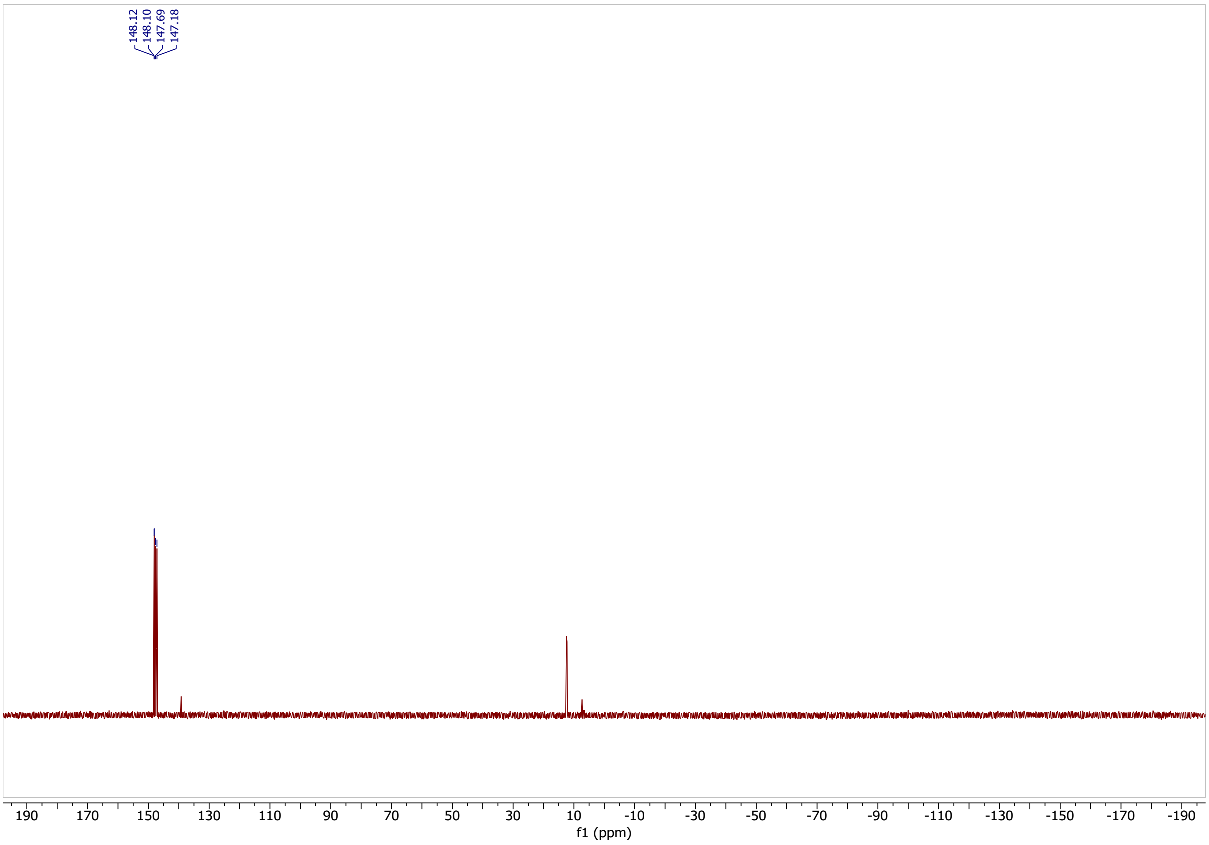

Supplementary Figure S167: ^{31}P NMR (162 MHz, $CDCl_3$) spectrum of **21.**

7.0 Selected Trityl Readings of Solid Phase Synthesis

1 2 3 4

Number from 3'	Raw	Avg Stepwise Yield %	Overall Yield %
2	283	100.0	100.0
4	304	100.0	100.0
8	321	100.0	100.0
12	296	98.9	88.2
15	323	100.0	100.0
16	303	99.1	87.6
17	305	99.2	87.6

LNA$_2$-OMe$_2$

1 2 3 4

Number from 3'	Raw	Avg Stepwise Yield %	Overall Yield %
2	325	100.0	100.0
4	306	98.0	94.2
8	341	100.0	100.0
12	319	99.0	89.7
16	364	100.0	100.0
17	383	100.0	100.0

LNA$_2$-MOE$_2$

1 2 3 4

Number from 3'	Raw	Avg Stepwise Yield %	Overall Yield %
2	281	100.0	100.0
3	218	88.1	77.6
6	230	95.0	77.6
10	306	100.0	100.0
14	323	100.0	100.0
17	368	100.0	100.0

LNA$_4$-OMe$_4$

1 2 3 4

Number from 3'	Raw	Avg Stepwise Yield %	Overall Yield %
2	324	100.0	100.0
4	283	95.6	87.3
8	320	98.1	87.3
12	293	98.8	87.3
16	356	100.0	100.0
17	379	100.0	100.0
18	369	98.3	74.7

LNA$_4$-MOE$_4$

1 2 3 4

Number from 3'	Raw	Avg Stepwise Yield %	Overall Yield %
2	327	100.0	100.0
4	295	96.6	90.2
8	320	98.5	90.2
12	285	98.8	87.2
16	345	100.0	100.0
18	338	98.9	82.6

LNA$_7$-OMe$_6$

1 2 3 4

Number from 3'	Base	Avg Stepwise Yield %	Overall Yield %
2	6	100.0	100.0
4	7	95.6	87.4
5	7	96.7	87.4
8	6	98.1	87.4
12	A	98.2	81.6
16	7	98.7	81.6
18	6	98.8	81.6

LNA$_7$-MOE$_6$

Supplementary Figure S168: Trityl monitor reading of the solid phase synthesis of oligonucleotides using the phosphoramidite monomers indicated.

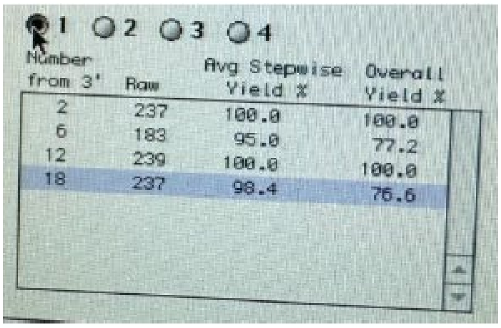

Number from 3'	Raw	Avg Stepwise Yield %	Overall Yield %
2	237	100.0	100.0
6	183	95.0	77.2
12	239	100.0	100.0
18	237	98.4	76.6

Pip-F_2

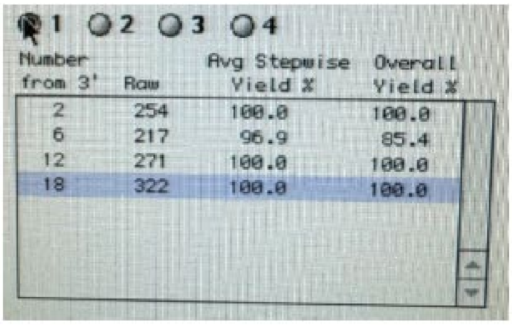

Number from 3'	Raw	Avg Stepwise Yield %	Overall Yield %
2	254	100.0	100.0
6	217	96.9	85.4
12	271	100.0	100.0
18	322	100.0	100.0

Pip-LNA_2

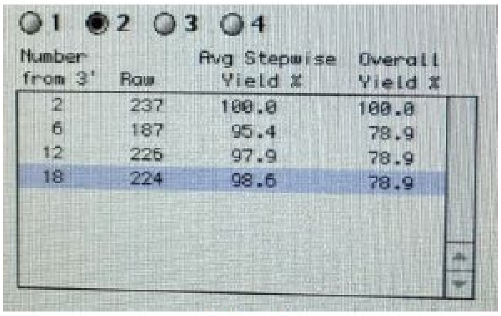

Number from 3'	Raw	Avg Stepwise Yield %	Overall Yield %
2	237	100.0	100.0
6	187	95.4	78.9
12	226	97.9	78.9
18	224	98.6	78.9

Pip-F_4

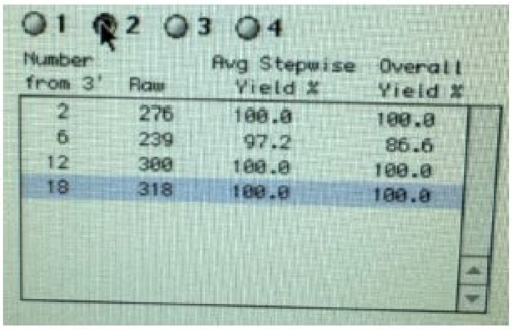

Number from 3'	Raw	Avg Stepwise Yield %	Overall Yield %
2	276	100.0	100.0
6	239	97.2	86.6
12	300	100.0	100.0
18	318	100.0	100.0

Pip-LNA_4

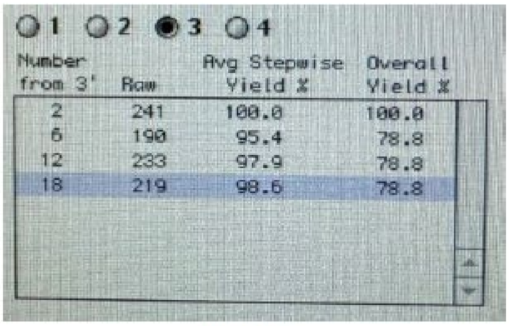

Number from 3'	Raw	Avg Stepwise Yield %	Overall Yield %
2	241	100.0	100.0
6	190	95.4	78.8
12	233	97.9	78.8
18	219	98.6	78.8

Pip-F_6

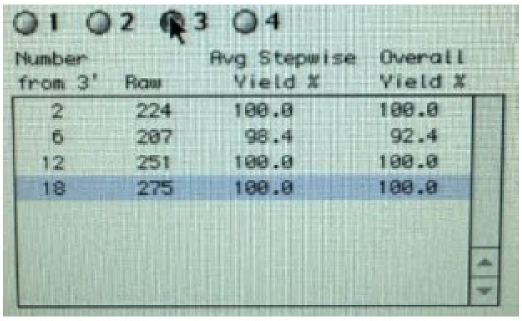

Number from 3'	Raw	Avg Stepwise Yield %	Overall Yield %
2	224	100.0	100.0
6	207	98.4	92.4
12	251	100.0	100.0
18	275	100.0	100.0

Pip-LNA_6

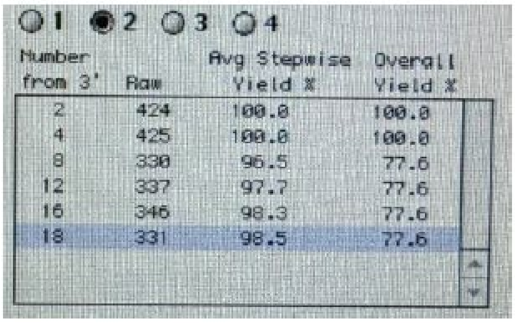

Number from 3'	Raw	Avg Stepwise Yield %	Overall Yield %
2	424	100.0	100.0
4	425	100.0	100.0
8	330	96.5	77.6
12	337	97.7	77.6
16	346	98.3	77.6
18	331	98.5	77.6

LNA_7-OMe_6-PO

Supplementary Figure S169: Trityl monitor reading of the solid phase synthesis using the phosphoramidite monomers indicated.

8.0 Detailed Chemical Structures of Oligonucleotides

OMe_2

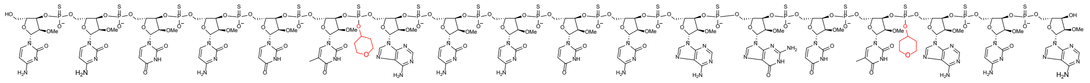

OMe_4

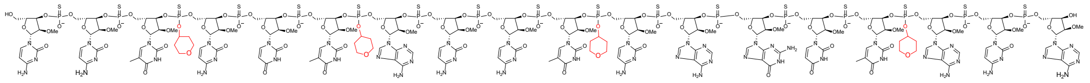

OMe_6

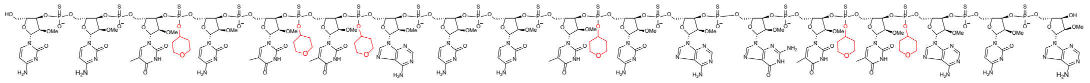

MOE_2

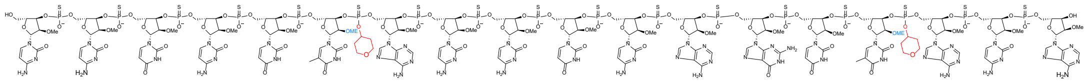

MOE_4

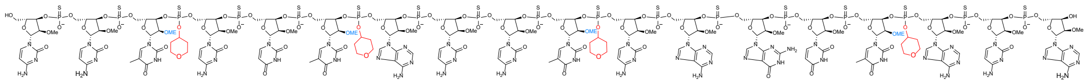

MOE_6

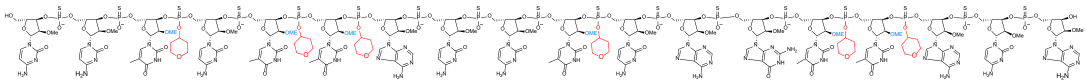

LNA_2-OMe_2

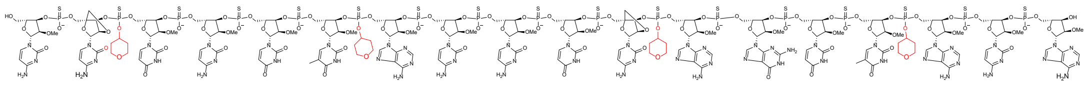

LNA_4-OMe_4

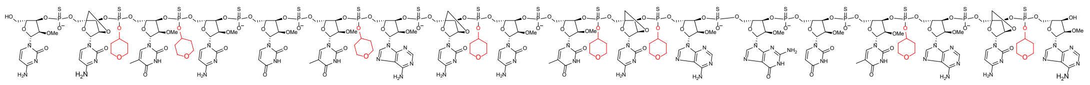

LNA_7-OMe_6

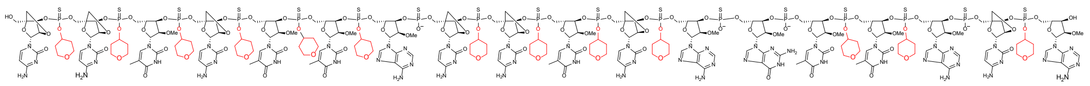

2'MOEPS+

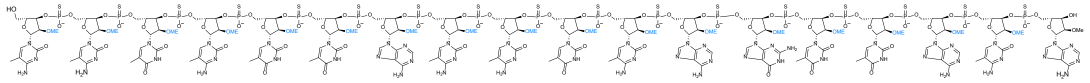

LNA_2-MOE_2

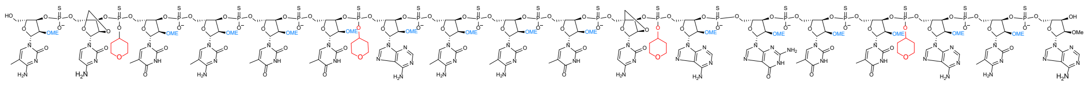

LNA_4-MOE_4

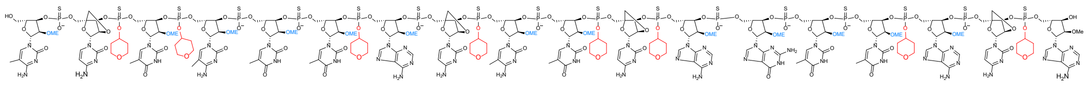

LNA_7-MOE_6

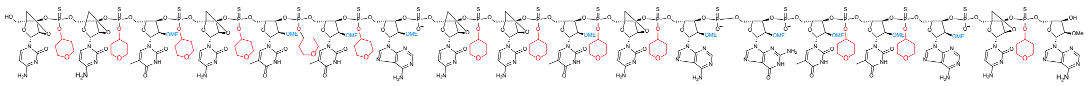

LNA_4

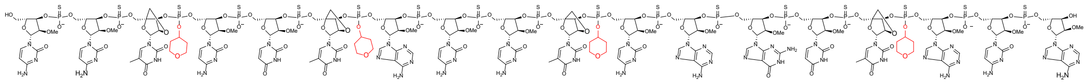

Continued

F_2

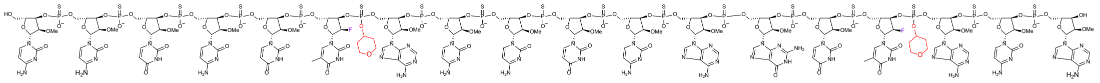

F_4

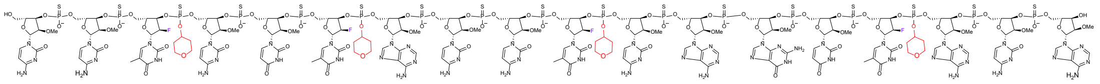

F_6

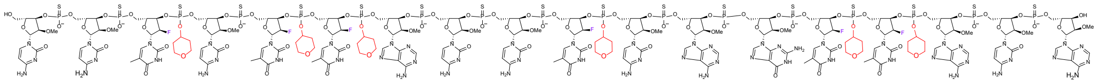

LNA_2-F_2

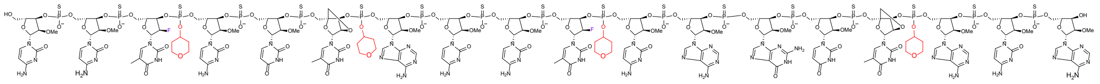

LNA_2-F_4

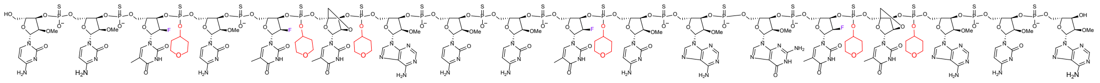

LNA_3-F_3

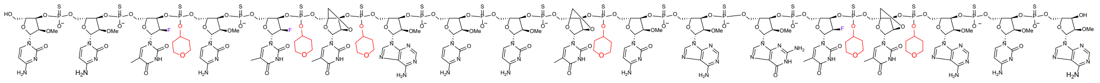

LNA_4-F_2

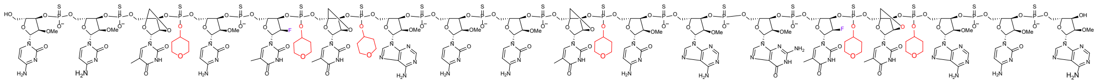

Pip-LNA_2

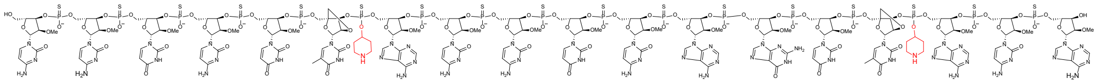

Pip-LNA_4

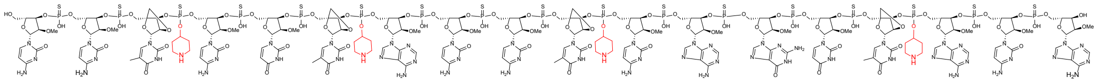

Pip-LNA_6

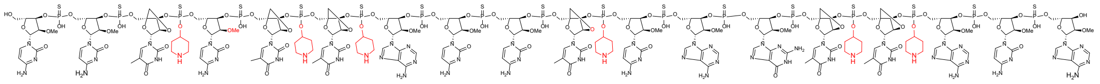

Pip-F_2

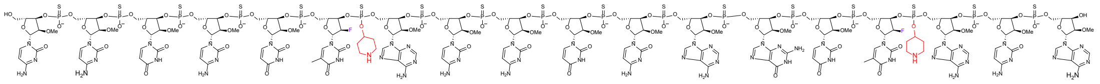

Pip-F_4

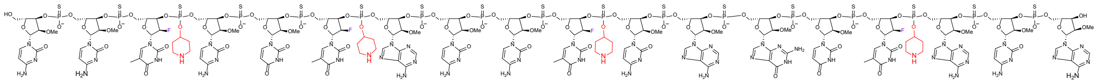

Pip-F_6

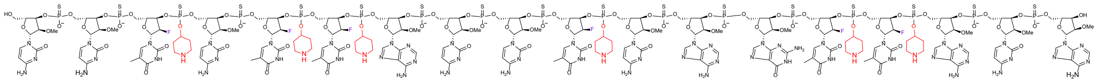

LNA_{11}-OMe_6-PO

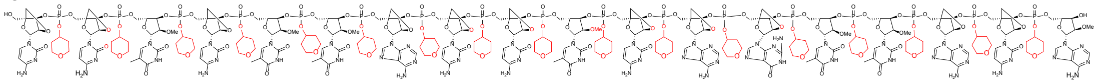

Continued

Pal-OMe$_4$

Pal-MOE$_4$

Pal-LNA$_4$

Pal-LNA$_4$-PS

Pal$_2$-LNA$_4$

Pal-Pip-LNA$_4$

Pal-Pip-F$_4$

Pal-LNA$_2$-OMe$_2$

Pal-LNA 2(C)-PS

Pal-LNA$_2$-F$_2$

Pal-LNA$_2$-F$_2$-PS

Pal-Pip-LNA$_2$

Pal-LNA$_2$-PS

Pal-F$_2$-PS

Pal-MOE$_2$-PS

Pal-2'MOEPS+

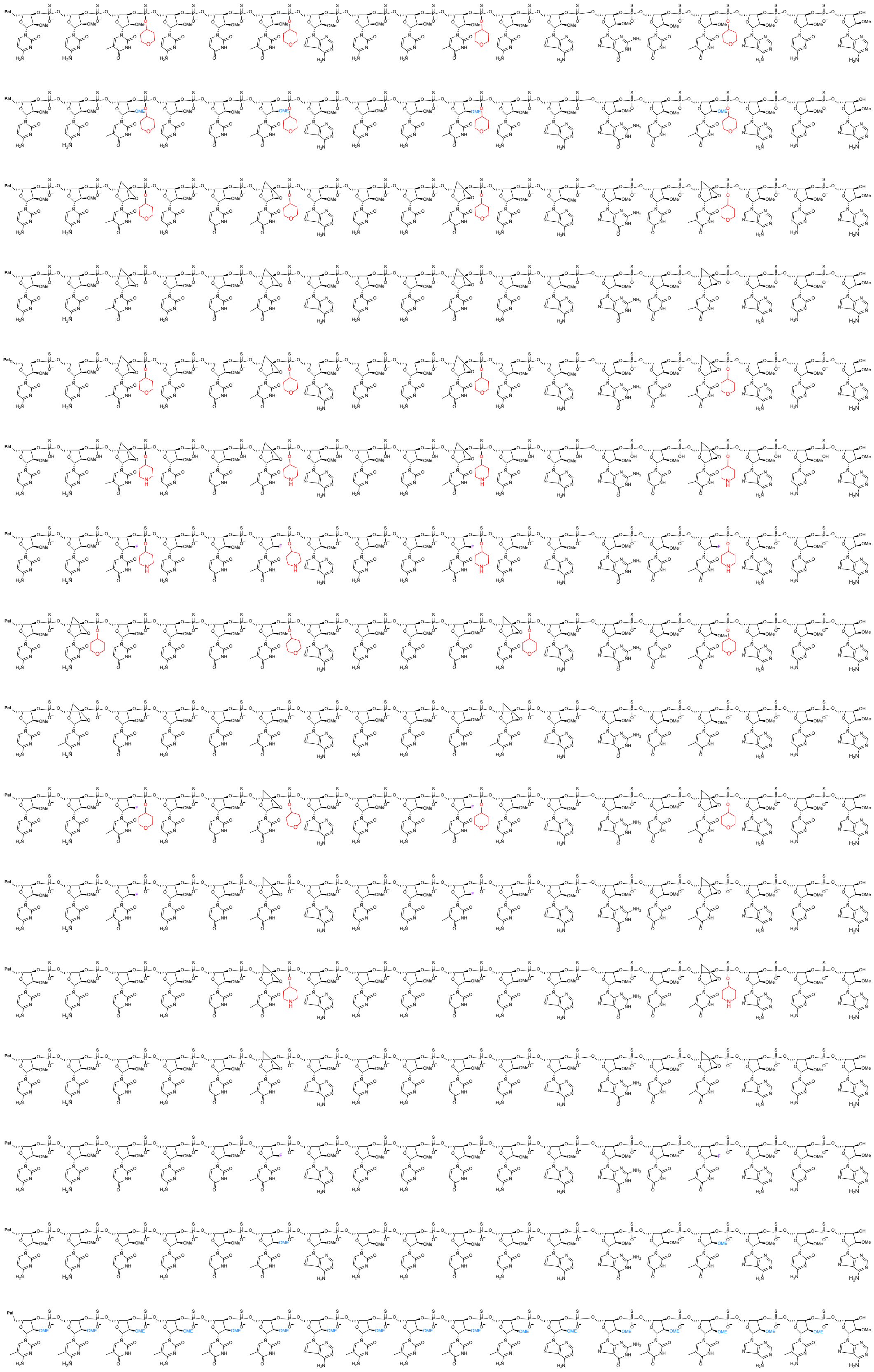

Supplementary Figure S170: Chemical structure of oligonucleotides. Pal: palmitate.

9.0 References

1. Dhara, D., Hill, A.C., Ramesh, A., Wood, M.J.A., El-Sagheer, A.H. and Brown, T. (2024) Synthesis, Biophysical and Biological Evaluation of Splice-Switching Oligonucleotides with Multiple LNA-Phosphothiotriester Backbones. *J. Am. Chem. Soc.*, **146**, 29773–29781.
2. Kang, S.-H., Cho, M.-J. and Kole, R. (1998) Up-Regulation of Luciferase Gene Expression with Antisense Oligonucleotides: Implications and Applications in Functional Assay Development. *Biochemistry*, **37**, 6235–6239.
